# Highly selective α-aryloxyalkyl C–H functionalisation of aryl alkyl ethers†

**DOI:** 10.1039/d2sc04463c

**Published:** 2022-10-20

**Authors:** Jonathan D. Bell, Iain Robb, John A. Murphy

**Affiliations:** Department of Pure and Applied Chemistry 295 Cathedral Street Glasgow G1 1XL UK john.murphy@strath.ac.uk

## Abstract

We report highly selective photocatalytic functionalisations of alkyl groups in aryl alkyl ethers with a range of electron-poor alkenes using an acridinium catalyst with a phosphate base and irradiation with visible light (456 nm or 390 nm). Experiments indicate that the reaction operates *via* direct single-electron oxidation of the arene substrate ArOCHRR′ to its radical cation by the excited state organic photocatalyst; this is followed by deprotonation of the ArOC–H in the radical cation to yield the radical ArOC˙RR′. This radical then attacks the electrophile to form an intermediate alkyl radical that is reduced to complete the photocatalytic cycle. The oxidation step is selective for activated arenes (ArOR) over their non-activated counterparts and the subsequent deprotonation of the methoxy group affords the α-aryloxyalkyl radical that leads to a wide range of functionalised products in good to excellent yield.

## Abstract 2

We report highly selective photocatalytic functionalisations of alkyl groups in aryl alkyl ethers with a range of electron-poor alkenes using an acridinium catalyst with a phosphate base and irradiation with visible light (456 nm or 390 nm).

## Graphical abstract

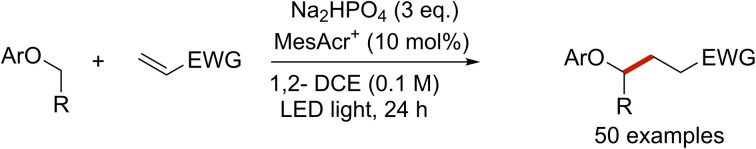

## Introduction

Aryl alkyl ethers occur widely in both natural products and pharmaceutical compounds. From the top 200 small molecule pharmaceuticals by retail sales in 2020 the aryl alkyl ether moiety was present in APIs such as: aripiprazole, empagliflozin, metoprolol and tamsulosin.^1^ Aryl alkyl ethers are economical building blocks and therefore they are key targets for functionalisation.^2–13^

**Scheme 1:**
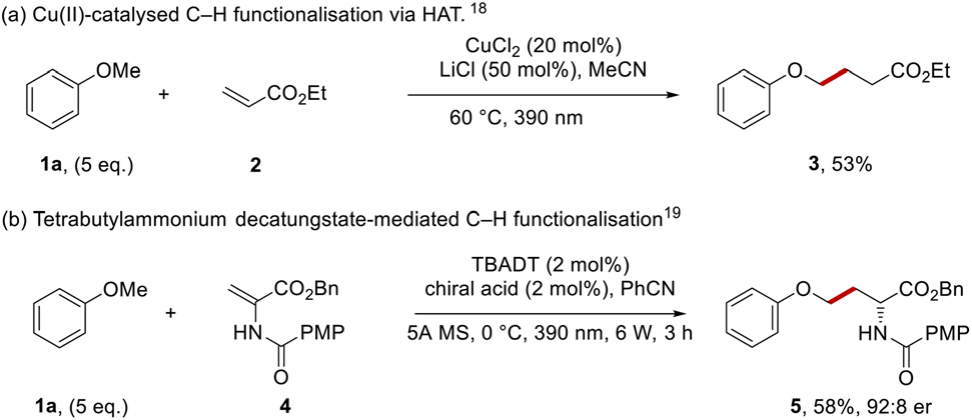
HAT pathways of anisole functionalisation.

For our purposes, a highly selective method for functionalisation was required. Recent developments have seen very reactive hydrogen atom transfer (HAT) agents,^14,15^ notably Cl atoms (Cl˙) or oxyl radicals (RO˙) used to form radicals by abstraction of ArOC–H hydrogen atoms. Thus, Barriault *et al.* formed Cl˙ from the chloride counterion of photocatalytic iridium chloride salts and these radicals mediated the coupling of anisole with dimethyl maleate.^16^ In a related approach, Wu *et al.* used chlorine atoms, generated by photooxidation of chloride ions in hydrogen atom transfer (HAT) chemistry for the functionalisation of aryl alkyl ethers.^17^ More recently, the group of Rovis used a copper(ii) catalyst and lithium chloride (Scheme 1a) to facilitate couplings with anisole 1a.^18^ Under the reaction conditions, coordination of chloride ion to CuCl_2_ gave the photoactive CuCl_3_^−^ complex; irradiation with light generated a chlorine atom that abstracted an H-atom from anisole to form the phenoxymethyl radical for Giese coupling with ethyl acrylate 2 to form 3.

A recent example using oxyl radicals in an enantioselective manner was reported by Wang *et al.*, where photoactivated tetrabutylammonium decatungstate (TBADT, an oxyl radical)^19^ was the HAT agent (Scheme 1b). In the presence of a chiral catalyst, this converted amidoesters, *e.g.*4, into protected amino acids such as phenyl ether 5.

Both chlorine atoms^20^ and oxyl radicals^21,22^ are highly reactive entities, capable of abstracting an H atom from a wide variety of C–H bonds and indeed both types of radicals react very successfully with many groups besides aryl alkyl ethers. From classical radical chemistry, chlorine atoms are used on an industrial scale to functionalise aliphatic hydrocarbons.^20^ Because they are so reactive, they often lack selectivity, and this means that, in deployment with complex molecules, their use may be limited, although recent advances have shown that reactivity of Cl atoms can sometimes be modulated.^23,24^

**Scheme 2:**
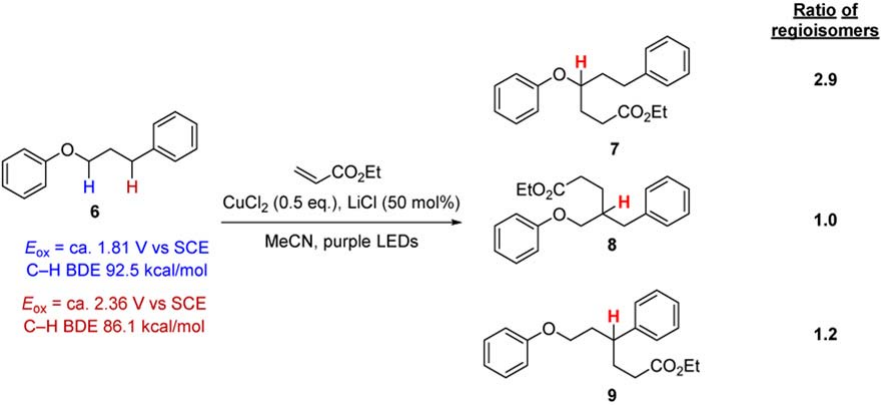
Testing for selectivity in C–H functionalisation with a HAT protocol.

We were concerned that these reaction conditions might not be sufficiently selective in functionalising aryl alkyl ether moiety in more complex molecules. To probe this point, 3-phenoxypropylbenzene 6 was selected which has multiple hydrogen atoms that might be attacked by Cl atoms, as the PhOC–H bonds and the benzylic PhC–H bonds are weak (*i.e.* abstraction will lead to quite stabilised radicals) while the C–H bonds of the central CH_2_ group should be stronger. Applying the conditions in Scheme 2 resulted in the formation of three regioisomers 7–9 that could not be separated even with preparative HPLC in a ratio of (1.0/1.2/2.9, see ESI†). Accordingly, this approach was not at all selective based on C–H bond strength. We also examined these conditions with dimethyl fumarate and benzylidenemalononitrile and in these cases, all three regioisomers were formed too.

In view of this result, it was clear that a different approach was required and we selected a tailored redox activation of ArOC–H bonds in aryl alkyl ethers, using an acridinium salt photocatalyst, and taking advantage of the inherent differences between the redox potentials between the phenoxy (*E*_ox_ = +1.81 V *vs.* SCE for anisole) and phenyl ring (*E*_ox_ = +2.36 V *vs.* SCE) systems.^25^

**Scheme 3:**
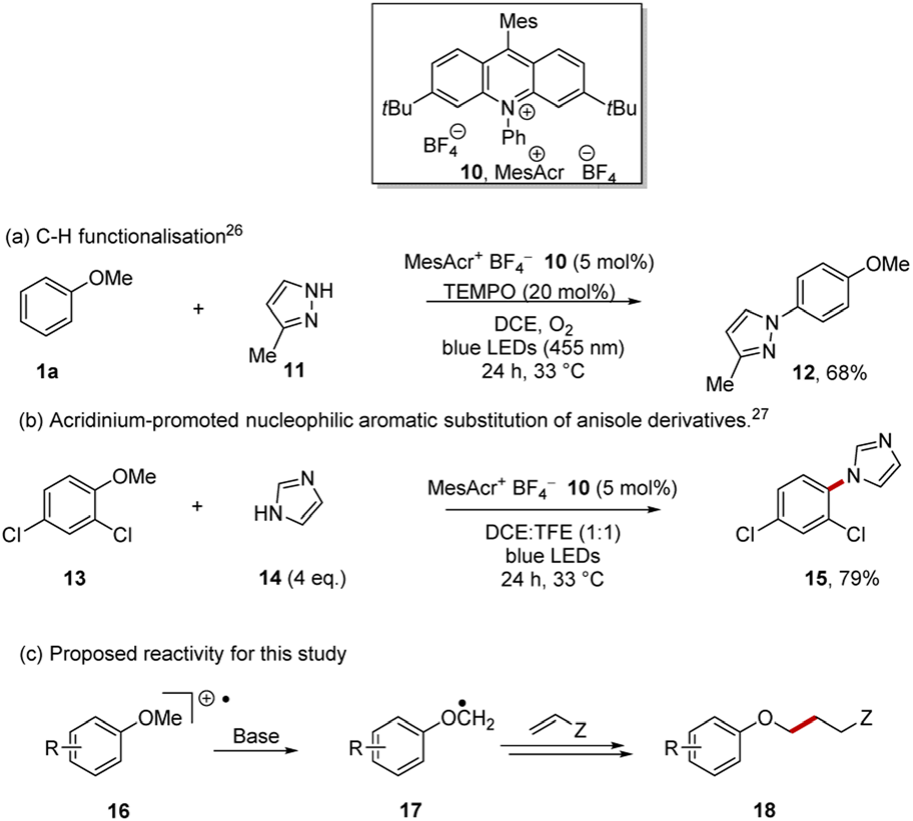
Direct aryl alkyl ether oxidation with acridinium catalyst 10.

Acridinium photocatalysts^27–29^ have become widely used and the mesitylacridinium salt 10 is the most popular, as alongside highly positive excited state reduction potentials, suitable for conversion of arene substrates to their radical cations, the substituents inhibit catalyst deactivation (Scheme 3).^30^ Following Nicewicz *et al.*, the photocatalyst 10 can be accessed in four steps from commercially available starting materials.^31^

Direct oxidation of arenes to their radical cations has been achieved with 10, leading principally to two types of reactions:^32^ (i) C–H functionalisation reactions of arenes as shown in the preparation of 12 (Scheme 3a)^26,33–37^ and (ii) substitution of an alkoxy or halo substituent on the aryl ring of the radical cations of arenes by nucleophiles as exemplified by the formation of 15 (Scheme 3b).^27,38–42^ In both these cases, the aryl ether *e.g.* anisole 1a is oxidised to its radical cation 16 by the excited state, MesAcr^+^*, and the ring of the arene radical cation is then attacked by a nucleophile.

More recently, applications following deprotonation of radical cations have been reported in alkyl thioethers and in benzylic systems.^43–45^ In our case, following oxidation of the aryl ether to its radical cation 16, deprotonation was planned to give the desired radical 17. Giese reactions should provide suitable traps for these radicals, affording product 18.^46^

## Results and discussion

The photoredox functionalisation reaction was initially investigated with anisole (1a) as the aryl alkyl ether, dimethyl fumarate 19 as the electrophile, disodium hydrogen phosphate as the base, 1,2-dichloroethane (DCE) as solvent and with irradiation with blue light (blue LEDs, Kessil Lamp, 456 nm). This was successful, affording product 20a (81%) as shown in Scheme 3. An extensive substrate scope was completed to gain further understanding of this transformation. Aryl methyl ethers were studied that had functional groups bound to the aromatic ring. 4-Fluoroanisole and 4-chloroanisole afforded diesters 20b and 20c in 83% and 56% yield, respectively. A ketal substrate gave 20d in poor yield (45%), perhaps due to an electron-withdrawing effect of the ketal although alternative conditions later improved this yield (see below). *m*-Dimethoxybenzene gave 20e (38%) and, again, alternative conditions later gave an improved yield.

Variation of the alkyl chain of aryl alkyl ether afforded satisfying results. When ethyl phenyl ether was used as substrate, this produced 20g in 98% isolated yield and hexyl phenyl ether gave 20h in 76% isolated yield. The oxidation of benzyl phenyl ether results in the ready formation of a radical adjacent to the oxygen atom and thus diester 20i was produced in 93% yield. Phthalimide, acetate and benzoate substituents were each compatible with the transformation as seen in 20j–20l (74–100%).

This methodology was then explored in the construction of highly sterically congested ethers 20m–20o from isopropyl, cyclobutyl and cyclopentyl phenyl ethers. The successful synthesis of these compounds was achieved in isolated yields of 63–81%.

3-Methylanisole was chosen as a substrate to investigate the selectivity of the reaction, as the radical cation might be deprotonated on the ArMe or on the ArOMe groups leading to C–C bond formation at two different sites. In fact, the reaction resulted solely in the formation of the anticipated diester 20p in 71% yield. 2-Methylanisole (see ESI†) led to a mixture of regioisomers with C–H functionalisation occurring both at the benzylic and methoxy positions in an approx. 1 : 1 ratio. Meanwhile, 4-methylanisole underwent functionalisation at the benzylic position to give 20q (67%) and there was no functionalisation at the methoxy group.^47^

To test the selectivity in the presence of other aryl systems, 3-phenoxypropylbenzene 6 was selected as substrate. While the methylanisole substrates above featured both ArOC̲–H̲ and ArC̲–H̲ bonds associated with the same aryl ring, in this case, the C–H bonds were associated with a less activated aromatic ring. Crucially, the reaction of 6 with dimethyl fumarate resulted in diester 20r (74%) as sole product and no other regioisomers were detected. This reaction showed that a benzylic C–H position must be conjugated to an aryl group that is activated (*e.g.* by an alkoxy substituent) to compete for functionalization, and this was also observed with 4-methylbenzyl phenyl ether as diester 20s (93%) was isolated as sole product. These two reactions highlight the exceptional regioselectivity this reaction has over previously developed HAT approaches.

Finally, the more easily oxidised aryl ring in ArSC̲–H̲ outcompeted ArOC̲–H̲, as seen when 4-methoxythioanisole resulted in diester 20t (76%), with no functionalisation occurring at the methoxy C–H.

To illustrate the functionalisation with an alternative electrophile, anisole 1a was reacted with a range of substituted benzylidenemalononitriles, and this gave dinitriles 22a–22h (Scheme 5). There was no functionalisation of the tolyl group when 4-methylbenzylidenemalononitrile was employed as the Giese acceptor, and 22b (99%) was the only detected product. The halogenated benzylidenemalononitriles all resulted in efficient C–C formation, with compounds 22c–22f being isolated in high yields (86–97%).

A *p*-methoxy substituent on the benzylidenemalononitrile showed no competing reactivity and this resulted in dinitrile 22g (87%) as the only product. Even a *p*-nitroaryl substituent, (which often leads to problems in reactions that feature electron transfer)^48,49^ on the malononitrile resulted in 22h being isolated, albeit in lower yield (39%). The reaction of these alkylidene malononitriles with functionalised aryl alkyl ethers was investigated and this was successful too, as seen in the formation of dinitriles 22i–22r (68–100%). Just as in the reactions with dimethyl fumarate, sterically congested ethers were formed efficiently from isopropyl phenyl ether, with ethers 22s–22u (67–80%) being produced.

Examples of reactions with other electrophiles arising through coupling of anisole with a vinylidene diphosphonate and with a vinylidene diketone giving products 23 and 24 in 78% and 73% respectively are also shown.

**Scheme 4:**
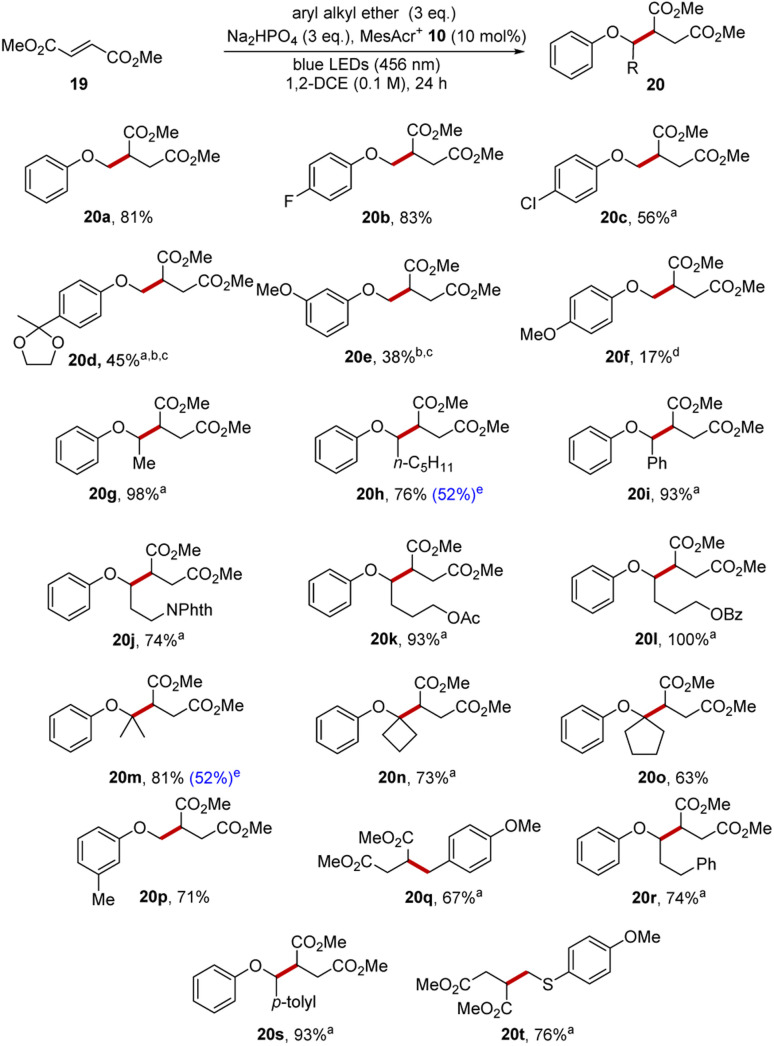
Products from reaction with dimethyl fumarate. All yields are isolated yields, unless indicated. ^a^Denotes that reverse stoichiometry was used, with the ether being used as limiting reagent and the alkene being used in excess. ^1^H-NMR yield with internal standard. ^c^See later in this paper. ^d^This reaction required the use of undried 1,2-dichloroethane when dried solvent was used, this resulted in a lower yield of product. ^e^Used aryl alkyl ether (1 eq.).

**Scheme 5:**
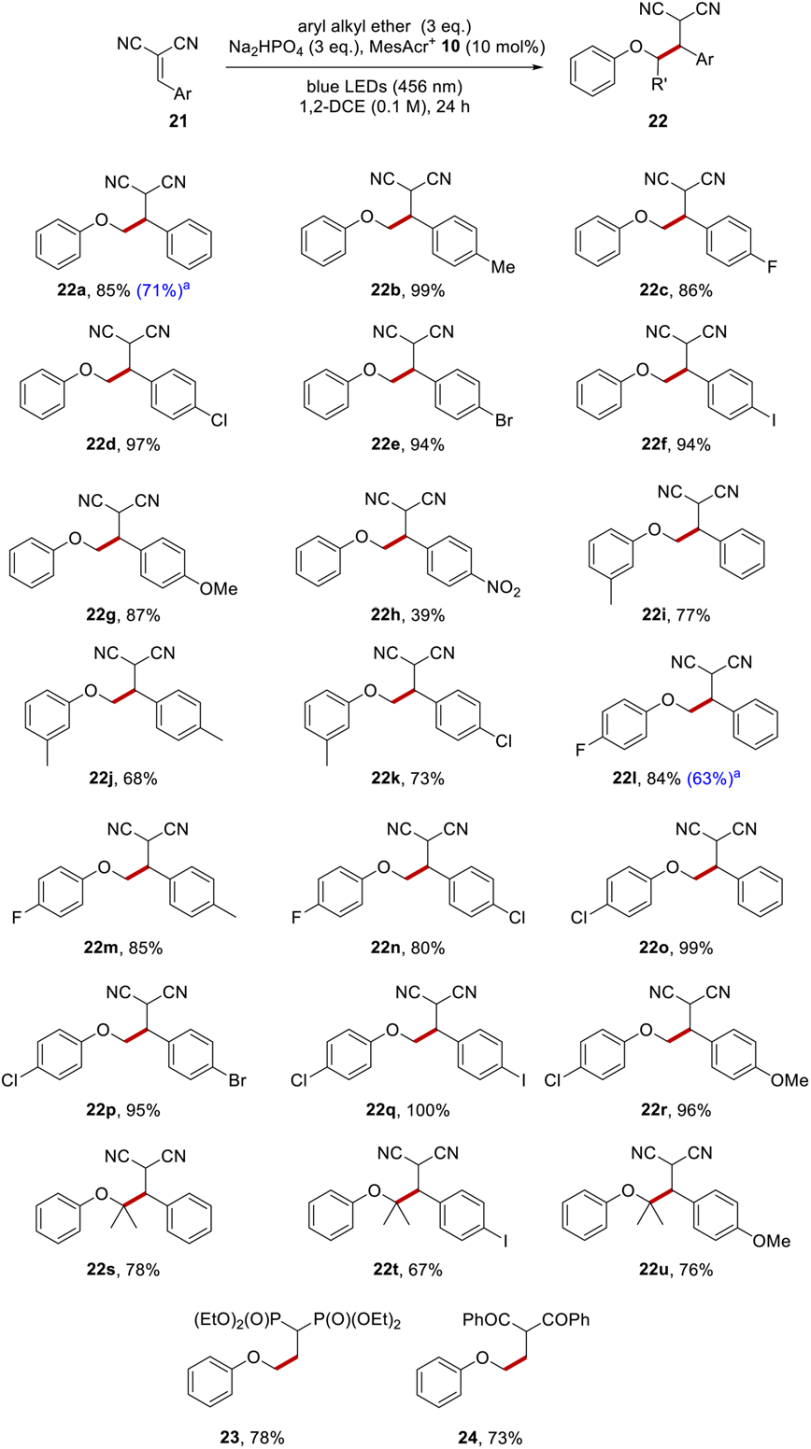
Aryl alkyl ether functionalisations with benzylidene-malononitrile. All yields are isolated yields after purification by flash chromatography. ^a^Used aryl alkyl ether (1 eq.).

To date, reactions had been carried out using aryl alkyl ether (3 eq.). To assess the effect of decreasing the amount of aryl alkyl ether to 1 eq., four substrates were examined which gave rise to the following products (20h, 20m, 22a and 22l). Useful yields (52%, 52%, 71% and 63%) respectively were still formed under the revised conditions (see Schemes 4 and 5 for a comparison of yields under the different conditions).

All the experiments above were consistent with the proposal in Scheme 3c, where the aryl alkyl ether radical cation was formed as an intermediate and then deprotonated to form the desired α-aryloxyalkyl radical 17. However, recent reports from Alexanian *et al.* under defined conditions showed that the combination of a photocatalyst with a phosphate base can alternatively operate through HAT^50^ or through proton-coupled electron transfer (PCET)^51^ pathways. A HAT pathway was noted in the functionalisation of alkanes with MesAcr^+^ and tripotassium phosphate in hexafluoroisopropanol.^50^ Here a highly reactive phosphate-derived oxyl radical was the key intermediate. In the PCET case, functionalisation of alkenes was achieved with an iridium photocatalyst and a monoanionic phosphate base in CH_2_Cl_2_. In view of these reports, we proposed to clarify whether HAT or PCET mechanisms were components of our transformation, or whether the reaction profile was simply consistent with the mechanism proposed in Scheme 3c.

**Scheme 6:**
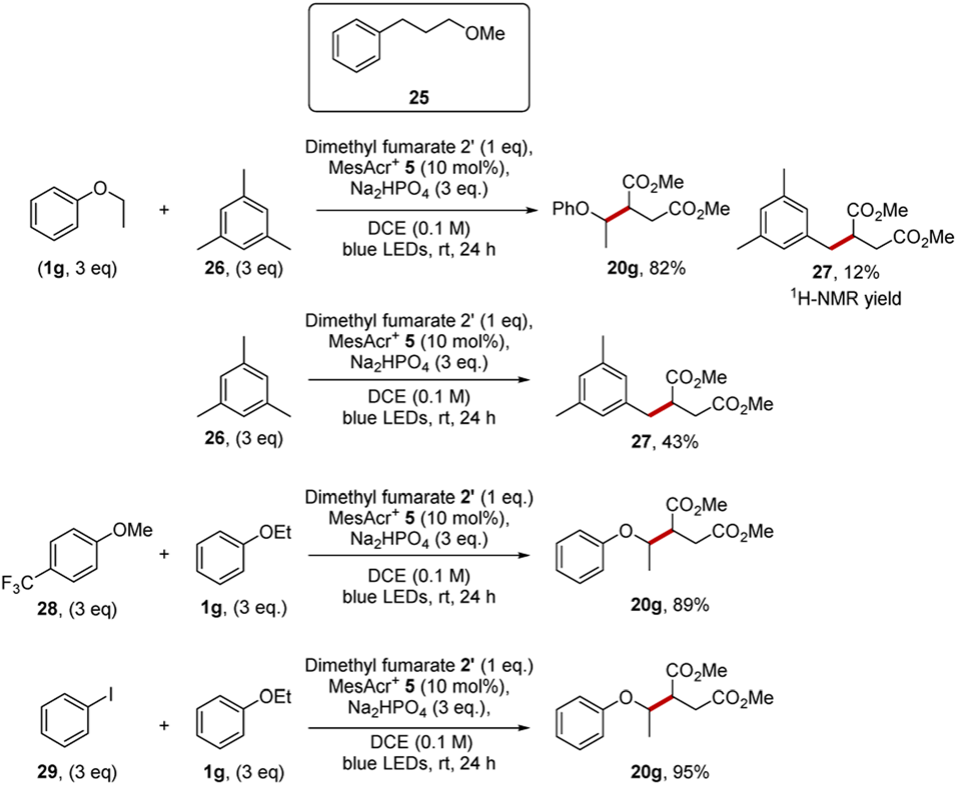
Mechanistic investigations conducted.

A test for a HAT pathway was carried out with a compound that has three sites susceptible for HAT activation, 3-methoxypropylbenzene (25) (Scheme 6), as a substrate that could show HAT activation, but that could not show oxidative functionalisation *via* the aryl radical cation. Benzylic C–H bonds and C–H bonds adjacent to oxygen atoms both have low bond dissociation energies and are excellent targets for HAT methodologies, as for a HAT event to be thermodynamically favorable a stronger bond must be formed, and a weaker bond must be broken.^3^ Experimental measurements have shown that C–H bonds adjacent to oxygen atoms are quite weak (92.5 ± 2.0 kcal mol^−1^, α-C–H bonds in diethyl ether)^52^ and that the benzylic C–H bonds are weak too (86.1 kcal mol^−1^, ArC̲–H̲ bonds in propylbenzene).^53^ Therefore, ether 25 should be a good substrate for HAT functionalisation by a reactive phosphoryloxyl radical.^50^ At the same time, the oxidation potential of ether 25 is too high (cyclic voltammetry of methyl 3-phenylpropyl ether showed an oxidation potential of +2.64 V *vs.* NHE)^54^ to allow electron transfer to MesAcr^+^*. The reaction of candidate substrate 25 with dimethyl fumarate and MesAcr^+^ yielded no product and only starting materials were recovered (97% determined by NMR). The lack of reactivity implies that this substrate shows no evidence of HAT under our conditions. HAT or PCET pathways were not expected under our conditions as generally, these transformations benefit from polar media as highlighted by the cases where water is added to the reaction mixture for successful HAT reactions.^55,56^ Additional Stern–Volmer studies tested for HAT and PCET reactions; this was accomplished by testing for quenching of MesAcr^+^* 5 with disodium hydrogen phosphate and a mixture of ether 25 with Na_2_HPO_4_. There was no quenching of the catalyst with disodium hydrogen phosphate, and this makes a HAT reaction pathway unfeasible. Ether 25 was tested for quenching of MesAcr^+^* under basic and non-basic conditions. In both cases, there was no quenching of the catalyst indicative of no PCET reaction pathways operating.^57^

Further support for the single-electron oxidation followed by deprotonation mechanism as proposed in Scheme 3c, came from competition experiments that were performed, in each case, between a pair of substrates. If the reaction was occurring *via* single-electron oxidation of the substrate, it might be expected that, in a reaction mixture containing two substrates, the more easily oxidised substrate would be targeted by the photoactivated catalyst, resulting in preferential functionalisation of that substrate.

The first competition was between ethyl phenyl ether 1g (*E*_ox_ = +1.81 V *vs.* SCE for anisole)^25^ and mesitylene 26 (*E*_ox_ = +2.07 V *vs.* SCE).^25^ We have already reported in Scheme 4 that coupling ethyl phenyl ether with dimethyl fumarate 19 gave product 20g in 98% yield. Our studies now showed that mesitylene 26 was also sufficiently electron-rich to react with dimethyl fumarate 19 under our conditions and diester 27 (43%) was isolated. When a 1 : 1 mixture of the two substrates (each 3 equiv.) was reacted with dimethyl fumarate (1 equiv.) for 24 h, ethyl phenyl ether outcompeted mesitylene and a 6.9 : 1.0 ratio of the compounds 20g and 27 was obtained. The diester 20g was isolated in 82% yield and 27 formed in 12% yield. So, in this experiment, the more easily oxidised ethyl phenyl ether 1g outcompetes mesitylene 26 in quenching MesAcr^+^* and thus diester 20g is formed preferentially over 27. Additional Stern–Volmer experiments also corroborated the observations from the competition experiment. Thus, ethyl phenyl ether was a good quencher of the acridinium catalyst (55.7 M^−1^) but mesitylene was a poor quencher of MesAcr^+^* (6.4 M^−1^).

Similar selectivity was again seen in competition experiments of 1g with other organic compounds. When mixed with the competitor 4-(trifluoromethyl)anisole 28 or iodobenzene 29 (*E*_ox_ = +2.17 V *vs.* SCE),^25^ the formation of 20g proceed readily as both these potential competitors were more electron-poor than 1g. Finally, the quantum yield of the reaction between anisole, dimethyl fumarate with MesAcr^+^ was measured and a value of 0.04 was calculated; this suggests that no radical chain mechanism is operating for these reactions.

**Scheme 7:**
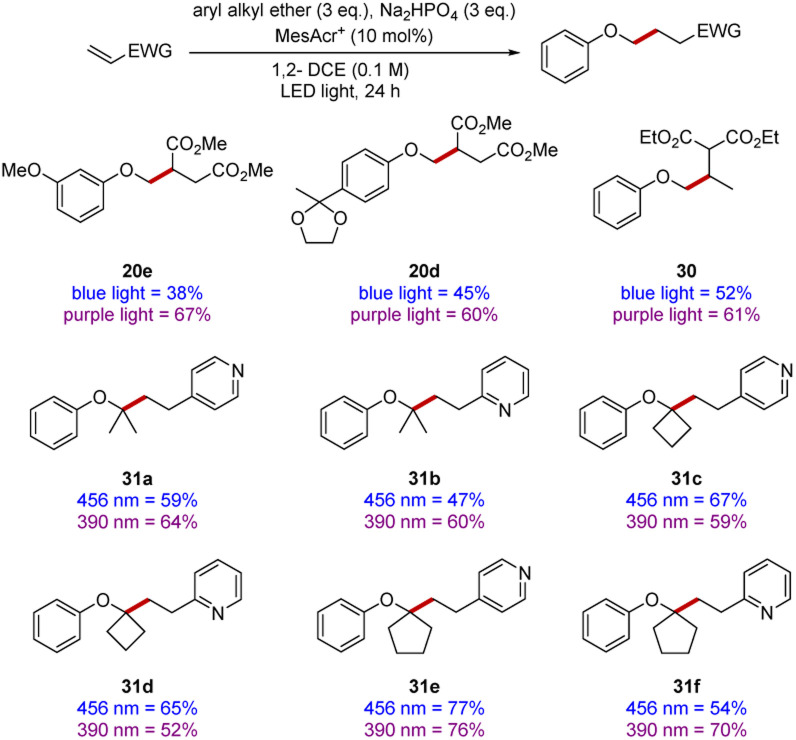
Electrophile scope at 390 nm *versus* 456 nm. All yields are isolated yields after purification by flash chromatography.

It was previously reported that different wavelengths of light had a significant impact upon the rates of reactions,^58,59^ although determining optimal wavelength was sometimes complex. Accordingly, we subjected a number of substrates (Scheme 7) to comparative experiments at 390 nm and 456 nm. Although almost all the yields in our earlier scoping studies were very good or excellent, a few yields were improved, such as for diesters 20d and 20e.

In the synthesis of diester 30, three equivalents of anisole were reacted with one equivalent of ethylidene diethyl malonate, the use of 390 nm irradiation resulted in diester 30 being isolated in 61% yield after 24 h. However, with 456 nm irradiation, 48 h were required to obtain a yield of 52% and significant amounts of starting electrophile remained (see ESI†).

To complete our study, 2- and 4-vinylpyridine were selected as they provide a convenient extension of the scope to heterocyclic electrophiles. Their reactivities were examined with both blue and purple light (see products 31a–31f), in some cases, use of different wavelengths did lead to a more efficient reaction but in some other cases this was not the case.

## Conclusion

To conclude, a photoredox reaction has been developed that functionalises aryl alkyl ethers like anisole 1a with electron-deficient alkenes to form adducts 18. Experiments suggest that the reaction operates *via* direct single-electron oxidation of the arene substrate by the excited state organic photocatalyst 10* followed by deprotonation of the radical cation 16 to yield the radical 17 responsible for attack on the electrophile.

The oxidation step is selective for activated arenes (ArOR) over their non-activated counterparts and the subsequent α-deprotonation of the alkoxy group leads to a wide range of functionalised products in good to excellent yield. The optimised reaction conditions facilitated the generation of a library of 50 compounds in good yields.

## Data availability

See ESI† for spectroscopic data in support of the compound structures and in support of the conclusions drawn in the paper.

## Author contributions

JDB, IR and JAM contributed to the drafting and revision of the paper, to the design of experiments and to the analysis and interpretation of data. JDB and IR performed the experimental work. JAM proposed and supervised the project.

## Conflicts of interest

There are no conflicts to declare.

## Supplementary Material

SC-013-D2SC04463C-s001
