# Supplementary material for: Highly selective α-aryloxyalkyl C–H functionalisation of aryl alkyl ethers
Source: Chem Sci. 2022 Oct 20;13(43):12921–6. doi: 10.1039/d2sc04463c (PMC9645420; doi:10.1039/d2sc04463c)
Supplement: SC-013-D2SC04463C-s001 [file SC-013-D2SC04463C-s001.pdf]

Electronic Supplementary Material (ESI) for Chemical Science.
This journal is © The Royal Society of Chemistry 2022

Supporting information for:

Highly Selective α-Aryloxyalkyl C-H Functionalisation of Aryl Alkyl Ethers

Jonathan D. Bell, Iain Robb, and John A. Murphy

Table of Contents

1. **General experimental details** **SI.2**
2. **Regioselectivity of the $CuCl_2$ catalytic system with 3-phenoxypropylbenzene** **SI.3**
3. **Optimisation** **SI.15**
4. **Experimental Procedures and Spectroscopic Data for all Compounds** **SI.17**
5. **Regioselectivity with *para-, meta- and ortho-*methylanisole** **SI.55**
6. **Reaction with 3-methoxypropylbenzene** **SI.56**
7. **Competition experiments** **SI.57**
8. **Stern-Volmer Experiments** **SI.60**
9. **Investigation into the use of different loadings of reagent** **SI.65**
10. **Quantum yield determination** **SI.67**
11. **Reactions with Purple light (390 nm) and Diethyl Ethylidenemalonate** **SI.71**
12. **Reactions with methyl acrylate in the Giese coupling reaction** **SI.73**
13. **^{1}H and ^{13}C NMR Spectra for all Compounds** **SI.77**
14. **References** **SI.154**

1. General experimental details

All reagents and starting materials were obtained from commercial sources and used as received. Dry 1,2-dichloroethane was either obtained from Acros Organics or distilled over CaH_2, other dry solvents were purified using a PureSolv 400 solvent purification system. The reagent tetramethylethylenediamine was dried over CaH_2 and distilled before use. All photocatalytic reactions were performed in an oven-dried 2–5 mL Kinesis KX Microwave Vial under an atmosphere of argon and were purged with argon for at least 5 minutes prior to irradiation. A Kessil® LED Photoreaction Light PR160L 456 nm was used as 456 nm light source in photocatalytic reactions and a Kessil® LED Photoredox Light PR160 390 nm was used as the 390 nm light source. Brine refers to a saturated solution of sodium chloride. Flash column chromatography was carried out using silica gel (Fisher matrix silica 60). Merck aluminium-backed plates pre-coated with silica gel 60 (UV254) were used for thin-layer chromatography and visualised by staining with $KMnO_4$. ^1H NMR and ^{13}C NMR spectra were recorded on a Bruker DPX 400 or 500 spectrometer with chemical shift values in ppm relative to residual solvent signals as the internal standard, for ^1H NMR spectra: $CHCl_3$ δ_H = 7.26 ppm, DMSO-d_5, δ_H = 2.50 ppm or CD_2HOD δ_H = 3.31 ppm. For ^{13}C NMR spectra $CDCl_3$ δ_C = 77.2 ppm DMSO-d_6 δ_C = 39.5 ppm and MeOD-d_4 δ_C = 49.0 ppm. Proton and carbon assignments are based on two-dimensional COSY, HSQC, HMBC, and DEPT experiments. IR spectra were recorded on Shimadzu 1 IRAffinity-1 instrument. Melting points were determined on a Reichert platform melting point apparatus. GC-MS data were recorded on Thermo Finnigan Polaris Q, mass range 50-650 Da. The column temperature was 320 °C, and the carrier gas was helium with a flow rate of 1 mL/min. The adsorbent was Crossbond® (0.25 µm) with column dimensions of 30 m x 0.25 mm. Results are reported as *m/z*. All samples were prepared in $CHCl_{3,}$ and electron ionisation (EI) was used as the ionisation method.

2. Regioselectivity of the $CuCl_2$ system with 3-phenoxypropylbenzene

Summary of findings

3-Phenoxypropylbenzene was used as substrate in a HAT reaction using a $CuCl_2$ catalyst following a previously reported procedure.[1] This reaction was performed with three different Giese acceptors: Ethyl acrylate, dimethyl fumarate and benzylidenemalononitrile. From each of these reactions, we observed that all three different regioisomers were produced.

Reaction between ethyl acrylate and 3-phenoxypropylbenzene with $CuCl_2$ and LiCl

The reaction was performed following a previously reported procedure.[1] An oven-dried microwave vial equipped with a magnetic stirrer bar was taken into a glovebox and copper (II) chloride, ultra dry, 99.995% (metal basis, 0.0200 g, 0.150 mmol), anhydrous lithium chloride (0.0064 g, 0.150 mmol), ethyl acrylate (0.0300 g, 0.300 mmol), 3-phenoxypropylbenzene (**6**, 0.318 g, 1.50 mmol) and anhydrous acetonitrile (1 mL) were added. The vial was sealed and taken outside of the glovebox. The reaction mixture was irradiated by purple light (390 nm) for 36 h. The reaction mixture was concentrated *in vacuo* and gave the crude product which was purified by flash chromatography using silica gel. Eluting with 10→20% diethyl ether in hexane gave 0.0397 g of material. Analysis of the reaction mixture indicated that all three possible regioisomers had formed and 3-phenylpropanal formed too.

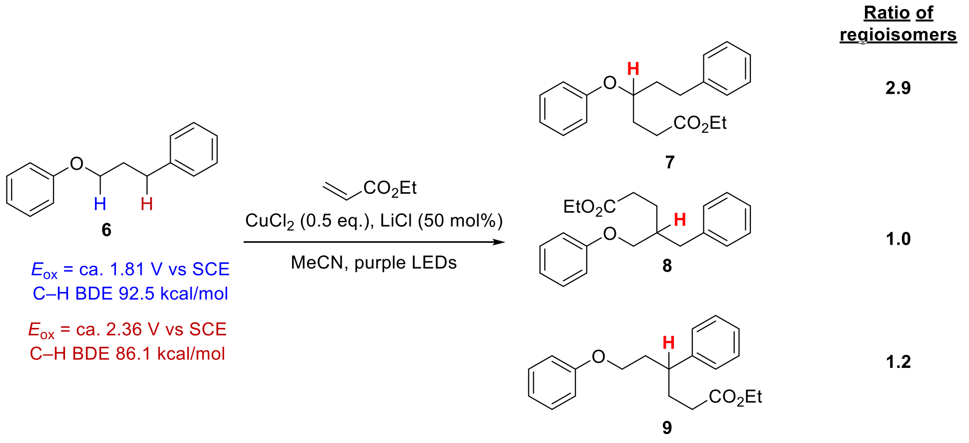

From the ^{1}H-NMR spectrum of the reaction mixture, a signal corresponding to 3-phenylpropanal (9.83 ppm) was observed and the presence of this compound was also confirmed by GCMS. ^{13}C-NMR indicated there were four compounds that contained quaternary phenyl carbon atoms between 140.0–143.6 ppm and these are due to the three regioisomers of ethyl ester product and 3-phenylpropanal. HSQC also provided conclusive evidence of the presence of three regioisomers with the presence of three methine cross-signals that were found at: (2.14, 39.6 ppm), (2.87, 41.8 ppm), and (4.35, 75.8 ppm). These three methine signals were determined to be the three red hydrogen atoms shown in the scheme above.

^{1}H-NMR of the reaction mixture after column chromatography

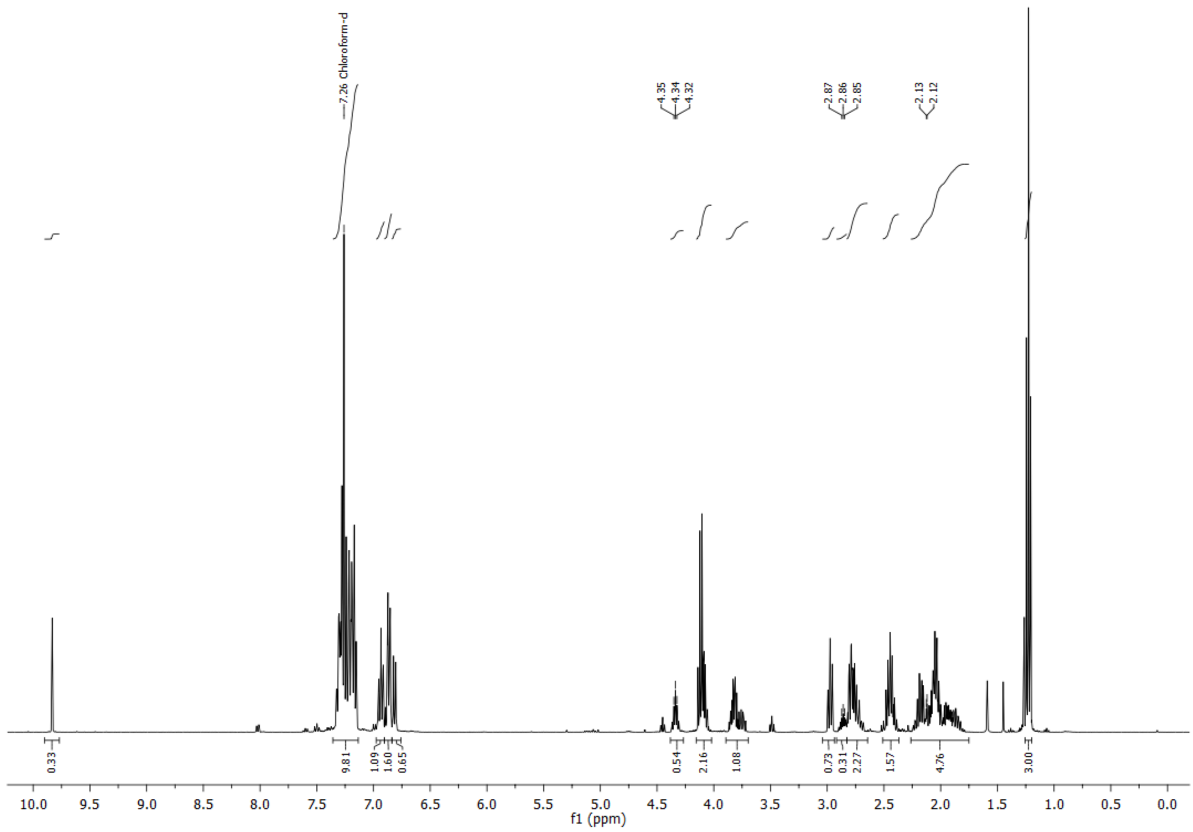

^{13}C-NMR of the reaction mixture after column chromatography

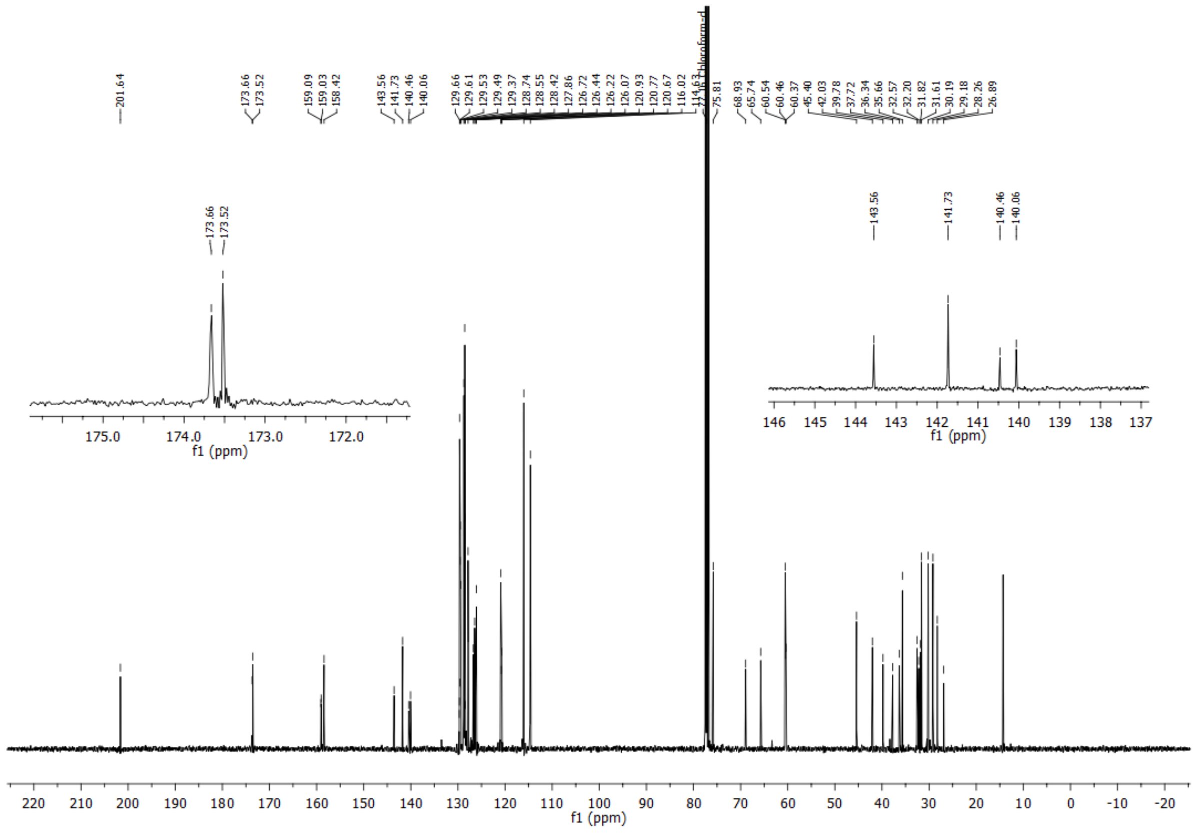

HSQC spectrum of the reaction mixture after column chromatography

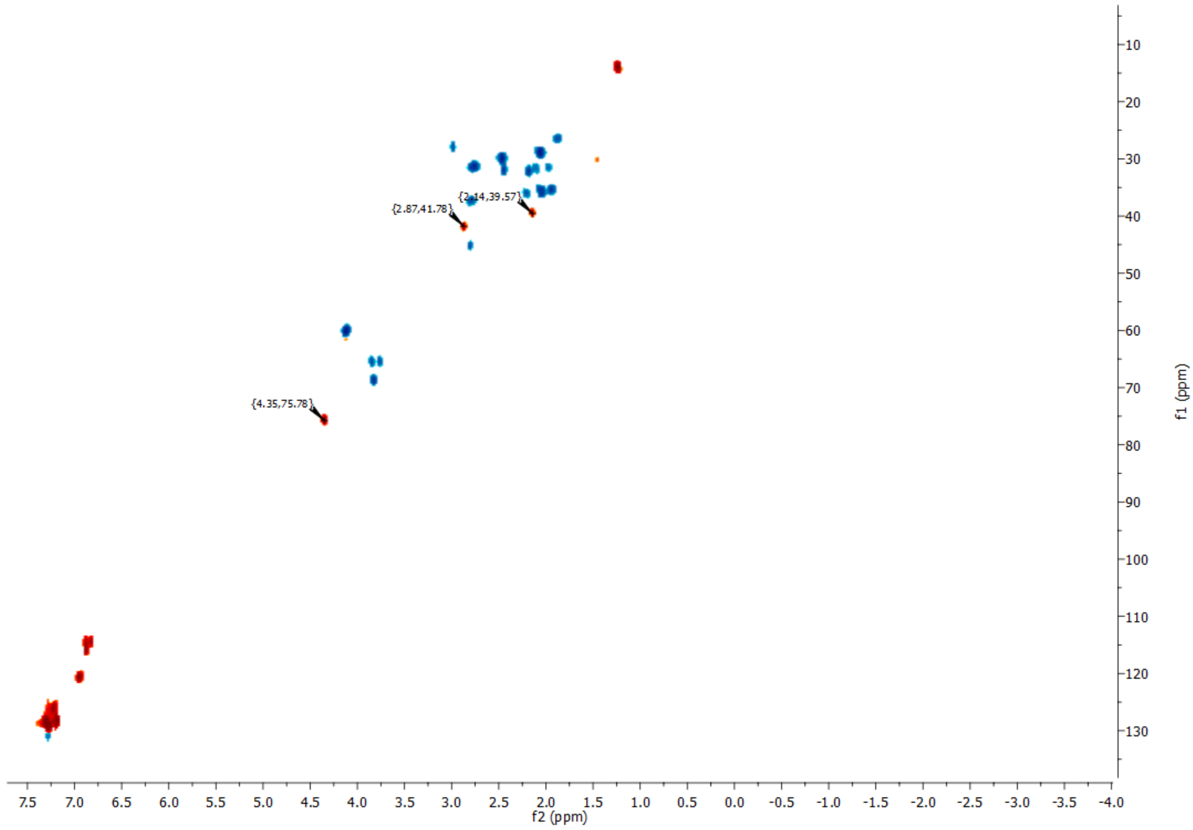

To fully evaluate the reaction mixture, the material was purified by reverse phase preparative HPLC and this was conducted using a Gilson preparative HPLC system of 322 pumps coupled to a 151 UV/Vis 163 spectrometer, 234 Autoinjector, and a GX-271 liquid handler using a Waters XBridge Prep C18 5μm 0BD 19 × 15 mm column at room temperature. Purification of this material was performed using the following gradient method over 10 min using a flow rate of 15 mL/min, with 0.1% TFA modifier and UV monitoring at 254 nm. For 0–1 minute 5% MeCN in H_2O was used to elute. From 1 to 3 minutes a linear gradient of 5% to 80% MeCN in water was applied and this was followed by a linear gradient of 80% to 95% between 3 to 6 minutes. Finally, a gradient of 95% to 5% MeCN in H_2O was implemented between 6 to 9 minutes. Analysis was conducted using Gilson Trilution software. The collected fractions were combined and concentrated *in vacuo* and this resulted in the return of 17.5 mg of material, NMR and GCMS analysis indicated there was no separation of the regioisomers. The material was studied with ^{13}C-NMR with two different pulse delay (d1) times (d1 = 2 s and d1 = 5 s) to calculate the ratio of regioisomers. From these two carbon spectra the ratio of regioisomers was determined to be 1.0/1.2/2.9. Analysis of the chromatogram from the GCMS indicated one regioisomer had a residence time of 15.91 minutes and the other two regioisomers had residence times of 16.06 and 16.09 minutes.

^{1}H-NMR of material after preparative HPLC

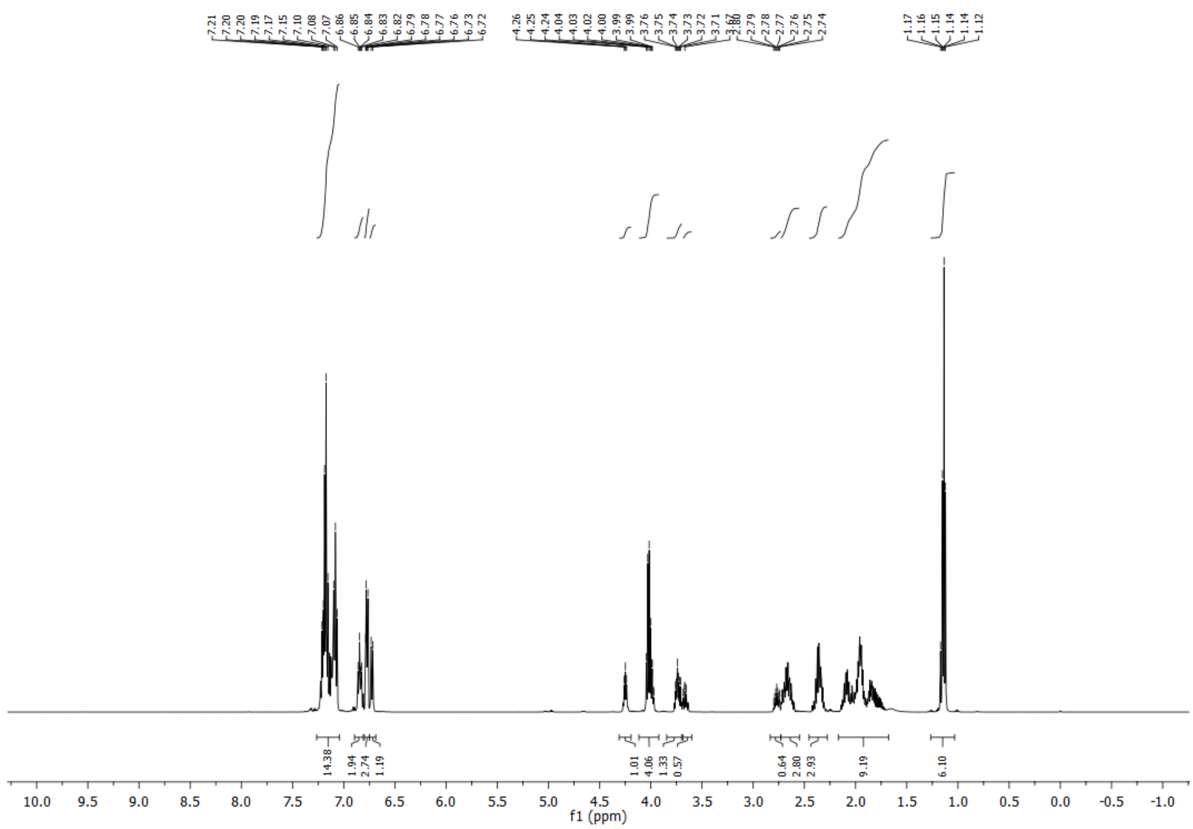

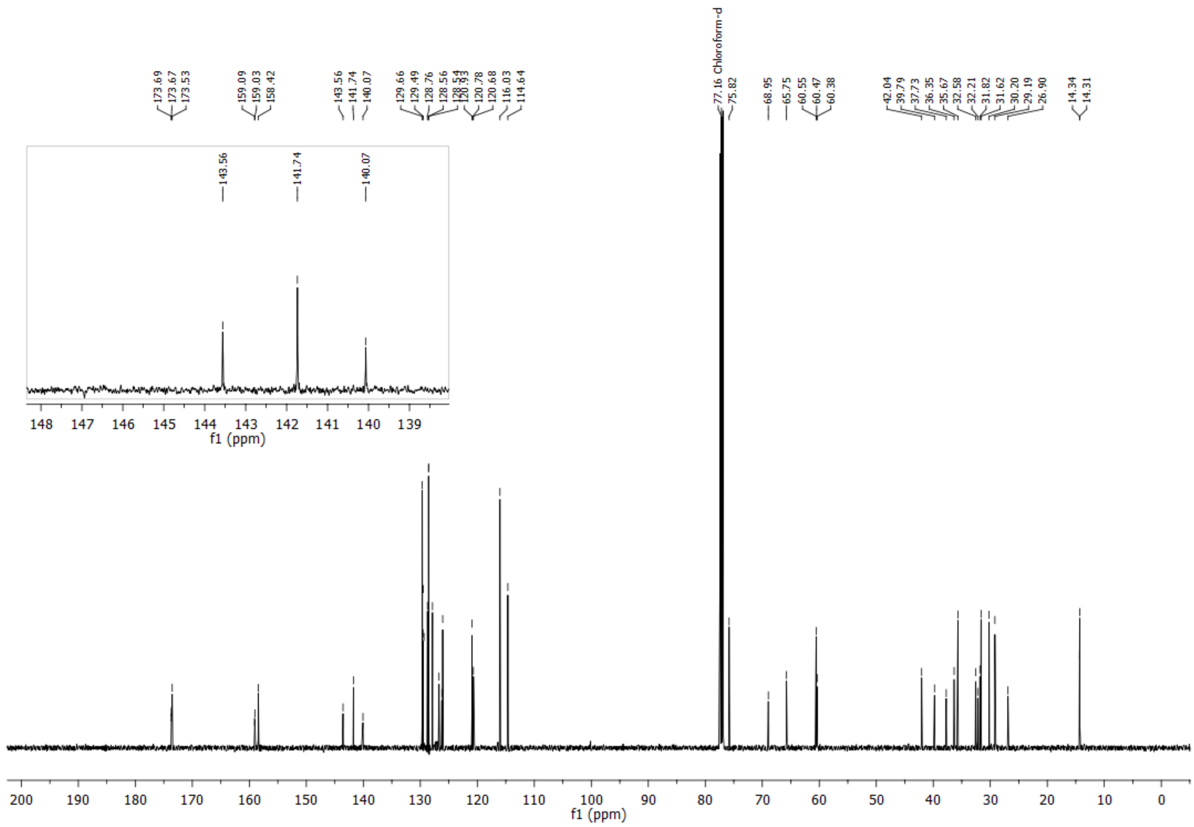

GCMS of material after preparative HPLC

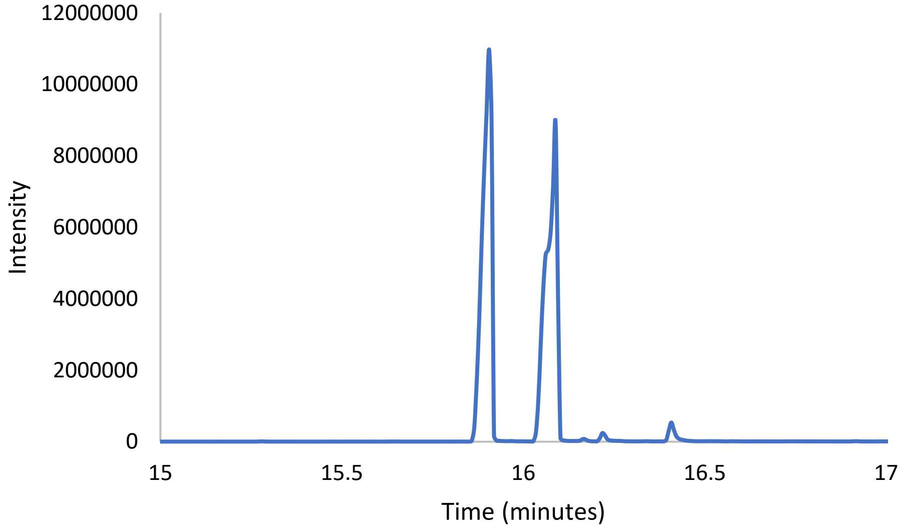

Reaction between dimethyl fumarate and 3-phenoxypropylbenzene with $CuCl_2$ and LiCl

This reaction was performed as previously described above but using dimethyl fumarate (0.0432 g, 0.300 mmol) instead of ethyl acrylate. Purification by flash chromatography eluting with 0→30% diethyl ether in hexane gave a mixture of all three possible regioisomers (0.0617 g). This material was also analysed as previously described and from this, the ratio of regioisomers was determined to be 1.0/1.4/3.2. The NMR spectra of the material from this reaction are shown on page SI.8 and the corresponding spectra of material acquired between dimethyl fumarate and 3-phenoxypropylbenzene and $MesAcr^+$ are shown on page SI.117. Comparison between these two reactions indicate the greater selectivity achieved with this photoredox approach.

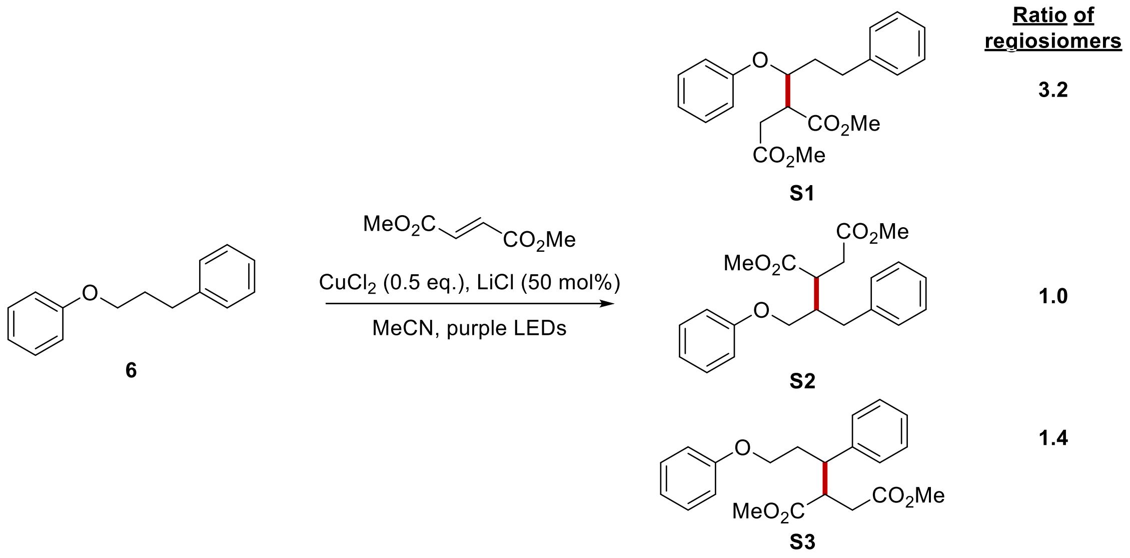

^{1}H-NMR of the reaction mixture after column chromatography

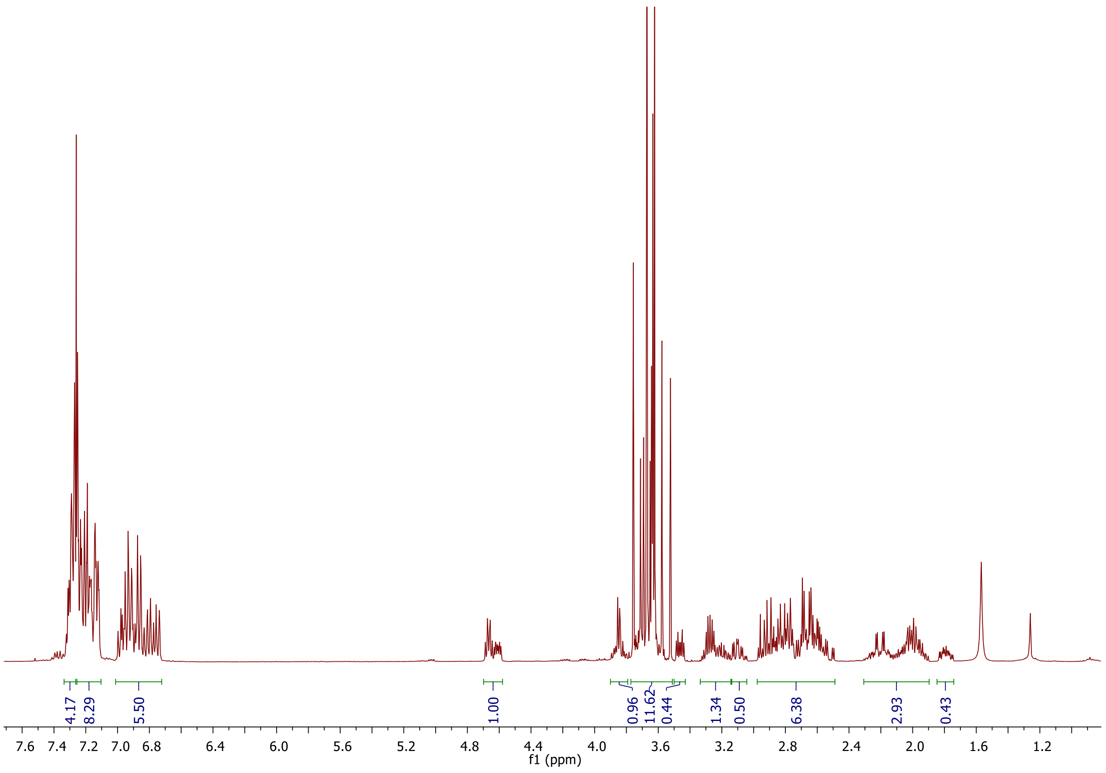

^{13}C-NMR of the reaction mixture after column chromatography

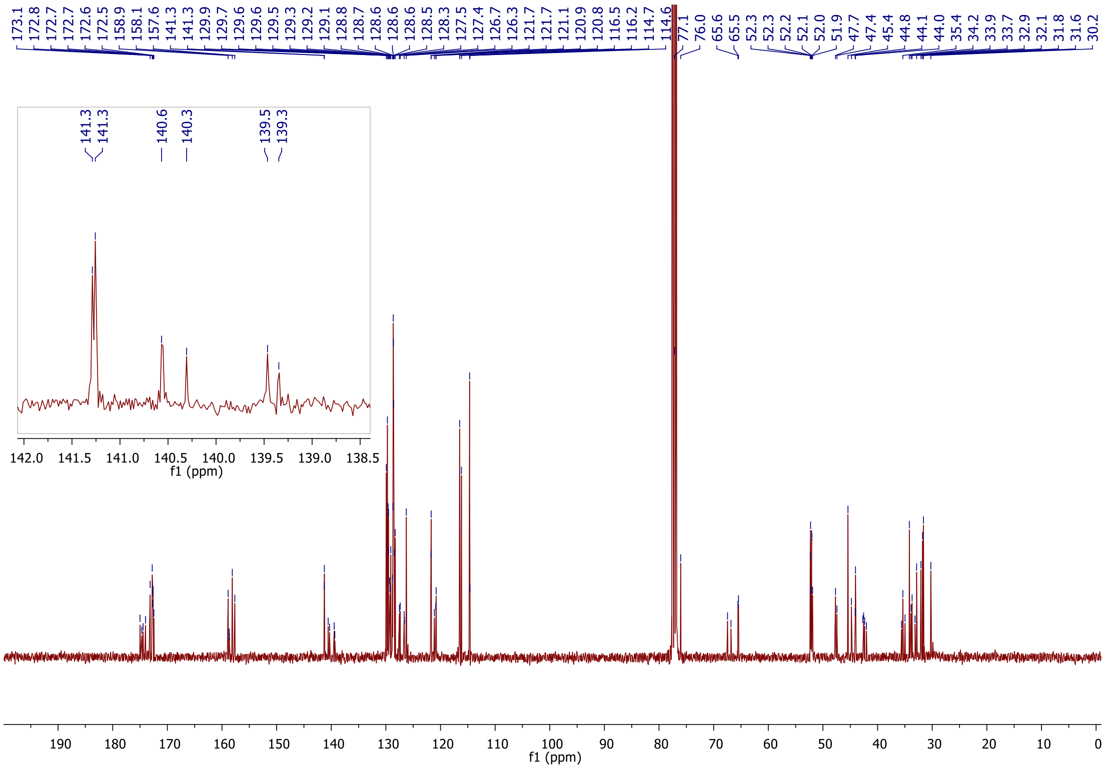

HSQC spectrum of the reaction mixture after column chromatography

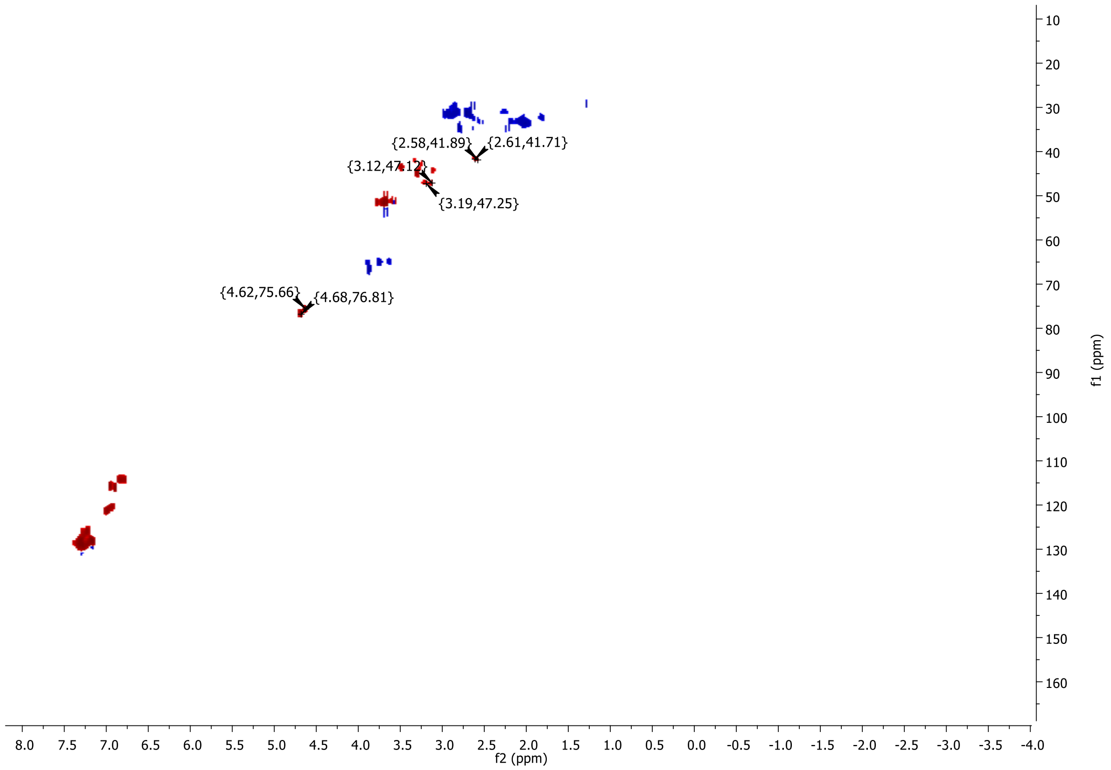
{2.58,41.89}
{2.61,41.71}
{3.12,47.12}
{3.19,47.25}
{4.62,75.66}
{4.68,76.81}
f1 (ppm)
f2 (ppm)

GCMS trace of the reaction mixture after column

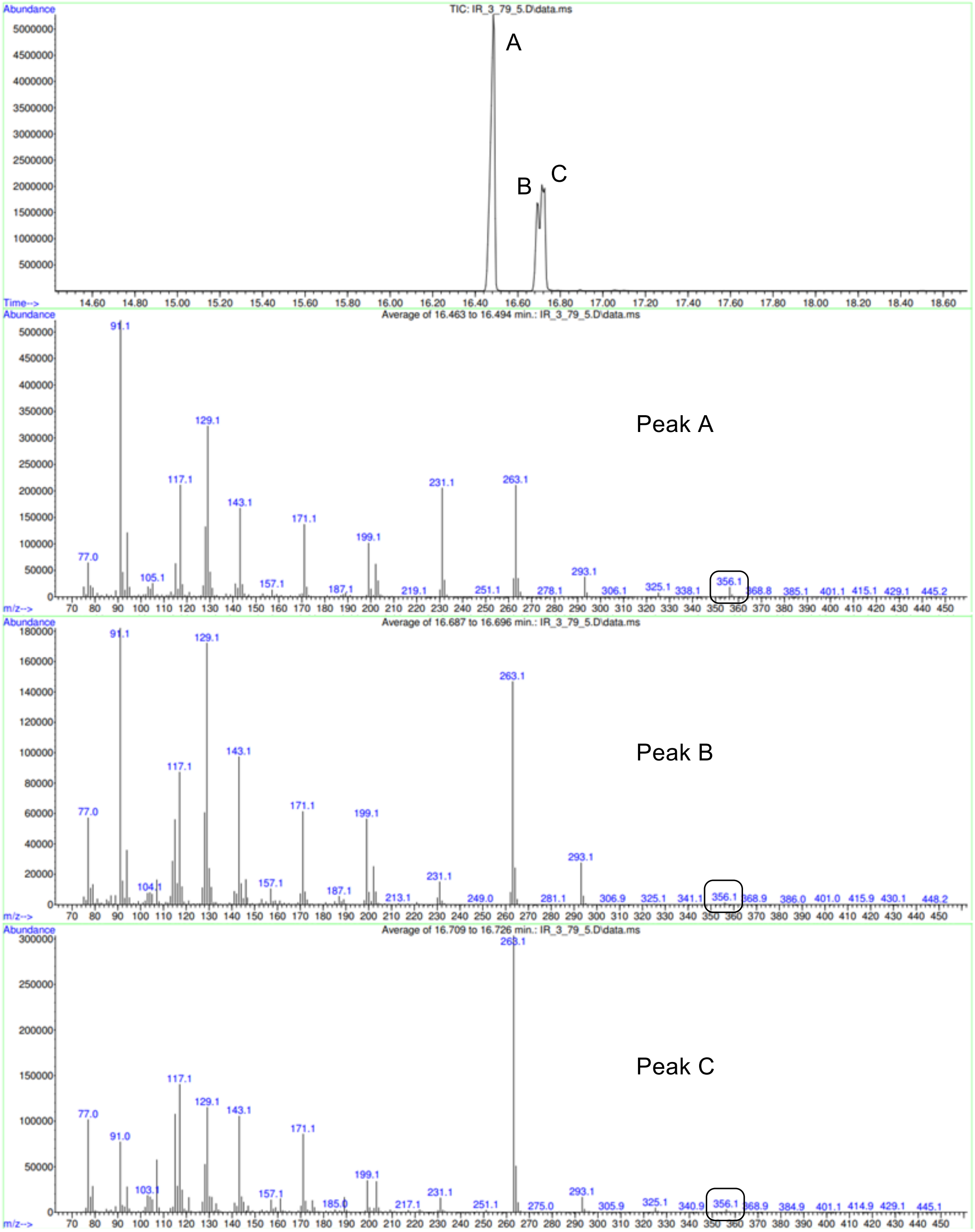

Reaction between benzylidenemalononitrile and 3-phenoxypropylbenzene with $CuCl_2$ and LiCl

This reaction was performed as previously described above using benzylidenemalononitrile (0.0463 g, 0.300 mmol) instead of ethyl acrylate. Purification by flash chromatography eluting with 0→30% diethyl ether in hexane gave a mixture of all three possible regioisomers and 2-benzylmalononitrile (**S7**) (0.0339 g). 2-Benzylmalononitrile was observed in the NMR spectrum of the material after column chromatography [^{1}H-NMR (400 MHz, $CDCl_3$) 3.90 (1H, t, J = 7.0 Hz, 2-H), 3.29 (2H, d, J = 7.0 Hz, 1′-H_2); ^{13}C-NMR spectra (101 MHz, $CDCl_3$) 25.1 (CH), 36.9 (CH_2), 112.3 (2 × CN), 129.0 (CH), 129.3 (2 × CH), 129.5 (2 × CH), 133.1 (C)] as well as by GCMS. This material was also analysed as previously described and from this the ratio of regioisomers was determined to be 1.0/1.6/9.2.

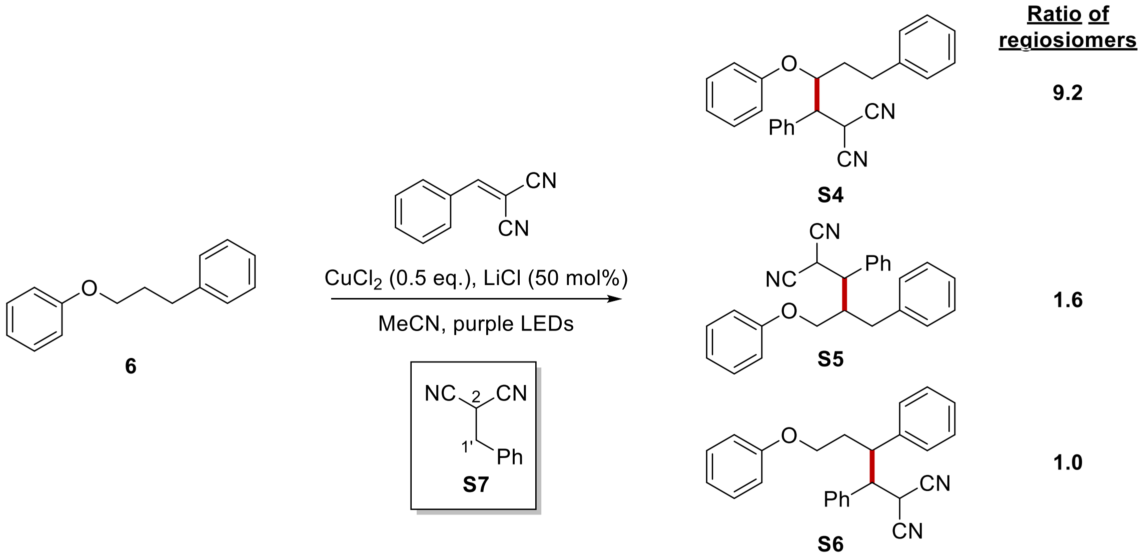

^{1}H-NMR of the reaction mixture after column chromatography

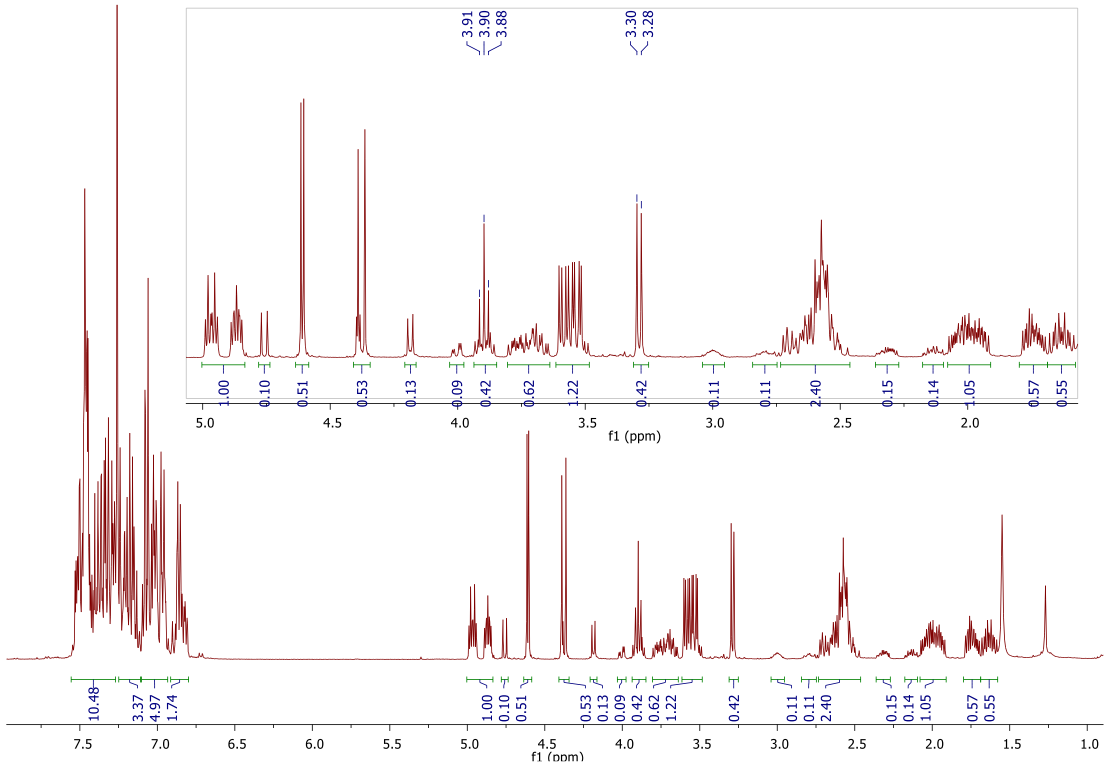

*The peaks labelled in the insert above correspond to 2-benzylmalononitirle

^{13}C-NMR of the reaction mixture after column chromatography

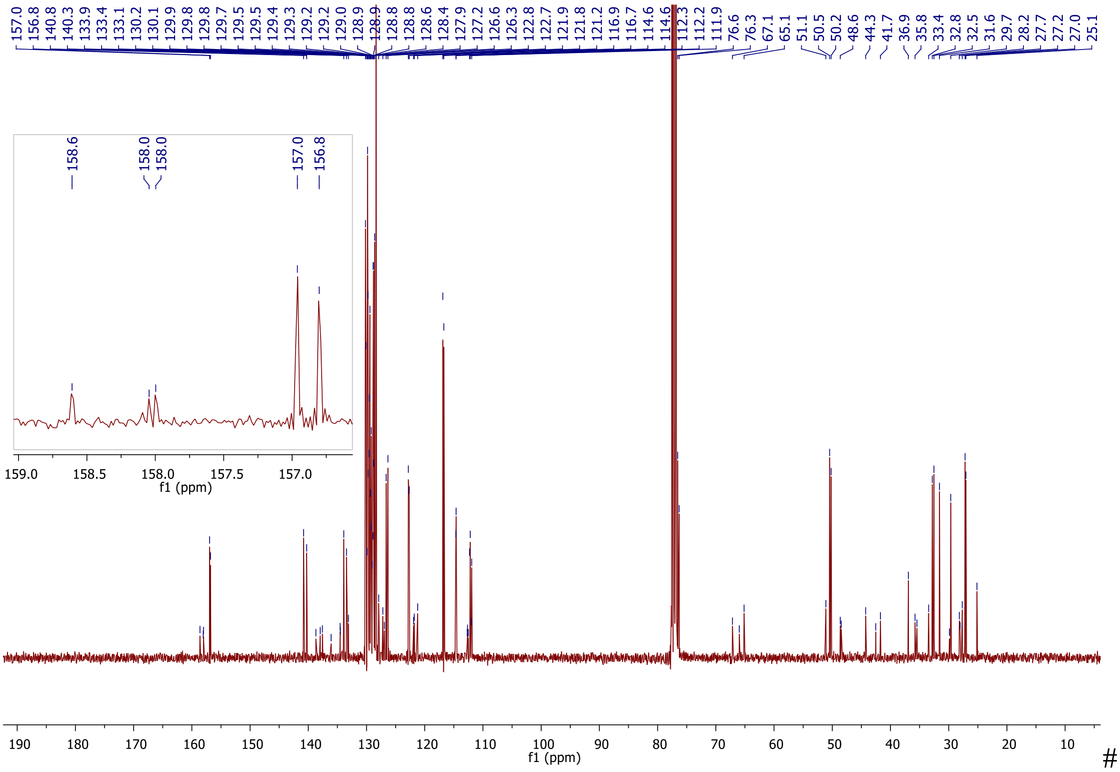

#

HSQC spectrum of the reaction mixture after column chromatography

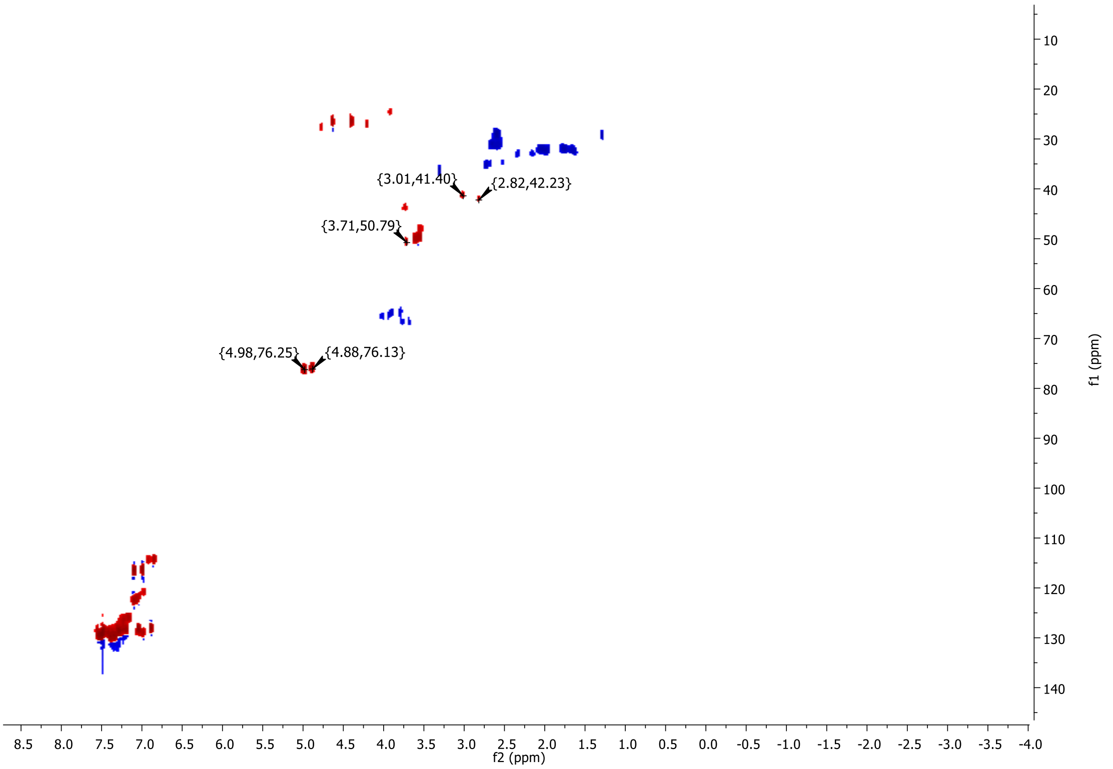

GCMS trace of the reaction mixture after column

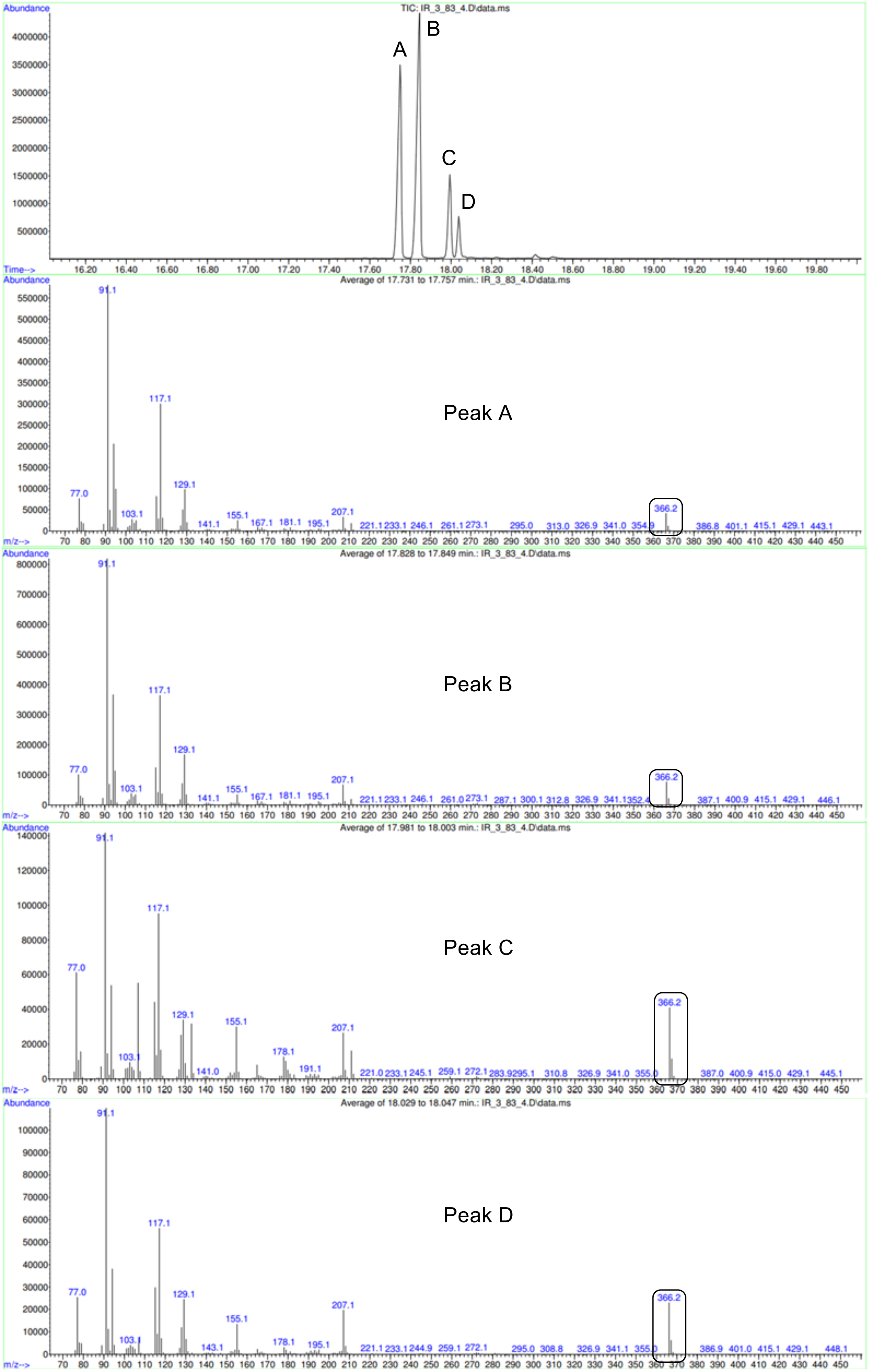

3. Optimisation tables and development of the reaction with MesAcr$^+$

The reaction between dimethyl fumarate and anisole was chosen as a model reaction to find optimal conditions for this transformation. It was found that the reaction was highly efficient in 1,2-dichloroethane but only limited conversions were found in other solvents that had similar polarity (THF, Dimethyl carbonate, and toluene).

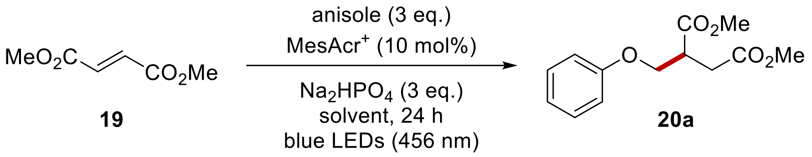

Solvent	NMR Yield of **20a**
THF	0%
Dimethyl carbonate	5%
Toluene	0.5%
1,2-dichloroethane	81%, isolated yield

A number of different bases were found to be compatible with this reaction. The heterogenous base Na_2HPO_4 gave the product in an 81% yield. The organic and homogeneous bases pyridine and 2,6-lutidine were also found to be suitable. As disodium hydrogen phosphate is less toxic and easier to handle it was chosen as base.

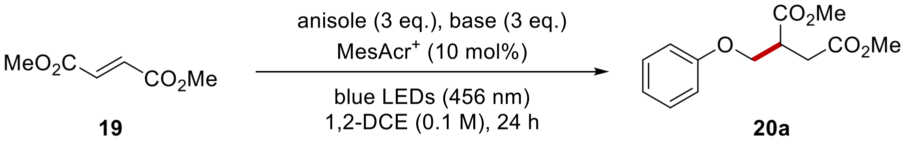

Base	Isolated Yield of **20a**
Na_2HPO_4	81%
Pyridine	80%
2,6-Lutidine	82%

Intrigued that all the bases performed well in our optimisation study it was wondered if the reaction would propagate if there was no base present. When the reaction was performed for 24 h with no base present a comparable yield was achieved. However, on shorter reaction times it was found that base was essential to achieve high conversions and good yields of **20a**.

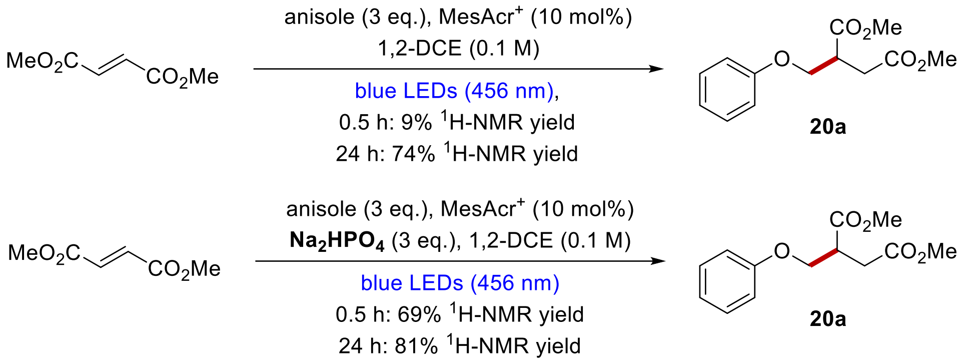

4. Experimental Procedures and Spectroscopic Data for all Compounds

Catalyst synthesis

MesAcr+ **10** was prepared following a literature synthesis, all steps were carried out as in the previously reported synthesis.[2]

Methyl 2,4,6-trimethylbenzoate (S8)[2]

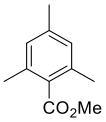

To a heterogeneous mixture of mesitylenecarboxylic acid (6.57 g, 40.0 mmol), potassium carbonate (8.29 g, 60.0 mmol) and *N*,*N*-dimethylformamide (50 mL) was added iodomethane (3.00 mL, 48.0 mmol) dropwise. The resulting reaction mixture was stirred at room temperature for 20 h. The reaction mixture was poured onto water (200 mL) and the organic material was extracted with diethyl ether (6 × 50 mL). The organic fractions were combined, washed with water (100 mL), brine (100 mL) and dried over $MgSO_4$. The dried organic fraction was concentrated *in vacuo*, and this gave methyl 2,4,6-trimethylbenzoate (**S8**) as a colourless oil (7.02 g, 99%). ^{1}H-NMR (400 MHz, $CDCl_3$) 2.29 (3H, s, CH_3), 2.30 (6H, s, 2 × CH_3), 3.90 (3H, s, CH_3), 6.86 (2H, s, 2 × CH); ^{13}C-NMR (101 MHz, $CDCl_3$), 19.8 (2 × CH_3), 21.2 (CH_3), 51.8 (CH_3), 128.5 (2 × CH), 131.0 (C), 135.3 (2 × C), 139.4 (C), 170.7 (C); *m/z* (CI) 178 (M^+, 90), 163 (60), 147 (100), 91 (100), 77 (80).

3,3′-Oxybis(*tert*-butylbenzene) (S9)[2]

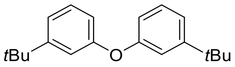

A mixture of 3-*tert*-butylphenol (10.6 g, 70.4 mmol), 1-bromo-3-*tert*-butylbenzene (10.0 g, 46.9 mmol), 2,2,6,6-tetramethyl-3,5-heptanedione (0.979 mL, 4.69 mmol), copper(I) iodide (0.894 g, 4.69 mmol), caesium carbonate (30.6 g, 93.8 mmol) and dimethylformamide (9 mL) was heated at 110 °C for 24 h. The reaction mixture was concentrated *in vacuo* with toluene being added to remove any residual dimethylformamide. The resulting residue was dissolved in diethyl ether (200 mL) and the heterogenous impurities were removed with filtration through celite. The filtrate was washed with water (2 × 100 mL), brine (100 mL), dried over $MgSO_4$ and concentrated *in vacuo*. Purification by flash chromatography, eluting with 2% ethyl acetate in hexane, gave 3,3′-oxybis(*tert*-butylbenzene) (**S9**) as a colourless oil (11.6 g, 87%). ^{1}H-NMR (400 MHz, $CDCl_3$) 1.33 (18H, s, 6 × CH_3), 6.81 (2H, ddd, J = 8.0, 2.3, 0.9 Hz, 6-H and 6′-H), 7.12 (2H, t, J = 2.3 Hz, 2-H and 2′-H), 7.13–7.14 (2H, m, 4-H and 4′-H), 7.27 (2H, t, J = 8.0 Hz, 5-H, 5′-H); ^{13}C-NMR (101 MHz, $CDCl_3$) 31.4 (6 × CH_3), 34.9 (2 × C), 115.6

(2 × CH), 116.4 (2 × CH), 120.2 (2 × CH), 129.3 (2 × CH), 153.5 (2 × C), 157.2 (2 × C); *m/z* (CI) 282 (M^+. 60), 267 (100), 211 (50), 183 (55), 126 (90), 98 (95), 57 (100).

3,6-Di-*tert*-butyl-9-mesitylxanthylium tetrafluoroborate (S10)[2]

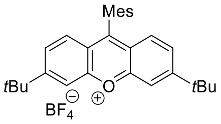

To an anhydrous solution of 3,3′-oxybis(*tert*-butylbenzene) (**S9**) (5.70 g, 20.2 mmol), tetramethylethylenediamine (6.21 mL, 41.4 mmol) in hexane (20 mL) at −78 °C was added *sec*-butyllithium (1.6 M in hexane, 37.6 mL) dropwise. The resulting reaction mixture was warmed to room temperature, stirred for 4 h at room temperature, cooled back to −78 °C and a solution of methyl 2,4,6-trimethylbenzoate (**S8**) (3.63 g, 20.4 mmol) in dry hexane (20 mL) was added dropwise. The resulting reaction mixture was warmed to room temperature and stirred for 16 h. Water (25 mL) was added to the reaction mixture and the biphasic mixture was stirred for 0.5 h. Diethyl ether (3 × 40 mL) was used to extract the organics from the biphasic mixture, the organic fractions were combined and washed with water (2 × 100 mL) and brine (100 mL). Hydrochloric acid (37%, 9 mL) was added to the diethyl ether solution, this mixture was vigorously stirred for 0.5 h and then diluted with water (100 mL). The layers were separated, and the organic layer was further extracted with water until the aqueous fractions became colourless. Sodium tetrafluoroborate (6.65 g, 60.6 mmol) was added to the combined aqueous fractions, and this resulted in the formation of a bright yellow solid and this was extracted from the aqueous solution with dichloromethane (3 × 100 mL). Tetrafluoroboric acid-diethyl ether complex (2.46 mL, 20.2 mmol) was added to the combined organic fractions, and this mixture was swirled to obtain homogeneity. The dichloromethane solution was washed with water (100 mL) and aq. $NaBF_4$ solution (1 M, 100 mL), dried over $NaBF_4$ and concentrated *in vacuo*. The resulting residue was purified by trituration with hexanes, giving a solid that was filtered, and the residue was washed with *n*-pentane. The removal of residual solvent *in vacuo* gave 3,6-di-*tert*-butyl-9-mesitylxanthylium tetrafluoroborate (**S10**) as a yellow-orange solid (6.43 g, 61%). ^{1}H-NMR (400 MHz, $CDCl_3$) 1.51 (18H, s, 6 × CH_3), 1.83 (6H, s, 2 × CH_3), 2.46 (3H, s, CH_3), 7.14 (2H, s, 2 × CH), 7.74 (2H, d, *J* = 8.9 Hz, 1-H and 8-H), 7.91 (2H, dd, *J* = 8.9, 1.7 Hz, 2-H, 7-H), 8.43 (2H, d, *J* = 1.7 Hz, 4-H and 5-H); ^{13}C-NMR (101 MHz, $CDCl_3$) 20.2 (2 × CH_3), 21.4 (CH_3), 30.5 (6 × CH_3), 37.7 (C), 116.7 (2 × CH), 122.1 (2 × C), 127.5 (C), 128.7 (2 × CH), 129.3 (2 × CH), 129.4 (2 × CH), 135.4 (2 × C), 141.5 (C), 158.5 (2 × C), 171.3 (2 × C), 174.6 (C); *m/z* (CI) 411 (M^+, 100), 396 (80), 355 (50), 281 (50), 207 (60), 73 (70).

3,6-Di-*tert*-butyl-9-mesityl-10-phenylacridin-10-ium tetrafluoroborate (10)[2]

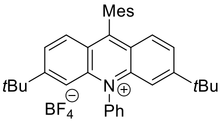

To an anhydrous solution of 3,6-di-*tert*-butyl-9-mesitylxanthylium tetrafluoroborate (**S10**) (2.00 g, 4.01 mmol) in dichloromethane (8 mL) was added acetic acid (0.688 mL, 12.0 mmol), triethylamine (0.838 mL, 6.02 mmol) and aniline (0.440 mL, 4.81 mmol). The reaction mixture was covered with aluminium foil to exclude light, sparged with argon gas for 10 min, and stirred for 18 h at room temperature. Dichloromethane (20 mL) was used to dilute the reaction mixture before it was poured onto water (50 mL) and separated. The aqueous layer was further extracted with dichloromethane (2 × 30 mL). Once all the organic fractions were combined, they were washed with water (50 mL) and sat. aq. $NaHCO_3$ solution (50 mL). Tetrafluoroboric acid-diethyl ether complex (0.550 mL, 4.01 mmol) was added to the dichloromethane solution. The acidified organic layer was further washed with aq. $NaBF_4$ solution (1 M, 50 mL), dried over $NaBF_4$ and concentrated *in vacuo*. The resulting residue was purified by triturated with a mixture of diethyl ether and hexane (1:2 ratio) and this gave 3,6-di-*tert*-butyl-9-mesityl-10-phenylacridin-10-ium tetrafluoroborate (**10**) as a yellow solid (2.01 g, 88%). ^{1}H-NMR (400 MHz, $CDCl_3$) 1.29 (18H, s, 6 × CH_3), 1.86 (6H, s, 2 × CH_3), 2.49 (3H, s, CH_3), 7.16 (2H, s, 2 × CH), 7.42 (2H, s, 2 × CH), 7.69 (2H, d, J = 7.2 Hz, 2 × CH), 7.76–7.82 (4H, m, 4 × CH), 7.86–8.02 (3H, m, 3 × CH); ^{13}C-NMR (101 MHz, $CDCl_3$) 19.7 (2 × CH_3), 20.8 (CH_3), 29.7 (6 × CH_3), 36.2 (C), 114.6 (2 × CH), 123.6 (2 × C), 126.9 (2 × CH), 127.6 (2 × CH), 127.8 (2 × CH), 128.4 (2 × CH), 128.8 (CH), 131.1 (2 × CH), 131.3 (C), 135.7 (2 × C), 136.4 (C), 139.7 (C), 141.7 (2 × C), 161.8 (C), 163.0 (2 × C); *m/z* (ESI) 486 $[M]^+$.

Alkene synthesis

2-Methylene-1,3-diphenylpropane-1,3-dione (S11)[3]

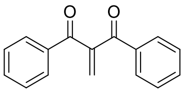

This compound was prepared following a previously reported synthesis.[3] A solution of 1,3-diphenylpropanedione (0.500 g, 2.23 mmol), iron(III) trichloride (0.0600 g, 0.223 mmol), potassium persulfate (1.21 g, 4.46 mmol) and dimethylacetamide (9 mL) was heated at 110 °C for 4 h. The reaction mixture was cooled and diluted with diethyl ether (50 mL), washed with lithium chloride solution (10% in water), water (50 mL) and brine (50 mL). The washed organic layer was dried over $MgSO_4$, concentrated *in vacuo* and this gave the crude product. Purification by flash chromatography eluting with 30% diethyl ether in hexane gave 2-methylene-1,3-diphenylpropane-1,3-dione (**S11**) as a yellow oil (0.297 g, 56%). ^{1}H-NMR (400 MHz, $CDCl_3$) 6.34 (2H, s, CH_2), 7.41–7.50 (4H, m, 4 × CH), 7.58

(2H, tt, J = 7.4, 1.3 Hz, 2 × CH), 7.85–7.91 (4H, m, 4 × CH); ^{13}C-NMR (101 MHz, $CDCl_3$) 128.8 (4 × CH), 129.7 (2 × CH), 131.4 (CH_2), 133.6 (4 × CH), 136.4 (C), 148.2 (C), 194.3 (C); *m/z* (EI) 236 (M^+, 5), 208 (10), 105 (100), 77 (100).

Tetraethyl ethene-1,1-diylbis(phosphonate) (S12)[4]

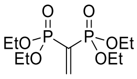

This compound was prepared following a previously reported synthesis.[4] A solution of tetraethyl methylenediphosphonate (1.00 g, 3.47 mmol), paraformaldehyde (0.521 g, 17.4 mmol), diethylamine (0.400 mL, 3.82 mmol) and methanol (10 mL) was heated under reflux for 18 h. The reaction mixture was concentrated *in vacuo*, and this gave the crude product tetraethyl (1-methylenemethoxy)methylene bisphosphonate. Toluene was added and the excess paraformaldehyde and methanol were removed azeotropically *in vacuo* and this was done twice. The residue was dissolved in toluene (10 mL), followed by the addition *p*-TsOH (0.0600 g, 0.350 mmol), and the reaction mixture was heated under reflux for 20 h and then concentrated *in vacuo*, which gave the crude product. Flash chromatography with silica gel, eluting with 5% MeOH in dichloromethane, gave tetraethyl ethene-1,1-diylbis(phosphonate) (**S12**) as a colourless oil (0.820 g, 79%). Chromatography of the product had to be performed quickly as the silica gel enabled addition of methanol to the alkene, but material of sufficient purity was isolated. ^{1}H-NMR (400 MHz, $CDCl_3$) 1.33 (12H, t, J = 7.1 Hz, 4 × CH_3), 4.13 (8H, m 4 × CH_2), 6.97 (2H, ddd, J = 40, 27, 8 Hz, CH_2); ^{13}C-NMR (101 MHz, $CDCl_3$) 16.1–16.6 (m, CH_3), 62.6–63.0 (m, CH_2), 132.2 (t, J = 166.6 Hz, C), 149.0 (CH_2); *m/z* (EI) 300 (M^+, 5), 273 (60), 245 (70), 217 (80), 171 (100).

4-Methylbenzylidenemalononitrile (S13)[5]

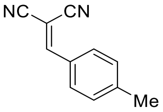

This compound was prepared using an adapted literature synthesis.[6] A solution of 4-methylbenzaldehyde (0.892 mL, 7.57 mmol), malononitrile (0.500 g, 7.57 mmol), piperidine (37 μL, 0.379 mmol) in ethanol (15 mL) was heated under reflux for 3 h. The reaction mixture was cooled to room temperature and filtered. The solid was washed with a minimum amount of ethanol which gave 4-methylbenzylidenemalononitrile (**S13**) as a light brown crystalline solid (0.836 g, 66%). Mp 133–134 °C (lit.[5] 136 °C); ^{1}H-NMR (400 MHz, $CDCl_3$) 2.46 (3H, s, CH_3), 7.34 (2H, d, J = 8.2 Hz, 2 × CH), 7.72 (1H, s, CH), 7.81 (2H, d, J = 8.2 Hz, 2 × CH); ^{13}C-NMR (101 MHz, $CDCl_3$) 22.1 (CH_3), 81.4 (C),

113.0 (C≡N), 114.1 (C≡N), 128.6 (C), 130.5 (2 × CH), 131.1 (2 × CH), 146.5 (C), 159.9 (CH); *m/z* (EI) 168 (M^+, 85), 141 (100), 114 (70), 89 (40).

4-Fluorobenzylidenemalononitrile (S14)[7]

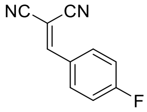

4-Fluorobenzylidenemalononitrile (**S14**) was synthesised as described for **S13** using 4-fluorobenzaldehyde (0.812 mL, 7.57 mmol) which gave 4-fluorobenzylidenemalononitrile (**S7**) as an orange solid (0.296 g, 23%). Mp 124–126 °C (lit.[7] 125–127 °C); ^{1}H-NMR (500 MHz, $CDCl_3$) 7.19–7.30 (2H, m, 2 × CH), 7.74 (1H, s, CH), 7.91–8.02 (2H, m, 2 × CH); ^{13}C-NMR (126 MHz, $CDCl_3$) 82.6 (d, J = 2.8 Hz, C), 112.6 (C≡N), 113.7 (C≡N), 117.4 (d, $^2J_{CF}$ = 22.5 Hz, 2 × CH), 127.5 (d, $^4J_{CF}$ = 3.2 Hz, C), 133.6 (d, $^3J_{CF}$ = 9.4 Hz, 2 × CH), 158.4 (CH), 166.3 (d, $^1J_{CF}$ = 260.2 Hz, C); *m/z* (EI) 172 (M^+, 100), 145 (95), 121 (90).

4-Chlorobenzylidenemalononitrile (S15)[5]

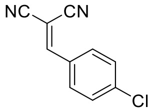

4-Chlorobenzylidenemalononitrile (**S15**) was synthesised as described for **S13** using 4-chlorobenzaldehyde (0.545 g, 7.57 mmol) which gave 4-chlorobenzylidenemalononitrile (**S15**) as a yellow solid (1.04 g, 73%). Mp 160–162 °C (lit.[5] 161 °C); ^{1}H-NMR (400 MHz, $CDCl_3$) 7.52 (1H, d, J = 8.6 Hz, 2 × CH), 7.74 (1H, s, CH), 7.85 (2H, d, J = 8.6 Hz); ^{13}C-NMR (101 MHz, $CDCl_3$) 83.5 (C), 112.5 (C≡N), 113.6 (C≡N), 129.4 (C), 130.2 (2 × CH), 132.0 (2 × CH), 141.3 (C), 158.4 (CH); *m/z* (EI) 190 (M^+, 30), 188 (M^+, 100), 161 (90), 153 (80), 137 (90), 125 (85), 99 (80).

4-Bromobenzylidenemalononitrile (S16)[5]

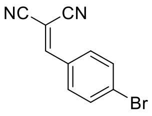

4-Bromobenzylidenemalononitrile (**S16**) was synthesised as described for **S13** using 4-bromobenzaldehyde (1.40 g, 7.57 mmol) which gave 4-bromobenzylidenemalononitrile (**S16**) as a brown solid (1.02 g, 58%). Mp 159–161 °C (lit.[5] 161 °C); ^{1}H-NMR (400 MHz, $CDCl_3$) 7.69 (2H, d, *J* = 8.7 Hz, 2 × CH), 7.72 (1H, s, CH), 7.77 (2H, d, *J* = 8.7 Hz, 2 × CH); ^{13}C-NMR (101 MHz, $CDCl_3$) 83.7 (C), 112.5 (C≡N), 113.6 (C≡N), 129.8 (C), 130.1 (C), 131.9 (2 × CH), 133.2 (2 × CH), 158.5 (CH); *m/z* (EI) 234 (100), 232 (100), 205 (60), 181 (40), 154 (90).

4-Iodobenzylidenemalononitrile (S17)[8]

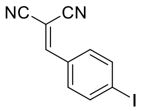

4-Iodobenzylidenemalononitrile (**S17**) was synthesised as described for **S13** using 4-iodobenzaldehyde (1.76 g, 7.57 mmol) which gave 4-iodobenzylidenemalononitrile (**S17**) as a brown solid (2.16 g, 100%). Mp 143–145 °C (lit.[8] 148 °C); ^{1}H-NMR (400 MHz, $CDCl_3$) 7.61 (2H, d, *J* = 8.4 Hz, 2 × CH), 7.70 (1H, s, CH), 7.91 (2H, d, *J* = 8.4 Hz, 2 × CH); ^{13}C-NMR (101 MHz, $CDCl_3$) 83.7 (C), 103.0 (C), 112.5 (C≡N), 113.6 (C≡N), 130.2 (C), 131.7 (2 × CH), 139.2 (2 × CH), 158.9 (CH); *m/z* (EI) 280 (M^+, 100), 153 (75), 126 (75), 100 (60), 87 (30), 74 (50), 63 (40).

4-Methoxybenzylidenemalononitrile (S18)[5]

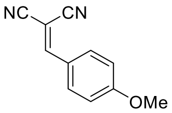

4-Methoxybenzylidenemalononitrile (**S18**) was synthesised as described for **S13** using 4-methoxybenzaldehyde (1.03 g, 7.57 mmol) which gave 4-methoxybenzylidenemalononitrile (**S18**) as a yellow solid (1.09 g, 78%). Mp 111–114 °C (lit.[5] 116 °C); ^{1}H-NMR (400 MHz, $CDCl_3$) 3.92 (3H, s, OMe), 7.01 (2H, d, *J* = 9.0 Hz, 2 × CH), 7.65 (1H, s, CH), 7.91 (2H, d, *J* = 9.0 Hz, 2 × CH); ^{13}C-NMR (101 MHz, $CDCl_3$) 55.3 (CH_3), 78.2 (C), 112.8 (C≡N), 113.9 (C≡N), 114.6 (2 × CH), 123.5 (C), 133.0 (2 × CH), 158.3 (CH), 164.3 (C); *m/z* (EI) 168 (M^+, 80), 141 (100), 114 (60), 89 (40).

4-Nitrobenzylidenemalononitrile (S19)[5]

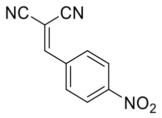

4-Nitrobenzylidenemalononitrile (**S19**) was synthesised as described for **S13** using 4-nitrobenzaldehyde (1.14 g, 7.57 mmol) which gave 4-nitrobenzylidenemalononitrile (**S19**) as a brown solid (0.981 g, 65%). Mp 162–164 °C (lit.[5] 161 °C); ^{1}H-NMR (400 MHz, $CDCl_3$) 7.89 (1H, s, CH), 8.07 (2H, d, *J* = 8.8 Hz, 2 × CH), 8.38 (2H, d, *J* = 8.8 Hz, 2 × CH); ^{13}C-NMR (101 MHz, $CDCl_3$) 87.6 (C), 111.7 (C≡N), 112.8 (C≡N), 124.8 (2 × CH), 131.4 (2 × CH), 135.9 (C), 150.5 (C), 157.0 (CH); *m/z* (EI) 199 (M^+, 95), 169 (50), 153 (90), 141 (90), 125 (100).

Ether synthesis

2-(4′-Methoxyphenyl)-2-methyI-1,3-dioxolane (S20)[9]

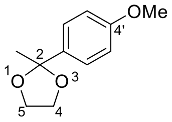

4-Methoxyacetophenone (5.53 g, 36.7 mmol), ethylene glycol (5.00 g, 80.6 mmol) and *para*-toluenesulfonic acid (0.050 g, 0.290 mmol) were dissolved in toluene (50 mL) and this reaction mixture was heated under reflux, using Dean-Stark apparatus for 24 h. The reaction mixture was washed with 2 M NaOH (50 mL), water (50 mL) and brine (50 mL) before it was dried over $MgSO_4$. Concentration of the organic fraction *in vacuo* gave a residue that was purified by flash chromatography eluting with 20% diethyl ether in dichloromethane and this afforded 2-(4′-methoxyphenyl)-2-methyl-1,3-dioxolane (**S20**) as a colourless oil (4.42 g, 62%). Spectroscopic data were in agreement with literature data.[9] ^{1}H-NMR (400 MHz, $CDCl_3$) 1.65 (3H, s, 2-CH_3), 3.75–3.82 (5H, m, OCH_3, 4-*H*H and 5-*H*H), 4.02 (2H, td, *J* = 6.1, 4.2 Hz, 4-H*H* and 5-H*H*), 6.83–6.90 (2H, m, 2 × CH), 7.35–7.44 (2H, m, 2 × CH); ^{13}C-NMR (101 MHz, $CDCl_3$) 27.8 (CH_3), 55.4 (CH_3), 64.5 (2 × CH_2), 108.9 (C), 113.6 (2 × CH), 126.7 (2 × CH), 130.8 (C), 159.4 (C); *m/z* (EI) 194 (M^+, 5), 179 (100), 163 (10), 135 (80), 119 (10), 92 (30), 77 (25).

Hexyl phenyl ether (S21)[10]

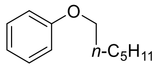

A solution of phenol (1.04 g, 11.0 mmol), potassium carbonate (1.79 g, 13.0 mmol), 1-bromohexane (0.845 mL, 6.00 mmol) and acetone (20 mL) was heated under reflux for 16 h. The reaction mixture was concentrated *in vacuo* and the resulting residue was dissolved in diethyl ether (50 mL). The organic layer was washed with 2 M aqueous NaOH (2 × 50 mL), brine (50 mL), passed through a hydrophobic frit, and concentrated *in vacuo* which gave hexyl phenyl ether (**S21**) as a colourless oil (0.911 g, 85%). Spectroscopic data were in agreement with literature data.[10] ^{1}H-NMR (400 MHz, $CDCl_3$) 0.86–0.96 (3H, m, CH_3), 1.29–1.52 (6H, m, 3 × CH_2), 1.73–1.83 (2H, m, CH_2), 3.95 (2H, t, *J* = 6.6 Hz, CH_2), 6.86–6.96 (3H, m, 3 × CH), 7.24–7.30 (2H, m, 2 × CH); ^{13}C-NMR (101 MHz, $CDCl_3$) 13.5 (CH_3), 22.1 (CH_2), 25.2 (CH_2), 28.8 (CH_2), 31.1 (CH_2), 67.4 (CH_2), 114.0 (2 × CH), 120.0 (CH), 128.9 (2 × CH), 158.6 (C); *m/z* (ESI): 179 $[M+H]^+$.

3-(1′,3′-Dioxoisoindolin-2′-yl)propyl phenyl ether (S22)[11]

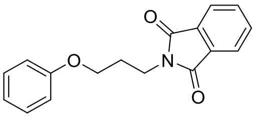

A heterogenous mixture of phenol (0.565 g, 6.00 mmol), potassium carbonate (1.40 g, 10.0 mmol), *N*-(3-bromopropyl)phthalimide (1.34 g, 5.00 mmol) and *N*,*N*-dimethylformamide (10 mL) was stirred at room temperature for 17 h. The reaction mixture was partitioned between diethyl ether (10 ml) and water (10 ml). The organic layer was separated, and the aqueous layer was further extracted with diethyl ether (2 x 10 ml). The organic fractions were combined, washed with 2 M aqueous NaOH (2 × 20 mL), brine (30 mL) passed through a hydrophobic frit, concentrated *in vacuo* which gave 3-(1′,3′-dioxoisoindolin-2′-yl)propyl phenyl ether (**S22**) as a white solid (1.19 g, 85%). Spectroscopic data were in agreement with the literature data.[11] ^{1}H NMR (400 MHz, $CDCl_3$) 2.21 (2H, quintet, *J* = 6.9 Hz, CH_2), 3.94 (2H, t, *J* = 6.9 Hz, CH_2), 4.06 (2H, t, *J* = 6.1 Hz, CH_2), 6.81–6.87 (2H, m, 2 × CH), 6.91–6.98 (1H, m, CH), 7.23–7.28 (2H, m, 2 × CH), 7.71–7.76 (2H, m, 2 × CH), 7.84–7.90 (2H, m, 2 × CH); ^{13}C NMR (101 MHz, $CDCl_3$) 27.8 (CH_2), 35.0 (CH_2), 65.0 (CH_2), 114.0 (2 × CH), 120.3 (CH), 122.7 (2 × CH), 128.9 (2 × CH), 131.7 (2 × C) 133.4 (2 × CH), 158.2 (C), 167.9 (2 × C); *m/z* (ESI): 282 $[M+H]^+$.

3-Phenoxypropyl acetate (S23)[12]

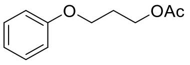

To a solution of 3-phenoxypropanol (0.500 g, 3.28 mmol) in dichloromethane (10 mL) was added triethylamine (0.914 mL, 6.56 mmol) and acetic anhydride (0.503 g, 4.92 mmol) at 0 °C. The reaction mixture was warmed to room temperature and stirred for 16 h. The reaction mixture was diluted with dichloromethane (40 mL) and water (50 mL). The organic fraction was washed with brine (50 mL), dried over $MgSO_4$, and concentrated *in vacuo*. Purification by flash chromatography eluting with 50% diethyl ether in hexane gave 3-phenoxypropyl acetate (**S23**) as a colourless oil (0.649 g, 100%). Spectroscopic data were in agreement with the literature data.[12] ^{1}H-NMR (400 MHz, $CDCl_3$) 2.08 (3H, s, CH_3), 2.14 (2H, quintet, J = 6.3 Hz, CH_2), 4.07 (2H, t, J = 6.3 Hz, CH_2), 4.30 (2H, t, J = 6.3 Hz, CH_2), 6.90–7.01 (3H, m, 3 × CH), 7.27–7.35 (2H, m, 2 × CH); ^{13}C-NMR (101 MHz, $CDCl_3$) 21.0 (CH_3), 28.7 (CH_2), 61.4 (CH_2), 64.2 (CH_2), 114.5 (2 × CH), 120.9 (CH), 129.5 (2 × CH), 158.8 (C), 171.1 (C); *m/z* (EI) 194 (M^+, 80), 133 (30), 101 (100), 94 (60), 77 (80), 65 (100).

3-Phenoxypropyl benzoate (S24)[13]

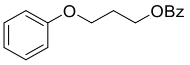

To a solution of 3-phenoxypropanol (0.308 g, 2.02 mmol), benzoic acid (0.247 g, 2.02 mmol), 4-dimethylaminopyridine (0.0250 g, 0.202 mmol) in dichloromethane (10 mL) was added *N*,*N*'-dicyclohexylcarbodiimide (0.500 g, 2.42 mmol) at 0 °C. The reaction was warmed to room temperature and stirred for 20 h. The heterogenous reaction mixture was filtered through celite and concentrated *in vacuo* and the resulting residue was dissolved in dichloromethane. Upon the addition of a further portion of dichloromethane, further urea precipitated and the mixture was filtered. Purification by flash chromatography eluting with 50% diethyl ether in hexane gave 3-phenoxypropyl benzoate (**S24**) as a colourless oil (0.437 g, 84%). Spectroscopic data in agreement with the literature data.[13] ^{1}H-NMR (400 MHz, $CDCl_3$) 2.28 (2H, p, J = 6.2 Hz, CH_2), 4.16 (2H, t, J = 6.2 Hz, CH_2), 4.56 (2H, t, J = 6.2 Hz, CH_2), 6.90–7.02 (3H, m, 3 × CH), 7.31 (2H, dd, J = 8.5, 7.5 Hz, 2 × CH), 7.46 (2H, t, J = 7.6 Hz, 2 × CH), 7.57 (1H, tt, J = 7.4, 1.2 Hz, CH), 8.07 (2H, dd, J = 8.2, 1.2 Hz, CH); ^{13}C-NMR (101 MHz, $CDCl_3$) 28.9 (CH_2), 62.0 (CH_2), 64.4 (CH_2), 114.6 (2 × CH), 120.9 (CH), 128.5 (2 × CH), 129.6 (2 × CH), 129.7 (2 × CH), 130.3 (C), 133.0 (CH), 158.9 (C), 166.6 (C); *m/z* (EI) 256 (M^+, 40), 164 (100), 133 (45), 105 (70), 94 (100), 78 (80), 65 (95).

Isopropyl phenyl ether (S25)[14]

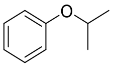

A solution of phenol (0.565 g, 6.00 mmol), sodium hydroxide (0.360 g, 9.00 mmol), 2-bromopropane (0.875 mL) in *N*,*N*-dimethylformamide (5 mL) was heated at 70 °C for 17 h. The reaction mixture was cooled to room temperature and partitioned between diethyl ether (10 mL) and water (10 mL). The organic layer was separated, and the aqueous layer was further extracted with diethyl ether (2 x 10 mL). The organic fractions were combined, washed with brine (3 x 20 ml), passed through a hydrophobic frit, concentrated *in vacuo* which gave isopropyl phenyl ether (**S25**) as a pale orange oil (0.715 g, 88%). Spectroscopic data were in agreement with the literature data.[14] ^{1}H-NMR (400 MHz, $CDCl_3$) 1.34 (6H, d, *J* = 6.1 Hz, 2 × CH_3), 4.55 (1H, sept , *J* = 6.1 Hz, CH), 6.86–6.95 (3H, m, 3 × CH), 7.22–7.31 (2H, m, 2 × CH); ^{13}C-NMR (101 MHz, $CDCl_3$) 21.6 (2 × CH_3), 69.3 (CH), 115.4 (2 × CH), 120.0 (CH), 128.9 (2 × CH), 157.4 (C); *m/z* (ESI): 137 $[M+H]^+$.

Cyclobutyl phenyl ether (S26)[15]

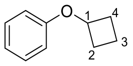

A mixture of phenol (0.941 g, 10.0 mmol), cyclobutyl bromide (1.89 mL, 20.0 mmol), potassium carbonate (2.76 g, 20.0 mmol) and dimethyl sulfoxide (20 mL) was heated at 50 °C for 20 h. The reaction mixture was poured onto water (50 mL), extracted with diethyl ether (2 × 30 mL), the organics were combined, washed with water (50 mL), brine (50 mL), dried over $MgSO_4$ and concentrated *in vacuo*. The resulting residue was purified by flash chromatography eluting with 5% dichloromethane in hexane which gave cyclobutyl phenyl ether (**S26**) as a colourless oil (1.29 g, 87%). Spectroscopic data were in agreement with the literature data.[15] ^{1}H-NMR (400 MHz, $CDCl_3$) 1.62–1.77 (1H, m, 3-*H*H), 1.80–1.95 (1H, m, 3-H*H*), 2.11–2.26 (2H, m, 2-*H*H and 4-*H*H), 2.39–2.53 (2H, m, 2-H*H* and 4-H*H*), 4.57–4.73 (1H, m, 1-H), 6.83 (2H, dd, *J* = 8.7, 1.0 Hz, 2 × CH), 6.94 (1H, tt, *J* = 7.5, 1.0 Hz, CH), 7.27 (2H, dd, *J* = 8.7, 7.4 Hz, 2 × CH); ^{13}C-NMR (101 MHz, $CDCl_3$) 13.4 (CH_2), 30.8 (2 × CH_2), 71.5 (CH), 115.1 (2 × CH), 120.1 (CH), 129.6 (2 × CH), 157.7 (C); *m/z* (EI) 148.2 (M^+, 40), 120 (100), 94 (60), 71 (40), 65 (30), 55 (50).

Cyclopentyl phenyl ether (S27)[16]

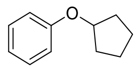

Cyclopentyl phenyl ether (**S27**) was synthesised as described for **S26** using phenol (0.941 g, 10.0 mmol), bromocyclopentane (2.14 mL, 20.00 mmol), potassium carbonate (2.76 g, 20.0 mmol) and DMSO (20 mL). Purification by flash chromatography eluting with 5% diethyl ether in hexane gave cyclopentyl phenyl ether (**S27**) as a colourless oil (1.18 g, 73%). Spectroscopic data were in agreement with the literature data.[16] ^{1}H-NMR (400 MHz, $CDCl_3$) 1.57–2.00 (8H, m, 4 × CH_2), 4.78 (1H, tt, *J* = 6.2, 3.0 Hz, CH), 6.86–6.98 (3H, m, 3 × CH), 7.23–7.33 (2H, m, 2 × CH); ^{13}C-NMR (101 MHz, $CDCl_3$) 24.2 (2 × CH_2), 33.0 (2 × CH_2), 79.2 (CH), 115.7 (2 × CH), 120.3 (CH), 129.5 (2 × CH), 158.3 (C); *m/z* (EI) 162 (M^+, 30), 94 (100), 77 (40), 65 (50), 51 (30).

3-Phenoxypropylbenzene (6)[17]

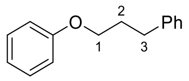

4-Phenoxypropylbenzene (**6**) was synthesised as described for **S21** using phenol (1.89 g, 20.1 mmol), 1-bromo-3-phenylpropane (1.00 g, 5.02 mmol), potassium carbonate (2.76 g, 20.1 mmol) and acetone (50 mL). The reaction mixture was heated at 60 °SC for 48 h. Purification by flash chromatography eluting with 5% diethyl ether in hexane gave 3-phenoxypropylbenzene (**6**) as a colourless oil (1.05 g, 98%). Spectroscopic data were in agreement with the literature data.[17] ^{1}H-NMR (400 MHz, $CDCl_3$) 2.08–2.24 (2H, m, 2-H_2), 2.84 (2H, t, *J* = 7.6 Hz, 3-CH_2), 3.99 (2H, t, *J* = 6.3 Hz, 1-H_2), 6.89–6.99 (3H, m, 3 × CH), 7.19–7.35 (7H, m, 7 × CH); ^{13}C-NMR (101 MHz, $CDCl_3$) 31.0 (CH_2), 32.3 (CH_2), 66.9 (CH_2), 114.7 (2 × CH), 120.7 (CH), 126.1 (CH), 128.6 (2 × CH), 128.7 (2 × CH), 129.6 (2 × CH), 141.7 (C), 159.2 (C); *m/z* (EI) 212 (M^+, 100), 118 (70), 103 (40), 91 (95).

1-Methyl-4-(phenoxymethyl)benzene (S28)[18]

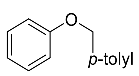

1-Methyl-4-(phenoxymethyl)benzene (**S28**) was synthesised as described for **S21** using phenol (0.282 g, 3.00 mmol), 4-methylbenzyl bromide (0.370 g, 0.300 mmol), potassium carbonate (0.553 g, 4.00 mmol) and acetone (10 mL). Purification by flash chromatography eluting with 10% diethyl ether in hexane which gave 1-methyl-4-(phenoxymethyl)benzene (**S28**) as a white solid (0.198 g, 97%). Spectroscopic data were in agreement with the literature data.[18] Mp 80–81 °C (lit.[19] 81.5–82.0 °C);

^{1}H-NMR (400 MHz, $CDCl_3$) 2.40 (3H, s, CH_3), 5.06 (2H, s, CH_2), 6.95–7.04 (3H, m, 3 × CH), 7.23 (2H, d, J = 7.8 Hz, 2 × CH), 7.27–7.39 (4H, m, 4 × CH); ^{13}C NMR (101 MHz, $CDCl_3$) 20.7 (CH_3), 69.4 (CH_2), 114.4 (2 × CH), 120.4 (C), 127.1 (2 × CH), 128.8 (2 × CH), 129.0 (2 × CH), 133.6 (C), 137.2 (C), 158.4 (C); m/z (EI) 198 (M^+, 80), 103 (100), 79.2 (100), 65 (100), 51 (80).

3-Methoxypropylbenzene (25)[20]

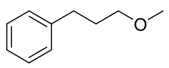

To a solution of 3-phenylpropanol (0.272 g, 2.00 mmol) in anhydrous THF (10 mL) was added sodium hydride (60% in mineral oil, 0.160 g, 4.00 mmol) and this was stirred at rt for 0.5 h. Methyl iodide (0.851 g, 6.00 mmol) was added dropwise to the reaction mixture and the resulting solution was stirred at rt for 18 h. The reaction mixture was quenched with the addition of water (40 mL), and then extraction of the aqueous solution with dichloromethane (3 × 30 mL). The organic fractions were combined, washed with water (40 mL), brine (40 mL), dried over $MgSO_4$ and concentrated *in vacuo*. The resulting residue was purified by flash chromatography with silica gel eluting with 40% diethyl ether in hexane and this gave 3-methoxypropylbenzene (**25**) as a colourless oil (0.249 g, 83%). Spectroscopic data were in agreement with the literature data.[20] ^{1}H-NMR (400 MHz, $CDCl_3$) 1.79–2.03 (2H, m, CH_2), 2.69 (2H, t, J = 7.6 Hz, CH_2), 3.35 (3H, s, CH_3), 3.39 (2H, t, J = 6.4 Hz, CH_2), 7.15–7.23 (3H, m, 3 × CH), 7.25–7.33 (2H, m, 2 × CH); ^{13}C-NMR (101 MHz, $CDCl_3$) 31.4 (CH_2), 32.4 (CH_2), 58.7 (CH_3), 72.1 (CH_2), 125.9 (CH), 128.5 (2 × CH), 128.6 (2 × CH), 142.1 (C); m/z (EI) 150 (M^+. 5), 117 (100), 103 (20), 91 (60), 77 (30), 65 (35), 51 (20).

Giese coupling reactions

Dimethyl 2-(phenoxymethyl)succinate (20a)[21]

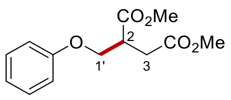

A solution of dimethyl fumarate (0.0432 g, 0.300 mmol), anisole (0.0973 g, 0.900 mmol), disodium hydrogen phosphate (0.127 g, 0.900 mmol) and acridinium catalyst **10** (0.0172 g, 0.0300 mmol) in anhydrous 1,2-dichloroethane (3 mL) was sparged with argon for 5 min. The reaction mixture was stirred and irradiated with blue light (456 nm) for 24 h and, no attempt to cool the reaction was made. A successful reaction was normally accompanied with a colour change from yellow to brown and the microwave vial being warm to the touch after irradiation. The reaction mixture was initially filtered to remove the inorganic base and then the filtrate was purified by flash chromatography eluting with 20 to

30% diethyl ether in hexane and this gave dimethyl 2-(phenoxymethyl)succinate (**20a**) as a colourless oil (0.0612 g, 81%). Spectroscopic data were in agreement with the literature data.[21] ^{1}H-NMR (500 MHz, $CDCl_3$) 2.77 (1H, dd, J = 17.0, 5.8 Hz, 3-*H*H), 2.98 (1H, dd, J = 17.0, 7.9 Hz, 3-H*H*), 3.37 (1H, m, 2-H), 3.73 (s, 3H), 3.77 (s, 3H), 4.24 (d, J = 5.7 Hz, 1′-H_2), 6.91 (d, J = 8.1 Hz, 2H), 6.99 (t, J = 7.3 Hz, 1H), 7.30 (t, J = 8.0 Hz, 2H); ^{13}C-NMR (126 MHz, $CDCl_3$) 32.6 (CH_2), 41.5 (CH), 51.9 (CH_3), 52.3 (CH_3), 67.3 (CH_2), 114.6 (2 × CH), 121.3 (CH), 129.5 (2 × CH), 158.4 (C), 172.2 (C), 172.5 (C); *m/z* (EI) 252 (M^+, 30), 221 (10), 189 (60), 159 (100), 127 (80).

Dimethyl 2-[(4″-fluorophenoxy)methyl]succinate (20b)

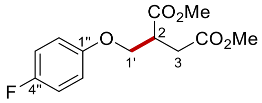

Dimethyl 2-[(4″-fluorophenoxy)methyl]succinate (**20b**) was synthesised as described for **20a** using dimethyl fumarate (0.0432 g, 0.300 mmol), and 4-fluoroanisole (0.114 g, 0.900 mmol). Purification by flash chromatography, eluting with 30% diethyl ether in hexane, gave dimethyl 2-[(4″-fluorophenoxy)methyl]succinate (**20b**) as a colourless oil (0.0674 g, 83%). ν_{max}/cm^{-1} (neat) 2955, 1736, 1506, 1437, 1202, 829; ^{1}H-NMR (400 MHz, $CDCl_3$) 2.72 (1H, dd, J = 17.0, 5.9 Hz, 3-*H*H), 2.93 (1H, dd, J = 17.0, 7.7 Hz, 3-H*H*), 3.31 (1H, app. dq, J = 7.7, 5.7 Hz, 2-H), 3.70 (3H, s, CH_3), 3.73 (3H, s, CH_3), 4.17 (2H, d, J = 5.7 Hz, 1′-H_2), 6.77–6.87 (2H, m, 2 × CH), 6.91–7.01 (2H, m, 2 × CH); ^{13}C-NMR (101 MHz, $CDCl_3$) 32.7 (CH_2), 41.6 (CH), 52.0 (CH_3), 52.4 (CH_3), 68.2 (CH_2), 115.9 (d, $^{3}J_{CF}$ = 6.0 Hz, 2 × CH), 116.0 (d, $^{2}J_{CF}$ = 22.1 Hz, 2 × CH), 154.6 (C), 157.7 (d, $^{1}J_{CF}$ = 238.9 Hz, C), 172.3 (C), 172.6 (C); *m/z* (ESI) 271.0972 ($[M+H]^+$. $C_{13}H_{16}O_5F^+$ requires 271.0976).

Dimethyl 2-[(4″-chlorophenoxy)methyl]succinate (20c)

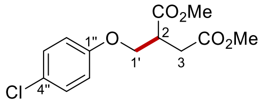

Dimethyl 2-[(4″-chlorophenoxy)methyl]succinate (**20c**) was synthesised as described for **20a** using 4-chloroanisole (0.0428 g, 0.300 mmol), and dimethyl fumarate (0.129 g, 0.900 mmol). Purification by flash chromatography eluting with 20 to 50% diethyl ether in hexane gave dimethyl 2-[(4″-chlorophenoxy)methyl]succinate (**20c**) as a colourless oil (0.0484 g, 56%). ν_{max}/cm^{-1} (neat) 2953, 1732, 1493, 1437, 1238, 1167, 1005, 826; ^{1}H-NMR (400 MHz, $CDCl_3$) 2.72 (1H, dd, J = 17.0, 5.7 Hz, 3-*H*H), 2.93 (1H, dd, J = 17.0, 7.6 Hz, 3-H*H*), 3.32 (1H, app. dq, J = 7.6, 5.7 Hz, 2-H), 3.70 (3H, s, CH_3), 3.74 (3H, s, CH_3), 4.18 (2H, d, J = 5.7 Hz, 1′-H_2), 6.81 (2H, d, J = 9.0 Hz, 2 × CH), 7.22 (2H, d, J = 9.0 Hz,

2 × CH); ^{13}C-NMR (101 MHz, $CDCl_3$) 32.6 (CH_2), 41.6 (CH), 52.1 (CH_3), 52.5 (CH_3), 67.8 (CH_2), 116.1 (2 × CH), 126.3 (C), 129.5 (2 × CH), 157.1 (C), 172.2 (C), 172.5 (C); *m/z* (ESI) 287.0676 ($[M+H]^+$. $C_{13}H_{16}O_5{}^{35}Cl^+$ requires 287.0681).

Dimethyl 2-((4″-(2‴-methyl-1‴,3‴-dioxolan-2‴-yl)phenoxy)methyl)succinate (20d)

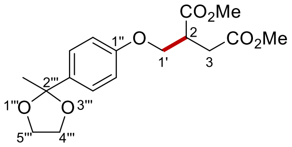

Dimethyl 2-((4″-(2‴-methyl-1‴,3‴-dioxolan-2‴-yl)phenoxy)methyl)succinate (**20d**) was synthesised as described for **20a** using dimethyl fumarate (0.0432 g, 0.300 mmol), 2-(4′-methoxyphenyl)-2-methyl-1,3-dioxolane (**S20**, 0.175 g, 0.900 mmol) and purple light. Purification with flash chromatography eluting with 40 to 60% diethyl ether in hexane gave dimethyl 2-((4″-(2‴-methyl-1‴,3‴-dioxolan-2‴-yl)phenoxy)methyl)succinate (**20d**) as a yellow oil (0.0605 g, 60%). ν_{max}/cm^{-1} (neat) 2959, 1672, 1597, 1576, 1248, 1169, 1026, 831; ^{1}H-NMR (400 MHz, $CDCl_3$) 1.63 (3H, s, 2‴-CH_3), 2.72 (1H, dd, J = 17.0, 5.8 Hz, 3-*H*H), 2.94 (1H, dd, J = 17.0, 7.8 Hz, 3-H*H*), 3.33 (1H, app. dq, J = 7.8, 5.7 Hz, 2-H), 3.70 (3H, s, OCH_3), 3.71–3.82 (5H, m, OCH_3, 4‴-*H*H and 5‴-*H*H), 3.96–4.07 (2H, m, 4‴-H*H* and 5‴-H*H*), 4.20 (2H, d, J = 5.5 Hz, 1′-H_2), 6.84 (2H, d, J = 8.9 Hz, 2 × CH), 7.38 (2H, d, J = 8.9 Hz, 2 × CH); ^{13}C-NMR (101 MHz, $CDCl_3$) 27.8 (CH_3), 32.7 (CH_2), 41.6 (CH), 52.0 (CH_3), 52.4 (CH_3), 64.5 (2 × CH_2), 67.5 (CH_2), 108.9 (C), 114.3 (2 × CH), 126.7 (2 × CH), 136.3 (C), 158.1 (C), 172.3 (C), 172.6 (C); *m/z* (ESI) 339.1436 ($[M+H]^+$ $C_{17}H_{23}O_7$ requires 339.1438).

Dimethyl 2-[(3″-methoxylphenoxy)methyl]succinate (20e)

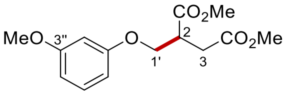

Dimethyl 2-[(3″-methoxylphenoxy)methyl]succinate (**20e**) was synthesised as described for **20a** using dimethyl fumarate (0.0432 g, 0.300 mmol), 3-methoxyanisole (0.124 g, 0.900 mmol) and purple light. Purification by flash chromatography eluting with 50 to 60% diethyl ether in hexane gave dimethyl 2-[(3″-methoxylphenoxy)methyl]succinate (**20e**) as a yellow oil (0.0570 g, 67%). ν_{max}/cm^{-1} (neat): 2953, 1732, 1591, 1437, 1198, 1080, 1038; ^{1}H-NMR (400 MHz, $CDCl_3$) 2.72 (1H, dd, J = 17.0, 5.8 Hz, 3-*H*H), 2.94 (1H, dd, J = 17.0, 7.9 Hz, 3-H*H*), 3.32 (1H, app. dq, J = 7.9, 5.6 Hz, 2-H), 3.70 (3H, s, OCH_3), 3.73 (3H, s, OCH_3), 3.78 (3H, s, OCH_3), 4.19 (2H, d, J = 5.5 Hz, 1′-H_2), 6.44 (1H, app. t, J = 2.3 Hz, CH), 6.48 (1H, ddd, J = 8.2, 2.3, 0.6 Hz, CH), 6.52 (1H, ddd, J = 8.2, 2.3, 0.6 Hz, CH), 7.16 (1H, app. t, J = 8.2 Hz, CH); ^{13}C-NMR (101 MHz, $CDCl_3$) 32.7 (CH_2), 41.6 (CH), 52.0 (CH_3), 52.4 (CH_3), 55.4

(CH_3), 67.5 (CH_2), 101.2 (CH), 106.8 (CH), 107.1 (CH), 130.0 (CH), 159.7 (C), 160.1 (C), 172.3 (C), 172.6 (C); *m/z* (ESI) 283.1172 ($[M+H]^+$. $C_{14}H_{19}O_6$ requires 283.1176).

Dimethyl 2-[(4″-methoxyphenoxy)methyl]succinate (20f)[21]

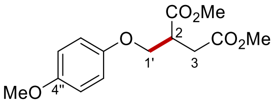

Dimethyl 2-[(4″-methoxyphenoxy)methyl]succinate (**20f**) was synthesised as described for **20a** using dimethyl fumarate (0.0432 g, 0.300 mmol), 1,4-dimethoxybenzene (0.124 g, 0.900 mmol) and blue light. Purification by flash chromatography eluting with 50 to 60% diethyl ether in hexane gave dimethyl 2-[(4″-methoxyphenoxy)methyl]succinate (**20f**) as a yellow oil (0.0144 g, 17%). Spectroscopic data were in agreement with the literature data.[21] ^{1}H-NMR (400 MHz, $CDCl_3$) 2.72 (1H, dd, J = 17.0, 5.8 Hz, 3-*H*H), 2.93 (1H, dd, J = 17.0, 7.9 Hz, 3-H*H*), 3.31 (1H, app. dq, J = 7.9, 5.6 Hz, 2-H), 3.70 (3H, s, CH_3), 3.73 (3H, s, CH_3), 3.76 (3H, s, CH_3), 4.14–4.16 (2H, m, 1′-H_2), 6.82 (4H, s, 4 × CH); ^{13}C-NMR (101 MHz, $CDCl_3$) 32.8 (CH_2), 41.8 (CH), 52.0 (CH_3), 52.4 (CH_3), 55.9 (CH_3), 68.4 (CH_2), 114.8 (2 × CH), 115.9 (2 × CH), 152.7 (C), 154.4 (C), 172.4 (C), 172.7 (C); *m/z* (APCI) 283.1166 ($[M+H]^+$. $C_{14}H_{19}O_6$ requires 283.1176).

Dimethyl 2-(1′-phenoxyeth-1′-yl)succinate (20g)

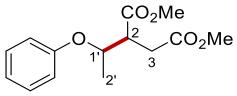

Dimethyl 2-(1′-phenoxyeth-1′-yl)succinate (**20g**) was synthesised as described for **20a** using ethyl phenyl ether (0.0366 g, 0.300 mmol), dimethyl fumarate (0.129 g, 0.900 mmol) and blue light. Purification by flash chromatography eluting with 20 to 30% diethyl ether in hexane gave dimethyl 2-(1′-phenoxyeth-1′-yl)succinate (**20g**) as a yellow oil and a 1.0:1.2 mixture of diastereoisomers that could not be separated by chromatography (0.0783 g, 98%). ν_{max}/cm^{-1} (neat) 2953, 1732, 1597, 1493, 1435, 1229, 1165, 754, 692; **data for major diastereoisomer:** ^{1}H-NMR (400 MHz, $CDCl_3$) 1.32 (3H, d, J = 6.2 Hz, 2′-H_3), 2.76 (1H, dd, J = 16.9, 4.5 Hz, 3-*H*H), 2.81–2.96 (1H, m, 3-H*H*), 3.15 (1H, ddd, J = 9.6, 6.0, 4.5 Hz, 2-H), 3.69 (6H, s, 2 × CH_3), 4.65–4.82 (1H, m, 1′-H), 6.84–7.02 (3H, m, 3 × CH), 7.21–7.34 (2H, m, 2 × CH); ^{13}C-NMR (101 MHz, $CDCl_3$) 17.9 (CH_3), 32.2 (CH_2), 47.7 (CH), 52.0 (CH_3), 52.2 (CH_3), 73.8 (CH), 116.4 (2 × CH), 121.6 (CH), 129.7 (2 × CH), 157.5 (C), 172.8 (2 × C); **Data for minor diastereoisomer:** ^{1}H-NMR (400 MHz, $CDCl_3$) 1.27 (3H, d, J = 6.3 Hz, 2′-H_3), 2.64 (1H, dd, J = 16.9, 4.0 Hz, 3-*H*H), 2.81–2.96 (1H, m, 3-H*H*), 3.40 (1H, ddd, J = 10.4, 4.7, 4.2 Hz, 2-H), 3.66 (3H, s, CH_3), 3.74 (3H, s, CH_3), 4.65–4.82 (1H, m, 1′-H), 6.84–7.02 (3H, m, 3 × CH), 7.21–7.34

(2H, m, 2 × CH); ^{13}C-NMR (101 MHz, $CDCl_3$) 16.5 (CH_3), 30.5 (CH_2), 45.6 (CH), 52.0 (CH_3), 52.3 (CH_3), 73.0 (CH), 116.2 (2 × CH), 121.6 (CH), 129.8 (2 × CH), 157.2 (C), 172.6 (C), 173.1 (C); *m/z* (ESI) 267.1222 ($[M+H]^+$. $C_{14}H_{19}O_5^+$ requires 267.1227).

Dimethyl 2-(1′-phenoxyhex-1′-yl)succinate (20h)

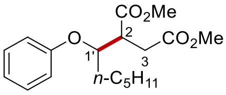

Dimethyl 2-(1′-phenoxyhex-1′-yl)succinate (**20h**) was synthesised as described for **20a** using dimethyl fumarate (0.0430 g, 0.300 mmol), hexyl phenyl ether (**S21**) (0.123 g, 0.900 mmol) and blue light. Purification by flash chromatography eluting with 0 to 30% ethyl acetate in hexane gave dimethyl 2-(1′-phenoxyhex-1′-yl)succinate (**20h**) as a brown oil and a 1.0:1.2 mixture of diastereoisomers that could not be separated by column chromatography (0.0730 g, 76%). ν_{max}/cm^{-1} (neat) 2951, 2928, 1736, 1597, 1587, 1491; **Data for major diastereoisomer:** ^{1}H-NMR (400 MHz, $CDCl_3$) 0.82–0.90 (3H, m, CH_3), 1.21–1.75 (8H, m, 4 × CH_2), 2.59–2.69 (1H, m, 3-*H*H), 2.78–3.02 (1H, m, 3-H*H*), 3.16–3.26 (1H, m, 2-H), 3.61 (3H, s, CH_3), 3.68 (3H, s, CH_3), 4.57–4.70 (1H, m, 1′-H), 6.84–7.00 (3H, m, 3 × CH), 7.22–7.32 (2H, m, 2 × CH); ^{13}C-NMR (101 MHz, $CDCl_3$) 13.4 (CH_3), 22.0 (CH_2), 24.4 (CH_2), 30.4 (CH_2), 30.8 (CH_2), 31.6 (CH_2), 44.8 (CH), 51.3 (CH_3), 51.4 (CH_3), 77.4 (CH), 115.5 (2 × CH), 120.9 (CH), 129.0 (2 × CH), 157.2 (C), 172.1 (2 × C); **Data for minor diastereoisomer:** ^{1}H-NMR (400 MHz, $CDCl_3$) 0.82–0.90 (3H, m, CH_3), 1.21–1.75 (8H, m, 4 × CH_2), 2.59–2.69 (1H, m, 3-*H*H), 2.78–3.02 (1H, m, 3-H*H*), 3.45 (1H, dt, J = 10.7, 4.0 Hz, 2-H), 3.64 (3H, s, CH_3), 3.74 (3H, s, CH_3), 4.57–4.70 (1H, m, 1′-H), 6.84–7.00 (3H, m, 3 × CH), 7.22–7.32 (2H, m, 2 × CH); ^{13}C-NMR (101 MHz, $CDCl_3$) 13.4 (CH_3), 22.0 (CH_2), 25.0 (CH_2), 29.7 (CH_2) 31.0 (CH_2), 31.2 (CH_2), 43.5 (CH), 51.5 (CH_3), 51.7 (CH_3), 77.4 (CH), 115.8 (2 × CH), 120.9 (CH), 129.2 (2 × CH), 157.6 (C), 172.3 (C), 172.7 (C); *m/z* (ESI) 323.1842 ($[M+H]^+$. $C_{18}H_{27}O_5^+$ requires 323.1853).

Dimethyl 2-[phenoxy(phenyl)methyl]succinate (20i)

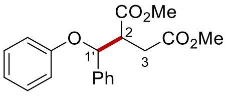

Dimethyl 2-[phenoxy(phenyl)methyl]succinate (**20i**) was synthesised as described for **20a** using phenyl benzyl ether (0.0553 g, 0.300 mmol), dimethyl fumarate (0.129 g, 0.900 mmol) and blue light. Purification by flash chromatography eluting with 20% to 30% diethyl ether in hexane gave dimethyl 2-[phenoxy(phenyl)methyl]succinate (**20i**) as a yellow oil and a 1.0:1.1 mixture of diastereoisomers that could not be separated by chromatography (0.0925 g, 93%). ν_{max}/cm^{-1} (neat) 2949, 1754, 1491,

1439, 1229, 1169, 750, 696; **Data for major diastereoisomer:** ^{1}H-NMR (400 MHz, $CDCl_3$) 2.57 (1H, dd, *J* = 16.9, 4.0 Hz, 3-*H*H), 3.04 (1H, dd, *J* = 16.9, 10.4 Hz, 3-H*H*), 3.39 (1H, ddd, *J* = 10.4, 5.2, 4.0 Hz, 2-H), 3.62 (3H, s, CH_3), 3.74 (3H, s, CH_3), 5.61 (1H, d, *J* = 5.2 Hz, 1′-H), 6.75–6.94 (3H, m, 3 × CH), 7.11–7.21 (2H, m, 2 × CH), 7.24–7.40 (5H, m, 5 × CH); ^{13}C-NMR (101 MHz, $CDCl_3$) 31.1 (CH_2), 49.4 (CH), 52.3 (2 × CH_3), 79.6 (CH), 116.4 (2 × CH), 121.6 (CH), 126.4 (2 × CH), 128.3 (CH), 129.5 (4 × CH), 137.2 (C), 157.8 (C), 172.1 (C), 172.3 (C); **Data for minor diastereoisomer:** ^{1}H-NMR (400 MHz, $CDCl_3$) 2.46 (1H, dd, *J* = 16.7, 4.4 Hz, 3-*H*H), 2.64 (1H, dd, *J* = 16.7, 10.3 Hz, 3-H*H*), 3.51 (1H, ddd, *J* = 10.3, 7.5, 4.4 Hz, 2-H), 3.59 (3H, s, CH_3), 3.61 (3H, s, CH_3), 5.40 (1H, d, *J* = 7.5 Hz, 1′-H), 6.75–6.94 (3H, m, 3 × CH), 7.11–7.21 (2H, m, 2 × CH), 7.24–7.40 (5H, m, 5 × CH); ^{13}C-NMR (101 MHz, $CDCl_3$) 32.5 (CH_2), 48.6 (CH), 51.9 (CH_3), 52.0 (CH_3), 80.4 (CH), 116.0 (2 × CH), 121.5 (CH), 127.0 (2 × CH), 128.7 (CH), 128.9 (4 × CH), 138.5 (C), 157.6 (C), 172.0 (C), 172.4 (C); *m/z* (ESI) 329.1370 ($[M+H]^+$. $C_{19}H_{21}O_5^+$ requires 329.1384).

Dimethyl 2-[3′-(1″,3″-dioxoisoindolin-2″-yl)-1′-phenoxyprop-1′-yl]succinate (20j)

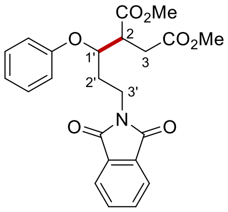

Dimethyl 2-[3′-(1″,3″-dioxoisoindolin-2″-yl)-1′-phenoxyprop-1′-yl]succinate (**20j**) was synthesised as described for **20a** using 3-(1′,3′-dioxoisoindolin-2′-yl)propyl phenyl ether (**S22**) (0.0850 g, 0.300 mmol), dimethyl fumarate (0.129 g, 0.900 mmol) and blue light. Purification by flash chromatography eluting with 0 to 50% ethyl acetate in hexane gave dimethyl 2-[3′-(1″,3″-dioxoisoindolin-2″-yl)-1′-phenoxyprop-1′-yl]succinate (**20j**) as a brown gum and a 1.0:1.5 mixture of diastereoisomers that could not be separated by chromatography (0.0940 g, 74%). ν_{max}/cm^{-1} (neat): 2951, 1732, 1707, 1587, 1489; **Data for major diastereoisomer:** ^{1}H-NMR (400 MHz, $CDCl_3$) 1.80–2.17 (2H, m, 2′-H_2), 2.67 (1H, dd, *J* = 17.0, 4.4Hz, 3-*H*H), 2.90 (1H, dd, *J* = 17.0, 9.8 Hz, 3-H*H*), 3.24–3.32 (1H, m, 2-H), 3.63 (3H, s, CH_3), 3.66 (3H, s, CH_3), 3.75–3.94 (2H, m, 3′-H_2), 4.70–4.81 (1H, m, 1′-H), 6.86–6.98 (3H, m, 3 × CH), 7.19–7.26 (2H, m, 2 × CH), 7.66–7.73 (2H, m, 2 × CH), 7.76–7.84 (2H, m, 2 × CH); ^{13}C-NMR (101 MHz, $CDCl_3$) : 29.3 (CH_2), 30.6 (CH_2), 34.1 (CH_2), 44.7 (CH), 51.7 (CH_3), 51.8 (CH_3), 75.4 (CH), 116.0 (2 × CH), 121.4 (CH), 122.7 (2 × CH), 129.1 (2 × CH), 131.6 (2 × C), 133.4 (2 × CH), 157.0 (C), 167.7 (2 × C), 171.8 (C), 172.1 (C); **Data for minor diastereoisomer:** ^{1}H-NMR (400 MHz, $CDCl_3$) 1.80–2.17 (2H, m, 2′-H_2), 2.60 (1H, dd, *J* = 16.9, 3.7 Hz, 3-*H*H), 2.79 (1H, dd, *J* = 16.9, 10.8 Hz, 3-H*H*), 3.48 (1H, dt, *J* = 10.7, 3.9 Hz, 2-H), 3.62 (3H, s, CH_3), 3.70 (3H, s, CH_3), 3.75–3.94 (2H, m, 3′-H_2), 4.70–4.81 (1H, m, 1′-H), 6.86–6.98 (3H, m, 3 × CH), 7.19–7.26 (2H, m, 2 × CH), 7.66–7.73 (2H,

m, 2 × CH), 7.76–7.84 (2H, m, 2 × CH); ^{13}C-NMR (101 MHz, $CDCl_3$) 29.3 (CH_2), 31.3 (CH_2), 34.8 (CH_2), 43.1 (CH), 51.4 (2 × CH_3), 74.4 (CH), 115.5 (2 × CH), 121.3 (CH), 122.7 (2 × CH), 129.2 (2 × CH), 131.6 (2 × C), 133.4 (2 × CH), 156.3 (C), 167.6 (2 × C), 171.6 (C), 171.8 (C); *m/z* (ESI) 426.1534 ($[M+H]^+$. $C_{23}H_{24}NO_7^+$ requires 426.1547).

Dimethyl 2-(3′-acetoxy-1′-phenoxypropyl)succinate (20k)

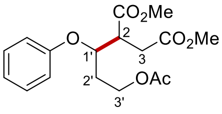

Dimethyl 2-(3′-acetoxy-1′-phenoxypropyl)succinate (**20k**) was synthesised as described for **20a** using 3-phenoxypropyl acetate (**S23**) (0.0583 g, 0.300 mmol), dimethyl fumarate (0.129 g, 0.900 mmol) and blue light. Purification by flash chromatography eluting with 60% diethyl ether in hexane gave dimethyl 2-(3′-acetoxy-1′-phenoxypropyl)succinate (**20k**) as a yellow oil and a 1.0:1.2 mixture of diastereoisomers that could not be separated by chromatography (0.0939 g, 93%). ν_{max}/cm^{-1} (neat) 2953, 1732, 1597, 1491, 1437, 1366, 1223, 1165, 1047, 754, 692; **Data for major diastereoisomer:** ^{1}H-NMR (400 MHz, $CDCl_3$) 1.80–2.10 (5H, m, CH_3 and 2′-H_2), 2.56–2.72 (1H, m, 3-*H*H), 2.91 (1H, dd, *J* = 17.0, 9.7 Hz, 3-H*H*), 3.23 (1H, ddd, *J* = 9.7, 5.0, 4.5 Hz, 2-H), 3.62 (3H, s, CH_3), 3.67 (3H, s, CH_3), 4.06–4.17 (1H, m, 3′-*H*H), 4.19–4.30 (1H, m, 3′-H*H*), 4.72–4.87 (1H, m, 1′-H), 6.87–7.02 (3H, m, 3 × CH), 7.21–7.34 (2H, m, 2 × CH) ^{13}C-NMR (101 MHz, $CDCl_3$) 21.0 (CH_3), 30.5 (CH_2), 31.7 (CH_2), 45.7 (CH), 52.0 (CH_3), 52.3 (CH_3), 60.7 (CH_2), 75.0 (CH), 116.4 (2 × CH), 121.9 (CH), 129.7 (2 × CH), 158.0 (C), 170.9 (C), 172.5 (C), 172.8 (C); **Data for minor diastereoisomer:** ^{1}H-NMR (400 MHz, $CDCl_3$) 1.80–2.10 (5H, m, CH_3 and 2′-H_2), 2.56–2.72 (1H, m, 3-*H*H), 2.83 (1H, dd, *J* = 16.9, 10.5 Hz, 3-H*H*), 3.48 (1H, dt, *J* = 10.5, 4.1 Hz, 2-H), 3.64 (3H, s, CH_3), 3.74 (3H, s, CH_3), 4.06–4.17 (1H, m, 3′-*H*H), 4.19–4.30 (1H, m, 3′-H*H*), 4.72–4.87 (1H, m, 1′-H), 6.87–7.02 (3H, m, 3 × CH), 7.21–7.34 (2H, m, 2 × CH); ^{13}C-NMR (101 MHz, $CDCl_3$) 21.0 (CH_3), 30.3 (CH_2), 31.7 (CH_2), 44.1 (CH), 52.0 (CH_3), 52.4 (CH_3), 61.0 (CH_2), 74.0 (CH), 116.1 (2 × CH), 122.0 (CH), 129.9 (2 × CH), 157.4 (C), 170.9 (C), 172.3 (C), 172.4 (C); *m/z* (ESI) 339.1434 ($[M+H]^+$. $C_{17}H_{23}O_7^+$ requires 339.1438).

Dimethyl 2-(3′-benzoyloxy-1′-phenoxypropyl)succinate (20l)

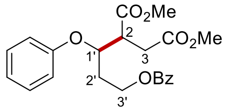

Dimethyl 2-(3′-benzoyloxy-1′-phenoxypropyl)succinate (**20l**) was synthesised as described for **20a** using 3-phenoxypropyl benzoate (**S24**) (0.0487 g, 0.300 mmol), dimethyl fumarate (0.129 g, 0.900 mmol) and blue light. Purification by flash chromatography eluting with 30 to 50% diethyl ether in hexane gave dimethyl 2-(3′-benzoyloxy-1′-phenoxypropyl)succinate (**20l**) as a yellow oil and a 1.0:1.3 mixture of diastereoisomers that could not be separated by chromatography (0.122 g, 100%). ν_{max}/cm^{-1} (neat) 2951, 1719, 1597, 1491, 1437, 1225, 1167, 1111, 1026, 752, 711, 692; **Data for major diastereoisomer:** ^{1}H-NMR (400 MHz, $CDCl_3$) 1.96–2.24 (2H, m, 2′-H_2), 2.60–2.73 (1H, m, 3-*H*H), 2.94 (1H, dd, *J* = 17.0, 9.7 Hz, 3-H*H*), 3.31 (1H, dt, *J* = 9.7, 4.8 Hz, 2-H), 3.62 (3H, s, CH_3), 3.66 (3H, s, CH_3), 4.33–4.43 (1H, m, 3′-*H*H), 4.47–4.57 (1H, m, 3′-H*H*), 4.84–4.96 (1H, m, 1′-H), 6.89–7.01 (3H, m, 3 × CH), 7.19–7.32 (2H, m, 2 × CH), 7.38–7.49 (2H, m, 2 × CH), 7.52–7.61 (1H, m, CH), 7.96–8.04 (2H, m, 2 × CH); ^{13}C-NMR (101 MHz, $CDCl_3$) 30.7 (CH_2), 31.8 (CH_2), 45.7 (CH), 52.0 (CH_3), 52.3 (CH_3), 61.3 (CH_2), 75.0 (CH), 116.4 (2 × CH), 122.0 (CH), 128.5 (2 × CH), 129.7 (2 × CH), 129.8 (2 × CH), 130.2 (C), 133.2 (CH), 157.9 (C), 166.5 (C), 172.4 (C), 172.8 (C); **Data for minor diastereoisomer:** ^{1}H-NMR (400 MHz, $CDCl_3$) 1.96–2.24 (2H, m, 2′-H_2), 2.60–2.73 (1H, m, 3-*H*H), 2.86 (1H, dd, *J* = 16.8, 10.5 Hz, 3-H*H*), 3.53 (1H, dt, *J* = 10.5, 4.1 Hz, 2-H), 3.64 (3H, s, CH_3), 3.71 (3H, s, CH_3), 4.33–4.43 (1H, m, 3′-*H*H), 4.47–4.57 (1H, m, 3′-H*H*), 4.84–4.96 (1H, m, 1′-H), 6.89–7.01 (3H, m, 3 × CH), 7.19–7.32 (2H, m, 2 × CH), 7.38–7.49 (2H, m, 2 × CH), 7.52–7.61 (1H, m, CH), 7.96–8.04 (2H, m, 2 × CH); ^{13}C-NMR (101 MHz, $CDCl_3$) 30.2 (CH_2), 31.9 (CH_2), 44.1 (CH), 52.0 (CH_3), 52.4 (CH_3), 61.5 (CH_2), 74.0 (CH), 116.2 (2 × CH), 122.0 (CH), 128.5 (2 × CH), 129.7 (2 × CH), 129.9 (2 × CH), 130.2 (C), 133.2 (CH), 157.4 (C), 166.4 (C), 172.3 (C), 172.4 (C); *m/z* (ESI) 401.1591 ($[M+H]^+$. $C_{22}H_{25}O_7^+$ requires 401.1595).

Dimethyl 2-(2′-phenoxypropan-2′-yl)succinate (20m)[22]

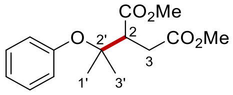

Dimethyl 2-(2′-phenoxypropan-2′-yl)succinate (**20m**) was synthesised as described for **20a** using dimethyl fumarate (0.0432 g, 0.300 mmol), isopropyl phenyl ether (**S25**) (0.123 g, 0.900 mmol) and blue light. Purification by flash chromatography eluting with 0 to 25% ethyl acetate in toluene gave dimethyl 2-(2′-phenoxypropan-2′-yl)succinate (**20m**) as a brown oil (0.068 g, 81%). Spectroscopic data were in agreement with the literature data.[22] ^{1}H-NMR (400 MHz, $CDCl_3$) 1.29 (3H, s, 1′-H_3), 1.31 (3H,

s, 3′-H$_3$), 2.87–3.04 (2H, m, 3-H$_2$), 3.29 (1H, dd, J = 10.3, 4.3 Hz, 2-H), 3.69 (3H, s, CH$_3$), 3.74 (3H, s, CH$_3$), 6.93–6.99 (2H, m, 2 × CH), 7.10 (1H, tt, J = 7.4, 1.2 Hz, CH), 7.20–7.30 (2H, m, 2 × CH); ^{13}C-NMR (101 MHz, CDCl$_3$) 23.1 (CH$_3$), 25.1 (CH$_3$), 31.9 (CH$_2$), 51.1 (CH), 51.4 (CH$_3$), 51.4 (CH$_3$), 79.4 (C), 123.5 (CH), 123.7 (2 × CH), 128.6 (2 × CH), 153.7 (C), 172.4 (C), 172.9 (C); *m/z* (ESI) 281.1382 ([M+H]$^+$. $C_{15}H_{21}O_5^+$ requires 281.1384.

Dimethyl 2-(1′-phenoxycyclobut-1′-yl)succinate (20n)

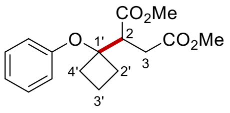

Dimethyl 2-(1′-phenoxycyclobut-1′-yl)succinate (**20n**) was synthesised as described for **20a** using cyclobutyl phenyl ether (**S26**) (0.0445 g, 0.300 mmol), dimethyl fumarate (0.129 g, 0.900 mmol) and blue light. Purification by flash chromatography eluting with 30 to 50% diethyl ether in hexane gave dimethyl 2-(1′-phenoxycyclobut-1′-yl)succinate (**20n**) as a brown oil (0.0644 g, 73%). ν_{max}/cm^{-1} (neat) 2951, 1732, 1585, 1489, 1435, 1217, 1157, 754, 694; ^{1}H-NMR (400 MHz, CDCl$_3$) 1.41–1.56 (1H, m, 3′-*H*H), 1.71–1.84 (1H, m, 3′-H*H*), 2.25–2.38 (1H, m, 2′-*H*H), 2.41–2.67 (4H, m, 3-*H*H, 2′-H*H* and 4′-H$_2$), 2.95 (1H, dd, J = 17.4, 11.2 Hz, 3-H*H*), 3.54–3.63 (4H, m, CH$_3$ and 2-H), 3.77 (3H, s, CH$_3$), 6.79–6.86 (2H, m, 2 × CH), 6.95–7.01 (1H, m, CH), 7.22–7.30 (2H, m, 2 × CH); ^{13}C-NMR (101 MHz, CDCl$_3$) 12.7 (CH$_2$), 30.3 (CH$_2$), 30.6 (CH$_2$), 31.2 (CH$_2$), 44.9 (CH), 51.4 (CH$_3$), 51.7 (CH$_3$), 81.2 (C), 118.2 (2 × CH), 121.3 (CH), 129.0 (2 × CH), 153.9 (C), 172.4 (2 × C); *m/z* (ESI) 293.1376 ([M+H]$^+$. $C_{16}H_{21}O_5^+$ requires 293.1384).

Dimethyl 2-(1′-phenoxycyclopent-1′-yl)succinate (20o)

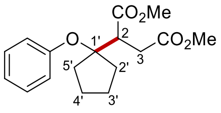

Dimethyl 2-(1′-phenoxycyclopent-1′-yl)succinate (**20o**) was synthesised as described for **20a** using cyclopentyl phenyl ether (**S27**) (0.0487 g, 0.300 mmol), dimethyl fumarate (0.129 g, 0.900 mmol) and blue light. Purification by flash chromatography eluting with 30 to 50% diethyl ether in hexane gave dimethyl 2-(1′-phenoxycyclopent-1′-yl)succinate (**20o**) as a yellow oil (0.0576 g, 63%). ν_{max}/cm^{-1} (neat) 2951, 1734, 1593, 1487, 1437, 1225, 978, 696; ^{1}H-NMR (400 MHz, CDCl$_3$) 1.44–2.21 (8H, m, 4 × CH$_2$), 2.82 (1H, dd, J = 17.2, 3.3 Hz, 3-*H*H), 2.98 (1H, dd, J = 17.2, 11.4 Hz, 3-H*H*), 3.64 (3H, s, CH$_3$). 3.68–3.79 (4H, m, CH$_3$ and 2-H), 6.95 (2H, dd, J = 8.6, 0.9 Hz, 2 × CH), 7.01 (1H, t, J = 7.4 Hz, CH), 7.23–7.29 (2H, m, 2 × CH); ^{13}C-NMR (101 MHz, CDCl$_3$) 23.1 (CH$_2$). 24.0 (CH$_2$), 32.2 (CH$_2$), 33.9

(CH_2), 34.9 (CH_2), 46.8 (CH), 51.3 (CH_3), 51.5 (CH_3), 90.2 (C), 120.4 (2 × CH), 121.9 (CH), 128.8 (2 × CH), 154.3 (C), 172.3 (C), 172.7 (C); *m/z* (ESI) 329.1352 ($[M+Na]^+$. $C_{17}H_{22}O_5Na^+$ requires 329.1359).

Dimethyl 2-[(3″-methylphenoxy)methyl]succinate (20p)

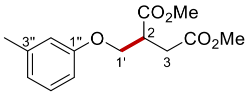

Dimethyl 2-[(3″-methylphenoxy)methyl]succinate (**20p**) was synthesised as described for **20a** using dimethyl fumarate (0.0432 g, 0.300 mmol), 3-methylanisole (0.109 g, 0.900 mmol) and blue light. Purification by flash chromatography, eluting with 30% diethyl ether in hexane, gave dimethyl 2-[(3″-methylphenoxy)methyl]succinate (**20p**) as a colourless oil (0.0566 g, 71%). ν_{max}/cm^{-1} (neat) 2954, 1736, 1437, 1260, 1157, 777; ^{1}H-NMR (400 MHz, $CDCl_3$) 2.32 (3H, s, 3″-CH_3), 2.73 (1H, dd, *J* = 17.0, 5.7 Hz, 3-*H*H), 2.94 (1H, dd, *J* = 17.0, 7.9 Hz, 3-H*H*), 3.33 (1H, app. dq, *J* = 7.9, 5.7 Hz, 2-H), 3.70 (3H, s, CH_3), 3.74 (3H, s, CH_3), 4.17–4.23 (2H, m, 1′-H_2), 6.65–6.74 (2H, m, 2 × CH), 6.78 (1H, d, *J* = 7.6 Hz, CH), 7.16 (1H, t, *J* = 7.6 Hz, CH); ^{13}C-NMR (101 MHz, $CDCl_3$) 21.6 (CH_3), 32.7 (CH_2), 41.7 (CH), 52.0 (CH_3), 52.4 (CH_3), 67.4 (CH_2), 111.6 (CH), 115.6 (CH), 122.2 (CH), 129.3 (CH), 139.7 (C), 158.5 (C), 172.3 (C), 172.7 (C); *m/z* (ESI) 267.1223 ($[M+H]^+$. $C_{14}H_{19}O_5^+$ requires 267.1227).

Dimethyl 2-[(4″-methoxyphenyl)methyl]succinate (20q)[23]

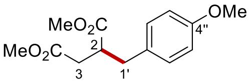

Dimethyl 2-[(4″-methoxyphenyl)methyl]succinate (**20q**) was synthesised as described for **20a** using 4-methylanisole (0.0367 g, 0.300 mmol), dimethyl fumarate (0.129 g, 0.900 mmol) and blue light. Purification by flash chromatography eluting with 20 to 50% diethyl ether in hexane gave dimethyl 2-[(4″-methoxyphenyl)methyl]succinate (**20q**) as an oil (0.0539 g, 67%). Spectroscopic data were in agreement with the literature data.[23] ^{1}H-NMR (400 MHz, $CDCl_3$) 2.40 (1H, dd, *J* = 16.8, 5.0 Hz, 3-*H*H), 2.60–2.75 (2H, m, 3-H*H* and 1′-*H*H), 2.98 (1H, dd, *J* = 13.7, 6.3 Hz, 1′-H*H*), 3.04–3.13 (1H, m, 2-H), 3.64 (3H, s, CH_3), 3.67 (3H, s, CH_3), 3.78 (CH_3), 6.82 (2H, d, *J* 8.7 Hz, 2 × CH), 7.06 (2H, d, *J* = 8.7 Hz, 2 × CH); ^{13}C-NMR (101 MHz, $CDCl_3$) 34.4 (CH_2), 36.4 (CH_2), 42.7 (CH), 51.2 (CH_3), 51.4 (CH_3), 54.7 (CH_3), 113.5 (2 × CH), 129.5 (2 × CH), 129.6 (C), 157.9 (C), 171.8 (C), 174.3 (C); *m/z* (ESI) 267 ($[M+H]^+$, 100%).

Dimethyl 2-(1′-phenoxy-3′-phenylprop-1′-yl)succinate (20r)

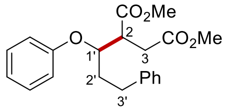

Dimethyl 2-(1′-phenoxy-3′-phenylprop-1′-yl)succinate (**20r**) was synthesised as described for **20a** using 3-phenoxypropylbenzene (**6**) (0.0637 g, 0.300 mmol), dimethyl fumarate (0.129 g, 0.900 mmol) and blue light. Purification by flash chromatography eluting with 20 to 50% diethyl ether in hexane gave dimethyl 2-(1′-phenoxy-3′-phenylprop-1′-yl)succinate (**20r**) as a yellow oil and a 1.0:1.2 mixture of diastereoisomers that could not be separated by chromatography (0.0791 g, 74%). ν_{max}/cm^{-1} (neat) 2951, 1732, 1597, 1435, 1225, 1163,1030, 752, 692; **Data for major diastereoisomer:** ^{1}H-NMR (400 MHz, $CDCl_3$) 1.73–2.10 (2H, m, 2′-H_2), 2.55–3.00 (4H, m, 3-H_2 and 3′-H_2), 3.23–3.34 (1H, m, 2-H), 3.63 (3H, s, CH_3), 3.67 (3H, s, CH_3), 4.57–4.72 (1H, m, 1′-H), 6.82–7.03 (3H, m, 3 × CH), 7.09–7.36 (7H, m, 7 × CH); ^{13}C-NMR (101 MHz, $CDCl_3$) 31.6 (CH_2), 31.7 (CH_2), 34.1 (CH_2), 45.4 (CH), 52.0 (CH_3), 52.2 (CH_3), 77.1 (CH), 116.5 (2 × CH), 121.7 (CH), 126.2 (CH), 128.5 (2 × CH), 128.6 (2 × CH), 129.7 (2 × CH), 141.2 (C), 158.1 (C), 172.7 (C), 173.1 (C); **data for minor diastereoisomer:** ^{1}H-NMR (400 MHz, $CDCl_3$) 1.73–2.10 (2H, m, 2′-H_2), 2.55–3.00 (4H, m, 3-H_2 and 3′-H_2), 3.41–3.52 (1H, dt, *J* = 10.6, 3.9 Hz, 2-H), 3.64 (3H, s, CH_3), 3.67 (3H, s, CH_3), 4.57–4.72 (1H, m, 1′-H), 6.82–7.03 (3H, m, 3 × CH), 7.09–7.36 (7H, m, 7 × CH); ^{13}C-NMR (101 MHz, $CDCl_3$) 30.2 (CH_2), 32.0 (CH_2), 32.8 (CH_2) 44.0 (CH), 52.0 (CH_3), 52.3 (CH_3), 76.0 (CH), 116.1 (2 × CH), 121.7 (CH), 126.2 (CH), 128.6 (4 × CH), 129.9 (2 × CH), 141.2 (C), 157.6 (C), 172.6 (2 × C); *m/z* (ESI) 357.1689 ([M+H]$^+$. $C_{21}H_{25}O_5^+$ requires 357.1697).

Dimethyl 2-[phenoxy(*p*-tolyl)methyl]succinate (20s)

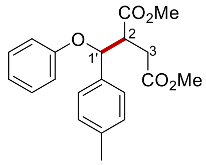

Dimethyl 2-[phenoxy(*p*-tolyl)methyl]succinate (**20s**) was synthesised as described for **20a** using 1-methyl-4-(phenoxymethyl)benzene (**S28**, 0.0595 g, 0.300 mmol), dimethyl fumarate (0.129 g, 0.900 mmol) and blue light. Purification by flash chromatography eluting with 20 to 30% diethyl ether in hexane gave dimethyl 2-[phenoxy(*p*-tolyl)methyl]succinate (**20s**) as a yellow oil and a 1.0:1.1 mixture of diastereoisomers that could not be separated by chromatography (0.0890 g, 93%). ν_{max}/cm^{-1} (neat) 2951, 1732, 1597, 1493, 1435, 1225, 1163, 754; **Data for major diastereoisomer:** ^{1}H-NMR (400 MHz, $CDCl_3$) 2.31 (3H, s, CH_3), 2.57 (1H, dd, *J* = 16.9, 3.9 Hz, 3-*H*H), 3.03 (1H, dd, *J* = 16.9, 10.5 Hz, 3-H*H*), 3.37 (1H, ddd, *J* = 10.5, 5.1, 3.9 Hz, 2-H), 3.60 (3H, s, CH_3), 3.63 (3H, s, CH_3), 5.59 (1H, d, *J* =

5.1 Hz, OCH), 6.76–6.92 (3H, m, 3 × CH), 7.10–7.28 (6H, m, 6 × CH); ^{13}C-NMR (101 MHz, $CDCl_3$) 21.3 (CH_3), 31.1 (CH_2), 49.4 (CH), 51.9 (CH_3), 52.3 (CH_3), 80.3 (CH), 116.0 (2 × CH), 121.5 (CH), 126.3 (2 × CH), 129.4 (2 × CH), 129.5 (2 × CH), 135.4 (C), 137.9 (C), 157.6 (C), 172.0 (C), 172.8 (C); **Data for minor diastereoisomer:** ^{1}H-NMR (400 MHz, $CDCl_3$) 2.31 (3H, s, CH_3), 2.45 (1H, dd, J = 16.7, 4.3 Hz, 3-*H*H), 2.64 (1H, dd, J = 16.7, 10.4 Hz, 3-H*H*), 3.49 (1H, ddd, J = 10.4, 7.6, 4.3 Hz, 2-H), 3.61 (3H, s, CH_3), 3.74 (3H, s, CH_3), 5.36 (1H, d, J = 7.6 Hz, OCH), 6.76–6.92 (3H, m, 3 × CH), 7.10–7.28 (6H, m, 6 × CH); ^{13}C-NMR (101 MHz, $CDCl_3$) 21.3 (CH_3), 32.5 (CH_2), 48.7 (CH), 51.9 (CH_3), 52.3 (CH_3), 79.5 (CH), 116.4 (2 × CH), 121.5 (CH), 126.9 (2 × CH), 129.4 (2 × CH), 129.5 (2 × CH), 134.8 (C), 138.4 (C), 157.8 (C), 172.4 (C), 173.0; *m/z* (ESI) 365.1351 ($[M+Na]^+$. $C_{20}H_{22}O_5Na^+$ requires 365.1359).

Dimethyl 2-{[(4″-methoxyphenyl)thio]methyl}succinate (20t)

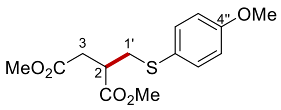

Dimethyl 2-{[(4″-methoxyphenyl)thio]methyl}succinate (**20t**) was synthesised as described for **20a** using 4-methoxy-thioanisole (0.0463 g, 0.300 mmol), dimethyl fumarate (0.129 g, 0.900 mmol) and blue light. Purification by flash chromatography eluting with 20 to 50% diethyl ether in hexane gave dimethyl 2-[(4″-methoxyphenyl)methyl]succinate (**20t**) as a yellow oil (0.0680 g, 76%). ν_{max}/cm^{-1} 2951, 1732, 1591, 1493, 1437, 1244, 1171, 1030, 827; ^{1}H-NMR (500 MHz, $CDCl_3$) 2.73 (1H, dd, J = 16.8, 5.3 Hz, 3-*H*H), 2.79 (1H, dd, J = 16.8, 7.9 Hz, 3-H*H*), 2.91–3.04 (2H, m, 2-H and 1′-*H*H), 3.20 (1H, dd, J = 12.5, 4.8 Hz, 1′-H*H*), 3.64 (3H, s, CH_3), 3.65 (3H, s, CH_3), 3.79 (3H, s, CH_3), 6.85 (2H, d, J = 8.8 Hz, 2 × CH), 7.37 (2H, d, J = 8.8 Hz, 2 × CH); ^{13}C-NMR (126 MHz, $CDCl_3$) 34.6 (CH_2), 37.6 (CH_2), 41.3 (CH), 52.0 (CH_3), 52.2 (CH_3), 55.5 (CH_3), 114.9 (2 × CH), 125.2 (C), 134.2 (2 × CH), 159.6 (C), 172.1 (C), 173.7 (C); *m/z* (ESI) 299.0943 ($[M+H]^+$. $C_{14}H_{19}O_5S^+$ requires 299.0948).

2-(2′-Phenoxy-1′-phenyleth-1′-yl)malononitrile (22a)[24]

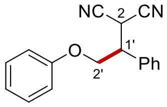

2-(2′-Phenoxy-1′-phenyleth-1′-yl)malononitrile (**6a**) was synthesised as described for **20a** using benzylidenemalononitrile (0.0463 g, 0.300 mmol), anisole (0.0973 g, 0.900 mmol) and blue light. Purification by flash chromatography eluting with 30% diethyl ether in hexane 2-(2′-phenoxy-1′-phenyleth-1′-yl)malononitrile (**22a**) as a brown gum (0.067 g, 85%). Spectroscopic data in agreement with literature data.[24] ^{1}H-NMR (400 MHz, $CDCl_3$) 3.72 (1H, ddd, J = 8.7, 5.7, 4.7 Hz, 1′-H), 4.34–

4.47 (2H, m, 2′-H_2), 4.55 (1H, d, J = 5.7 Hz, 2-H), 6.92–7.00 (2H, m, 2 × CH), 7.01–7.08 (1H, m, CH), 7.30–7.37 (2H, m, 2 × CH), 7.42–7.50 (5H, m, 5 × CH); ^{13}C-NMR (101 MHz, $CDCl_3$) 26.0 (CH), 45.6 (CH), 66.6 (CH_2), 110.8 (C≡N), 111.3 (C≡N), 114.2 (2 × CH), 121.7 (CH), 127.7 (2 × CH), 129.0 (2 × CH), 129.1 (CH), 129.3 (2 × CH), 133.3 (C), 157.0 (C); m/z (ESI): 262.0 $[M]^+$.

2-[2′-Phenoxy-1′-(4″-methylphenyl)eth-1′-yl]malononitrile (22b)

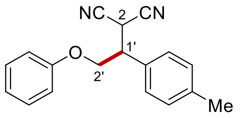

2-[2′-Phenoxy-1′-(4″-methylphenyl)eth-1′-yl]malononitrile (**22b**) was synthesised as described for **20a** using 4-methylbenzylidenemalononitrile (**S13**, 0.0505 g, 0.300 mmol), anisole (0.0973 g, 0.900 mmol) and blue light. Purification by flash chromatography eluting with 30% diethyl ether in hexane gave 2-[2′-phenoxy-1′-(4″-methylphenyl)eth-1′-yl]malononitrile (**22b**) as a yellow oil (0.0820 g, 99%).* ν_{max}/cm^{-1} (neat) 2918, 1599, 1497, 1470, 1238, 1040, 818, 754, 691; ^{1}H-NMR (500 MHz, $CDCl_3$) 2.42 (3H, s, CH_3), 3.63–3.80 (1H, m, 1′-H), 4.33–4.47 (2H, m, 2′-H), 4.55 (1H, d, J = 5.6 Hz, 2-H), 6.98 (2H, d, J = 7.9 Hz, 2 × CH), 7.07 (1H, t, J = 7.4 Hz, CH), 7.29 (2H, d, J = 8.0 Hz, 2 × CH), 7.32–7.34 (4H, m, 4 × CH); ^{13}C-NMR (126 MHz, $CDCl_3$) 21.2 (CH_3), 26.7 (CH), 45.7 (CH), 67.2 (CH_2), 111.5 (C≡N), 111.9 (C≡N), 114.7 (2 × CH), 122.2 (CH), 128.1 (2 × CH), 129.8 (2 × CH), 130.1 (2 × CH), 130.8 (C), 139.6 (C), 157.6 (C); m/z (ESI) 275.1195 ($[M-H]^-$. $C_{18}H_{15}N_2O^+$ requires 275.1190).

*In this series of compounds, no infrared peaks that correspond to the nitrile group stretches were apparent; similar phenomena have been reported previously.[25]

2-[2′-Phenoxy-1′-(4″-fluorophenyl)eth-1′-yl]malononitrile (22c)

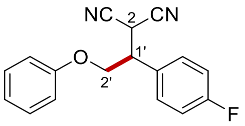

2-[2′-Phenoxy-1′-(4″-fluorophenyl)eth-1′-yl]malononitrile (**22c**) was synthesised as described for **20a** using 2-(4′-fluorobenzylidene)malononitrile (**S14**, 0.0516 g, 0.300 mmol), anisole (0.0973 g, 0.900 mmol) and blue light. Purification by flash chromatography eluting with 40% diethyl ether in hexane gave 2-[2′-phenoxy-1′-(4″-fluorophenyl)eth-1′-yl]malononitrile (**22c**) as a colourless oil (0.0677 g, 86%). ν_{max}/cm^{-1} (neat) 2913, 1599, 1512, 1468, 1231, 1163, 839, 756; ^{1}H-NMR (500 MHz, $CDCl_3$) 3.69 (1H, m, 1′-H), 4.33 – 4.46 (2H, m, 2′-H_2), 4.56 (1H, d, J = 5.8 Hz, 2-H), 6.98 (2H, dd, J = 8.7, 0.9 Hz, 2 × CH), 7.08 (1H, t, J = 7.4 Hz, CH), 7.14–7.24 (2H, m, 2 × CH), 7.32–7.42 (2H, m, 2 × CH),

7.45–7.56 (2H, m, 2 × CH); ^{13}C-NMR (126 MHz, $CDCl_3$) 26.7 (CH), 45.5 (CH), 67.2 (CH_2), 111.3 (C≡N), 111.8 (C≡N), 114.8 (2 × CH), 116.7 ($^2J_{CF}$ = 21.7 Hz, 2 × CH), 122.5 (CH), 129.8 ($^4J_{CF}$ = 3.3 Hz, C), 130.0 (2 × CH), 130.2 ($^3J_{CF}$ = 8.3 Hz, 2 × CH), 157.5 (C), 163.4 ($^1J_{CF}$ = 249.6 Hz, C); *m/z* (ESI) 279.0942 ($[M-H]^-$. $C_{17}H_{12}FN_2O$ requires 279.0939).

2-[2′-Phenoxy-1′-(4″-chlorophenyl)eth-1′-yl]malononitrile (22d)

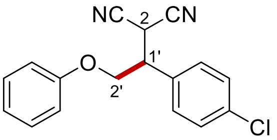

2-[2′-Phenoxy-1′-(4″-chlorophenyl)eth-1′-yl]malononitrile (**22d**) was synthesised as described for **20a** using 2-(4′-chlorobenzylidene)malononitrile (**S15**, 0.0566 g, 0.300 mmol), anisole (0.0973 g, 0.900 mmol) and blue light. Purification by flash chromatography eluting with 40% diethyl ether in hexane gave 2-[2′-phenoxy-1′-(4″-chlorophenyl)eth-1′-yl]malononitrile (**22d**) as a colourless oil (0.0864 g, 97%). ν_{max}/cm^{-1} (neat) 2953, 1732, 1599, 1495, 1435, 1242, 1167, 754, 692; ^{1}H-NMR (400 MHz, $CDCl_3$) 3.70 (1H, app. dt, *J* = 7.7, 5.5 Hz, 1′-H), 4.41–4.31 (2H, m, 2′-H), 4.54 (1H, d, *J* = 5.9 Hz, 2-H), 6.94 (2H, dd, *J* = 8.7, 0.9 Hz, 2 × CH), 7.00–7.10 (1H, m, CH), 7.48–7.29 (6H, m, 6 × CH); ^{13}C-NMR (101 MHz, $CDCl_3$) 26.6 (CH) 45.7 (CH), 67.1 (CH_2), 111.3 (C≡N), 111.8 (C≡N), 114.8 (2 × CH), 122.5 (CH), 129.7 (2 × CH), 129.9 (2 × CH), 130.0 (2 × CH), 132.4 (C), 135.9 (C), 157.5 (C); *m/z* (ESI) 295.0648 ($[M-H]^-$. $C_{17}H_{12}{}^{35}ClN_2O$ requires 295.0644).

2-[2′-Phenoxy-1′-(4″-bromophenyl)eth-1′-yl]malononitrile (22e)

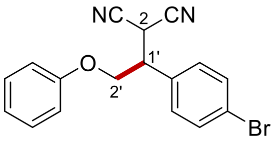

2-[2′-Phenoxy-1′-(4″-bromophenyl)eth-1′-yl]malononitrile (**22e**) was synthesised as described for **20a** using 2-(4′-bromobenzylidene)malononitrile (**S16**, 0.0699 g, 0.300 mmol), anisole (0.0973 g, 0.900 mmol) and blue light. Purification by flash chromatography eluting with 40% diethyl ether in hexane gave 2-[2′-phenoxy-1′-(4″-bromophenyl)eth-1′-yl]malononitrile (**22e**) as a colourless oil (0.0959 g, 94%). ν_{max}/cm^{-1} (neat) 2907, 1597, 1491, 1238, 1074, 1011, 827, 756, 691; ^{1}H-NMR (500 MHz, $CDCl_3$) 3.64–3.73 (1H, m, 1′-H), 4.33–4.40 (2H, m, 2′-H_2), 4.53 (1H, d, *J* = 5.9 Hz, 2-H), 6.94 (2H, dd, *J* = 8.7, 0.9 Hz, 2 × CH), 7.05 (1H, t, *J* = 7.4 Hz, CH), 7.29–7.38 (4H, m, 4 × CH), 7.60 (2H, d, *J* = 8.5 Hz, 2 × CH); ^{13}C-NMR (126 MHz, $CDCl_3$) 26.5 (CH), 45.7 (CH), 67.0 (CH_2), 111.3 (C≡N), 111.7 (C≡N),

114.8 (2 × CH), 122.5 (CH), 124.1 (C), 130.0 (4 × CH), 132.8 (2 × CH), 132.9 (C), 157.5 (C); *m/z* (ESI) 339.0143 ([M−H]$^{-}$. $C_{17}H_{12}{}^{79}BrN_2O^{-}$ requires 339.0138).

2-[2′-Phenoxy-1′-(4″-iodophenyl)eth-1′-yl]malononitrile (22f)

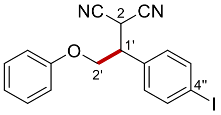

2-[2′-Phenoxy-1′-(4″-iodophenyl)eth-1′-yl]malononitrile (**22f**) was synthesised as described for **20a** using 2-(4′-iodobenzylidene)malononitrile (**S17**, 0.0699 g, 0.300 mmol), anisole (0.0973 g, 0.900 mmol) and blue light. Purification by flash chromatography eluting with 40% diethyl ether in hexane gave 2-[2′-phenoxy-1′-(4″-iodophenyl)eth-1′-yl]malononitrile (**22f**) as a colourless oil (0.110 g, 94%). ν_{max}/cm^{-1} (neat) 2905, 1599, 1589, 1244, 1045, 1007, 820, 758; ^{1}H-NMR (400 MHz, $CDCl_3$) 3.66 (1H, app. dt, *J* = 7.3, 5.7 Hz, 1′-H), 4.33–4.42 (2H, m, 2′-H_2), 4.53 (1H, d, *J* = 5.9 Hz, 2-H), 6.92–6.98 (2H, m, 2 × CH), 7.01–7.10 (1H, m, CH), 7.21 (2H, d, *J* = 8.4 Hz, 2 × CH), 7.29–7.39 (2H, m, 2 × CH), 7.80 (2H, d, *J* = 8.4 Hz, 2 × CH); ^{13}C-NMR (101 MHz, $CDCl_3$) 25.8 (CH), 45.2 (CH), 66.3 (CH_2), 95.1 (C), 110.6 (C≡N), 111.1 (C≡N), 114.2 (2 × CH), 121.9 (CH), 129.3 (2 × CH), 129.5 (2 × CH), 132.9 (C), 138.1 (2 × CH), 156.9 (C); *m/z* (ESI) 387.0007 ([M−H]$^{-}$. $C_{17}H_{12}ON_2I$ requires 387.0000).

2-[2′-Phenoxy-1′-(4″-methoxyphenyl)eth-1′-yl]malononitrile (22g)

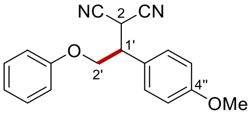

2-[2′-Phenoxy-1′-(4″-methoxyphenyl)eth-1′-yl]malononitrile (**22g**) was synthesised as described for **20a** using 2-(4′-methoxybenzylidene)malononitrile (**S18**, 0.0553 g, 0.300 mmol), anisole (0.0973 g, 0.900 mmol) and blue light. Purification by flash chromatography eluting with 40% diethyl ether in hexane gave 2-[2′-phenoxy-1′-(4″-methoxyphenyl)eth-1′-yl]malononitrile (**22a**) as a yellow oil (0.0759 g, 87%). ν_{max}/cm^{-1} (neat) 2909, 1599, 1516, 1497, 1242, 1180, 1032, 883, 756, 691; ^{1}H-NMR (400 MHz, $CDCl_3$) 3.67 (1H, app. dt, *J* = 8.9, 5.1 Hz, 1′-H), 3.83 (3H, s, CH_3), 4.44–4.29 (2H, m, 2′-H), 4.51 (1H, d, *J* = 5.6 Hz, 2-H), 7.00–6.91 (4H, m, 4 × CH), 7.04 (1H, tt, *J* = 7.4, 0.9 Hz, CH), 7.33 (2H, dd, *J* = 8.7, 7.4 Hz, 2 × CH), 7.38 (2H, d, *J* = 8.7 Hz, 2 × CH); ^{13}C-NMR (101 MHz, $CDCl_3$) 26.9 (CH), 45.6 (CH), 55.5 (CH_3), 67.4 (CH_2), 111.6 (C≡N), 112.1 (C≡N), 114.8 (2 × CH), 114.9 (2 × CH), 122.3 (CH), 125.8 (C), 129.6 (2 × CH), 129.9 (2 × CH), 157.7 (C), 160.6 (C); *m/z* (ESI) 291.1144 ([M−H]$^{-}$. $C_{18}H_{15}N_2O_2$ requires 291.1139).

2-[2′-Phenoxy-1′-(4″-nitrophenyl)eth-1′-yl]malononitrile (22h)

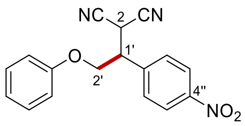

2-[2′-Phenoxy-1′-(4″-nitrophenyl)eth-1′-yl]malononitrile (**22h**) was synthesised as described for **20a** using 2-(4′-nitrobenzylidene)malononitrile (**S19**, 0.0600 g, 0.300 mmol), anisole (0.0973 g, 0.900 mmol) and blue light. Purification by flash chromatography eluting with 40% diethyl ether in hexane gave 2-[2′-phenoxy-1′-(4″-nitrophenyl)eth-1′-yl]malononitrile (**22h**) as a colourless oil (0.0360 g, 39%). ν_{max}/cm^{-1} (neat) 2913, 1599, 1522, 1348, 1236, 854, 756, 692; ^{1}H-NMR (400 MHz, $CDCl_3$) 3.84 (1H, app. q, J = 6.2 Hz, 1′-H), 4.43 (2H, d, J = 6.2 Hz, 2′-H_2), 4.60 (1H, d, J = 6.2 Hz, 2-H), 6.95 (2H, dd, J = 8.7, 0.9 Hz, 2 × CH), 7.03–7.11 (1H, m, CH), 7.30–7.40 (2H, m, 2 × CH), 7.68 (2H, d, J = 8.8 Hz, 2 × CH), 8.33 (2H, d, J = 8.8 Hz, 2 × CH); ^{13}C-NMR (101 MHz, $CDCl_3$) 26.2 (CH), 45.9 (CH), 66.7 (CH_2), 110.9 (C≡N), 111.4 (C≡N), 114.9 (2 × CH), 122.8 (CH), 124.7 (2 × CH), 129.7 (2 × CH), 130.1 (2 × CH), 140.9 (C), 148.8 (C), 157.3 (C); *m/z* (ESI) 306.0886 ([M−H]$^-$. $C_{17}H_{12}N_3O_3^-$ requires 306.0884).

2-[2′-(3″-Methylphenyl)-1′-phenyleth-1′-yl]malononitrile (22i)

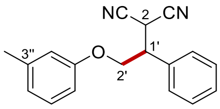

2-[2′-(3″-Methylphenyl)-1′-phenyleth-1′-yl]malononitrile (**22i**) was synthesised as described for **20a** using benzylidenemalononitrile (0.0463 g, 0.300 mmol), 3-methylanisole (0.110 g, 0.900 mmol) and blue light. Purification by flash chromatography eluting with 0 to 20% ethyl acetate in hexane gave 2-[2′-(3″-methylphenyl)-1′-phenyleth-1′-yl]malononitrile (**22i**) as a brown gum (0.0640 g, 77%). ν_{max}/cm^{-1} (neat) 3059, 3034, 2914, 1584, 1489; ^{1}H-NMR (400 MHz, $CDCl_3$) 2.35 (3H, s, CH_3), 3.70 (1H, ddd, J = 8.8, 5.7, 4.7 Hz, 1′-H), 4.32–4.43 (2H, m, 2′-H_2), 4.56 (1H, d, J = 5.7 Hz, 2-H), 6.72–6.79 (2H, m, 2 × CH), 6.82–6.88 (1H, m, CH), 7.20 (1H, t, J = 7.8 Hz, CH), 7.42–7.49 (5H, m, 5 × CH); ^{13}C-NMR (101 MHz, $CDCl_3$) 21.0 (CH_3), 26.0 (CH), 45.5 (CH), 66.4 (CH_2), 110.9 (C≡N), 111.0 (CH), 111.4 (C≡N), 114.9 (CH), 122.5 (CH), 127.7 (CH), 129.0 (4 × CH), 129.1 (CH), 133.3 (C), 139.5 (C), 157.0 (C); *m/z* (ESI) 277.1327 ([M+H]$^+$. $C_{18}H_{16}N_2O^+$ requires 277.1335).

2-[2′-(3″-Methylphenyl)-1′-(4‴-methylphenyl)eth-1′-yl]malononitrile (22j)

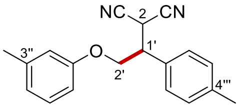

2-[2′-(3″-Methylphenyl)-1′-(4‴-methylphenyl)eth-1′-yl]malononitrile (**22j**) was synthesised as described for **20a** using 4-methylbenzylidenemalonodinitrile (**S13**, 0.0505 g, 0.300 mmol), 3-methylanisole (0.110 g, 0.900 mmol) and blue light. Purification by flash chromatography eluting with 0 to 30% ethyl acetate in hexane gave 2-[2′-(3″-methylphenyl)-1′-(4‴-methylphenyl)eth-1′-yl]malononitrile **22j** as a brown gum (0.0590 g, 68%). ν_{max}/cm^{-1} (neat) 3030, 3011, 2916, 1603, 1585, 1489; ^{1}H-NMR (400 MHz, $CDCl_3$) 2.35 (3H, s, CH_3), 2.39 (3H, s, CH_3), 3.62–3.72 (1H, m, 1′-H), 4.29–4.42 (2H, m, 2′-H_2), 4.52 (1H, d, *J* = 5.6 Hz, 2-H), 6.72–6.80 (2H, m, 2 × CH), 6.85 (1H, d, *J* = 7.5 Hz, CH), 7.20 (1H, t, *J* = 7.8 Hz, CH), 7.24–7.28 (2H, m, 2 × CH), 7.32–7.37 (2H, m, 2 × CH); ^{13}C-NMR (101 MHz, $CDCl_3$) 20.7 (CH_3), 20.9 (CH_3), 26.1 (CH), 45.3 (CH), 66.6 (CH_2), 110.9 (C≡N), 110.0 (CH), 111.4 (C≡N), 115.0 (CH), 122.4 (CH), 127.6 (2 × CH), 129.0 (CH), 129.6 (2 × CH), 130.3 (C), 139.0 (C), 139.4 (C), 157.1 (C); *m/z* (ESI) 291.1483 ([M+H]$^+$. $C_{19}H_{19}N_2O^+$ requires 291.1492).

2-[2′-(3″-Methylphenyl)-1′-(4‴-chlorophenyl)eth-1′-yl]malononitrile (22k)

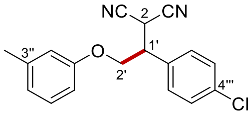

2-[2′-(3″-Methylphenyl)-1′-(4‴-chlorophenyl)eth-1′-yl]malononitrile (**22k**) was synthesised as described for **20a** using 4-chlorobenzylidenemalonodinitrile (**S15**, 0.0566 g, 0.300 mmol), 3-methylanisole (0.110 g, 0.900 mmol) and blue light. Purification by flash chromatography eluting with 0 to 30% ethyl acetate in hexane 2-[2′-(3″-methylphenyl)-1′-(4‴-chlorophenyl)eth-1′-yl]malononitrile (**22k**) as a brown gum (0.0680 g, 73%). ν_{max}/cm^{-1} (neat) 2914, 1585, 1489; ^{1}H-NMR (400 MHz, $CDCl_3$) 2.35 (3H, s, CH_3), 3.63–3.73 (1H, m, 1′-H), 4.31–4.40 (2H, m, 2′-H_2), 4.52 (1H, d, *J* = 5.9 Hz, 2-H), 6.71–6.79 (2H, m, 2 × CH), 6.86 (1H, d, *J* = 7.5 Hz, CH), 7.21 (2H, t, *J* = 7.8 Hz, 2 × CH), 7.37–7.48 (4H, m, 4 × CH); ^{13}C-NMR (101 MHz, $CDCl_3$) 21.0 (CH_3), 25.9 (CH), 45.0 (CH), 66.3 (CH_2), 110.7 (C≡N), 111.1 (C≡N and CH), 115.0 (CH), 122.7 (CH), 129.0 (CH), 129.1 (2 × CH), 129.2 (2 × CH), 131.8 (C), 135.2 (C), 139.5 (C), 156.9 (C); *m/z* (ESI) 309.0804 ([M−H]$^-$. $C_{18}H_{14}{}^{35}ClN_2O^-$ requires 309.0800).

2-[2′-(4″-Fluorophenyl)-1′-phenyleth-1′-yl]malononitrile (22l)

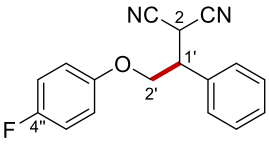

2-[2′-(4″-Fluorophenyl)-1′-phenyleth-1′-yl]malononitrile (**22l**) was synthesised as described for **20a** using benzylidenemalononitrile (0.0553 g, 0.300 mmol), 4-fluoroanisole (0.102 mL, 0.900 mmol) and blue light. Purification by flash chromatography eluting with 0 to 40% ethyl acetate in hexane 2-[2′-(4″-fluorophenyl)-1′-phenyleth-1′-yl]malononitrile (**22l**) as a brown gum (0.0710 g, 84%). ν_{max}/cm^{-1} (neat): 3064, 3034, 2914, 1601, 1504, 1470, 1202; ^{1}H-NMR (400 MHz, $CDCl_3$) 3.71 (1H, ddd, J = 8.7, 5.7, 4.7 Hz, 1′-H), 4.29–4.42 (2H, m, 2′-H_2), 4.53 (1H, d, J = 5.7 Hz, 2-H), 6.87–6.94 (2H, m, 2 × CH), 6.97–7.06 (2H, m, 2 × CH), 7.41–7.52 (5H, m, 5 × CH); ^{13}C-NMR (101 MHz, $CDCl_3$) 26.0 (CH), 45.5 (CH), 67.6 (CH_2), 110.8 (C≡N), 111.2 (C≡N), 115.5 (d, $^{3}J_{CF}$ = 8.1 Hz, 2 × CH), 115.7 (d, $^{2}J_{CF}$ = 23.3 Hz, 2 × CH), 127.6 (2 × CH), 129.0 (2 × CH), 129.1 (CH), 133.2 (C). 153.2 (C), 157.6 (d, $^{1}J_{CF}$ = 241.3 Hz, C); m/z (ESI) 281.1081 ($[M+H]^+$. $C_{17}H_{13}FN_2O^+$ requires 281.1085).

2-[2′-(4″-Fluorophenyl)-1′-(4‴-methylphenyl)eth-1′-yl]malononitrile (22m)

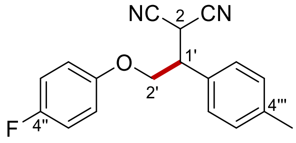

2-[2′-(4″-Fluorophenyl)-1′-(4‴-methylphenyl)eth-1′-yl]malononitrile (**22m**) was synthesised as described for **20a** using 4-methylbenzylidenemalononitrile (**S14**, 0.0505 g, 0.300 mmol), 4-fluoroanisole (0.102 mL, 0.900 mmol) and blue light. Purification by flash chromatography eluting with 0 to 30% ethyl acetate in hexane gave 2-[2′-(4″-fluorophenyl)-1′-(4‴-methylphenyl)eth-1′-yl]malononitrile (**22m**) as a brown gum (0.0750 g, 85%). ν_{max}/cm^{-1} (neat): 3051, 3028, 2914, 1603, 1504, 1471, 1202; ^{1}H-NMR (400 MHz, $CDCl_3$) 2.38 (3H, s, CH_3), 3.62–3.71 (1H, m, 1′-H), 4.27–4.40 (2H, m, 2′-H_2), 4.49 (1H, d, J = 5.7 Hz, 2-H), 6.86–6.93 (2H, m, 2 × CH), 6.97–7.05 (2H, m, 2 × CH), 7.25 (2H, d, J = 6.8 Hz, 2 × CH), 7.32 (2H, d, J = 8.2 Hz, 2 × CH); ^{13}C-NMR (101 MHz, $CDCl_3$) 20.7 (CH_3), 26.1 (CH), 45.2 (CH), 67.6 (CH_2), 110.9 (C≡N), 111.3 (C≡N), 115.5 (d, $^{3}J_{CF}$ = 8.0 Hz, 2 × CH), 115.7 (d, $^{2}J_{CF}$ = 23.3 Hz, 2 × CH), 127.5 (2 × CH), 129.6 (2 × CH), 130.2 (C), 139.1 (C), 153.2 (C), 157.5 (d, $^{1}J_{CF}$ = 241.0 Hz, C); m/z (ESI) 293.1098 ($[M-H]^-$. $C_{18}H_{14}FN_2O^-$ requires 293.1096).

2-[2′-(4″-Fluorophenyl)-1′-(4‴-chlorophenyl)eth-1′-yl]malononitrile (22n)

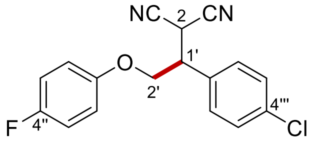

2-[2′-(4″-Fluorophenyl)-1′-(4‴-chlorophenyl)eth-1′-yl]malononitrile (**22n**) was synthesised as described for **20a** using 4-chlorobenzylidenemalononitrile (**S15**, 0.0566 g, 0.300 mmol), 4-fluoroanisole (0.102 mL, 0.900 mmol) and blue light. Purification by flash chromatography eluting with 0 to 30% ethyl acetate in hexane gave 2-[2′-(4″-fluorophenyl)-1′-(4‴-chlorophenyl)eth-1′-yl]malononitrile (**22n**) as a brown gum (0.0760 g, 80%). ν_{max}/cm^{-1} (neat): 2914, 1601, 1504, 1495, 1202; ^{1}H-NMR (400 MHz, $CDCl_3$) 3.64–3.73 (1H, m, 1′-H), 4.28–4.38 (2H, m, 2′-H_2), 4.50 (1H, d, J = 5.9 Hz, 2-H), 6.86–6.93 (2H, m, 2 × CH), 6.97–7.06 (2H, m, 2 × CH), 7.37–7.47 (4H, m, 4 × CH); ^{13}C-NMR (101 MHz, $CDCl_3$) 25.9 (CH), 45.0 (CH), 67.3 (CH_2), 110.0 (C≡N), 111.3 (C≡N), 115.6 (d, $^3J_{CF}$ = 8.0 Hz, 2 × CH), 115.6 (d, $^2J_{CF}$ = 23.4 Hz, 2 × CH), 129.0 (2 × CH), 129.3 (2 × CH), 131.7 (C), 135.3 (C), 153.0 (C), 157.6 (d, $^1J_{CF}$ = 241.7 Hz, C); *m/z* (ESI) 313.0553 ([M−H]$^-$. $C_{17}H_{11}{}^{35}ClFN_2O^-$ requires 313.0549).

2-[2′-(4″-Chlorophenyl)-1′-phenyleth-1′-yl]malononitrile (22o)[25]

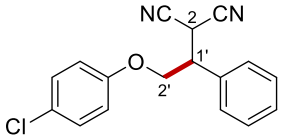

2-[2′-(4″-Chlorophenyl)-1′-phenyleth-1′-yl]malononitrile (**22o**) was synthesised as described for **20a** using 4-chloroanisole (0.128 g, 0.900 mmol), benzylidenemalononitrile (0.0463 g, 0.300 mmol) and blue light. Purification by flash chromatography eluting with 40% diethyl ether in hexane gave 2-[2′-(4″-chlorophenyl)-1′-phenyleth-1′-yl]malononitrile (**22o**) as a yellow oil (0.0872 g, 99%). Spectroscopic data were in agreement with the literature data.[25] ^{1}H-NMR (400 MHz, $CDCl_3$) 3.71 (1H, ddd, J = 8.8, 5.7, 4.7 Hz, 1′-H), 4.31 – 4.43 (2H, m, 2′-H_2), 4.51 (1H, d, J = 5.7 Hz, 2-H), 6.89 (2H, d, J = 9.0 Hz, 2 × CH), 7.28 (2H, d, J = 9.0 Hz, 2 × CH), 7.40–7.52 (5H, m, 5 × CH); ^{13}C-NMR (101 MHz, $CDCl_3$) 26.6 (CH), 46.0 (CH), 67.6 (CH_2), 111.4 (C≡N), 111.8 (C≡N), 116.1 (2 × CH), 127.3 (C), 128.2 (2 × CH), 129.6 (2 × CH), 129.8 (3 × CH), 133.7 (C), 156.2 (C); *m/z* (EI) 296 (M^+, 10), 169 (30), 130 (40), 104 (100),78 (40).

2-[2′-(4″-Chlorophenyl)-1′-(4‴-bromophenyl)eth-1′-yl]malononitrile (22p)

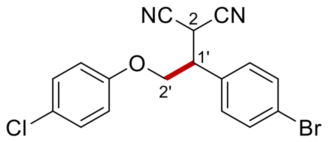

2-[2′-(4″-Chlorophenyl)-1′-(4‴-bromophenyl)eth-1′-yl]malononitrile (**22p**) was synthesised as described for **20a** using 4-bromobenzylidenemalononitrile (**S16**, 0.0699 g, 0.300 mmol), 4-chloroanisole (0.128 g, 0.900 mmol) and blue light. Purification by flash chromatography eluting with 50% diethyl ether in hexane gave 2-[2′-(4″-chlorophenyl)-1′-(4‴-bromophenyl)eth-1′-yl]malononitrile (**22p**) as a yellow oil (0.0970 g, 95%). ν_{max}/cm^{-1} (neat): 2911, 1595, 1489, 1236, 1009, 824; ^{1}H-NMR (400 MHz, $CDCl_3$) 3.68 (1H, app. dt, *J* = 7.6, 5.6 Hz, 1′-H), 4.29 – 4.39 (2H, m, 2′-H), 4.48 (1H, d, *J* = 5.9 Hz, 2-H), 6.88 (2H, d, *J* = 9.0 Hz, 2 × CH), 7.28 (2H, d, *J* = 9.0 Hz, 2 × CH), 7.32 (2H, d, *J* = 8.5 Hz, 2 × CH), 7.59 (2H, d, *J* = 8.5 Hz, 2 × CH); ^{13}C-NMR (101 MHz, $CDCl_3$) 26.4 (CH), 45.6 (CH), 67.4 (CH_2), 111.2 (C≡N), 111.6 (C≡N), 116.1 (2 × CH), 124.1 (C), 127.5 (C), 129.9 (4 × CH), 132.7 (C), 132.9 (2 × CH), 156.1 (C); *m/z* (ESI) 372.9757 ($[M-H]^-$. $C_{17}H_{11}{}^{79}Br^{35}ClN_2O^-$ requires 372.9749).

2-[2′-(4″-Chlorophenyl)-1′-(4‴-iodophenyl)eth-1′-yl]malononitrile (22q)

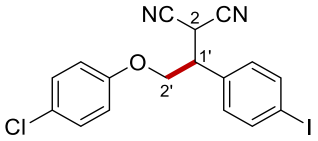

2-[2′-(4″-Chlorophenyl)-1′-(4‴-iodophenyl)eth-1′-yl]malononitrile (**22q**) was synthesised as described for **20a** using 2-(4′-iodobenzylidene)malononitrile (**S17**, 0.0840 g, 0.300 mmol), 4-chloroanisole (0.128 g, 0.900 mmol) and blue light. Purification by flash chromatography eluting with 50 to 60% diethyl ether in hexane gave 2-[2′-(4″-chlorophenyl)-1′-(4‴-iodophenyl)eth-1′-yl]malononitrile (**22q**) as a colourless oil (0.131 g, 100%). ν_{max}/cm^{-1} (neat) 2911, 1595, 1489, 1283, 1238, 1007, 824; ^{1}H-NMR (400 MHz, $CDCl_3$) 3.61–3.72 (1H, m, 1′-H), 4.28–4.38 (2H, m, 2′-H_2), 4.48 (1H, d, *J* = 5.9 Hz, 2-H), 6.87 (2H, d, *J* = 9.0 Hz, 2 × CH), 7.19 (2H, d, *J* = 8.4 Hz, 2 × CH), 7.28 (2H, d, *J* = 9.0 Hz, 2 × CH), 7.80 (2H, d, *J* = 8.4 Hz, 2 × CH); ^{13}C-NMR (101 MHz, $CDCl_3$) 26.4 (CH), 45.7 (CH), 67.4 (CH_2), 95.9 (C), 111.2 (C≡N), 111.6 (C≡N), 116.1 (2 × CH), 127.5 (C), 129.9 (2 × CH), 130.0 (2 × CH), 133.4 (C), 138.8 (2 × CH), 156.1 (C); *m/z* (ESI) 420.9619 ($[M-H]^-$. $C_{17}H_{11}{}^{35}ClIN_2O^-$ requires 420.9619).

2-[2′-(4″-Chlorophenyl)-1′-(4‴-methoxyphenyl)eth-1′-yl]malononitrile (22r)[25]

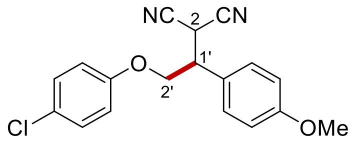

2-[2′-(4″-Chlorophenyl)-1′-(4‴-methoxyphenyl)eth-1′-yl]malononitrile (**22r**) was synthesised as described for **20a** using 4-methoxybenzylidenemalononitrile (**S18**, 0.0553 g, 0.300 mmol), 4-chloroanisole (0.128 g, 0.900 mmol) and blue light. Purification by flash chromatography eluting with 40% diethyl ether in hexane gave 2-[2′-(4″-chlorophenyl)-1′-(4‴-methoxyphenyl)eth-1′-yl]malononitrile (**22r**) as a yellow oil (0.093 g, 96%). Spectroscopic data were in agreement with the literature data.[25] ^{1}H-NMR (400 MHz, $CDCl_3$), 3.63 – 3.71 (1H, m, 1′-H), 4.26 – 4.40 (2H, m, 2′-H), 4.48 (1H, d, J = 5.6 Hz, 2-H), 6.88 (2H, d, J = 9.0 Hz, 2 × CH), 6.97 (2H, d, J = 8.8 Hz, 2 × CH), 7.28 (2H, d, J = 9.0 Hz, 2 × CH), 7.36 (2H, d, J = 8.8 Hz, 2 × CH); ^{13}C-NMR (101 MHz, $CDCl_3$) 26.9 (CH), 45.3 (CH), 55.5 (CH_3), 67.7 (CH_2), 111.5 (C≡N), 112.0 (C≡N), 114.9 (2 × CH), 116.1 (2 × CH), 125.5 (C), 127.2 (C), 129.5 (2 × CH), 129.8 (2 × CH), 156.2 (C), 160.5 (C); m/z (EI) 326 (M^+, 10), 199 (50), 134 (100), 99 (30).

2-(2′-Methyl-2′-phenoxy-1′-phenylprop-2′-yl)malononitrile (22s)

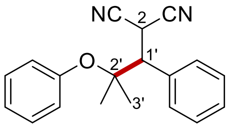

2-(2′-Methyl-2′-phenoxy-1′-phenylprop-2′-yl)malononitrile (**22s**) was synthesised as described for **20a** using benzylidenemalononitrile (0.0470 g, 0.300 mmol), isopropyl phenyl ether (**S25**, 0.123 g, 0.900 mmol) and blue light. Purification by flash chromatography eluting with 0 to 40% diethyl ether in hexane gave 2-(2′-methyl-2′-phenoxy-1′-phenylprop-2′-yl)malononitrile **22s** as a brown gum (0.0680 g, 78%). ν_{max}/cm^{-1} (neat): 3059, 3036, 2990, 2909, 1591, 1585, 1487; ^{1}H-NMR (400 MHz, $CDCl_3$) 1.26 (3H, s, CH_3), 1.40 (3H, s, CH_3), 3.42 (1H, d, J = 6.0 Hz, 1′-H), 4.81 (1H, d, J = 6.0 Hz, 2-H), 7.00–7.07 (2H, m, 2 × CH), 7.16 (1H, tt, J = 7.4, 1.2 Hz, CH), 7.28–7.35 (2H, m, 2 × CH), 7.41–7.48 (3H, m, 3 × CH), 7.51–7.59 (2H, m, 2 × CH); ^{13}C-NMR (101 MHz, $CDCl_3$) 23.7 (CH_3), 24.2 (CH_3), 26.5 (CH), 56.7 (CH), 80.6 (C), 112.6 (C≡N), 112.9 (C≡N), 123.8 (2 × CH), 124.2 (CH), 128.6 (4 × CH), 128.8 (2 × CH), 129.1 (CH), 134.7 (C), 152.8 (C); m/z (ESI) 291.1485 ($[M+H]^+$. $C_{19}H_{19}N_2O^+$requires 291.1492.

2-[2′-Methyl-2′-phenoxy-1′-(4″-iodophenyl)prop-2′-yl]malononitrile (22t)

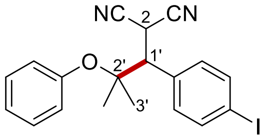

2-[2′-Methyl-2′-phenoxy-1′-(4″-iodophenyl)prop-2′-yl]malononitrile (**22t**) was synthesised as described for **20a** using 4-iodobenzylidenemalononitrile (**S17**, 0.0840 g, 0.300 mmol), isopropyl phenyl ether (**S25**, 0.123 g, 0.900 mmol) and blue light. Purification by flash chromatography eluting with 0 to 10% ethyl acetate in toluene gave 2-[2′-methyl-2′-phenoxy-1′-(4″-iodophenyl)prop-2′-yl]malononitrile (**22t**) as a brown gum (0.0840 g, 67%). ν_{max}/cm^{-1} (neat): 3059, 3036, 2980, 2914, 1591, 1485; ^{1}H-NMR (400 MHz, $CDCl_3$) 1.22 (3H, s, CH_3), 1.41 (3H, s, CH_3), 3.34 (1H, d, *J* = 5.9 Hz, 1′-H), 4.76 (1H, d, *J* = 5.9 Hz, 2-H), 6.98–7.03 (2H, m, 2 × CH), 7.14–7.20 (1H, m, CH), 7.29–7.35 (4H, m, 4 × CH), 7.76–7.82 (2H, m, 2 × CH); ^{13}C-NMR (101 MHz, $CDCl_3$) 24.0 (2 × CH_3), 26.3 (CH), 56.2 (CH), 80.3 (C), 94.8 (C), 112.3 (C≡N), 112.7 (C≡N), 123.7 (2 × CH), 124.3 (CH), 128.9 (2 × CH), 131.0 (2 × CH), 134.3 (2 × CH), 137.7 (C), 152.6 (C); *m/z* (ESI) 415.0312 ($[M{-}H]^-$. $C_{19}H_{16}N_2OI^-$ requires 415.0312).

2-[(2′-Methyl-2′-phenoxy-1′-(4″-methoxyphenyl)prop-2′-yl]malononitrile (22u)

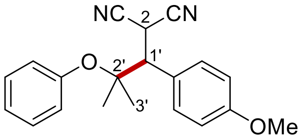

2-[(2′-Methyl-2′-phenoxy-1′-(4″-methoxyphenyl)prop-2′-yl]malononitrile (**22u**) was synthesised as described for **20a** using 4-methoxybenzylidenemalononitrile (**S18**, 0.0553 g, 0.300 mmol), isopropyl phenyl ether (**S25**, 0.123 g, 0.900 mmol) and blue light. Purification by flash chromatography eluting with 0 to 10% ethyl acetate in toluene gave 2-[(2′-methyl-2′-phenoxy-1′-(4″-methoxyphenyl)prop-2′-yl]malononitrile (**22u**) as a brown gum (0.0730 g, 76%). ν_{max}/cm^{-1} (neat): 2980, 2913, 1611, 1591, 1514, 1487; ^{1}H-NMR (400 MHz, $CDCl_3$) 1.28 (3H, s, CH_3), 1.40 (3H, s, CH_3), 3.40 (1H, d, *J* = 5.8 Hz, 1′-H), 3.87 (3H, s, CH_3), 4.80 (1H, d, *J* = 5.8 Hz, 2-H), 6.94–7.07 (4H, m, 4 × CH), 7.15–7.22 (1H, m, CH), 7.30–7.37 (2H, m, 2 × CH), 7.46–7.54 (2H, m, 2 × CH); ^{13}C-NMR (101 MHz, $CDCl_3$) 23.6 (CH_3), 24.3 (CH_3), 26.4 (CH), 54.8 (CH_3), 56.0 (CH), 80.8 (C), 112.7 (C≡N), 113.0 (C≡N), 113.9 (2 × CH), 123.8 (2 × CH), 124.1 (CH), 126.6 (C), 128.8 (2 × CH), 130.3 (2 × CH), 152.9 (C), 159.6 (C); *m/z* (ESI) 321.1593 ($[M+H]^+$. $C_{20}H_{21}N_2O_2^+$ requires 321.1598).

Tetraethyl 3-(phenoxy)propane-1,1-bisphosphonate (23)

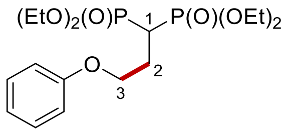

Tetraethyl 3-(phenoxy)propane-1,1-bisphosphonate (**23**) was synthesised as described for **20a** using tetraethyl ethene-1,1-diylbis(phosphonate) (**S12**) (0.0901 g, 0.300 mmol), anisole (0.0973 g, 0.900 mmol) and blue light. Purification by flash chromatography eluting with 20% diethyl ether in hexane gave tetraethyl 3-(phenoxy)propane-1,1-bisphosphonate (**23**) as a colourless oil (0.0921 g, 78%). ν_{max}/cm^{-1} (neat) 2980, 1599, 1240, 1016, 966, 754, 692; ^{1}H-NMR (400 MHz, $CDCl_3$) 1.33 (12H, td, J = 7.1, 2.2 Hz, 4 × CH_3), 2.30–2.53 (2H, m, 2-H_2), 2.71 (1H, tt, J = 23.8, 6.3 Hz, 1-H), 4.06–4.34 (10H, m, 4 × CH_2 and 3-H_2), 6.86–6.98 (3H, m, 3 × CH), 7.24–7.31 (2H, m, 2 × CH); ^{13}C-NMR (101 MHz, $CDCl_3$) 16.4 (d, $^{3}J_{CP}$ = 6.0 Hz, 4 x CH_3), 25.6 (t, $^{2}J_{CP}$ = 4.7 Hz, CH_2), 32.9 (t, $^{1}J_{CP}$ = 134.1 Hz, CH), 62.8 (dd, $^{2}J_{CP}$ = 22.9 and $^{4}J_{CP}$ = 6.6 Hz, 4 x CH_2), 65.5 (t, $^{3}J_{CP}$ = 7.3 Hz, CH_2), 114.6 (2 × CH), 120.9 (CH), 129.6 (2 × CH), 158.7 (C); *m/z* (ESI) 409.1528 ([M+H]$^+$. $C_{17}H_{31}O_7P_2^+$ requires 409.1540).

2-(2′-Phenoxyethyl)-1,3-diphenyl-1,3-dione (24)

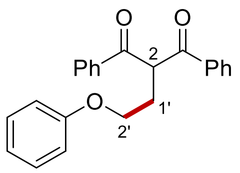

2-(2′-Phenoxyethyl)-1,3-diphenyl-1,3-dione (**24**) was synthesised as described for **20a** using 2-methylene-1,3-diphenylpropane-1,3-dione (**S11**) (0.0709 g, 0.300 mmol), anisole (0.0973 g, 0.900 mmol) and blue light (456 nm). Purification by flash chromatography eluting with 20% diethyl ether in hexane gave 2-(2′-phenoxyethyl)-1,3-diphenyl-1,3-dione (**24**) as a colourless oil (0.0562 g, 73%). ν_{max}/cm^{-1} (neat) 3061, 1693, 1672, 1597, 1497, 1242, 989, 754, 691; ^{1}H-NMR (400 MHz, $CDCl_3$) 2.61 (2H, q, J = 6.0 Hz, 1′-H_2), 4.11 (2H, t, J = 6.0 Hz, 2′-H_2), 5.68 (1H, t, J = 6.0 Hz, 2-H), 6.88 (2H, dd, J = 8.7, 1.0 Hz, 2 × CH), 6.95 (1H, t, J = 7.2, 1.0 Hz, CH), 7.23–7.32 (2H, m, 2 × CH), 7.43 (4H, t, J = 8.0 Hz, 4 × CH), 7.56 (2H, tt, J = 8.0, 1.3 Hz, 2 × CH), 8.00 (4H, dd, J = 8.0, 1.3 Hz, 4 × CH); ^{13}C-NMR (101 MHz, $CDCl_3$) 29.2 (CH_2), 52.8 (CH), 66.5 (CH_2), 114.6 (2 × CH), 121.2 (CH), 128.8 (4 × CH), 129.0 (4 × CH), 129.7 (2 × CH), 133.7 (2 × CH), 136.0 (C), 158.6 (C), 196.2 (2 × C); *m/z* (ESI) 345.1479 ([M+H]$^+$. $C_{23}H_{21}O_3^+$ requires 345.1485).

Diethyl 2-(1′-phenoxypropan-2′-yl)malonate (30)[26]

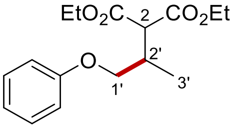

Diethyl 2-(1′-phenoxypropan-2′-yl)malonate (**30**) was synthesised as described for **20a** using diethyl ethylidenemalonate (0.0559 g, 0.300 mmol), anisole (0.0973 g, 0.900 mmol) with the exception that the reaction was irradiated with purple light. Purification by flash chromatography eluting with 20% diethyl ether in hexane gave diethyl 2-(1′-phenoxypropan-2′-yl)malonate (**30**) as a colourless oil (0.0541 g, 61%). Spectroscopic data were in agreement with the literature data.[26] ^{1}H-NMR (400 MHz, $CDCl_3$) 1.16 (3H, d, J = 7.0 Hz, 3′-H_3), 1.22 (3H, t, J = 7.1 Hz, CH_3), 1.26 (3H, t, J = 7.1 Hz, CH_3), 2.68 ‒ 2.82 (1H, m, 2′-H), 3.59 (1H, d, J = 7.7 Hz, 2-H), 3.95 (1H, J = 17.3, 5.6 Hz, 1′-*H*H), 3.98 (1H, J = 17.3, 5.7 Hz, 1′-H*H*), 4.11–4.25 (4H, m, 2 × CH_2), 6.86–6.91 (2H, m, 2 × CH), 6.93 (1H, tt, J = 7.6, 1.0 Hz, CH), 7.22–7.32 (2H, m, 2 × CH); ^{13}C-NMR (101 MHz, $CDCl_3$) 14.1 (CH_3), 14.2 (CH_3), 15.0 (CH_3), 33.6 (CH), 54.2 (CH), 61.4 (CH_2), 61.5 (CH_2), 70.2 (CH_2), 114.7 (2 × CH), 120.9 (CH), 129.5 (2 × CH), 158.9 (C), 168.7 (C), 168.8 (C); *m/z* (EI) 294 (20 (M^+), 242 (80), 201 (60), 175 (70), 145 (80), 134 (85), 109 (80), 99 (90), 83 (100), 55 (90).

4-(3′-Methyl-3′-phenoxybutyl)pyridine (31a)

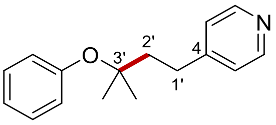

4-(3′-Methyl-3′-phenoxybutyl)pyridine (**31a**) was synthesised as described for **20a** using 4-vinylpyridine (0.0315 g, 0.300 mmol), isopropyl phenyl ether (**S25**, 0.123 g, 0.900 mmol) with the exception that the reaction was irradiated with purple light. Purification by flash chromatography eluting with 70 to 80% diethyl ether in dichloromethane gave 4-(3′-methyl-3′-phenoxybutyl)pyridine (**31a**) as a yellow oil (0.0461 g, 64%). ν_{max}/cm^{-1} (neat) 2974, 1599, 1487, 1225, 1121, 804, 781, 696; ^{1}H-NMR (400 MHz, $CDCl_3$) 1.37 (6H, s, 2 × CH_3), 1.91–2.10 (2H, m, CH_2), 2.78–2.99 (2H, m, CH_2), 6.99–7.05 (2H, m, 2 × CH), 7.07–7.14 (1H, m, CH), 7.16 (2H, dd, J = 4.5, 1.5 Hz, 2 × CH), 7.26–7.35 (2H, m, 2 × CH), 8.51 (2H, dd, J = 4.5, 1.5 Hz, 2 × CH); ^{13}C-NMR (101 MHz, $CDCl_3$) 26.8 (2 × CH_3), 30.0 (CH_2), 43.0 (CH_2), 79.8 (C), 123.5 (CH), 123.9 (2 × CH), 124.0 (2 × CH), 129.1 (2 × CH), 149.9 (2 × CH), 151.7 (C), 155.3 (C); *m/z* (ESI) 242.1533 ($[M+H]^+$. $C_{16}H_{20}ON$ requires 242.1539).

2-(3′-Methyl-3′-phenoxybutyl)pyridine (31b)

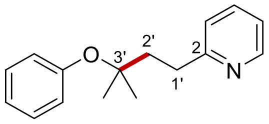

2-(3′-Methyl-3′-phenoxybutyl)pyridine (**31b**) was synthesised as described for **20a** using 2-vinylpyridine (0.0315 g, 0.300 mmol), isopropyl phenyl ether (**S25**, 0.123 g, 0.900 mmol) with the exception that the reaction was irradiated with purple light. Purification by flash chromatography eluting with 30 to 50% ethyl acetate in hexane gave 2-(3′-methyl-3′-phenoxybutyl)pyridine (**31b**) as a yellow oil (0.0434 g, 60%). ν_{max}/cm^{-1} (neat) 2974, 1589, 1487, 1433, 1366, 1227, 1121, 87, 696; ^{1}H-NMR (400 MHz, $CDCl_3$) 1.35 (6H, s, 2 × CH_3), 2.05–2.15 (2H, m, CH_2), 2.97–3.09 (2H, m, CH_2), 6.98–7.13 (4H, m, 4 × CH), 7.19 (1H, d, *J* = 7.8 Hz, 3-H), 7.26 (2H, t, *J* = 7.9 Hz, 2 × CH), 7.59 (1H, td, *J* = 7.7, 1.8 Hz, CH), 8.53 (1H, d, *J* = 4.8 Hz, CH); ^{13}C-NMR (101 MHz, $CDCl_3$) 26.8 (2 × CH_3), 33.3 (CH_2), 42.2 (CH_2), 80.2 (C), 121.1 (CH), 122.9 (CH), 123.3 (CH), 124.1 (2 × CH), 129.1 (2 × CH), 136.5 (CH), 149.4 (CH), 155.5 (C), 162.4 (C); *m/z* (ESI) 242.1539 ([M+H]$^+$. $C_{16}H_{20}ON$ requires 242.1539).

4-(2′-(1″-Phenoxycyclobutyl)ethyl)pyridine (31c)

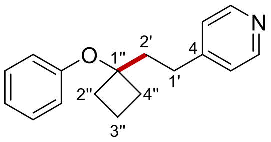

4-(2′-(1″-Phenoxycyclobutyl)ethyl)pyridine (**31c**) was synthesised as described for **20a** using 4-vinylpyridine (0.0315 g, 0.300 mmol), cyclobutyl phenyl ether (**S26**, 0.133 g, 0.900 mmol) and blue light. Purification by flash chromatography using alumina and eluting with 40 to 80% ethyl acetate in hexane gave 2-(3′-methyl-3′-phenoxybutyl)pyridine (**31c**) as a yellow oil (0.0510 g, 67%). ν_{max}/cm^{-1} (neat) 2936, 1599, 1489, 1416, 1229, 1167, 941, 752, 692; ^{1}H-NMR (400 MHz, $CDCl_3$) 1.61–1.80 (1H, m, 3″-*H*H), 1.81–1.20 (1H, m, 3″-H*H*), 2.16–2.29 (4H, m, 2′-H_2, 2″-*H*H, 4″-*H*H), 2.48 (2H, dt, *J* = 12.2, 10.0 Hz, 2″-H*H* and 4″-H*H*), 2.60–2.72 (2H, m, 1′-H_2), 6.84 (2H, d, *J* = 7.9 Hz,), 6.96 (1H, t, *J* = 7.4 Hz, CH), 7.00 (2H, d, *J* = 5.3 Hz, 3-H and 5-H), 7.26 (2H, dd, *J* = 7.9, 7.4 Hz, 2 × CH), 8.43 (2H, d, *J* = 5.3 Hz, 2-H and 5-H); ^{13}C-NMR (101 MHz, $CDCl_3$) 13.6 (CH_2), 29.1 (CH_2), 34.1 (2 × CH_2), 35.7 (CH_2), 81.0 (C), 118.0 (2 × CH), 121.2 (CH), 123.9 (2 × CH), 129.5 (2 × CH), 149.8 (2 × CH), 151.4 (C), 155.6 (C); *m/z* (ESI) 254.1539 ([M+H]$^+$. $C_{17}H_{20}ON$ requires 254.1539).

2-(2′-(1″-Phenoxycyclobutyl)ethyl)pyridine (31d)

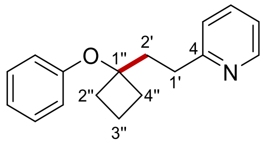

2-(2′-(1″-Phenoxycyclobutyl)ethyl)pyridine (**31d**) was synthesised as described for **20a** using 2-vinylpyridine (0.0315 g, 0.300 mmol), cyclobutyl phenyl ether (**S26**, 0.133 g, 0.900 mmol) and blue light. Purification by flash chromatography using alumina and eluting with 10% ethyl acetate in hexane gave 2-(3′-methyl-3′-phenoxybutyl)pyridine (**31d**) as a yellow oil (0.0496 g, 65%). ν_{max} (neat) 2934, 1593, 1491, 1229, 941, 748, 692; ^{1}H-NMR (400 MHz, $CDCl_3$) 1.68–1.94 (2H, m, 3″-H_2), 2.22–2.34 (2H, m, 2″-*H*H and 4″-*H*H), 2.35–2.57 (4H, m, 2′-H_2, 2″-H*H* and 4″-H*H*), 2.82–2.94 (2H, m, 1′-H_2), 6.84–6.90 (2H, m, 2 × CH), 6.92–6.99 (1H, m, CH), 7.04 (1H, d, *J* = 7.8 Hz, 3-H), 7.07 (1H, dd, *J* = 7.7, 4.8 Hz, 5-H), 7.21–7.32 (2H, m, 2 × CH), 7.53 (1H, td, *J* = 7.7, 1.8 Hz, 4-H), 8.51 (1H, dd, *J* = 4.8 Hz, 6-H); ^{13}C-NMR (101 MHz, $CDCl_3$) 13.6 (CH_2), 32.2 (CH_2), 34.1 (2 × CH_2), 34.7 (CH_2), 81.3 (C), 118.0 (2 × CH), 120.9 (CH), 121.1 (CH), 123.1 (CH), 129.4 (2 × CH), 136.4 (CH), 149.3 (CH), 155.8 (C), 162.0 (C); *m/z* (ESI) 254.1538 ($[M+H]^+$. $C_{17}H_{20}ON$ requires 254.1539).

4-(2′-(1″-Phenoxycyclopentyl)ethyl)pyridine (31e)

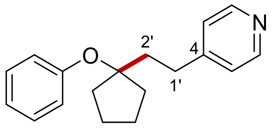

4-(2′-(1″-Phenoxycyclopentyl)ethyl)pyridine (**31e**) was synthesised as described for **20a** using 4-vinylpyridine (0.0315 g, 0.300 mmol), cyclopentyl phenyl ether (**S27**, 0.147 g, 0.900 mmol) and blue light. Purification by flash chromatography using alumina and eluting with 50 to 80% ethyl acetate in hexane gave 4-(2′-(1″-phenoxycyclopentyl)ethyl)pyridine (**31e**) as a yellow oil (0.0617 g, 77%). ν_{max} (neat) 2957, 1599, 1489, 1231, 1196, 804, 752, 694; ^{1}H-NMR (400 MHz, $CDCl_3$) 1.60–1.93 (6H, m, 3 × CH_2), 2.07–2.30 (4H, m, 2 × CH_2), 2.70–2.89 (2H, m, CH_2), 6.95–7.08 (5H, m, 5 × CH), 7.23–7.34 (2H, m, 2 × CH), 8.47 (2H, dd, *J* = 4.5, 1.4 Hz, 2-H and 6-H); ^{13}C-NMR (101 MHz, $CDCl_3$) 24.2 (2 × CH_2), 30.4 (CH_2), 37.9 (2 × CH_2), 39.0 (CH_2), 90.9 (C), 120.5 (2 × CH), 121.9 (CH), 123.9 (2 × CH), 129.4 (2 × CH), 149.8 (2 × CH), 151.6 (C), 155.9 (C); *m/z* (ESI) 268.1695 ($[M+H]^+$. $C_{18}H_{22}ON$ requires 268.1696).

2-(2′-(1″-Phenoxycyclopentyl)ethyl)pyridine (31f)

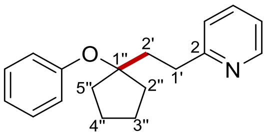

2-(2′-(1″-Phenoxycyclopentyl)ethyl)pyridine (**31f**) was synthesised as described for **20a** using 2-vinylpyridine (0.0315 g, 0.300 mmol), cyclopentyl phenyl ether (**S27**, 0.147 g, 0.900 mmol) with the exception that the reaction was irradiated with purple light. Purification by flash chromatography using alumina and eluting with 5 to 20% ethyl acetate in hexane gave 4-(2′-(1″-phenoxycyclopentyl)ethyl)pyridine (**31f**) as a yellow oil (0.0564 g, 70%). ν_{max} (neat) 2957, 1591, 1489, 1231, 1196, 748, 694; ^{1}H-NMR (400 MHz, $CDCl_3$) 1.50–1.85 (6H, m, 2″-*H*H, 3″-H_2, 4″-H_2 and 5″-*H*H), 2.01–2.11 (2H, m, 2″-H*H*, and 5″-H*H*), 2.18–2.25 (2H, m, 2′-H_2), 2.86–2.95 (2H, m, 1′-H_2), 6.86–7.02 (5H, m, 5 × CH), 7.13–7.21 (2H, m, 2 × CH), 7.46 (1H, td, *J* = 7.7, 1.8 Hz, 4-H), 8.43 (1H, d, *J* = 4.2 Hz, 6-H); ^{13}C-NMR (101 MHz, $CDCl_3$) 24.2 (2 × CH_2), 33.6 (CH_2), 37.8 (2 × CH_2), 38.1 (CH_2), 91.3 (C), 120.8 (2 × CH), 121.1 (CH), 121.7 (CH), 122.9 (CH), 129.3 (2 × CH), 136.4 (CH), 149.4 (CH), 156.1 (C), 162.3 (C); *m/z* (ESI) 268.1694 ([M+H]$^+$. $C_{18}H_{22}ON$ requires 268.1696).

5. Regioselectivity with *para*-, *meta*- and *ortho*-methylanisole

The use of *para*-, *meta*- and *ortho*-methylanisole under the optimised reaction conditions gave a range of different regioisomers depending upon the isomer. *para*-Methylanisole gave diester **3p** in 67% yield when subjected to the reaction conditions. When the methyl group was *meta* to the methoxy functional group, the reaction occurred selectively adjacent to the oxygen atom and **3q** (50%) was given. A mixture of isomers was produced in a 1.0:1.2 ratio when *ortho*-methylanisole was used as substrate in the reaction and this gave **S29** and **S30** in a combined yield of 40%. In these reactions, the arene was the limiting reagent and this gives rise to the differences in yield with scheme 2.

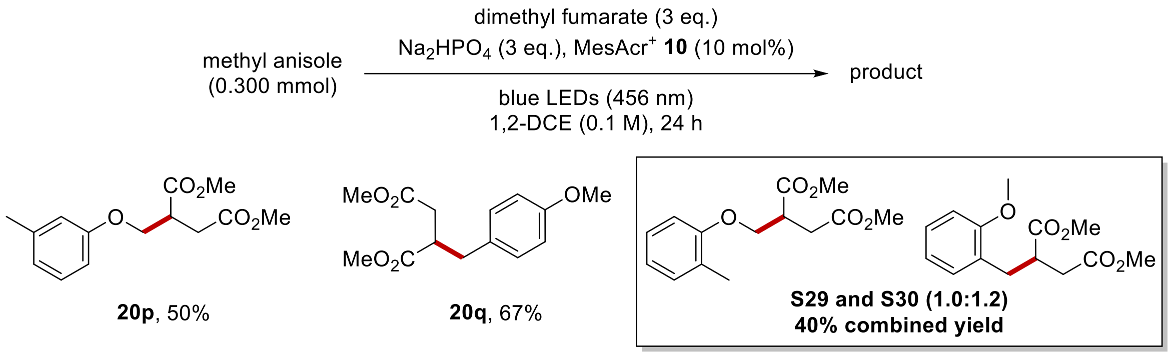

6. Reaction with 3-methoxypropylbenzene (25)

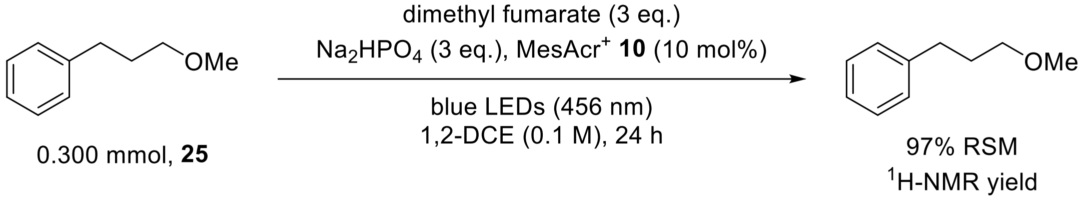

To a dry microwave vial was added 3-methoxypropylbenzene (**25**, 0.0451 g, 0.300 mmol), dimethyl fumarate (0.129 g, 0.900 mmol), disodium hydrogen phosphate (0.127 g, 0.900 mmol), acridinium catalyst **10** and anhydrous 1,2-dichloroethane (3 mL). The reaction mixture was sparged with argon for 5 minutes, afterwards it was stirred and irradiated with blue light (456 nm) for 24 h. The reaction mixture was filtered to remove the inorganic base and concentrated *in vacuo*. The resulting residue was dissolved in $CDCl_3$ (5 mL) and 1,1,2,2-tetrachloroethane (0.0408 g, 0.243 mmol, 5.97 ppm) was added as internal standard. The average of the three methylene groups (2.406) were used in the determining the amount of starting material that was returned.

$$moles\ of\ \mathbf{25} = \left(\frac{2.406}{2.00}\right) \times 0.243\ mmol = 0.292\ mmol\ (97\%)$$

^{1}H-NMR spectrum of the crude reaction mixture

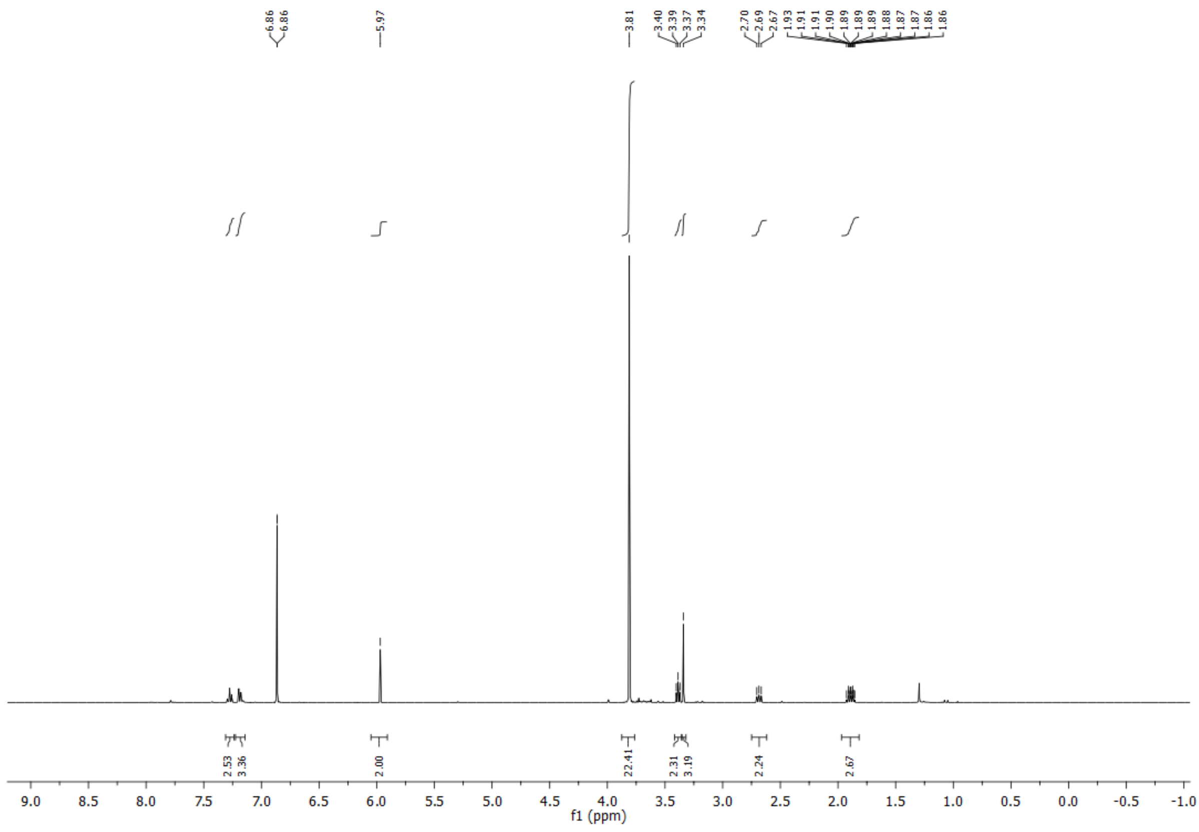

7. Competition experiments

Phenetole and mesitylene

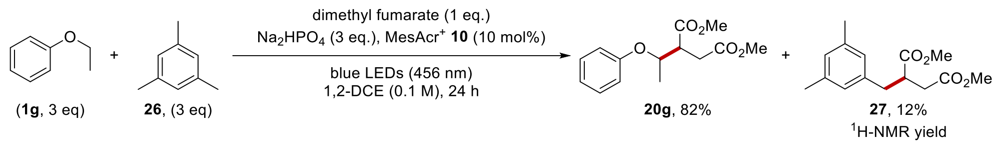

To a dry microwave vial was added ethyl phenyl ether (**1g**, 0.110 g, 0.900 mmol), mesitylene (**26**, 0.108 g, 0.900 mmol), dimethyl fumarate (0.0432 g, 0.300 mmol), disodium hydrogen phosphate (0.127 g, 0.900 mmol), MesAcr$^+$ **10** and anhydrous 1,2-dichloroethane (3 mL). The reaction mixture was sparged with argon for 5 minutes, afterwards it was stirred and irradiated with blue light (456 nm) for 24 h. An aliquot was taken for analysis by GCMS which indicated the crude reaction mixture had four components: Mesitylene (7.66) ethyl phenyl ether (7.90 minutes), the two diastereoisomers of **20g** (13.54 minutes and 13.58 minutes) and **27** (13.71 minutes). ^{1}H-NMR indicated there was a 6.9:1 ratio of **20g** to **27**. Purification of the crude reaction mixture with flash chromatography eluting with 40% diethyl ether in hexane gave diester **20g** in 82% yield and the yield of **27** was calculated to be 12%.

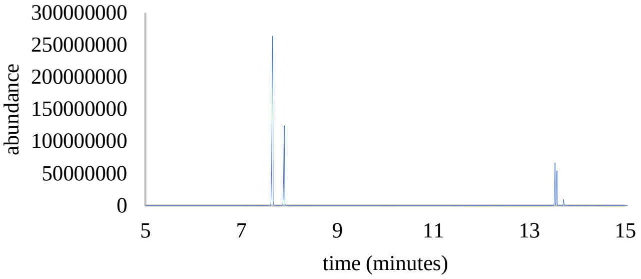

<u>^{1}H-NMR spectrum of crude reaction mixture</u>

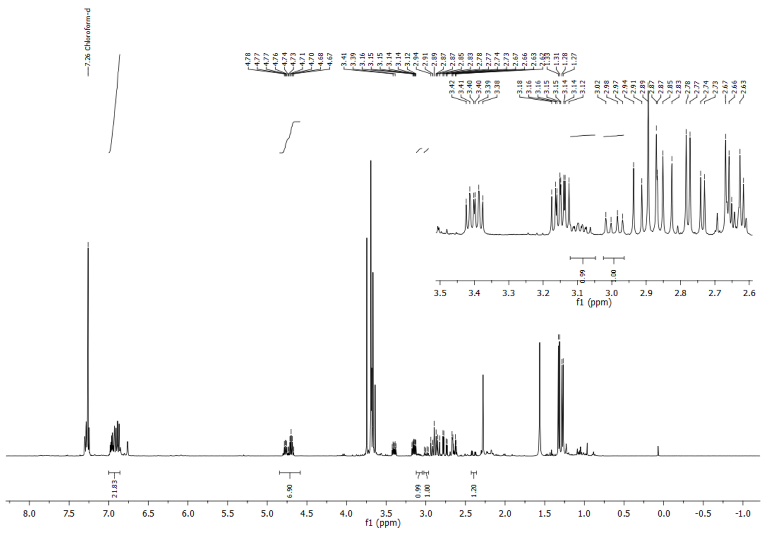

Ethyl phenyl ether and 4-(trifluoromethyl)anisole

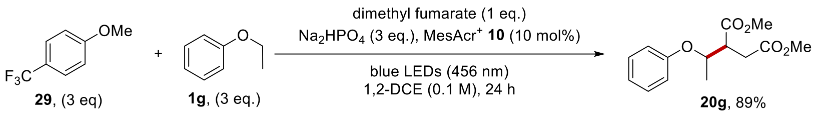

To a dry microwave vial was added ethyl phenyl ether (**1g**, 0.110 g, 0.900 mmol), 4-(trifluoromethyl)anisole (**29g**, 0.109 g, 0.900 mmol), dimethyl fumarate (0.0432 g, 0.300 mmol), disodium hydrogen phosphate (0.127 g, 0.900 mmol), $MesAcr^+$ **10** and anhydrous 1,2-dichloroethane (3 mL). The reaction mixture was sparged with argon for 5 minutes, afterwards it was stirred and irradiated with blue light (456 nm) for 24 h. An aliquot was taken for analysis by GCMS which indicated the crude reaction mixture had three main components: ethyl phenyl ether (7.37 minutes), 4-(trifluoromethyl)anisole (7.74 minutes) and the two diastereoisomers of **20g** (13.39 minutes and 13.43 minutes). Purification of the crude reaction mixture with flash chromatography eluting with 40% diethyl ether in hexane gave diester **20g** in 89% yield.

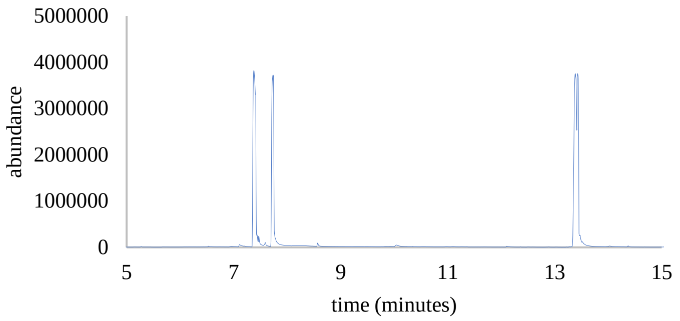

<u>^{1}H-NMR spectrum of crude reaction mixture</u>

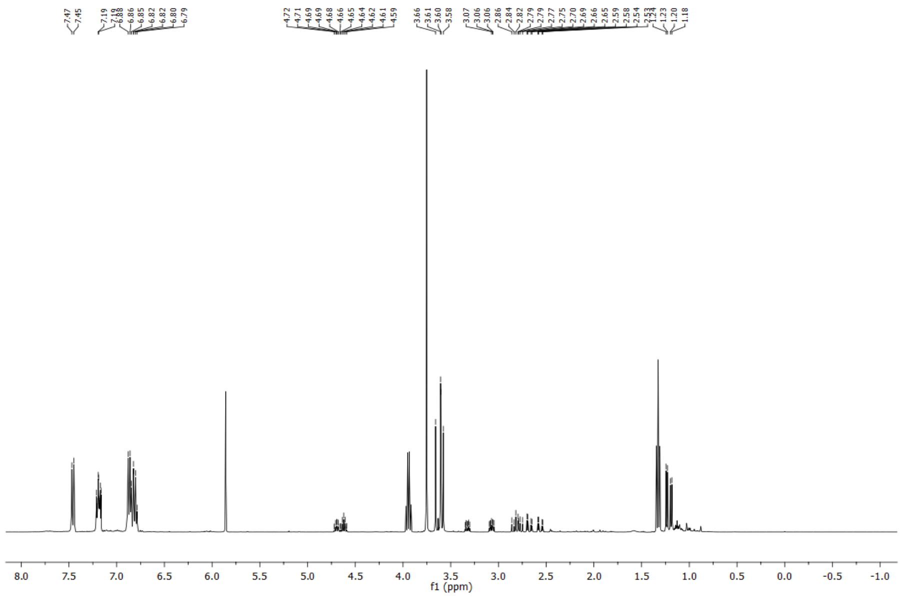

Ethyl phenyl ether and iodobenzene

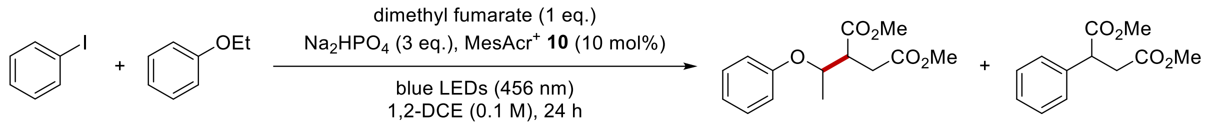

30, (3 eq) **1g**, (3 eq) **20g**, 95% **S24**, trace

To a dry microwave vial was added ethyl phenyl ether (**1g**, 0.110 g, 0.900 mmol), iodobenzene (**30**, 0.183 g, 0.900 mmol), dimethyl fumarate (0.0432 g, 0.300 mmol), disodium hydrogen phosphate (0.127 g, 0.900 mmol), MesAcr$^+$ **10** and anhydrous 1,2-dichloroethane (3 mL). The reaction mixture was sparged with argon for 5 minutes, afterwards it was stirred and irradiated with blue light (456 nm) for 24 h. An aliquot was taken for analysis by GCMS which indicated the crude reaction mixture had four components: ethyl phenyl ether (7.37 minutes), iodobenzene (8.23 minutes), the two diastereoisomers of **20g** (13.39 minutes and 13.43 minutes) and dimethyl phenylsuccinate **S24** (12.06 minutes). The diester **S24** was not observed by ^{1}H-NMR and it was deemed a trace by-product. Purification of the crude reaction mixture with flash chromatography eluting with 40% diethyl ether in exane gave diester **20g** in 95% yield

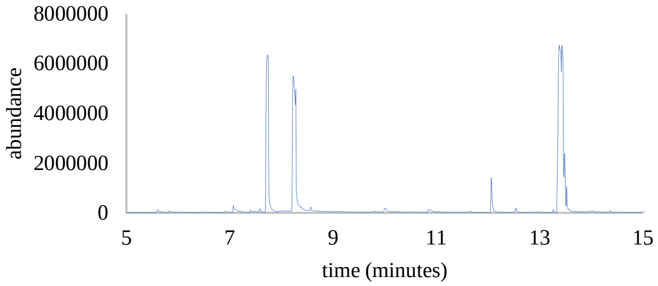

<u>^{1}H-NMR spectrum of crude reaction mixture</u>

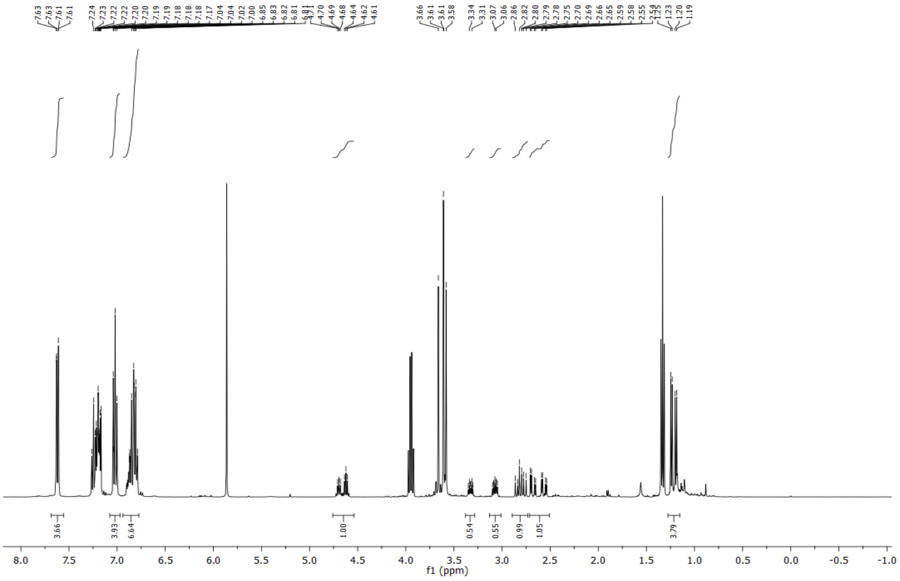

8. Stern-Volmer Experiments

Stern-Volmer quenching constants

All fluorescence measurements were performed on a Varian Cary Eclipse Fluorescence Spectrophotometer. The excitation wavelength was at 430 nm and not 456 nm due to the presence of Rayleigh scattering bands when the catalyst was excited at 456 nm, other parameters: Emission window 445–700 nm, excitation slit width 5 nm, emission slit width 5 nm, scan rate 150 nm/min, averaging time 0.4000 s, data interval 1.000 nm.

Quenching with anisole and general procedure

The quenching of the acridinium catalyst with anisole was performed as follows: In a 100 mL volumetric flask was prepared a solution of $MesAcr^{+}$ **10** (0.0299 g, 0.0521 mmol) in DCE (100 mL, c = 521.4 μM). To a separate 100 mL volumetric flask was added anisole (1.083 g, 10.01 mmol) in DCE (100 mL, c = 0.1001 M). The following samples were made up as below in a 10 mL volumetric flask and this was done by adding the amount of stock solution required then DCE was added until the volume was 10 mL.

Sample	Volume of solution	Concentration of acridinium	Amount of acridinium stock solution required	Concentration of anisole	Amount of anisole stock solution required
1	10 mL	5 μM	96 μL	0 mM	0 μL
2	10 mL	5 μM	96 μL	2 mM	200 μL
3	10 mL	5 μM	96 μL	4 mM	400 μL
4	10 mL	5 μM	96 μL	8 mM	799 μL
5	10 mL	5 μM	96 μL	12 mM	1,198 μL
6	10 mL	5 μM	96 μL	16 mM	1,598 μL
7	10 mL	5 μM	96 μL	20 mM	1,997 μL
8	10 mL	5 μM	96 μL	30 mM	2,996 μL

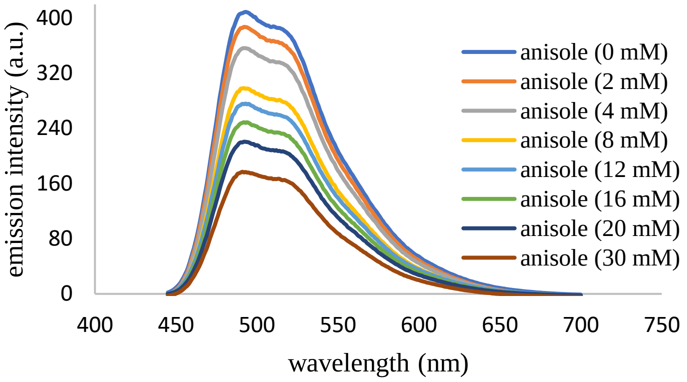

The fluorescence intensity of each sample was measured and the Stern-Volmer constant was calculated with the following equation $\frac{F°}{F} = 1 + K_{SV}[Q]$.[27] In the equation F° is the fluorescence intensity when the quencher was absent, F is the fluorescence intensity of the sample, K_{SV} is the Stern-Volmer quenching constant, and Q is concentration of quencher. From this data the Stern-Vomer quenching constant for anisole was determined to be 44.8 M^{-1}.

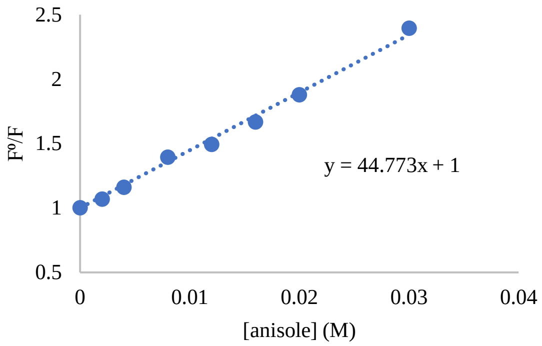

Stern-Volmer experiments with other compounds

Other Stern-Volmer experiments were performed with other using the method detailed above. The results of these experiments are shown on the next page.

Ethyl phenyl ether (phenetole)

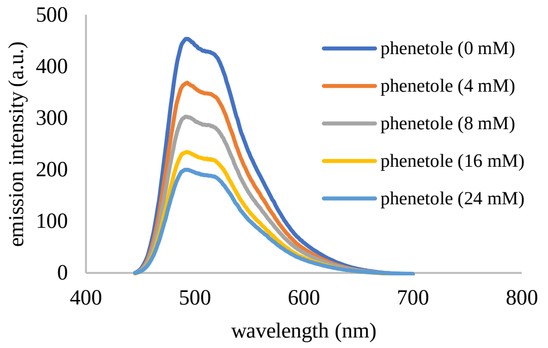

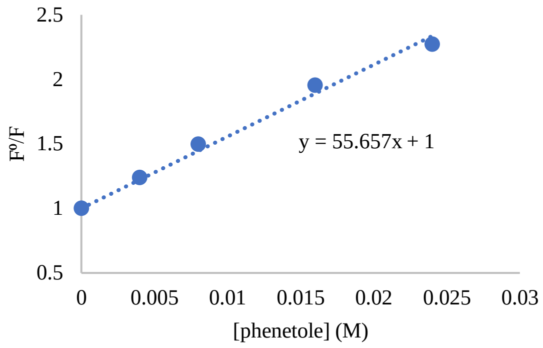

para-Dimethoxybenzene

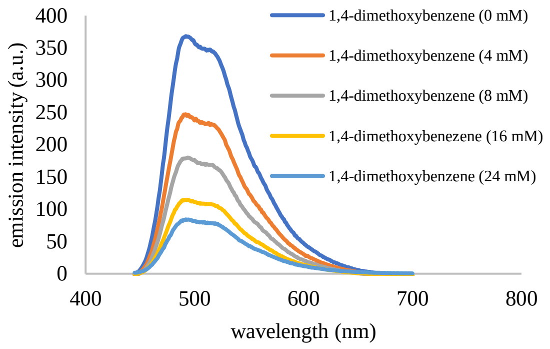

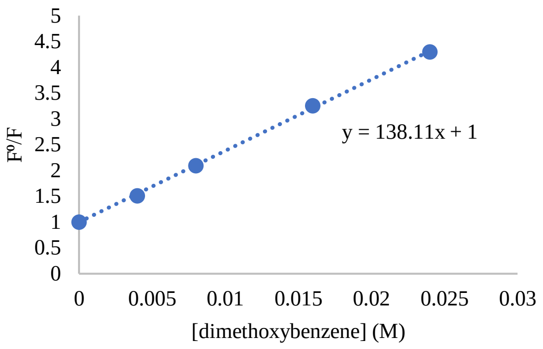

Mesitylene

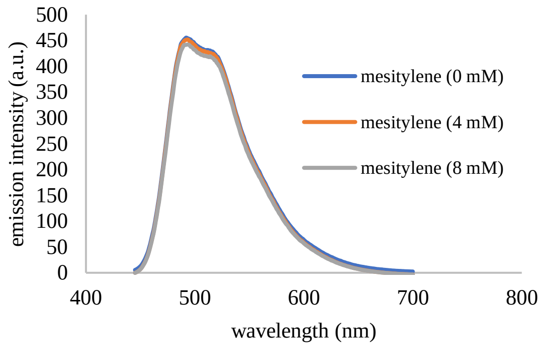

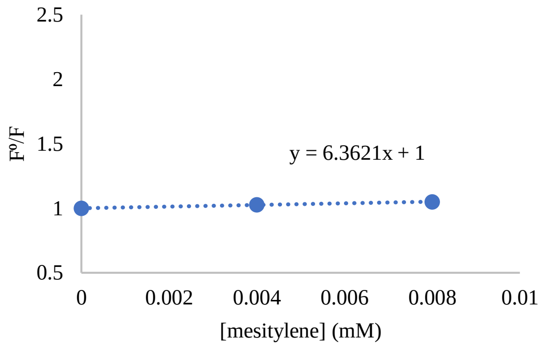

3-Methoxylpropylbenzene

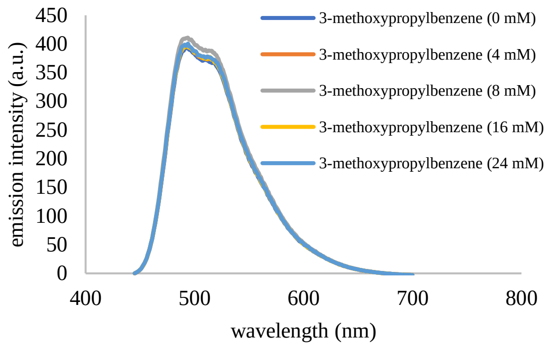

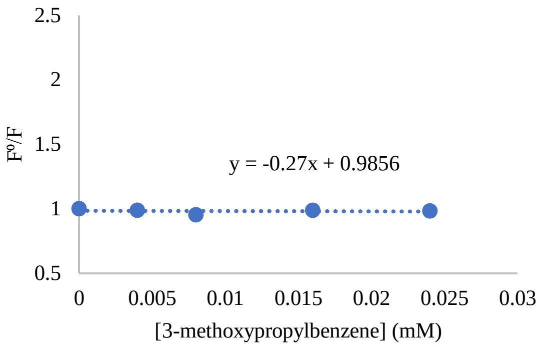

Sodium hydrogen phosphate

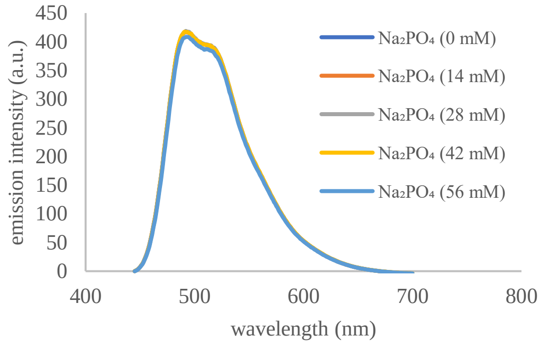

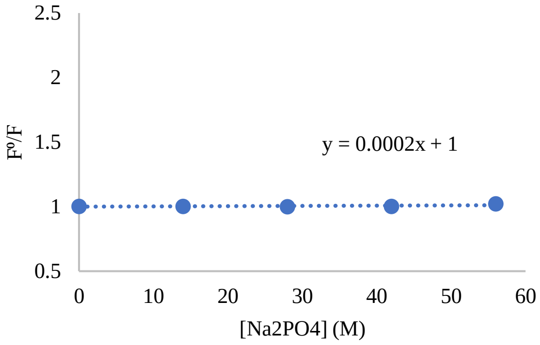

Summary of Stern-Volmer experiments

From the results shown above there are several observations. All the substrates that were used in the reaction that generated product (anisole, phenetole, 1,4-dimethoxybenzene and mesitylene) there was effective quenching of the catalyst. When a substrate was tested that did not form product (3-methoxypropylbenzene) there was no quenching of the catalyst. Furthermore, when the base, disodium hydrogen phosphate was tested there was also no quenching of the catalyst. The observations indicate that the reaction propagates via direct oxidation of the substrate from the excited catalyst and not via HAT by a phosphate-oxyl radical.

PCET experiment

Another potential reaction pathway is proton-coupled electron transfer (PCET).[27] To evaluate if this reaction pathway is feasible under our reaction conditions Na_2HPO_4 (10 mg) was added to the cuvette that was being used to investigate the quenching between 3-methoxypropylbenzene and MesAcr$^+$ (**10**). The addition of base to the mixture of 3-methoxypropylbenzene and MesAcr$^+$ (**10**) did not result in any quenching of the catalyst and thus PCET pathways for this reaction was ruled out.

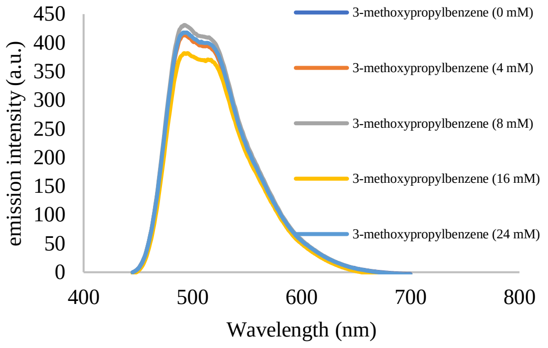

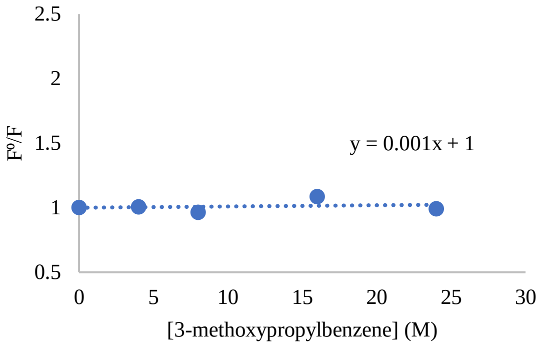

9. Investigation into the use of different loadings of reagent

It is understandable that using excess amounts of reagent does not meet sustainability goals, so a number of reactions were performed to understand if lower amounts of reagent could be used in the reaction. It was found that in all cases the higher loadings of reagents were required to obtain the highest yields, but useful amounts of product could be obtained when lower equivalents were used. Furthermore, the limiting reagent can be switched without any major effect upon the isolated yield of product.

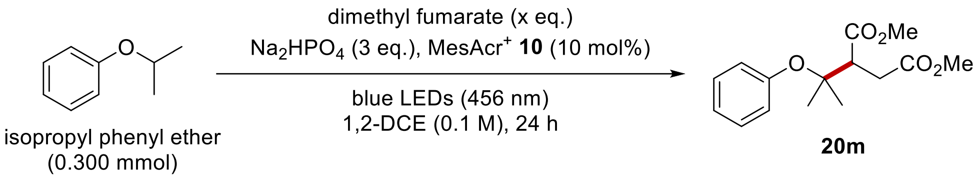

Entry	Alkene (mmol/eq)	Isolated yield
1	0.900 (3 eq.)	81%
2	0.450 (1.5 eq.)	55%
3	0.300 (1.0 eq.)	52%

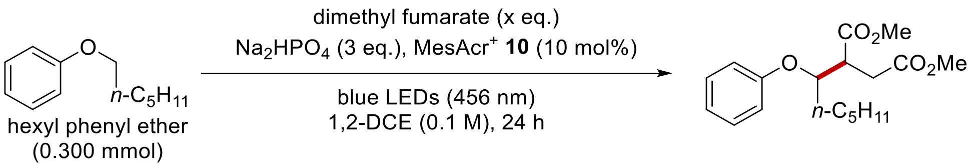

Entry	Alkene (mmol/eq)	Isolated yield
1	0.900 (3 eq.)	75%
2	0.450 (1.5 eq.)	51%
3	0.300 (1.0 eq.)	52%

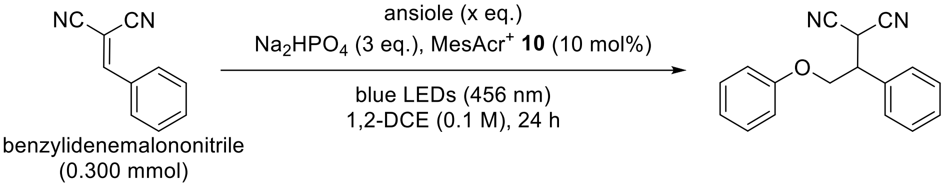

Entry	Ether (mmol/eq)	Isolated yield
1	0.900 (3 eq.)	85%
2	0.450 (1.5 eq.)	79%
3	0.300 (1.0 eq.)	71%

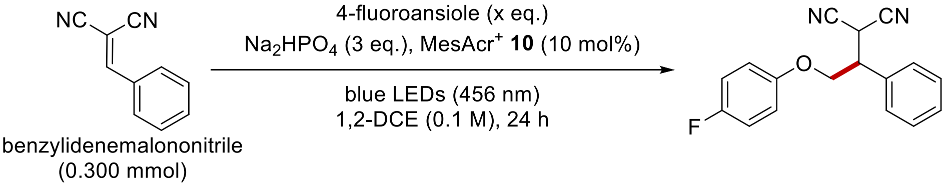

Entry	Ether (mmol/eq)	Isolated yield
1	0.900 (3 eq.)	85%
2	0.450 (1.5 eq.)	70%
3	0.300 (1.0 eq.)	63%

10. Quantum yield

General information

The quantum yield of the reaction was evaluated using an adopted procedure that was reported by Glorius.[28] To obtain a quantum yield value for this reaction the photon flux of the LED light being used must be known and this can be determined by potassium ferrioxalate actinometry.[29] While the majority of our experiments were performed with 456 nm of light potassium ferrioxalate has better sensitivity at lower wavelengths, so the quantum yield measurements were performed at 390 nm and this is similar to previous work where quantum yields were reported.[30,31] Initial investigation into determining the quantum yield of this reaction yield some inconsistent results due to the intense irradiation of our LED lamps therefore a few amendments were made to the procedure of Glorius *et al.* to correct for this. While performing the irradiation the potassium ferrioxalate solution was subject to intense stirring and this was to prevent any recombination reactions due to slow mass transport.[32] It has been reported that inefficient stirring results in an altered quantum yield value for the ferrioxalate actinometer. Another observation that was made during initial investigations was the generation of a precipitate after the irradiated ferrioxalate solution was developed by the 1,10-phenanthroline solution. It has been reported that the formation of this precipitate affects chemical equilibria and results in "systemically wrong measurements."[33,34] To correct for this, two adjustments were made, the irradiation time was reduced from 90 s to 30 s and the intensity of the Kessil LED lamp was set to its lowest intensity setting (25%).

Calculation of the photon flux

A solution of potassium ferrioxalate (0.737 g) in aq. H_2SO_4 solution (0.05 M, 10 mL) and a buffered solution of 1,10-phenanthroline (25 mg) that was dissolved in a mixture of sodium acetate (5.63 g) and aq. H_2SO_4 solution (0.5 M, 25 mL) were prepared. The buffered solution of 1,10-phenanthroline required vigorous shaking to ensure homogeneity. Both solutions were sensitive to light and were wrapped in foil, stored in the dark and used immediately.[35] To measure the photon flux of the LED lamp, the potassium ferrioxalate solution (2.6 mL) was placed into an oven-dried microwave vial. The ferrioxalate solution was exposed to 390 nm LED lamp (5 cm, 25% intensity setting) for 30 seconds. A non-irradiated sample (2.6 mL of ferrioxalate solution) was also prepared and to both solutions was added the buffered 1,10-phenanthroline solution (0.455 mL) and these solutions were then stirred in the dark (wrapped with foil) for 1 h to ensure complete complexation between the Fe^{2+} ions and the 1,10-phenanthroline ligand. Each solution was analysed by UV-Vis spectroscopy by transferring the solutions into a quartz cuvette (1×1×4 cm). The absorbance of both solutions at 510 nm was measured and the quantity of Fe^{2+} ions released by irradiation of light was calculated using the following equation:

$$\text{mol Fe}^{2+} = \frac{V \times \Delta A_{510\ nm}}{l \times \epsilon}$$

Where V is the total volume of the solution (0.00282 L), ΔA_{510nm} is the difference in absorbance at 510 nm (0.754), l is the path length (1 cm) and ε is the molar absorptivity at 510 nm (11,100 $Lmol^{-1}cm^{-1}$).[28] From this calculation it was determined the amount of $Fe^{2=}$ ions present in solution was 1.5159×10^{-7} moles. With the amount of Fe^{2+} ions being liberated by irradiation of the LED lamp the photon flux of the lamp could be measured, using the following equation:

$$\text{photon flux} = \frac{\text{mol of Fe}^{2+}}{\Phi \times t \times f}$$

In this equation, Φ is defined as the quantum yield of the potassium ferrioxalate actinometer at 390 nm (Φ = 1.12 at 390 nm),[36] t is the irradiation time of the oxalate solution (30 s), and f is the fraction of light absorbed at 390 nm by the ferrioxalate solution. The f value was calculated by measuring the UV-Vis spectrum of the ferrioxalate solution and then using the following equation:

$$f = 1 - 10^{-A_{390nm}}$$

In the equation A_{390nm} is the absorption at 390 nm of ferrioxalate solution. From the UV-Vis spectrum shown below, it was calculated that f = 0.9998.

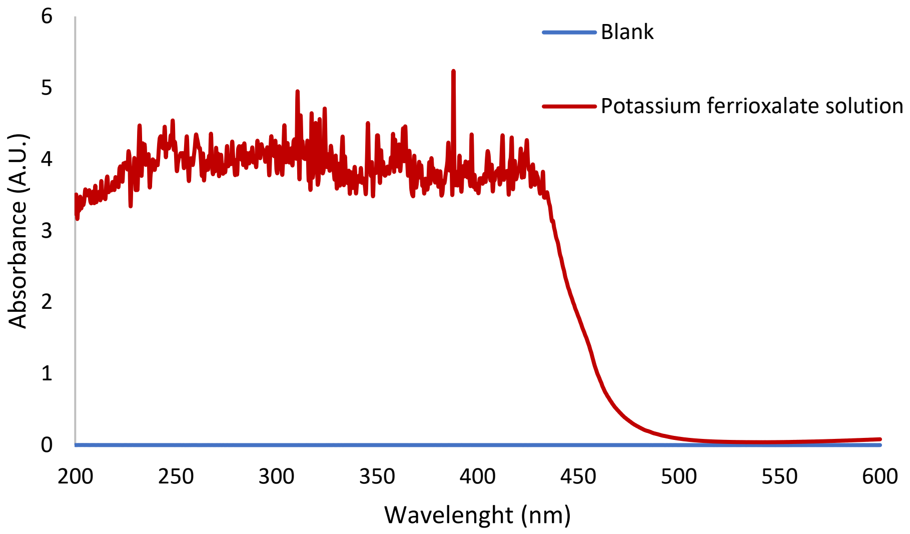

From these calculations it was determined that the photon flux of the LED lamp used was 5.655×10^{-9} mol s^{-1}.

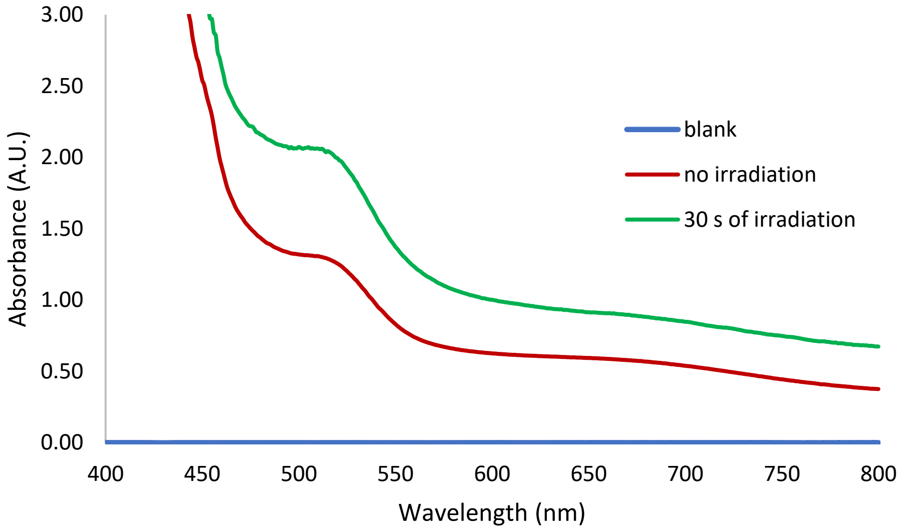

Determination of the reaction quantum yield

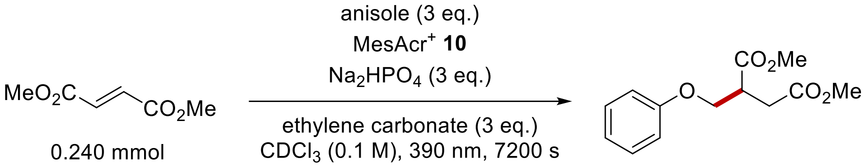

20a

The quantum yield of the reaction was measured using anisole and dimethyl fumarate as substrate. To a 10 mL volumetric flask was added anisole (0.326 mL, 3.00 mmol, 3 eq.), dimethyl fumarate (0.144 g, 1.00 mmol, 1 eq.), ethylene carbonate (internal standard, 0.0881 g, 1.00 mmol, 1.0 eq.), $MesAcr^{+}BF_4^{-}$ (**10**, 0.0566 g, 0.100 mmol, 10 mol%) and $CDCl_3$ (10 mL). This reaction mixture was studied by UV-Vis spectroscopy and this gave an f value of 0.9999. An oven-dried microwave vial was taken and Na_2HPO_4 (0.102 g, 0.720 mmol) was added and 2.4 mL of the reaction mixture stock solution was added to the vial. The vial was irradiated by the Kessil lamp (390 nm, 25% intensity) for 2 h (7,200 s) before the light and stirring was stopped. A 1 mL aliquot of the reaction mixture was taken and studied by ^{1}H-NMR to calculate a yield. It was determined that 0.00145 mmol (1.450×10^{-6} mol)

The quantum yield of the reaction was calculated with the following equation:

$$\Phi = \frac{\text{mol of product formed}}{\text{photon flux} \times \text{f} \times \text{t}}$$

The quantum yield of the reaction mixture was calculated to be 0.04.

11. Reactions with Purple light (390 nm) and Diethyl Ethylidenemalonate

Two reaction mixtures were prepared that contained diethyl ethylidenemalonate (0.300 mmol), anisole (0.900 mmol), MesAcr$^+$ **10** (0.030 mmol), disodium hydrogen phosphate (0.900 mmol) and anhydrous 1,2-dichloroethane (3 mL, 0.1 M). One of these reaction mixtures was irradiated with purple LEDs (390 nm) for 24 h and then a 0.1 mL aliquot was taken, concentrated *in vacuo*, dissolved in $CDCl_3$ and analysed by ^{1}H-NMR spectroscopy. The other reaction mixture was irradiated with blue LEDs (456 nm) for 48 h before an aliquot was removed for analysis by ^{1}H-NMR spectroscopy.

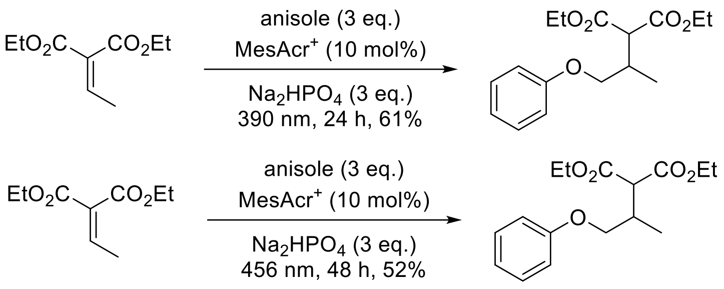

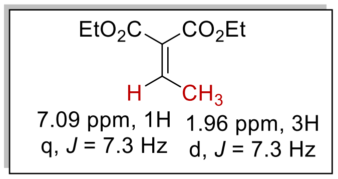

Diethyl ethylidenemalonate has diagnostic signals corresponding to a CH_3 and a CH group at 1.96 and 7.09 ppm respectively. The reaction that was performed with blue light resulted in substantial amounts of starting electrophile being returned after 48 h and this can be seen in the spectrum below:

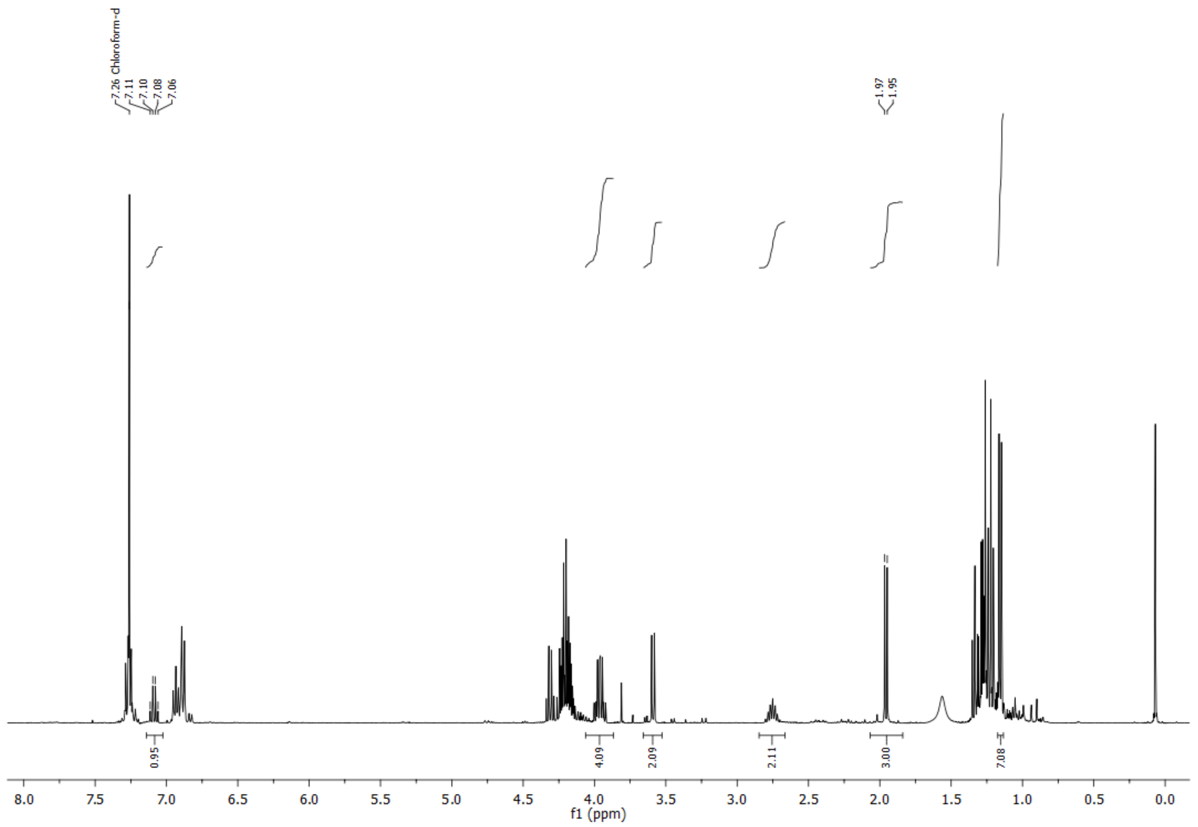

When the reaction was conducted with purple light (390 nm) only trace amounts of the starting electrophile was remained at the end of the reaction (24 h).

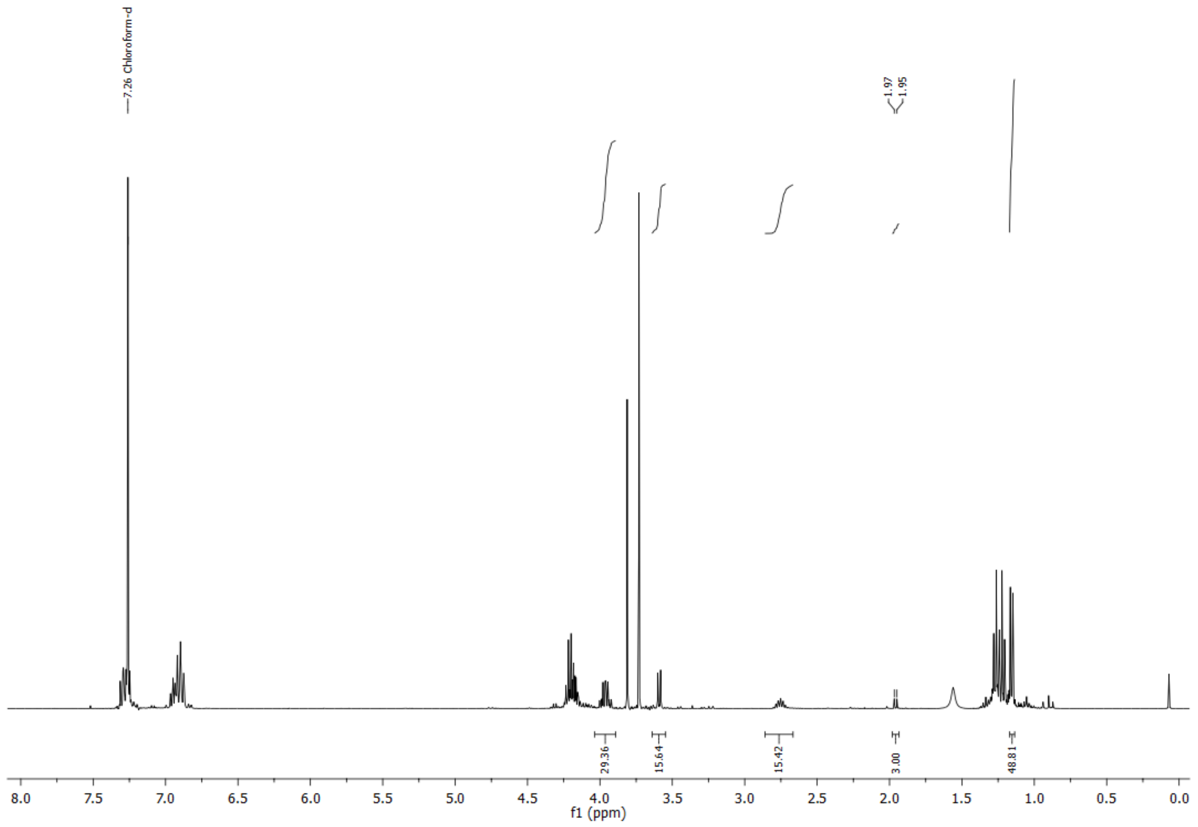

12. Reactions with methyl acrylate in the Giese coupling reaction

Several reactions were performed with methyl acrylate in a Giese coupling reactions using MesAcr$^+$ **10** and these results are summarised here. When methyl acrylate was reacted with anisole using MesAcr$^+$ with blue light ester **S31** (9%) was isolated. As greater yields were found by using a lower wavelength of light this was trialled with this reaction. The use of purple light (390 nm) with the reaction between anisole and methyl acrylate resulted in the formation of two compounds, ester **S31** (36%) and diester **S32** (15%). Buoyed by these results attention was turned to an ether where addition to methyl acrylate can only occur once and thus isopropyl phenyl ether was chosen for this purpose. Irradiation with blue light of a reaction mixture containing isopropyl phenyl ether, methyl acrylate and MesAcr$^+$ resulted in the formation of ester **S33** (12%). It was thought that the combination of purple light with isopropyl phenyl ether would result in a highly efficient Giese reaction with methyl acrylate. The combination of isopropyl phenyl ether, methyl acrylate, MesAcr$^+$ and purple light resulted in the isolation of two compounds, ester **S33** (41%) and chromane **S34** (26%). Cyclisation and the formation of chromane **S34** is thought to be promoted by the Thorpe-Ingold effect due to the presence of the *gem*-dimethyl groups.

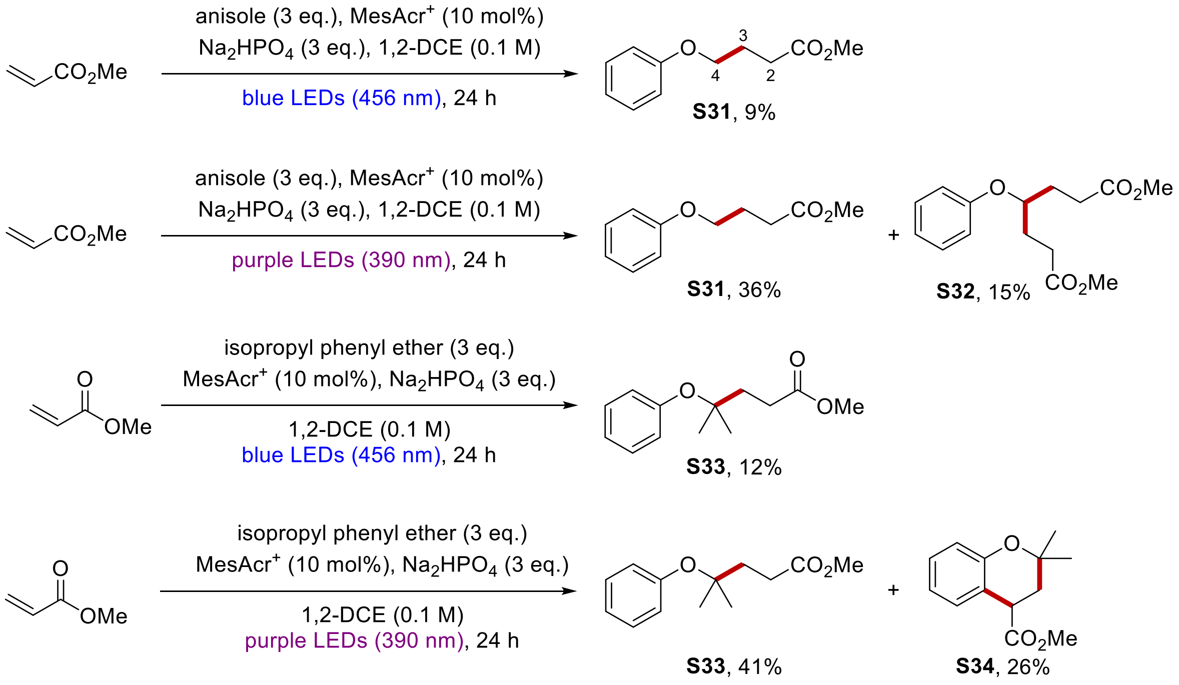

Experimental procedure and spectral data of the reactions involving methyl acrylate

Reaction of methyl acrylate with anisole using blue light

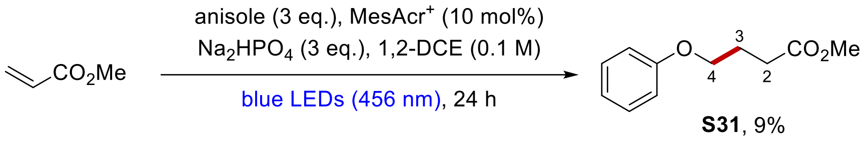

The reaction of methyl acrylate and anisole was carried out as described for the synthesis of **20a** using methyl acrylate (0.0258 g, 0.300 mmol), anisole (0.0973 g, 0.900 mmol) and blue light. Purification by flash chromatography eluting with 50% ethyl acetate in hexane gave methyl 4-phenoxybutanoate (**S31**) as a yellow oil (0.0050 g, 9%). ν_{max}/cm^{-1} (neat): 2951, 2928, 2870, 1734, 1599, 1495, 1242; ^{1}H-NMR (400 MHz, $CDCl_3$) 7.32–7.26 (2H, m, 2 × CH), 6.97–6.91 (1H, m, CH), 6.91–6.86 (2H, m, 2 × CH), 4.01 (2H, t, J = 6.0 Hz, 4-H_2), 3.69 (3H, s, CH_3), 2.54 (2H, t, J = 7.3 Hz, 2-H_2), 2.15–2.08 (2H, m, 3-H_2); ^{13}C-NMR (101 MHz, $CDCl_3$) 173.2 (C), 158.3 (C), 128.9 (2 × CH), 120.2 (CH), 114.0 (2 × CH), 66.0 (CH_2), 51.1 (CH_3), 30.1 (CH_2), 24.2 (CH_2); *m/z* (EI): 194.2 ($[M]^+$, 3), 163.2 (3), 101.2 (34), 94.2 (20), 77.2 (18), 59.1 (100).

Reaction of methyl acrylate with anisole using purple light

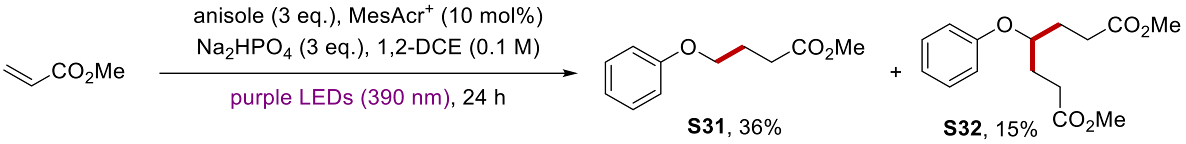

The reaction of methyl acrylate and anisole was carried out as described for the synthesis of **20a** using methyl acrylate (0.0258 g, 0.300 mmol), anisole (0.0973 g, 0.900 mmol) and purple light. At the end of the reaction, 1,1,2,2-tetrachloroethane was added as an internal standard and a ^{1}H-NMR was taken to determine the quantity of the products by measuring the integral of the OC$\underline{H}_2$R and $OCHR_2$ signals. **S31** (36%) and **S32** (15%) were formed.

^{1}H-NMR spectrum of the crude reaction mixture between methyl acrylate and anisole with purple light

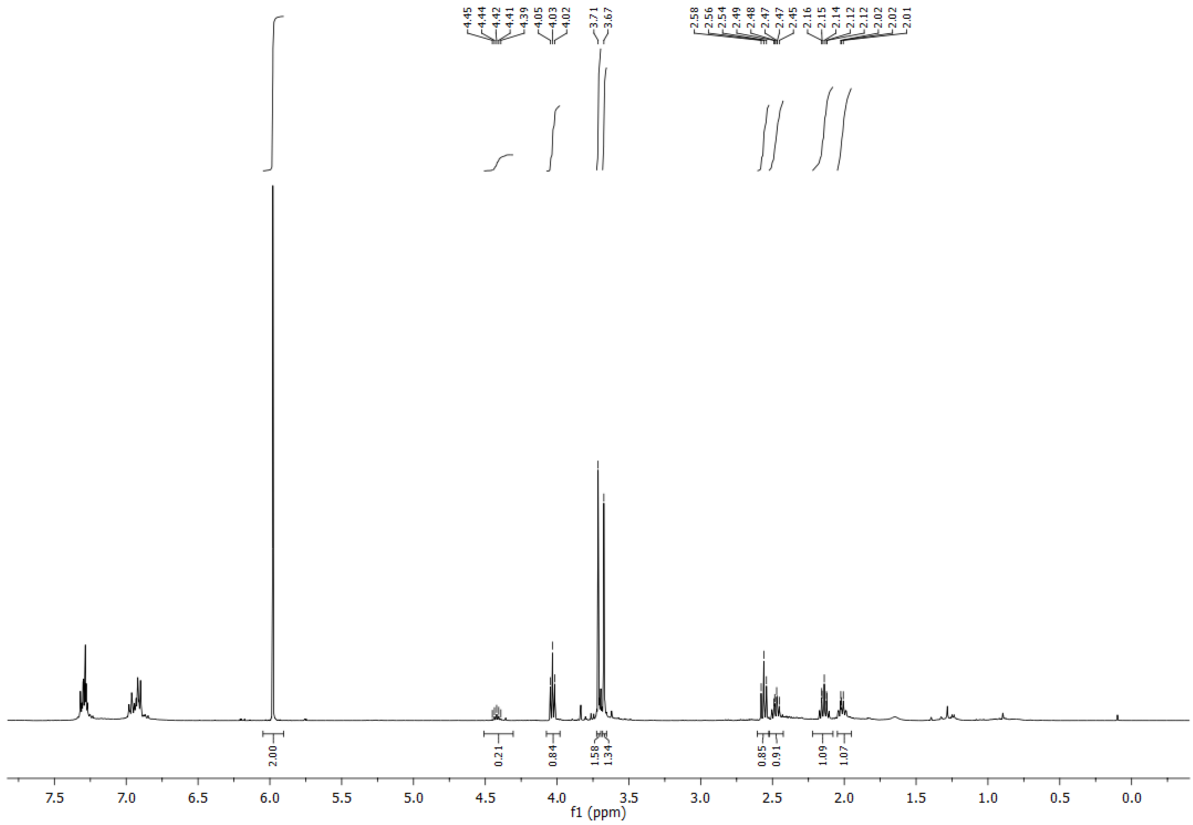

Reaction of methyl acrylate with isopropyl phenyl ether using blue light

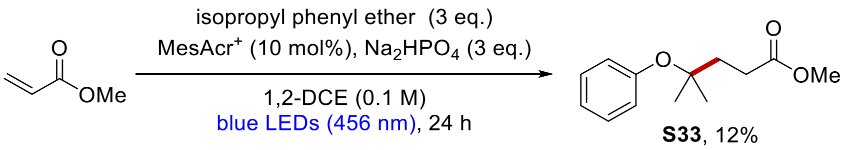

The reaction of methyl acrylate and isopropyl phenyl ether was carried out as described for the synthesis of **20a** using methyl acrylate (0.0258 g, 0.300 mmol), isopropyl phenyl ether (0.1226 g, 0.900 mmol) and blue light. Purification by flash chromatography eluting with 30% diethyl ether in hexane gave methyl 4-methyl-4-phenoxypentanoate (**S33**) as a pale yellow oil (0.0080 g, 12%). ν_{max}/cm^{-1} (neat): 2976, 2936, 1736, 1591, 1487, 1161; ^{1}H-NMR (400 MHz, $CDCl_3$) 7.29–7.22 (2H, m, 2 × CH), 7.10–7.03 (1H, m, CH), 6.99–6.94 (2H, m, 2 × CH), 3.69 (3H, s, CH_3), 2.62–2.54 (2H, m, 2-H_2), 2.05–1.98 (2H, m, 3-H_2), 1.28 (6H, s, 2 × CH_3); ^{13}C-NMR (101 MHz, $CDCl_3$) 174.6 (C), 155.3 (C), 129.1 (2 × CH), 124.0 (2 × CH), 123.6 (CH), 79.4 (C), 51.8 (CH_3), 37.2 (CH_2), 29.4 (CH_2), 26.6 (2 × CH_3); *m/z* (EI): 222.2 ([M]$^+$, 2), 207.1 (4), 191.1 (7), 147.1 (7), 135.1 (14), 129.1 (73), 94.0 (100).

Reaction of methyl acrylate with isopropyl phenyl ether using purple light

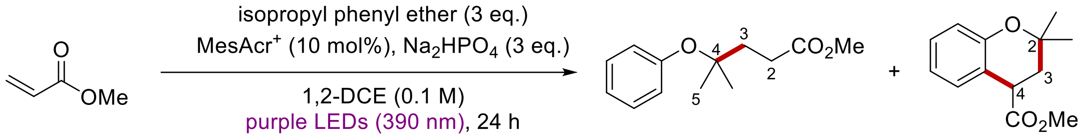

The reaction of methyl acrylate and isopropyl phenyl ether was carried out as described for the synthesis of **20a** using methyl acrylate (0.0258 g, 0.300 mmol), isopropyl phenyl ether (0.123 g, 0.900 mmol) and purple light. Purification by flash chromatography eluting with 15% diethyl ether in hexane gave methyl 4-methyl-4-phenoxypentanoate **S33** and methyl 2,2-dimethylchromane-4-carboxylate **S34** as colourless oils and a 1.0:1.6 mixture that could not be separated by flash chromatography (0.0448 g isolated material and this equates to a 41% yield for methyl 4-methyl-4-phenoxypentanoate and a 26% yield for methyl 2,2-dimethylchromane-4-carboxylate. **Data for methyl 4-methyl-4-phenoxypentanoate S33**: ^{1}H-NMR (400 MHz, $CDCl_3$) 1.30 (6H, s, 5-H_3 and 4-CH_3), 1.99–2.12 (2H, m, 3-CH_2), 2.50–2.73 (2H, m, 2-CH_2), 3.71 (3H, s, OCH_3), 6.99 (2H, ddd, *J* = 6.4, 2.2, 1.1 Hz, 2 × CH), 7.09 (1H, ddd, *J* = 7.1, 2.2, 1.1 Hz, CH), 7.25–7.32 (2H, m, 2 × CH); ^{13}C-NMR (101 MHz, $CDCl_3$) 26.5 (2 × CH_3), 29.3 (CH_2), 37.2 (CH_2), 51.8 (CH_3), 79.4 (C), 123.5 (CH), 124.0 (2 × CH), 129.1 (2 × CH), 155.2 (C), 174.5 (C); *m/z* (ESI) 245.1147 ($[M+Na]^+$. $C_{13}H_{18}O_3Na$ requires 245.1148). **Data for methyl 2,2-dimethylchromane-4-carboxylate S34:** ^{1}H-NMR (400 MHz, $CDCl_3$) 1.30 (3H, s, CH_3), 1.43 (3H, s, CH_3), 2.06 (1H, dd, *J* = 13.5, 6.4 Hz, 3-*H*H), 2.26 (1H, d, *J* = 13.5, 10.6 Hz, 3-H*H*), 3.79 (3H, s, CH_3), 3.90 (1H, dd, *J* = 10.5, 6.4 Hz, 4-H), 6.83 (1H, dd, *J* = 8.7, 1.3 Hz, CH), 6.89 (1H, td, *J* = 7.5, 1.2 Hz, CH), 7.15–7.21 (2H, m, 2 × CH); ^{13}C-NMR (101 MHz, $CDCl_3$) 25.1 (CH_3), 28.8 (CH_3), 36.3 (CH_2), 39.8 (CH), 52.4 (CH_3), 73.7 (C), 117.8 (C), 118.0 (CH), 120.2 (CH), 128.8 (CH), 128.9 (CH), 153.6 (C), 174.3 (C); *m/z* (ESI) 243.0993 ($[M+Na]^+$. $C_{13}H_{16}O_3Na$ requires 243.0992).

13. ^{1}H and ^{13}C NMR Spectra for all Compounds

Methyl 2,4,6-trimethylbenzoate (S8)

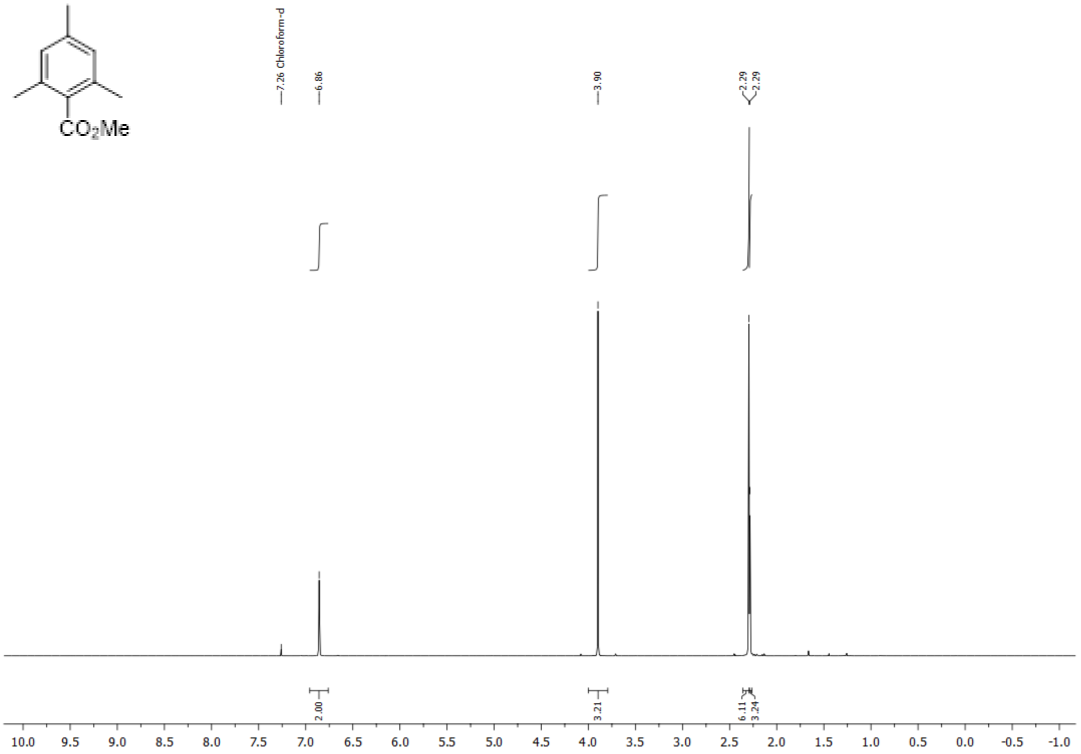

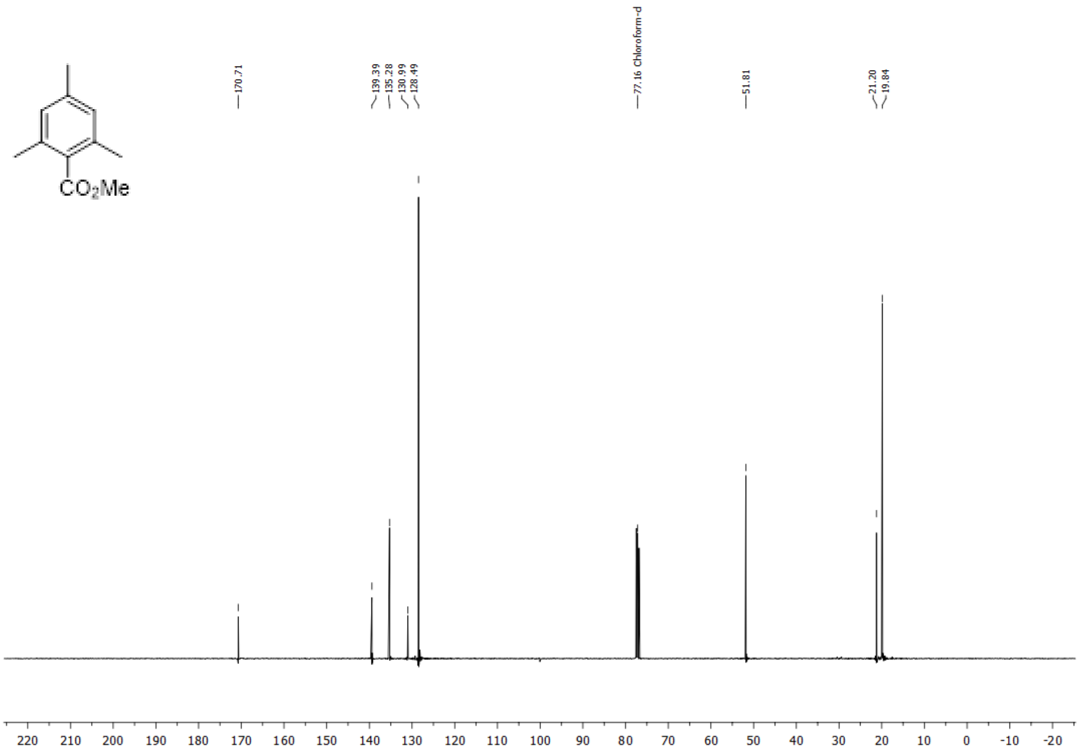

3,3′-Oxybis(*tert*-butylbenzene) (S9)

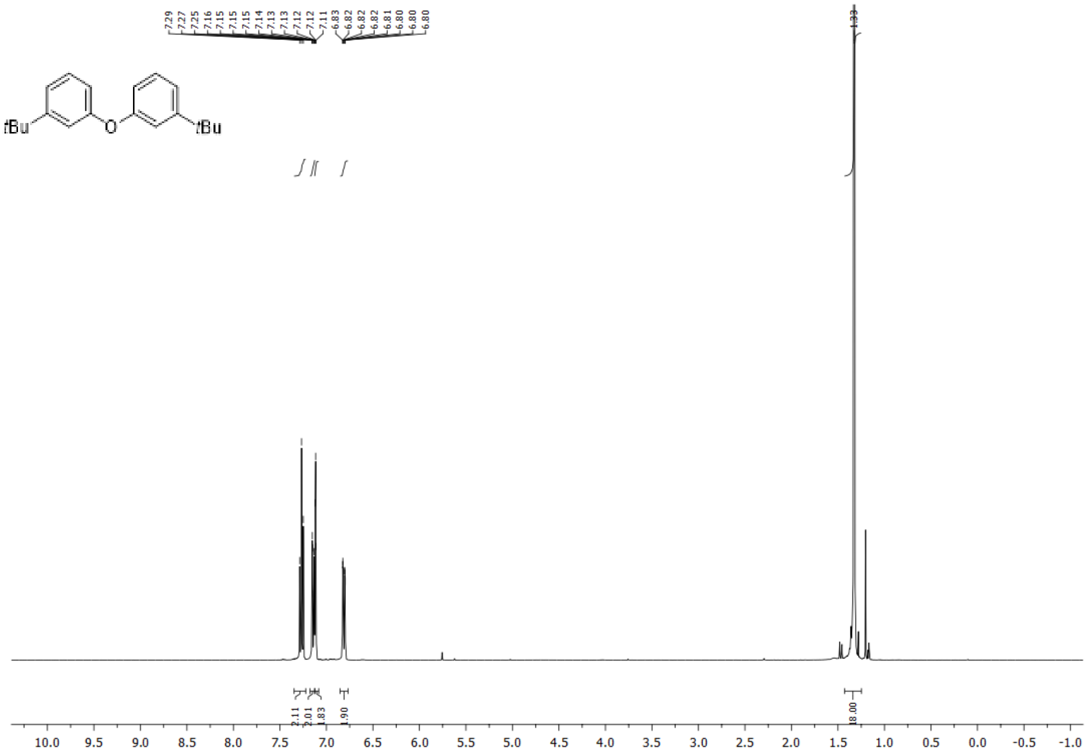

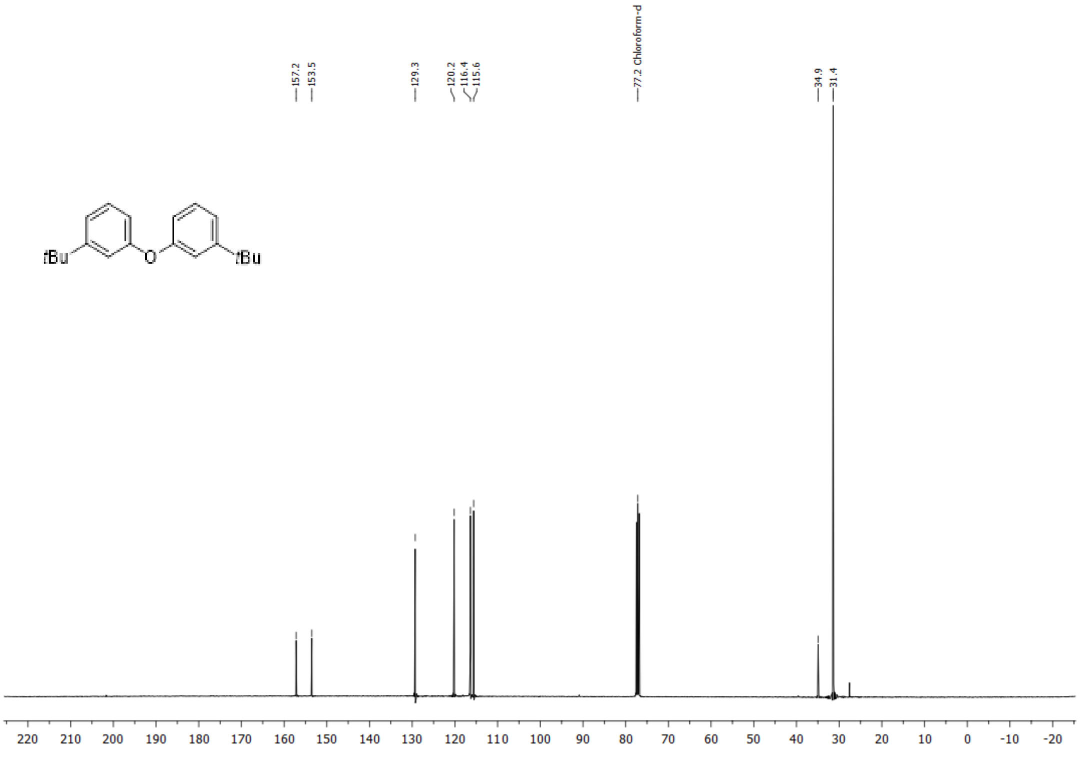

3,6-Di-*tert*-butyl-9-mesitylxanthylium tetrafluoroborate (S10)

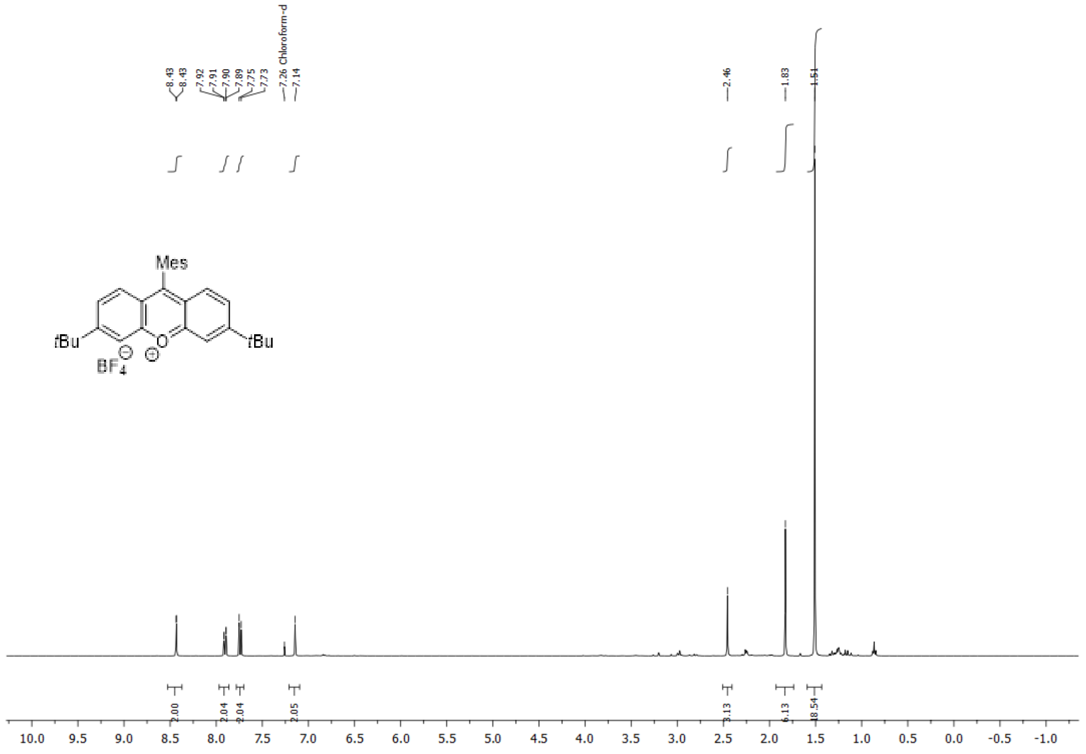

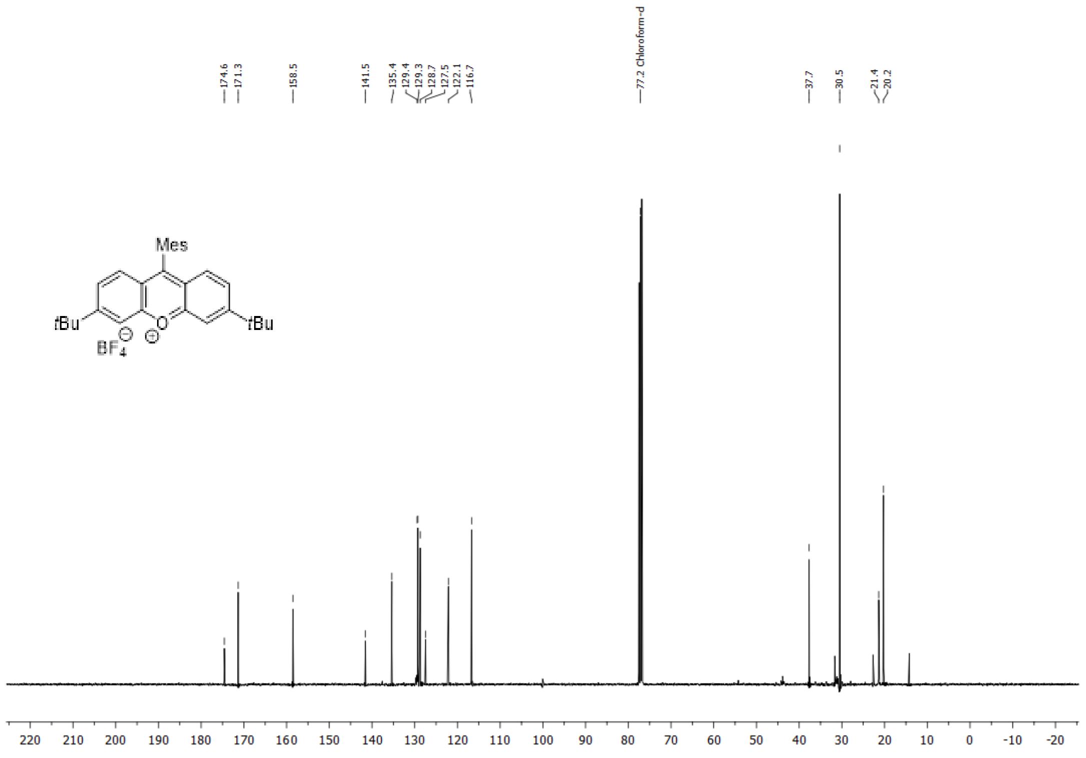

3,6-Di-*tert*-butyl-9-mesityl-10-phenylacridin-10-ium tetrafluoroborate (10)

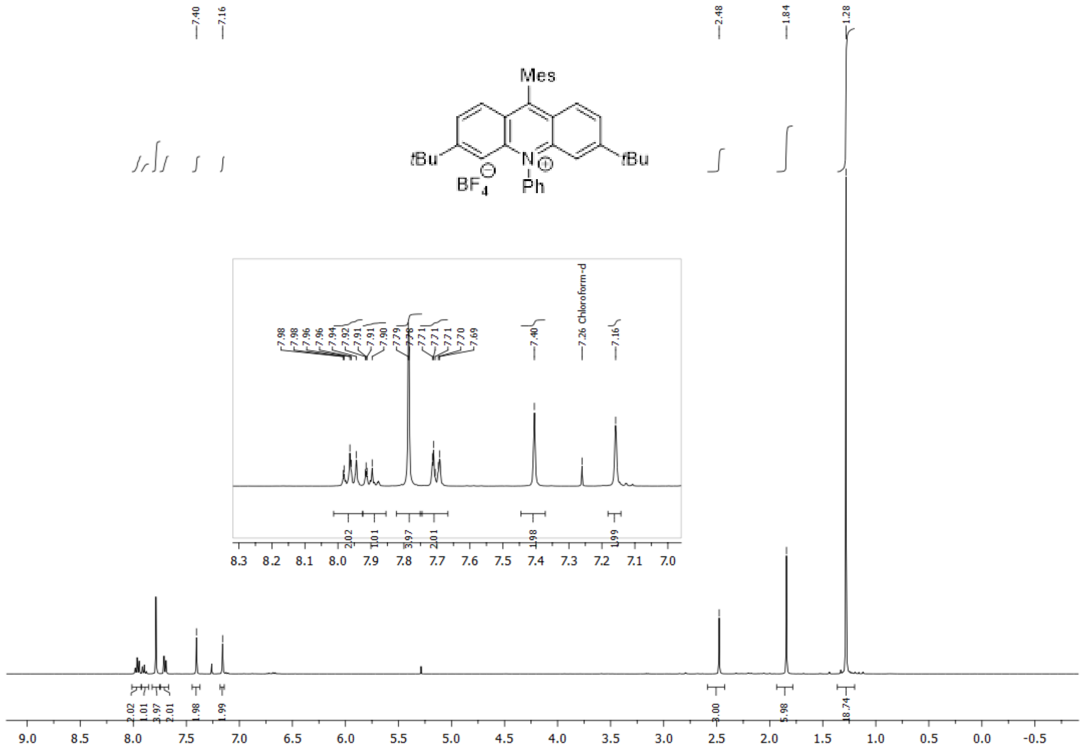

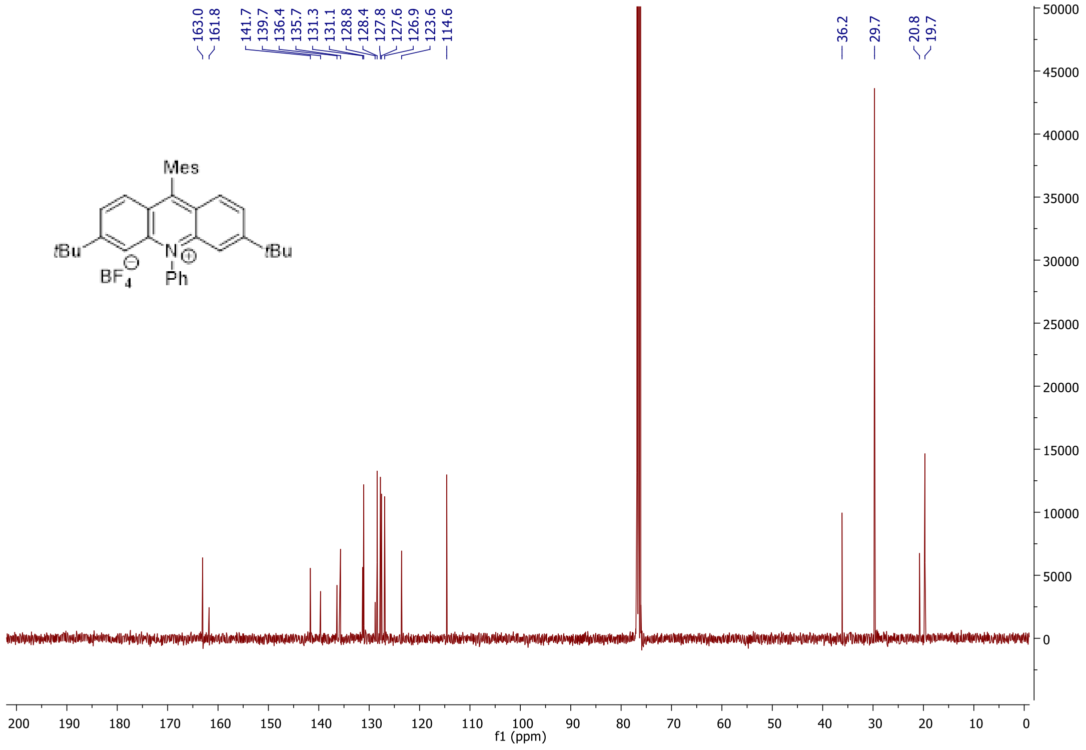

2-Methylene-1,3-diphenylpropane-1,3-dione (S11)

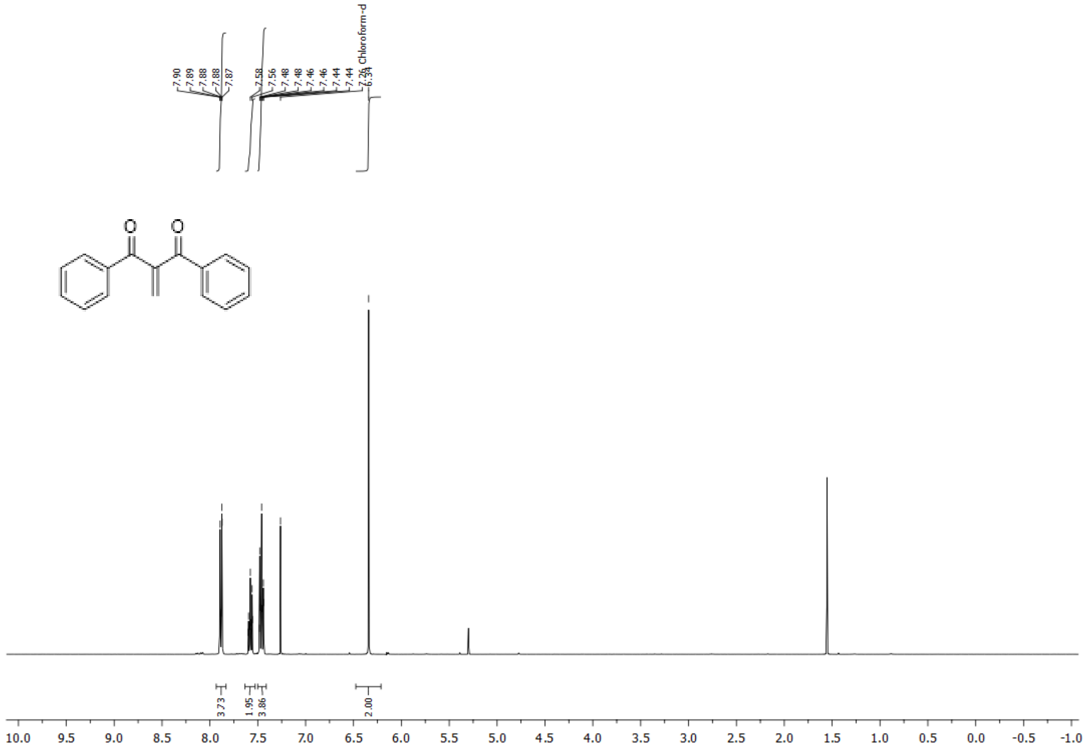

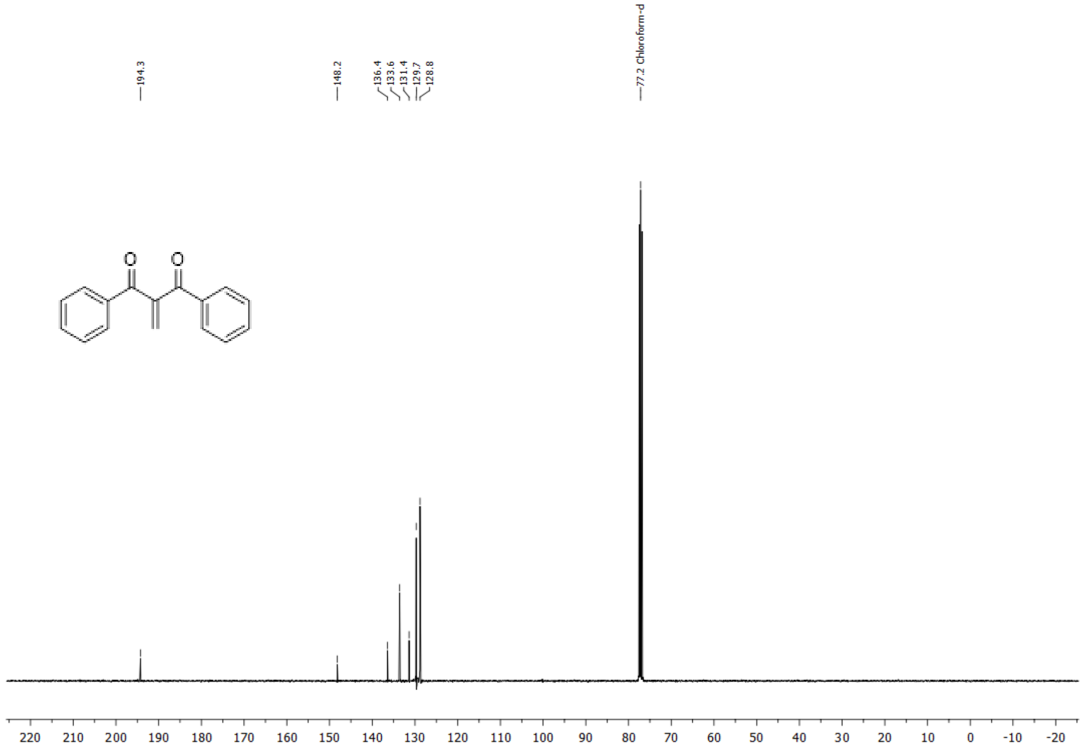

Tetraethyl ethene-1,1-diylbis(phosphonate) (S12)

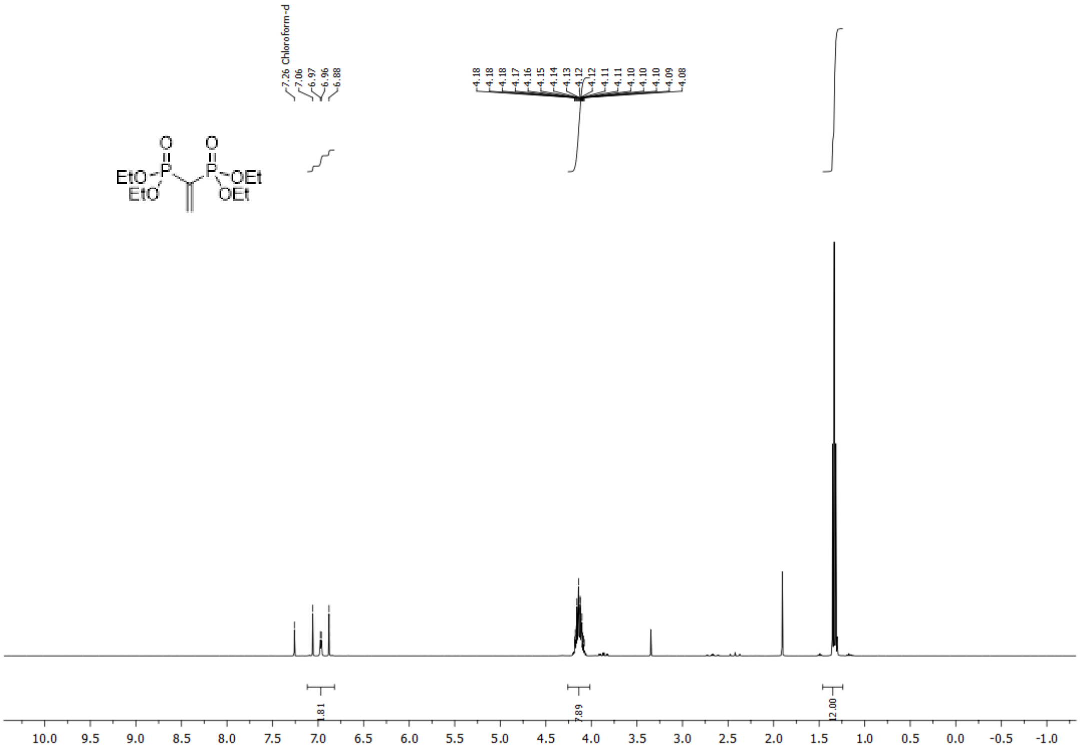

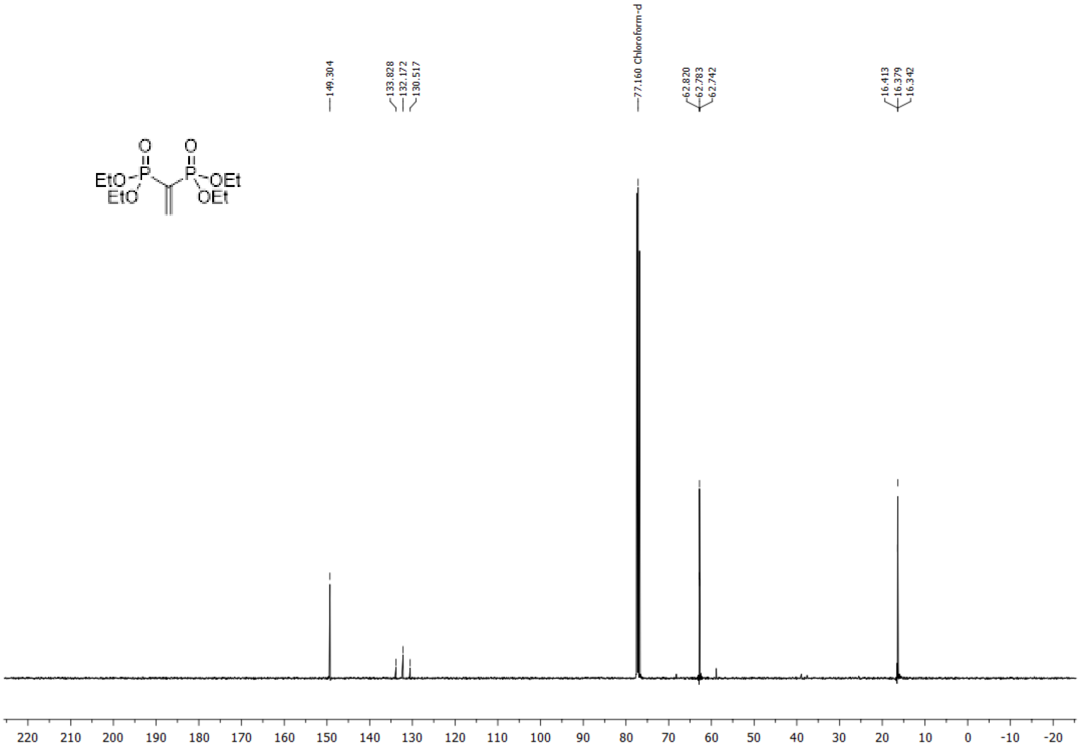

4-Methylbenzylidenemalononitrile (S13)

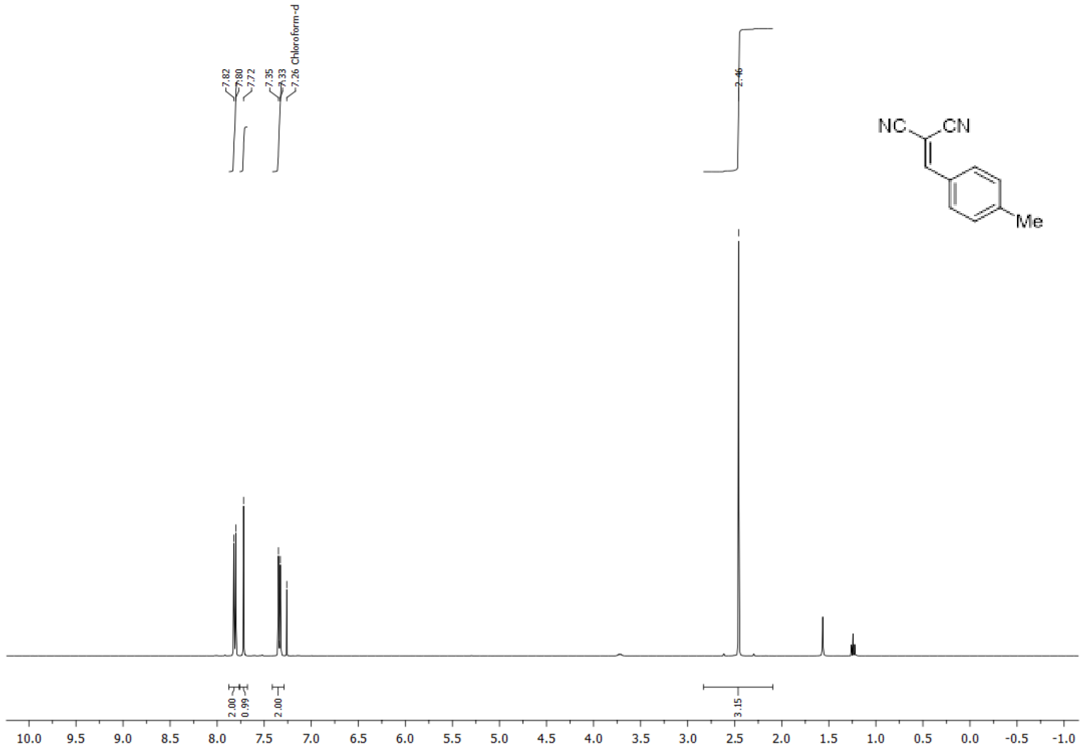

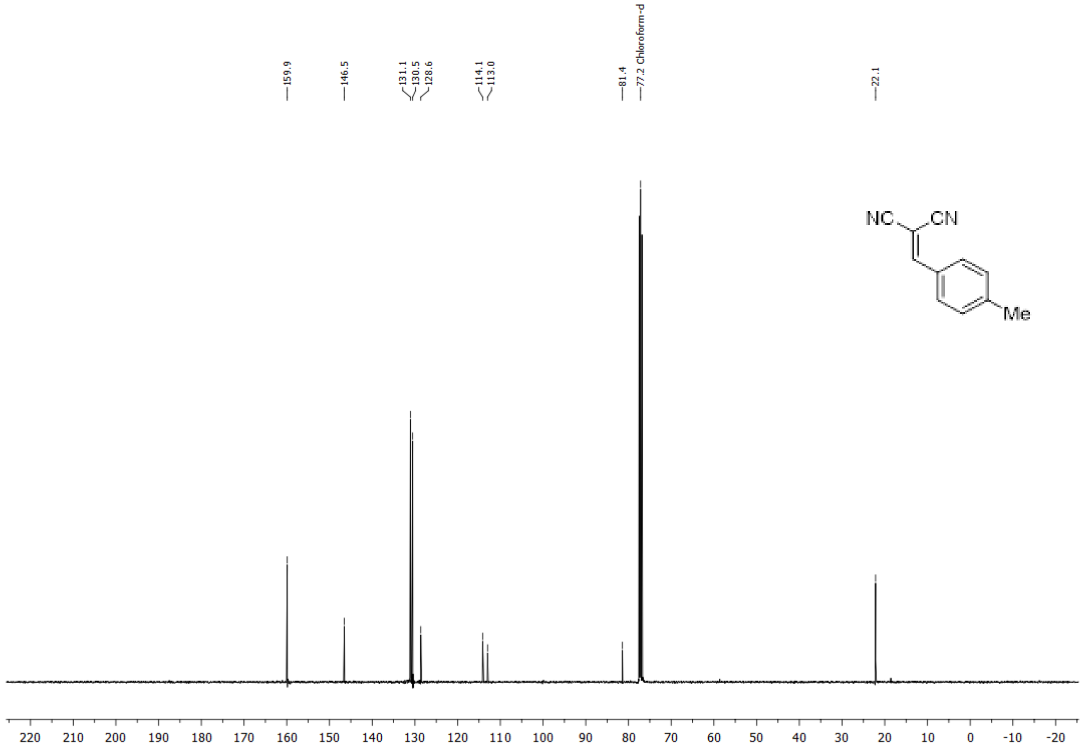

4-Fluorobenzylidenemalononitrile (S14)

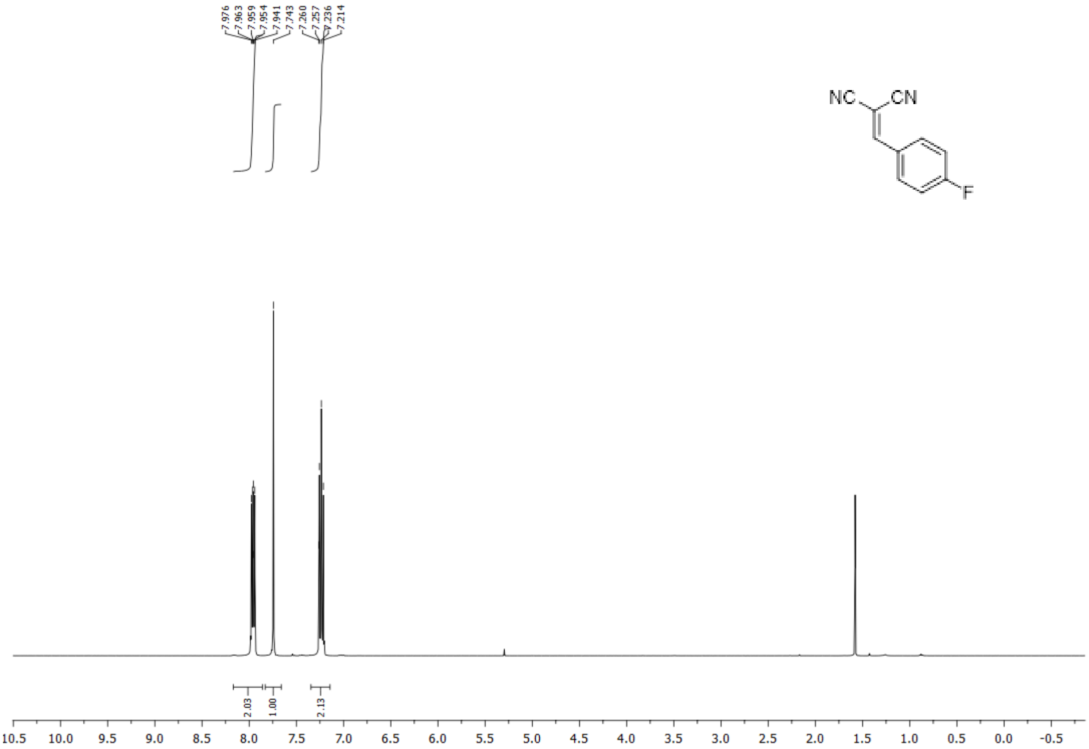

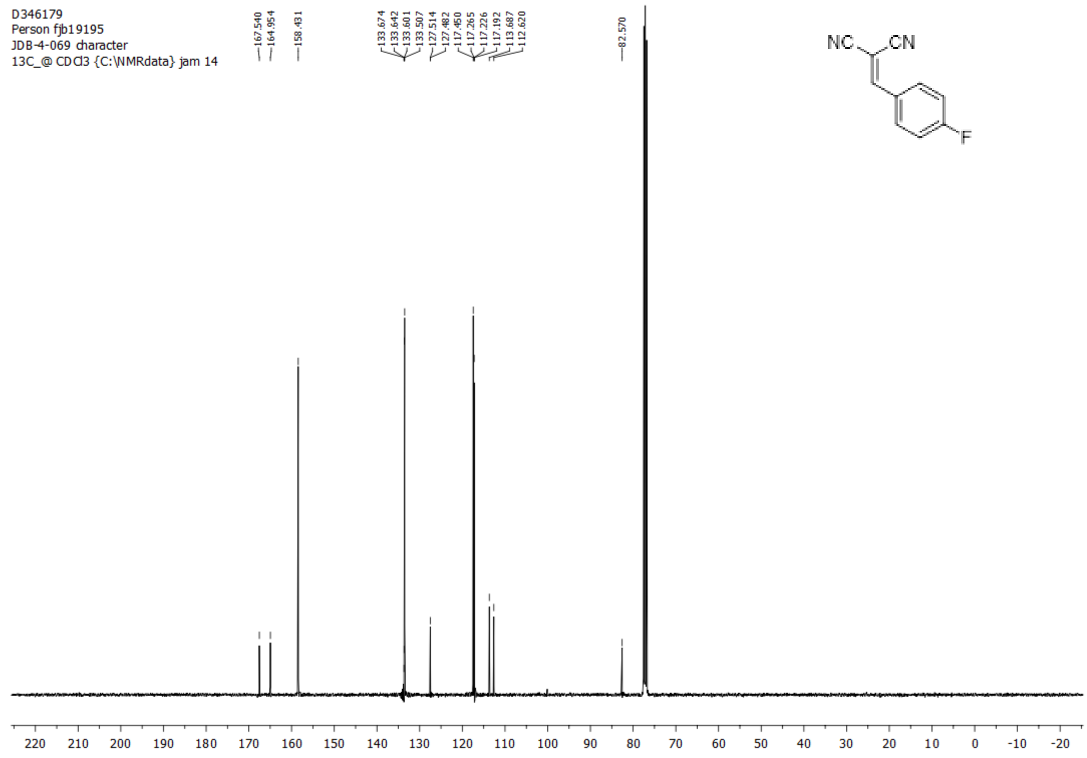

4-Chlorobenzylidenemalononitrile (S15)

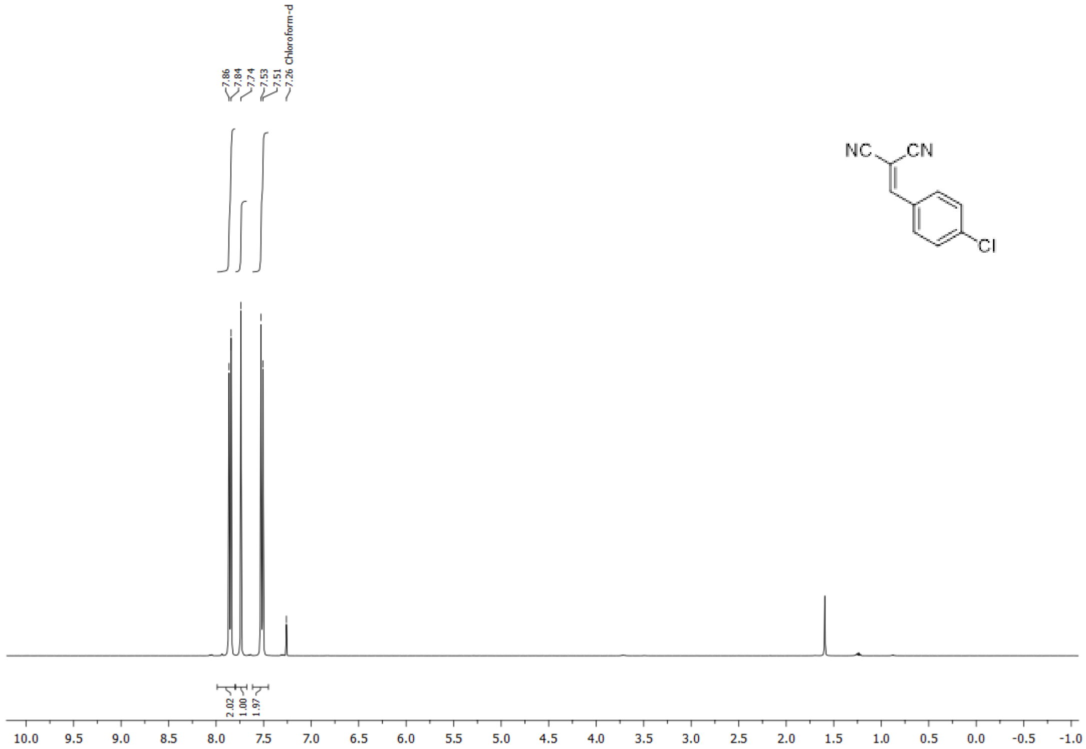

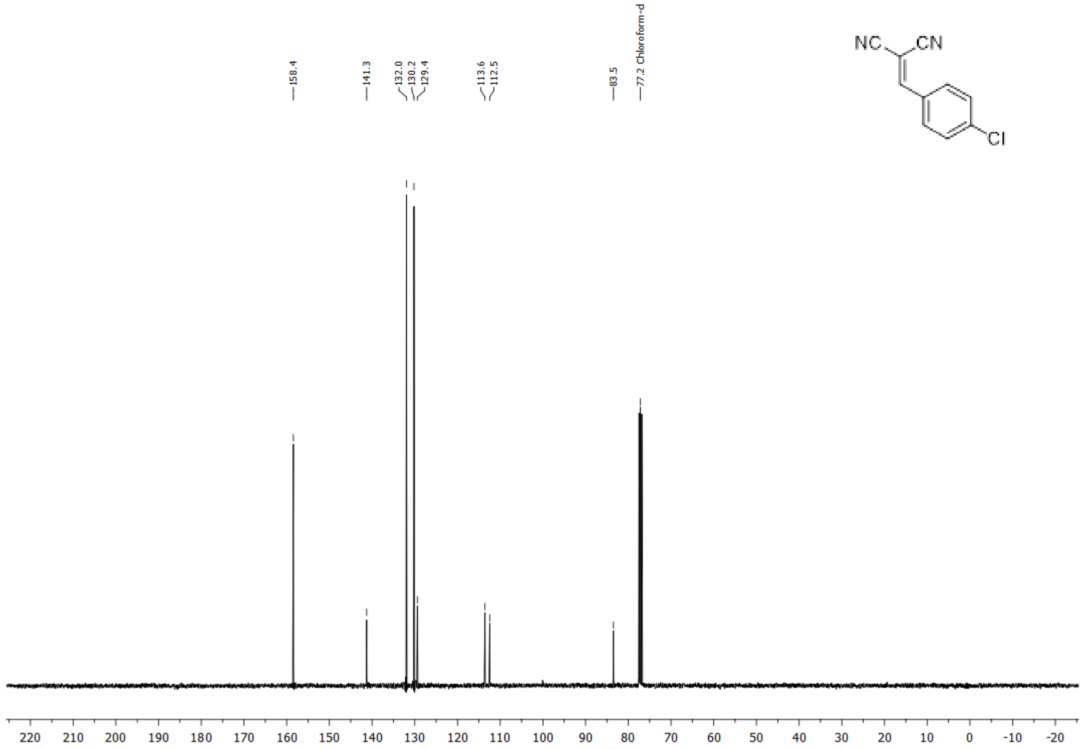

4-Bromobenzylidenemalononitrile (S16)

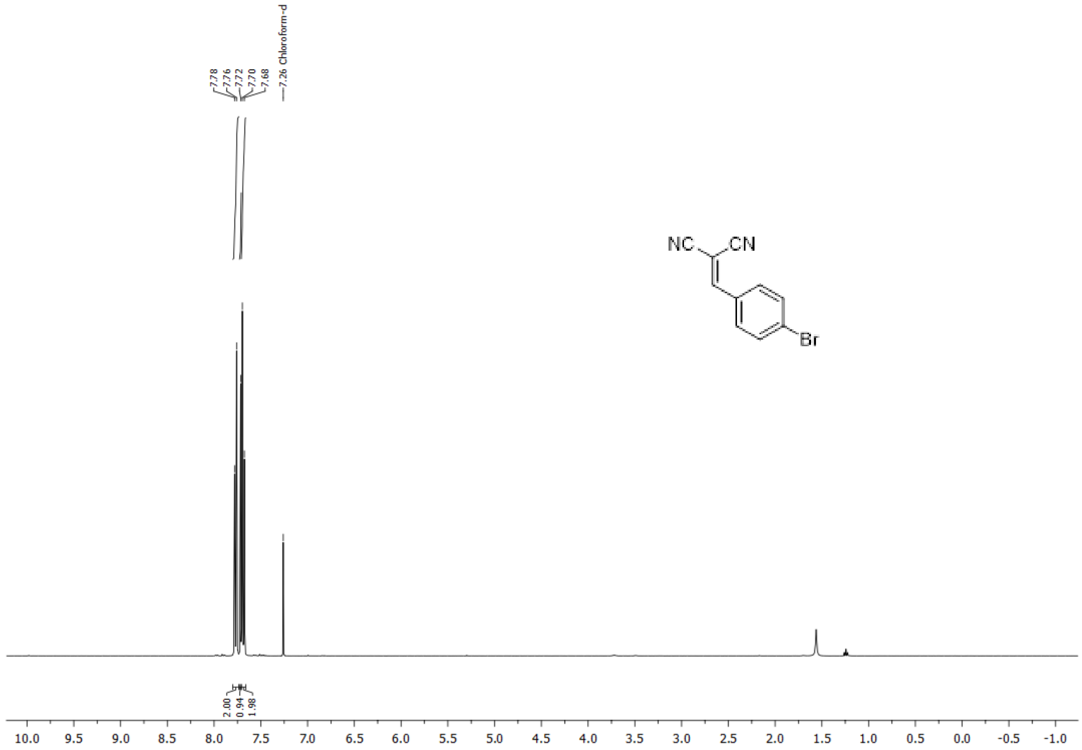

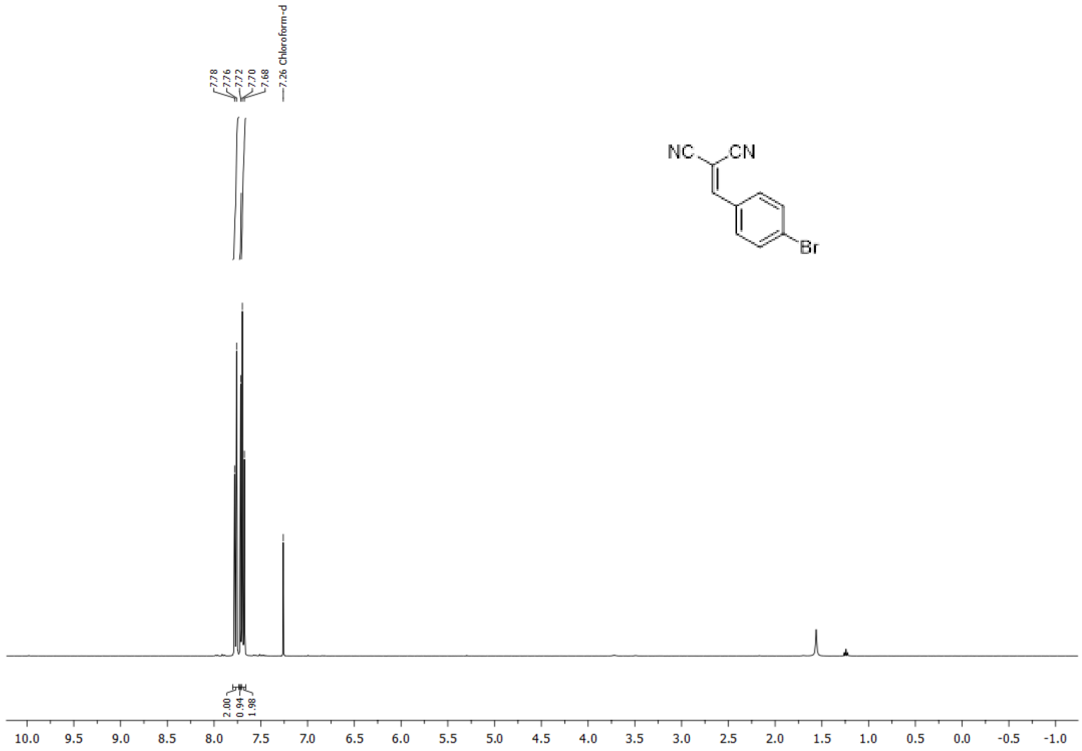

4-Iodobenzylidenemalononitrile (S17)

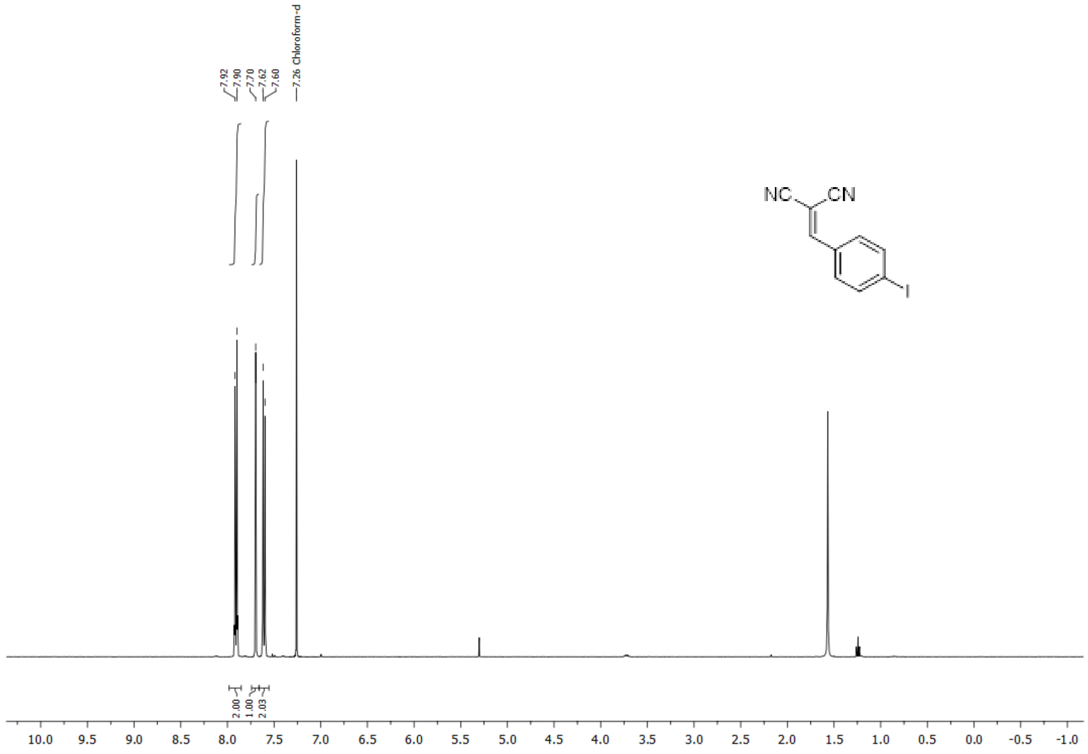

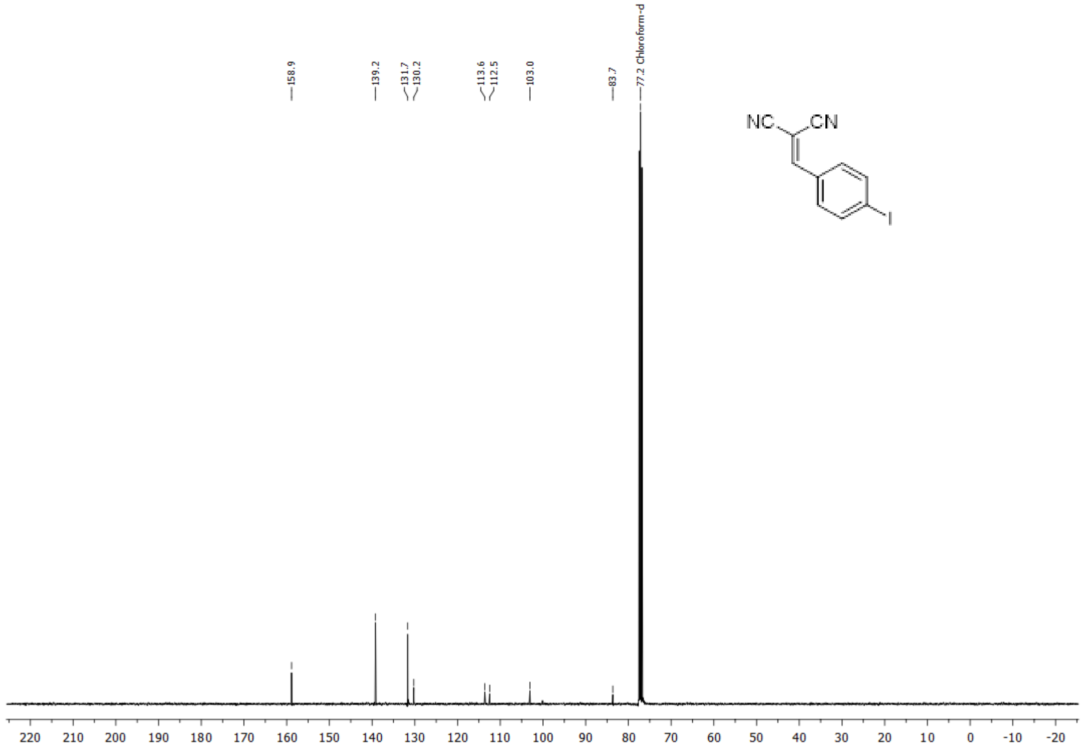

4-Methoxybenzylidenemalononitrile (S18)

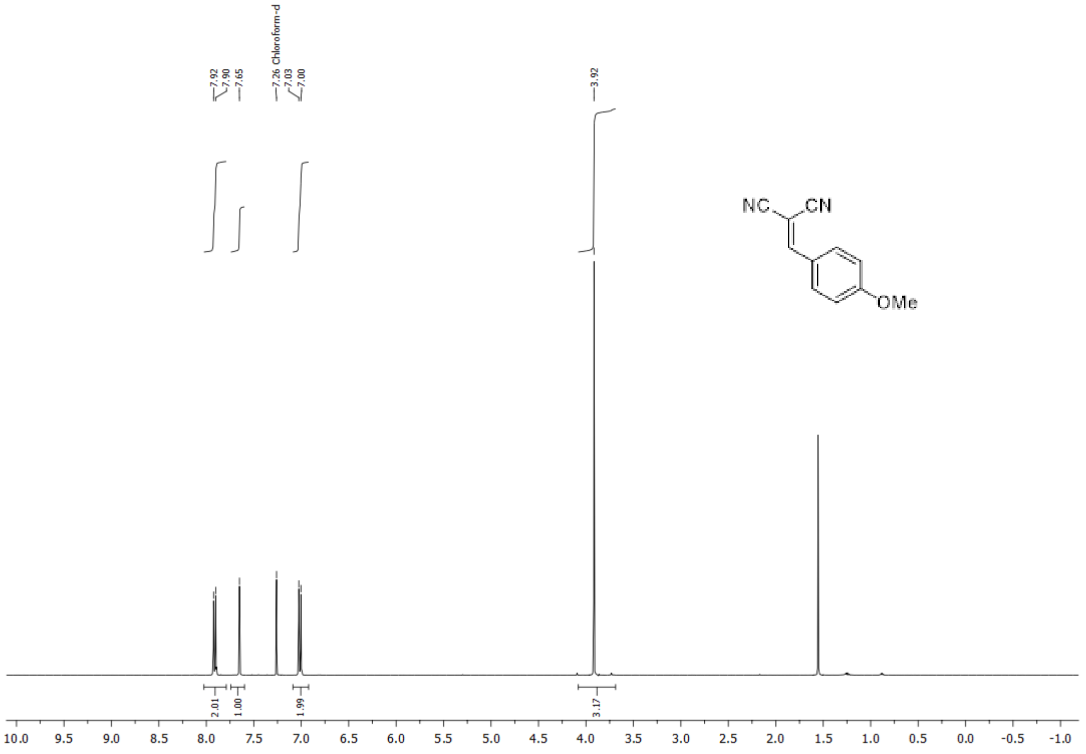

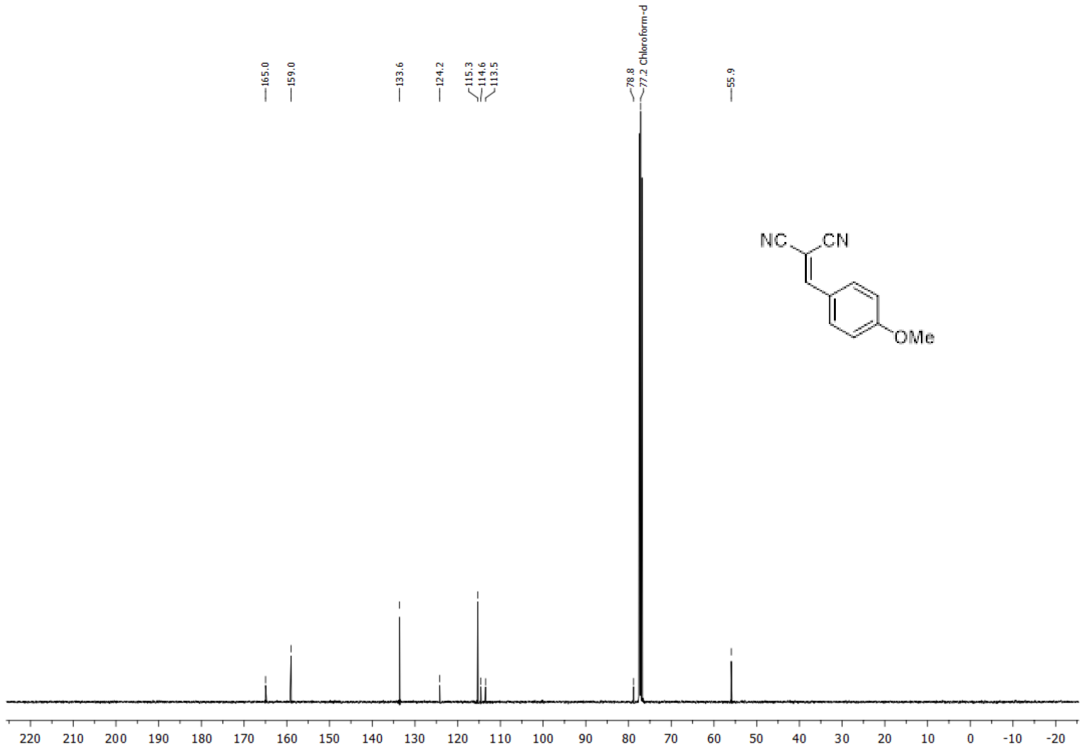

4-Nitrobenzylidenemalononitrile (S19)

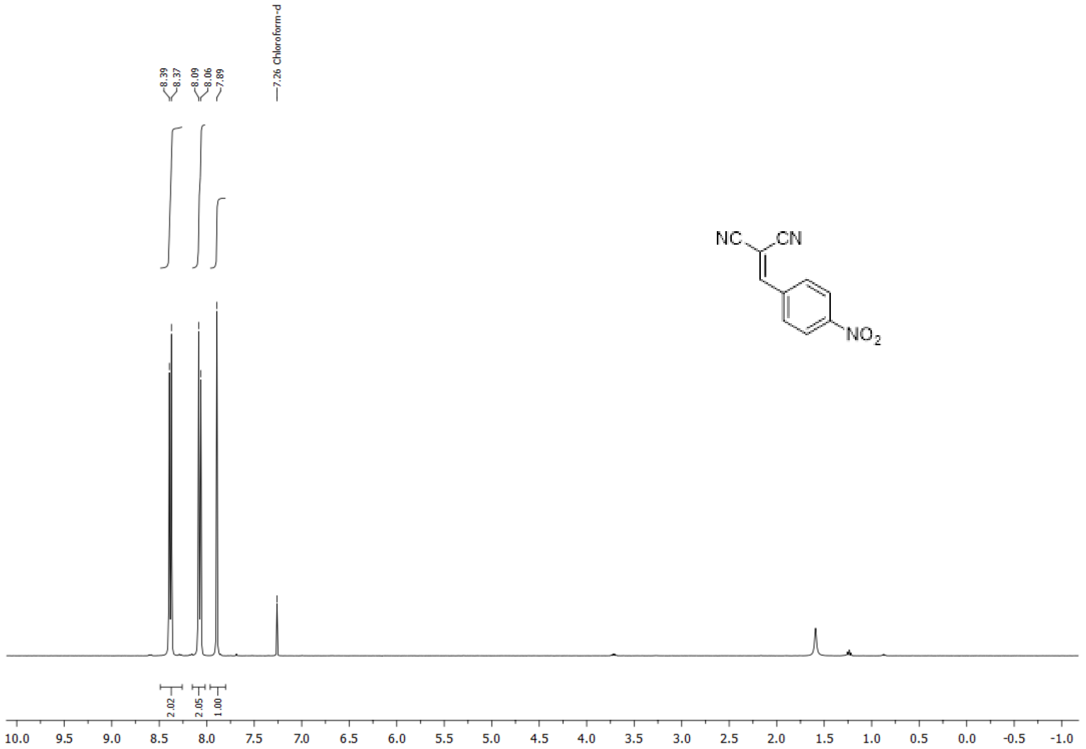

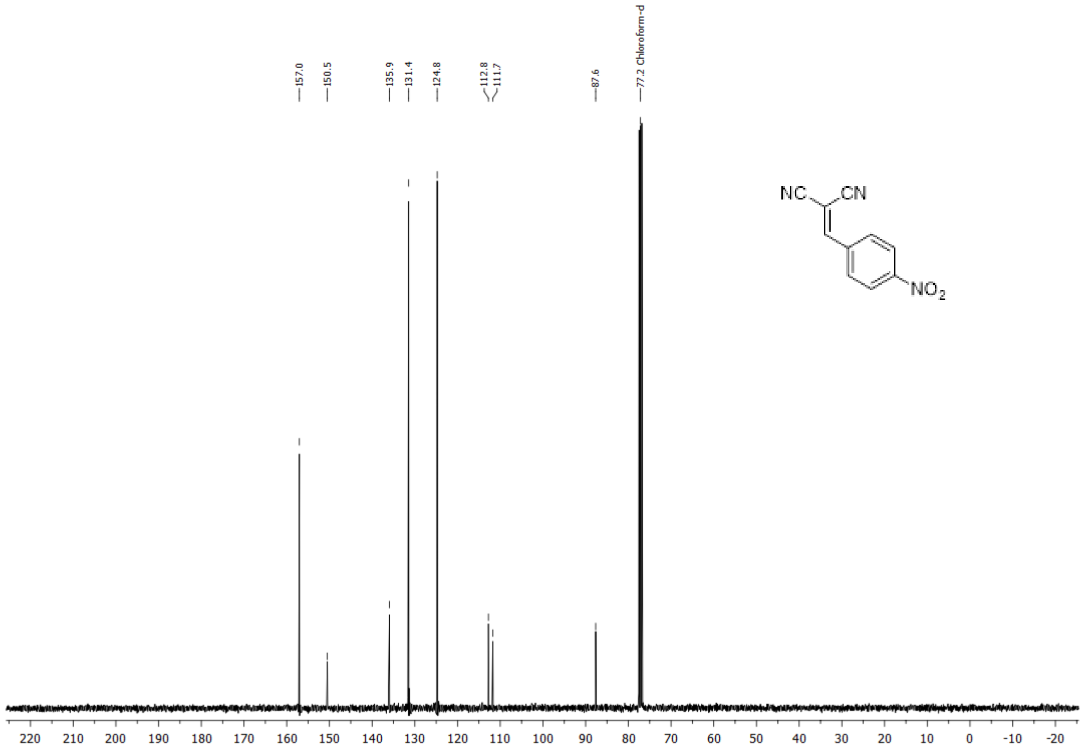

2-(4′-Methoxyphenyl)-2-methyI-1,3-dioxolane (S20)

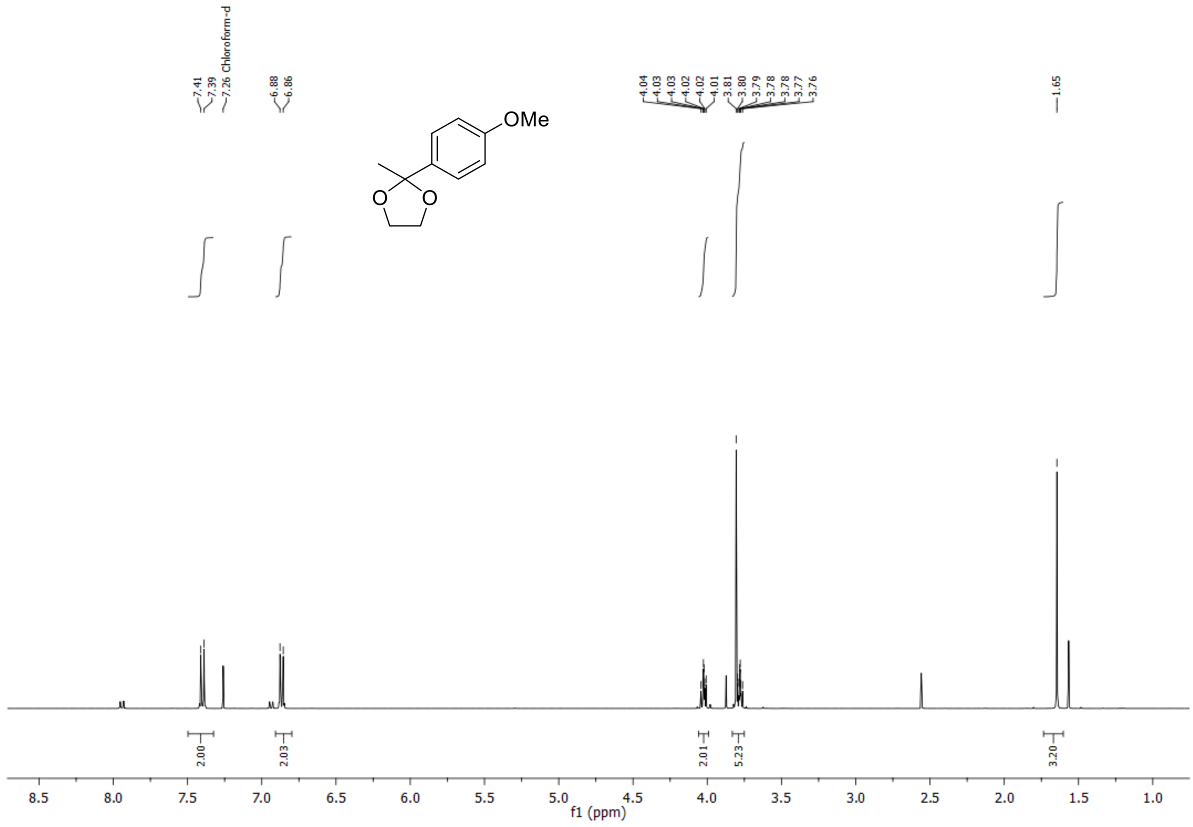

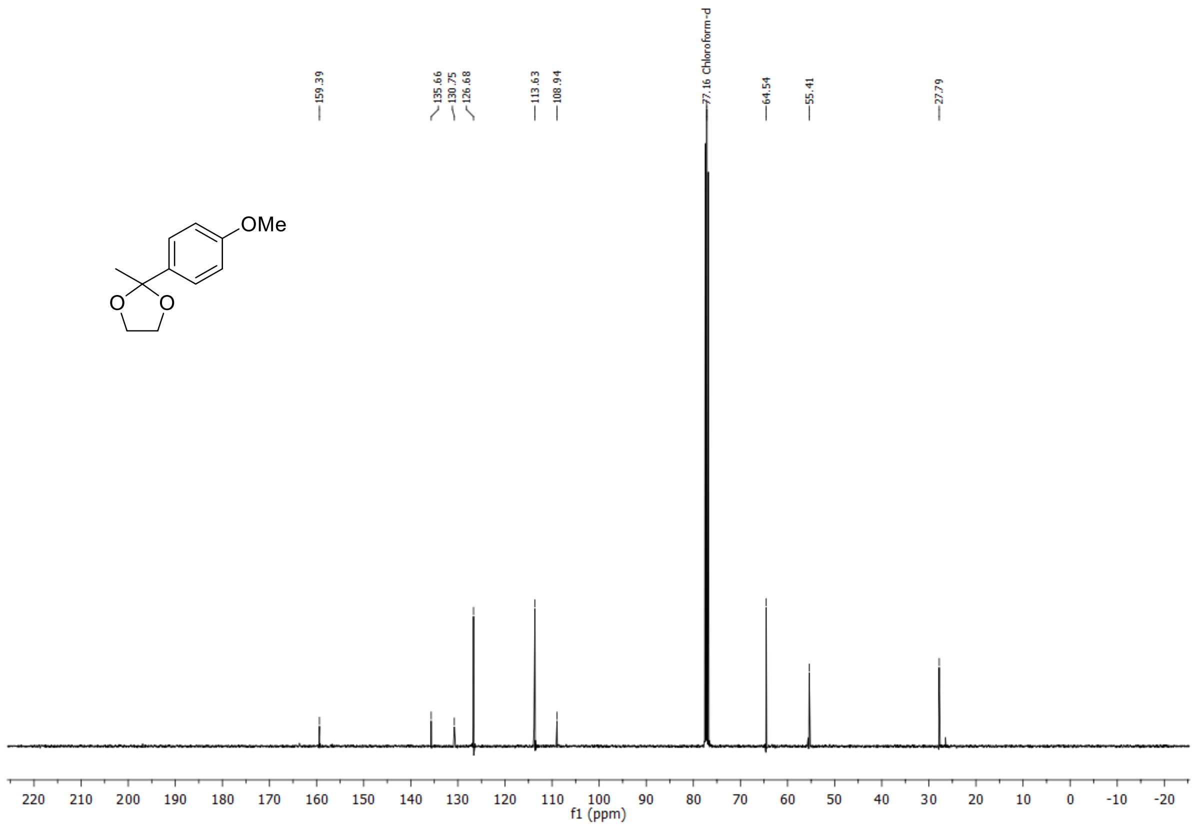

Hexyl phenyl ether (S21)

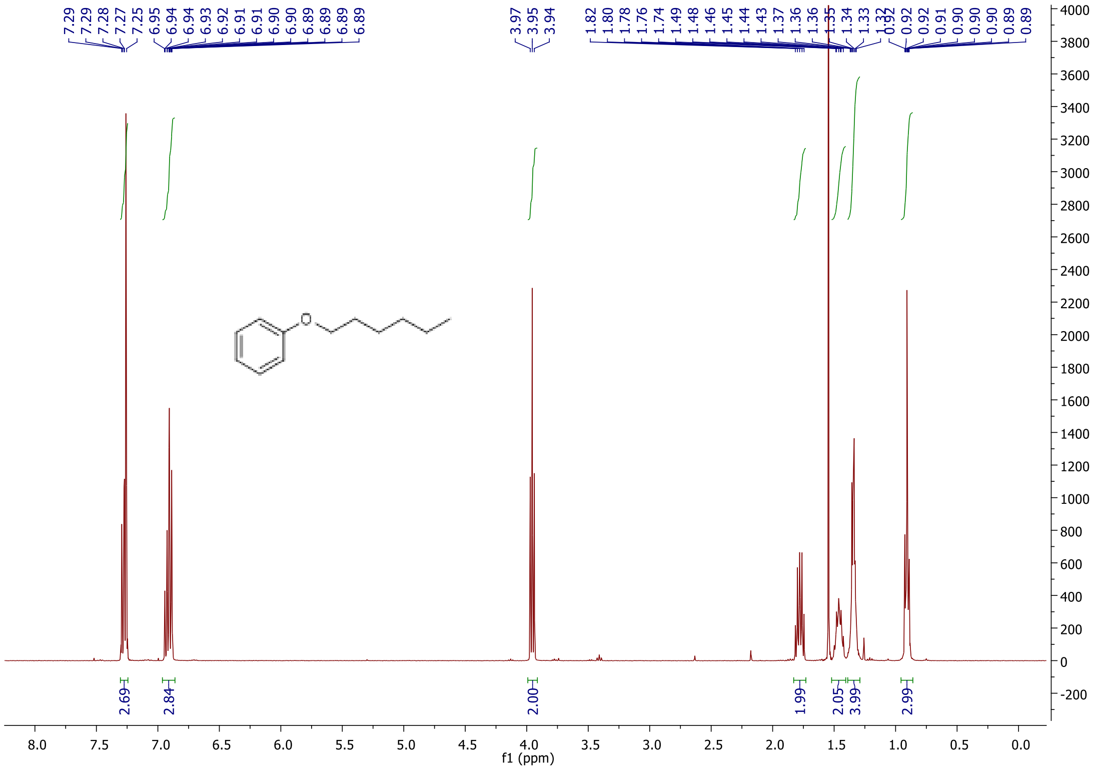

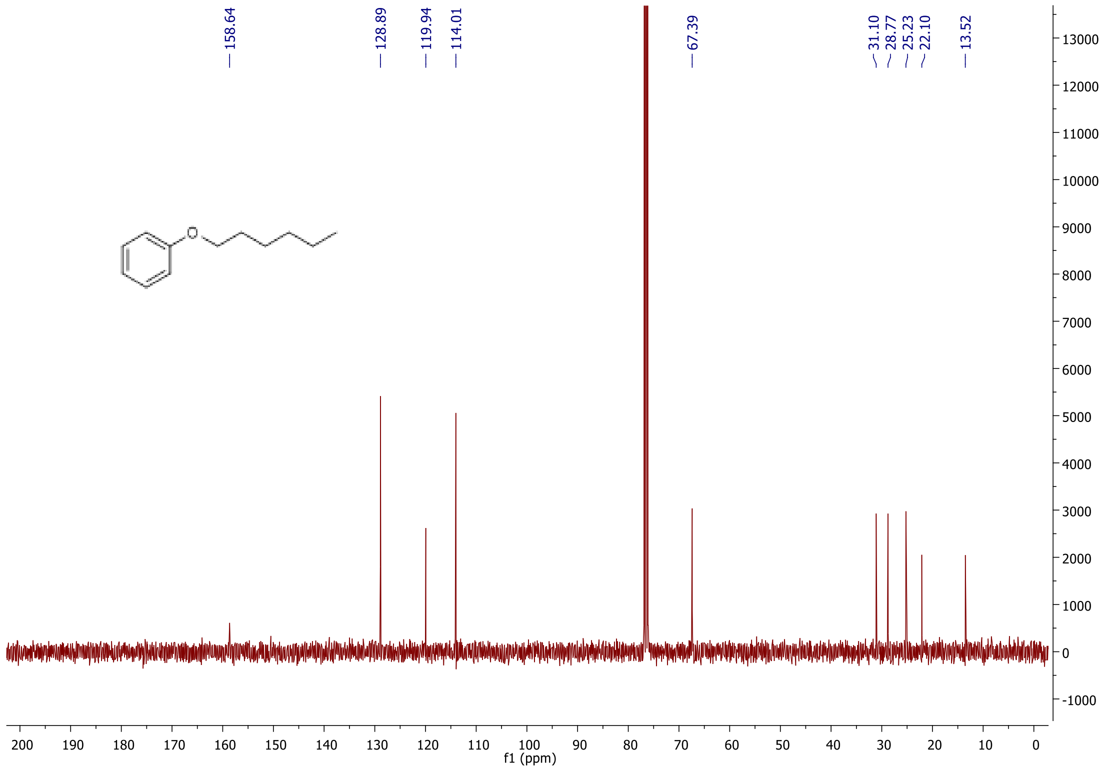

3-(1′,3′-Dioxoisoindolin-2′-yl)propyl phenyl ether (S22)

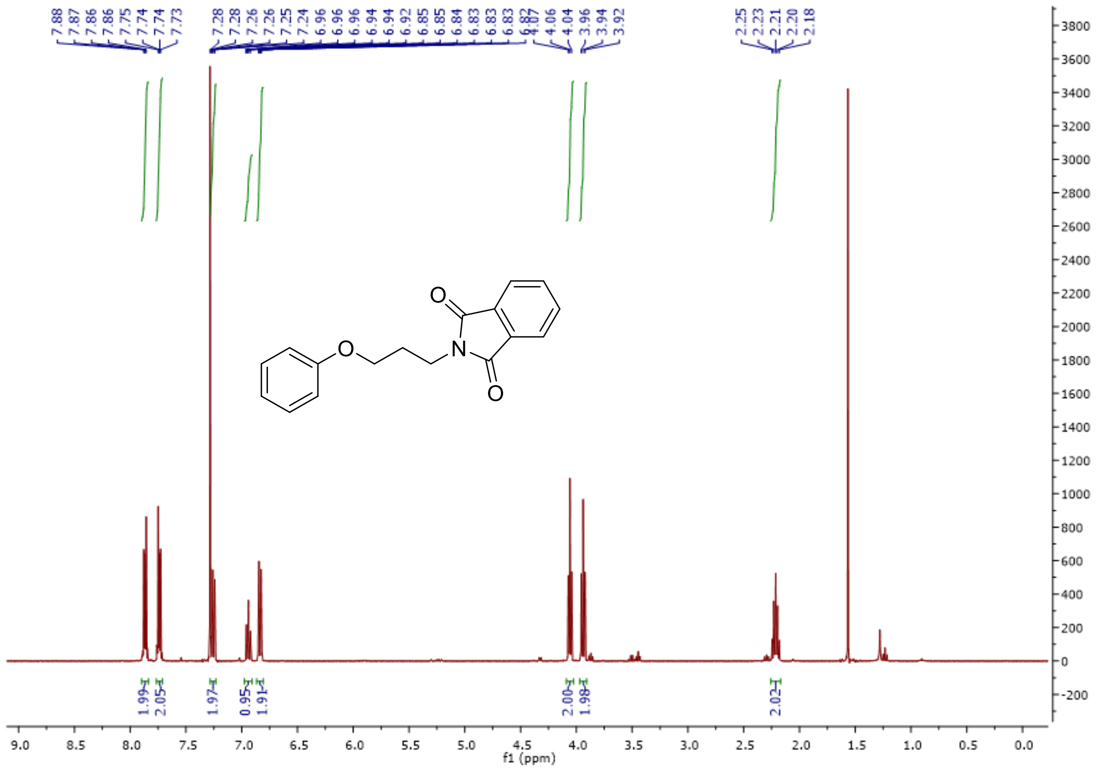

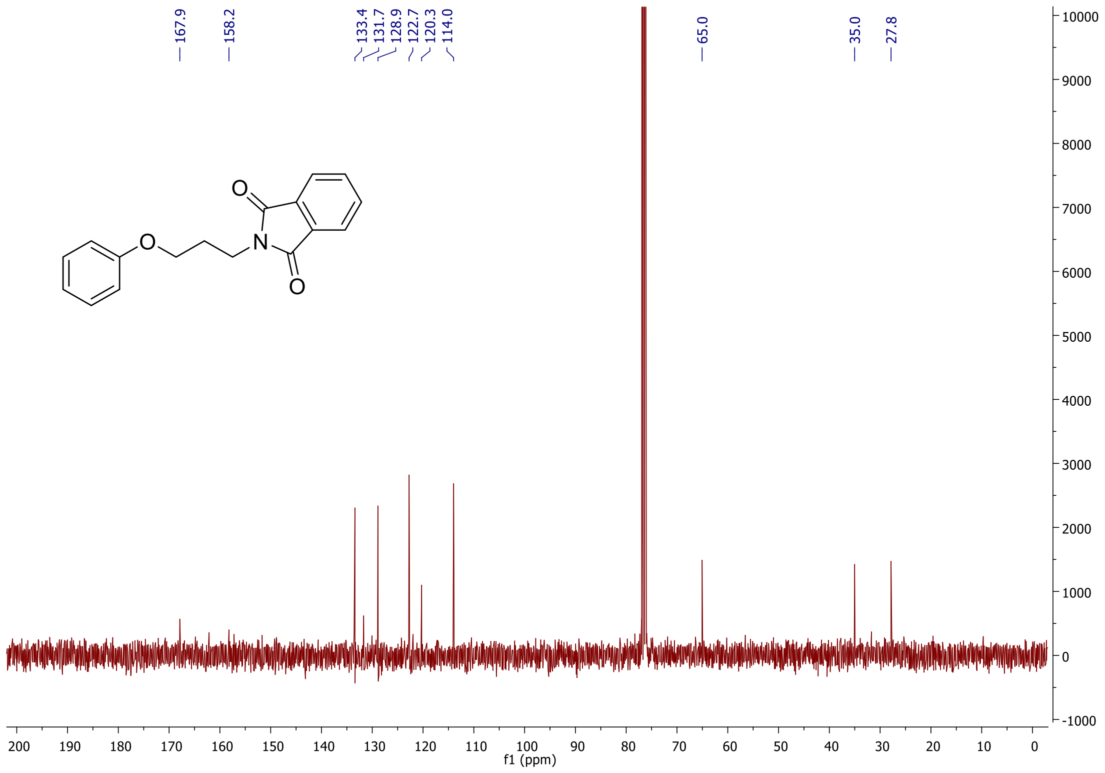

3-Phenoxypropyl acetate (S23)

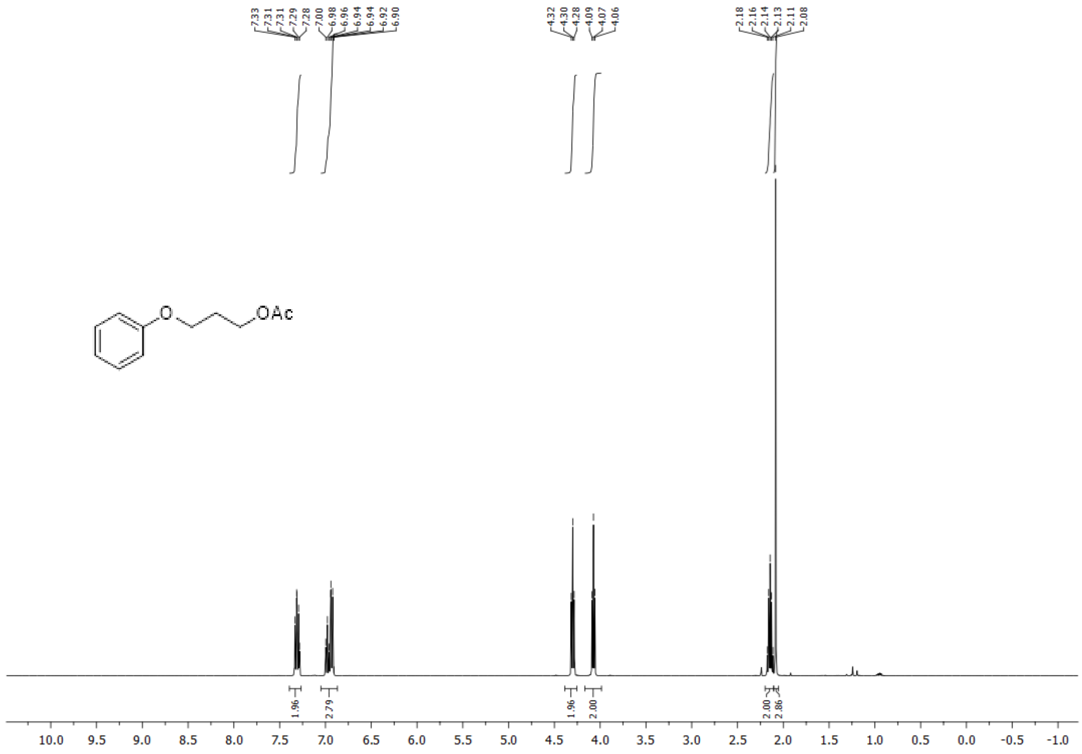

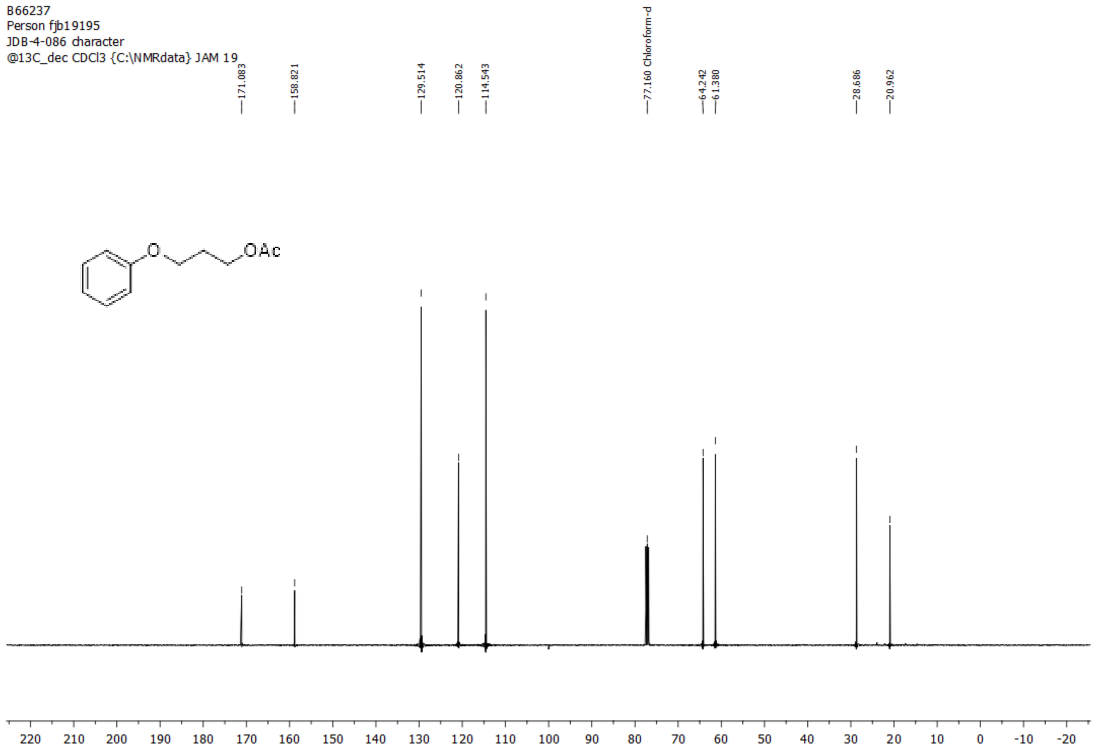

3-Phenoxypropyl benzoate (S24)

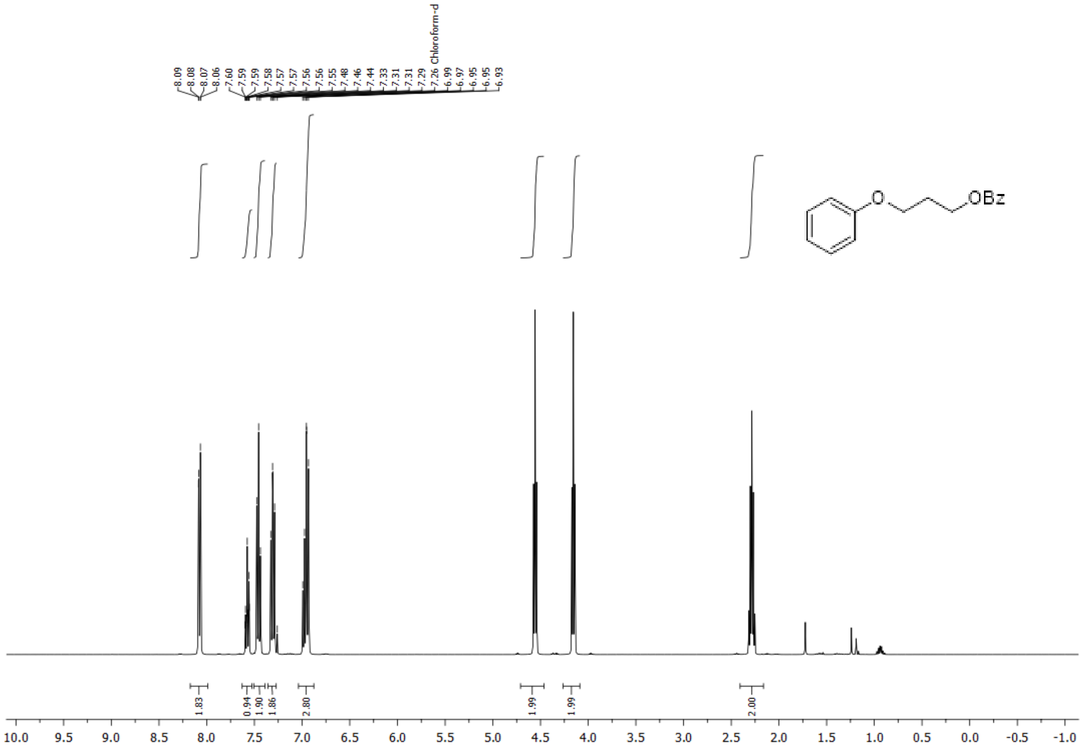

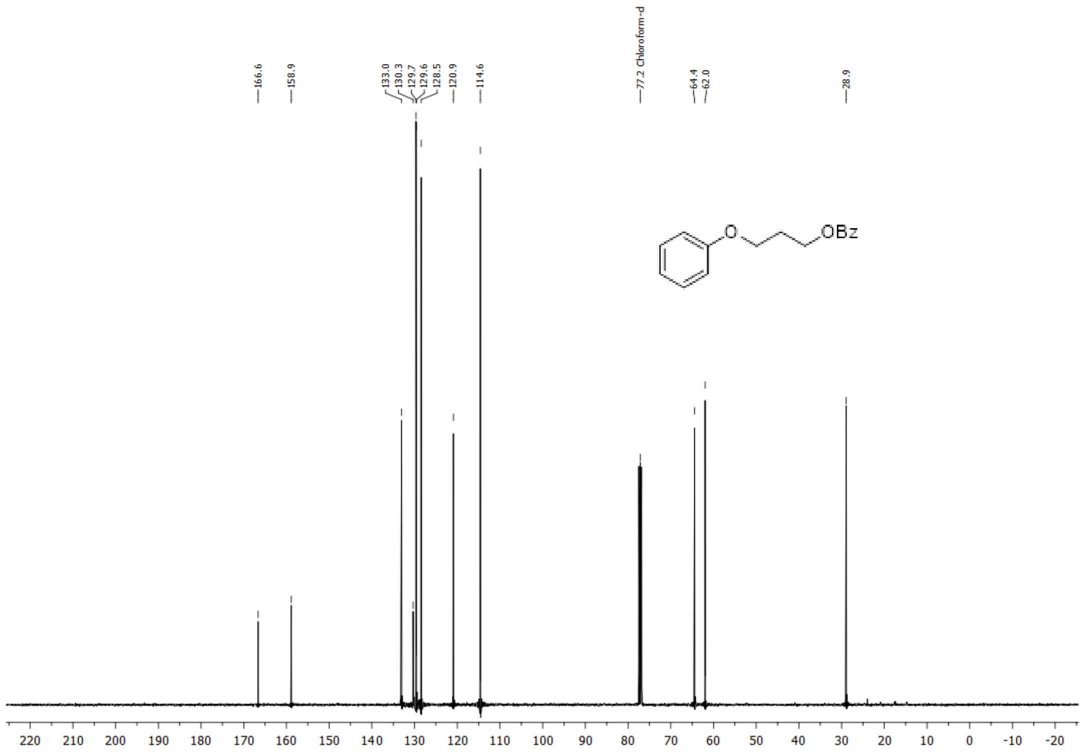

Isopropyl phenyl ether (S25)

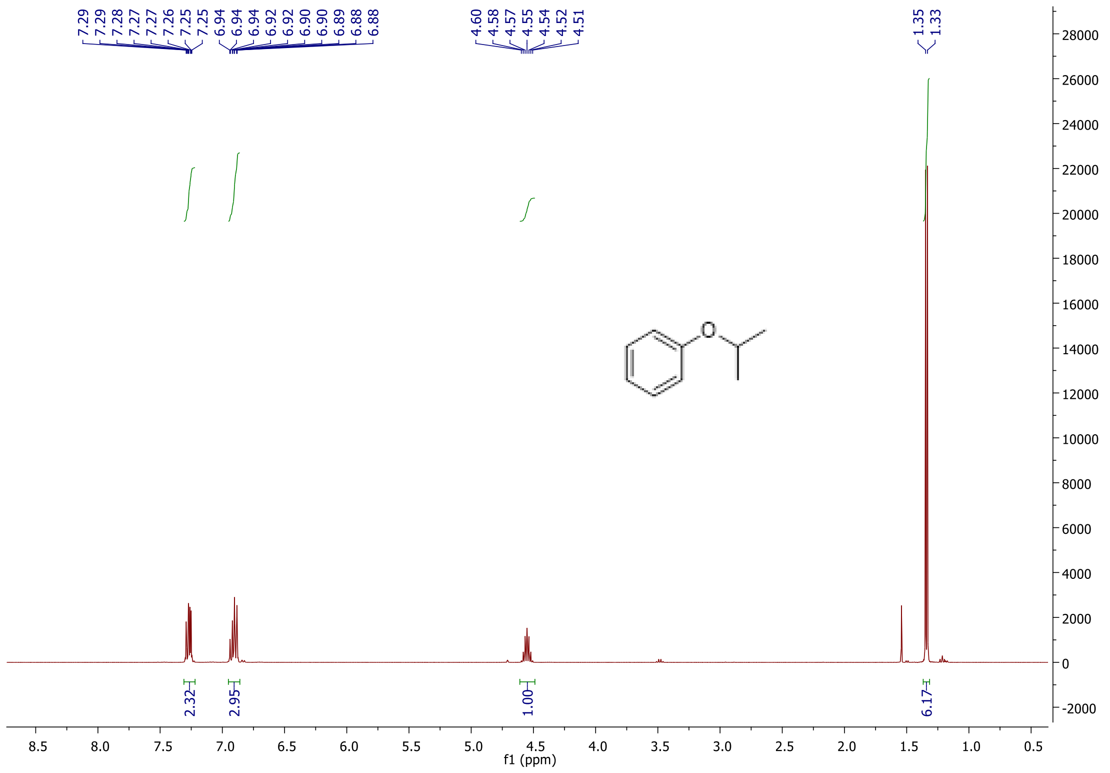

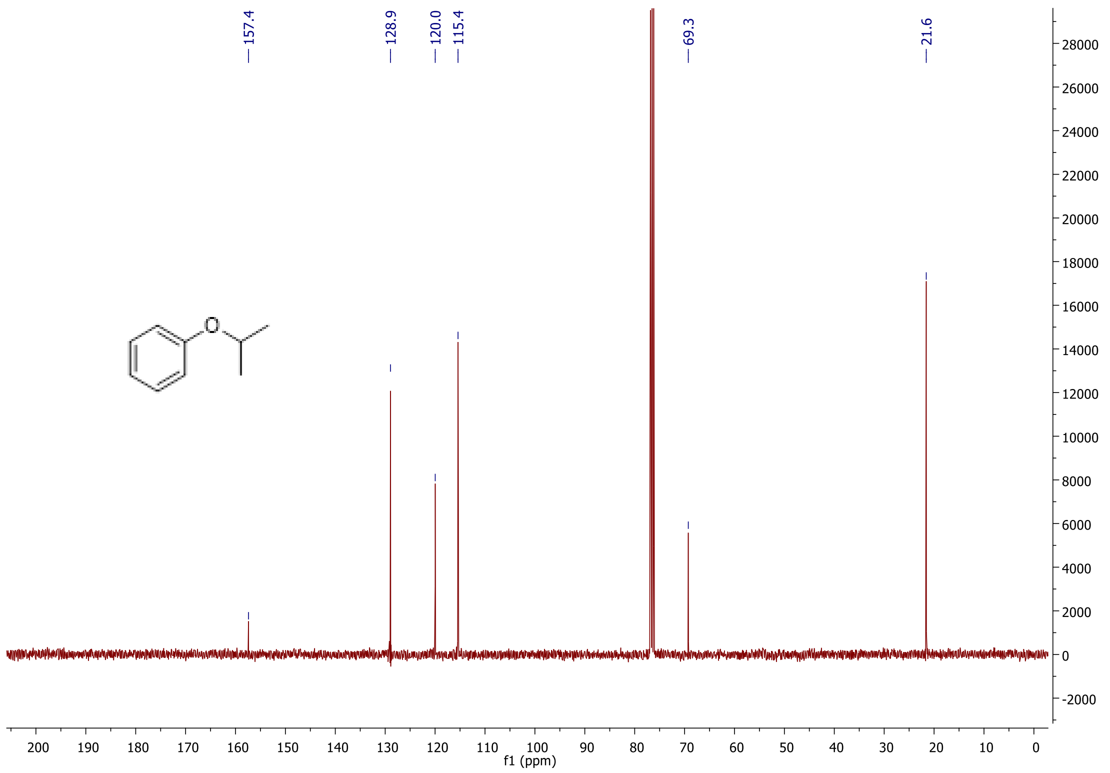

Cyclobutyl phenyl ether (S26)

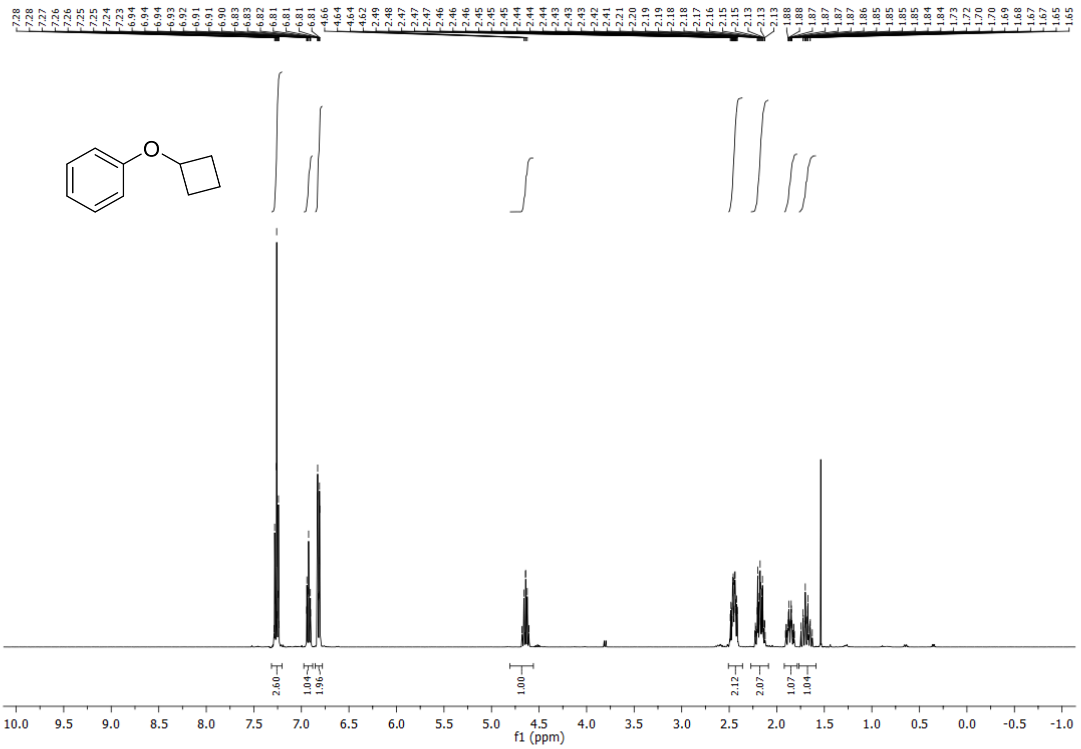

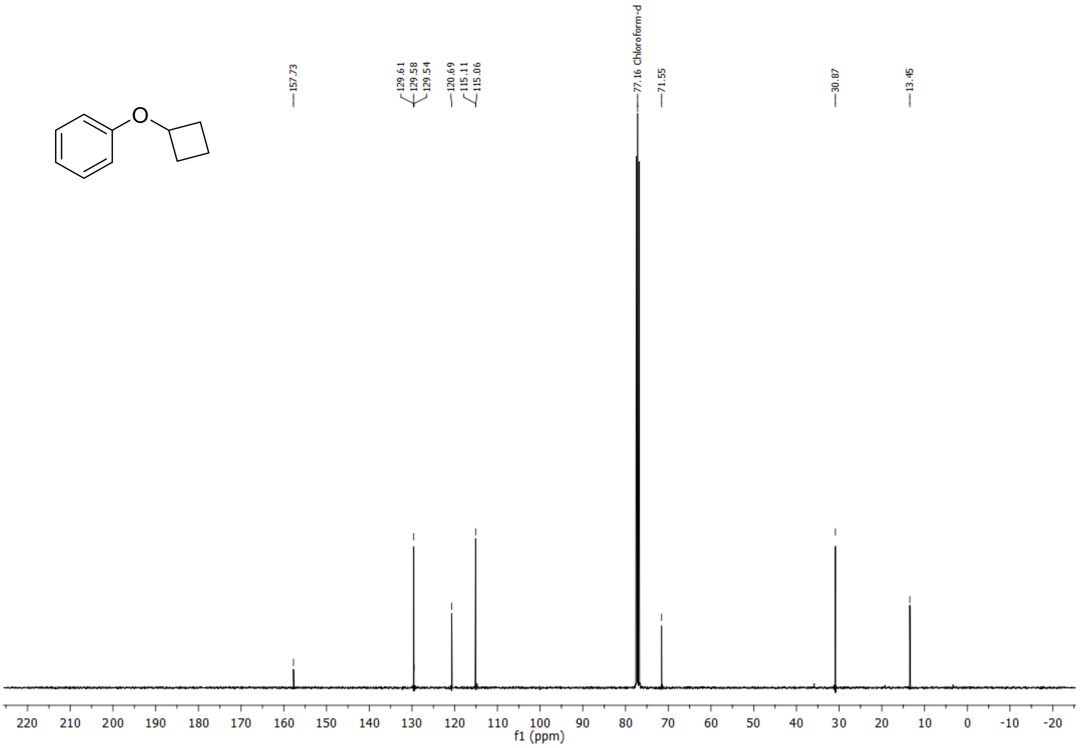

Cyclopentyl phenyl ether (S27)

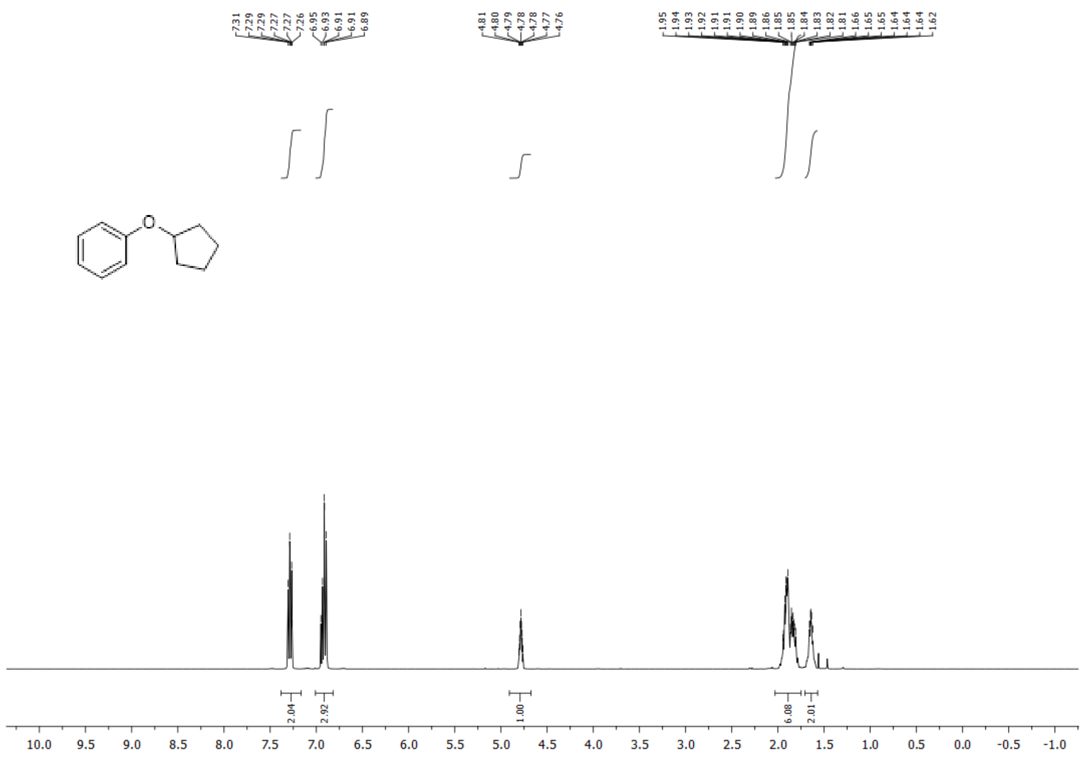

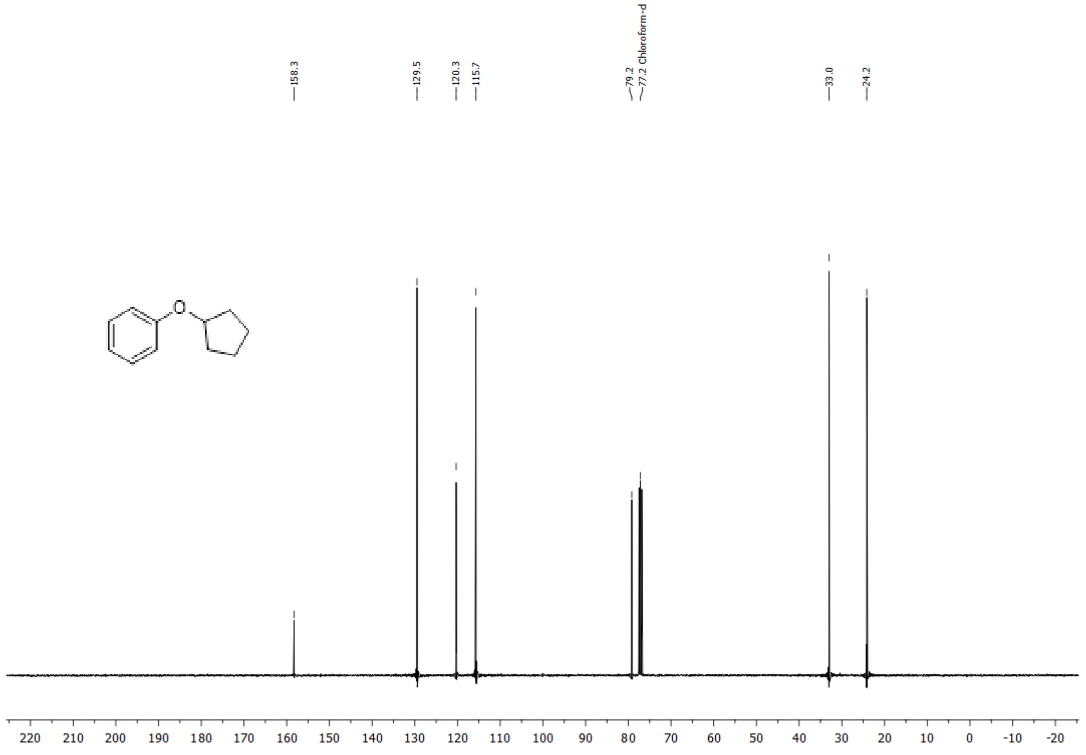

3-Phenoxypropylbenzene (6)

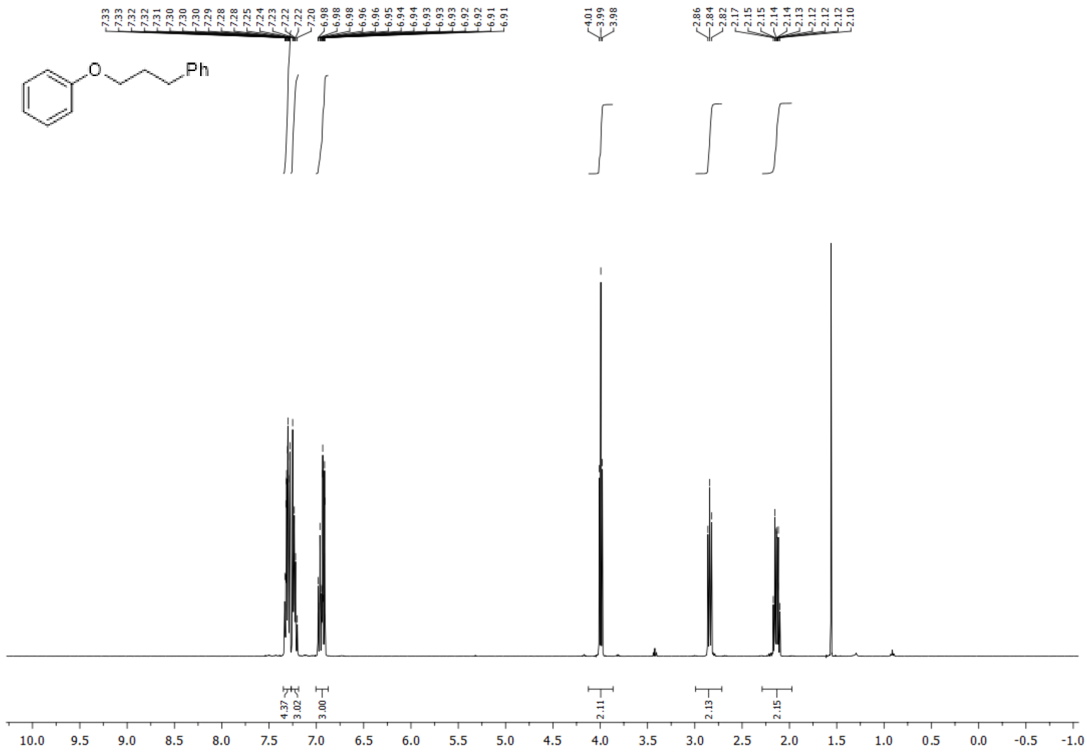

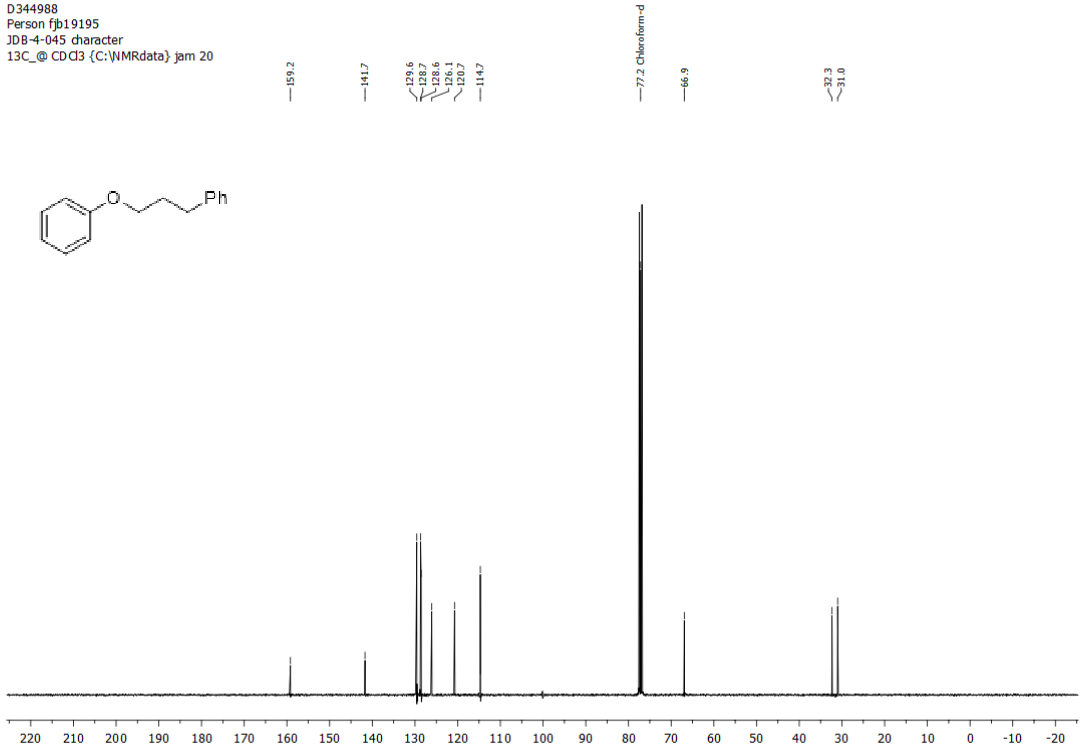

1-Methyl-4-(phenoxymethyl)benzene (S28)

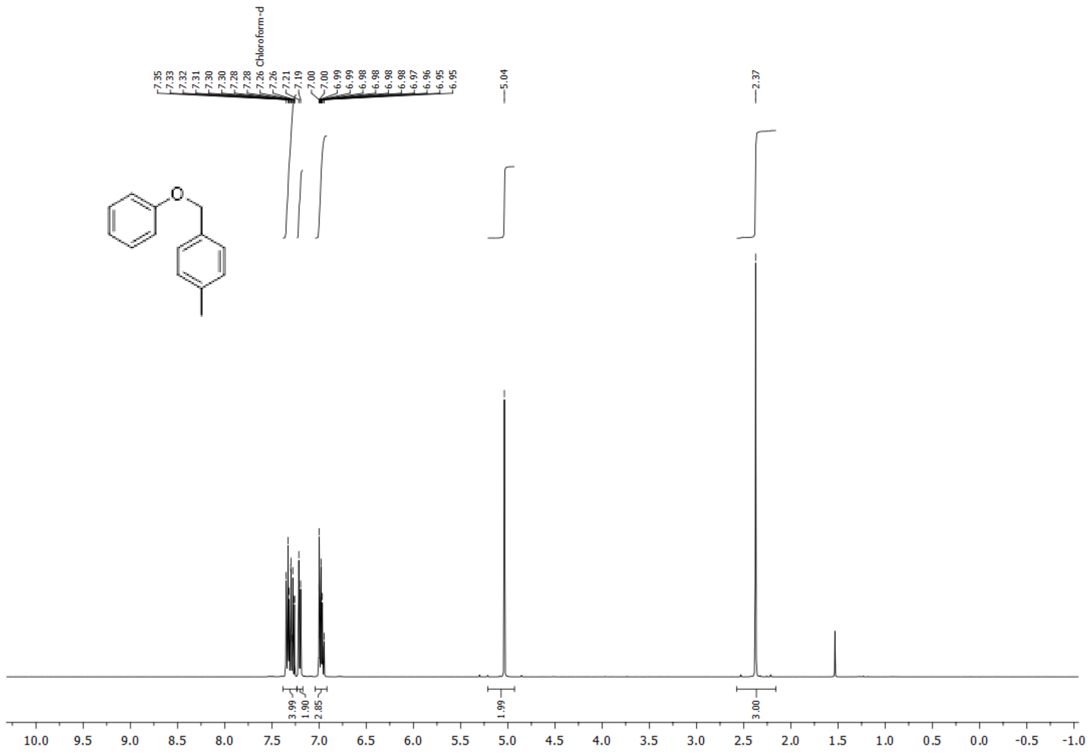

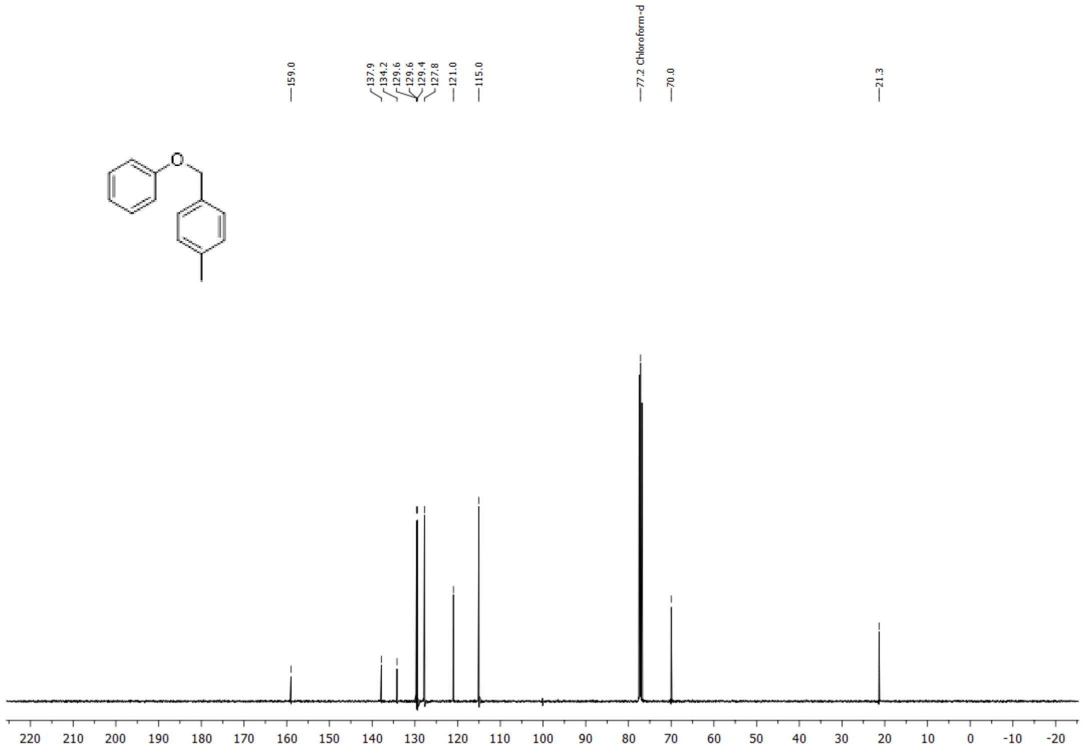

3-Methoxypropylbenzene (25)[19]

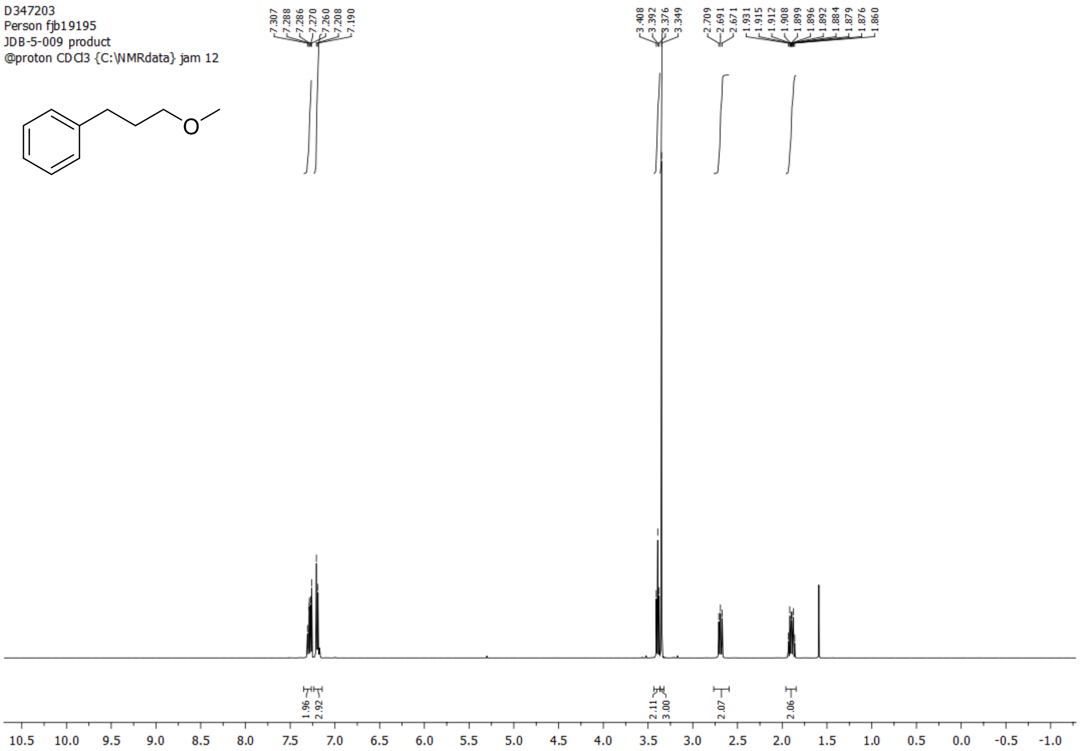

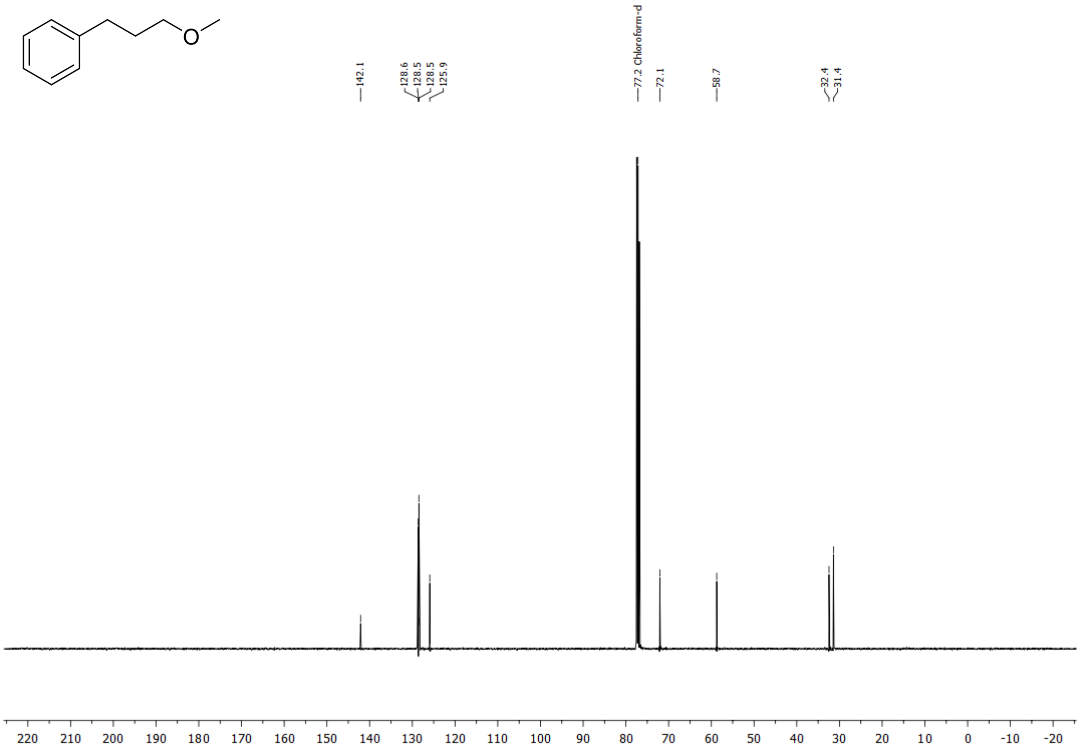

Dimethyl 2-(phenoxymethyl)succinate (20a)

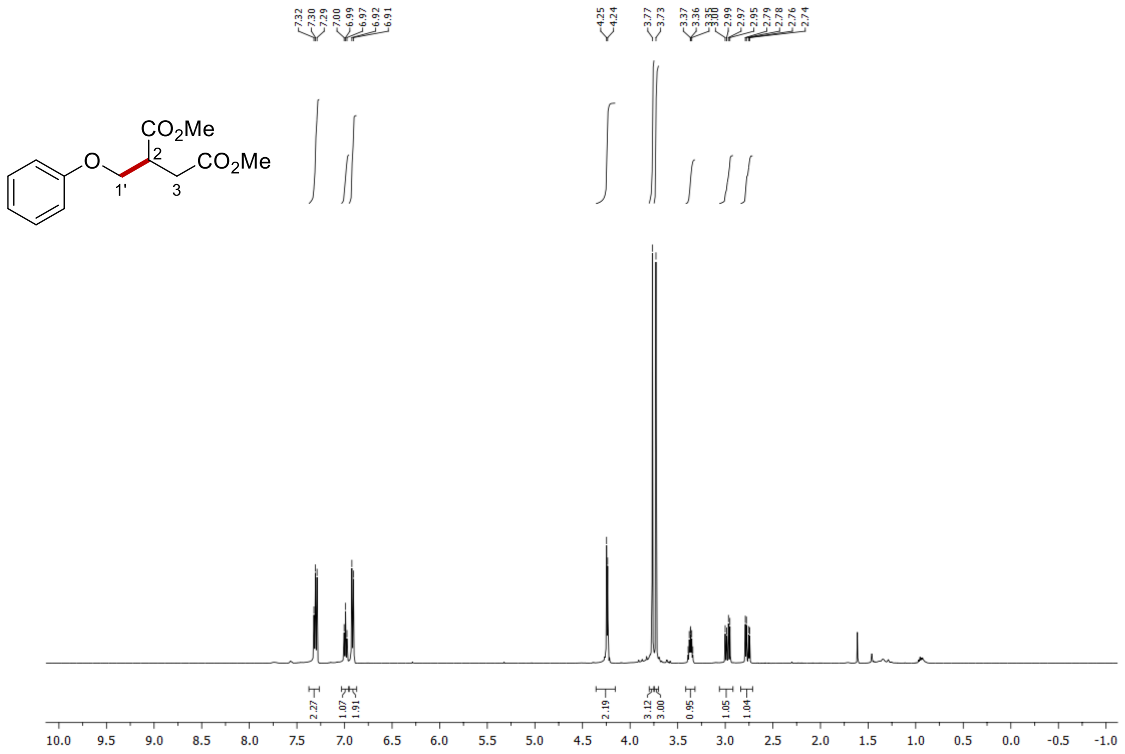

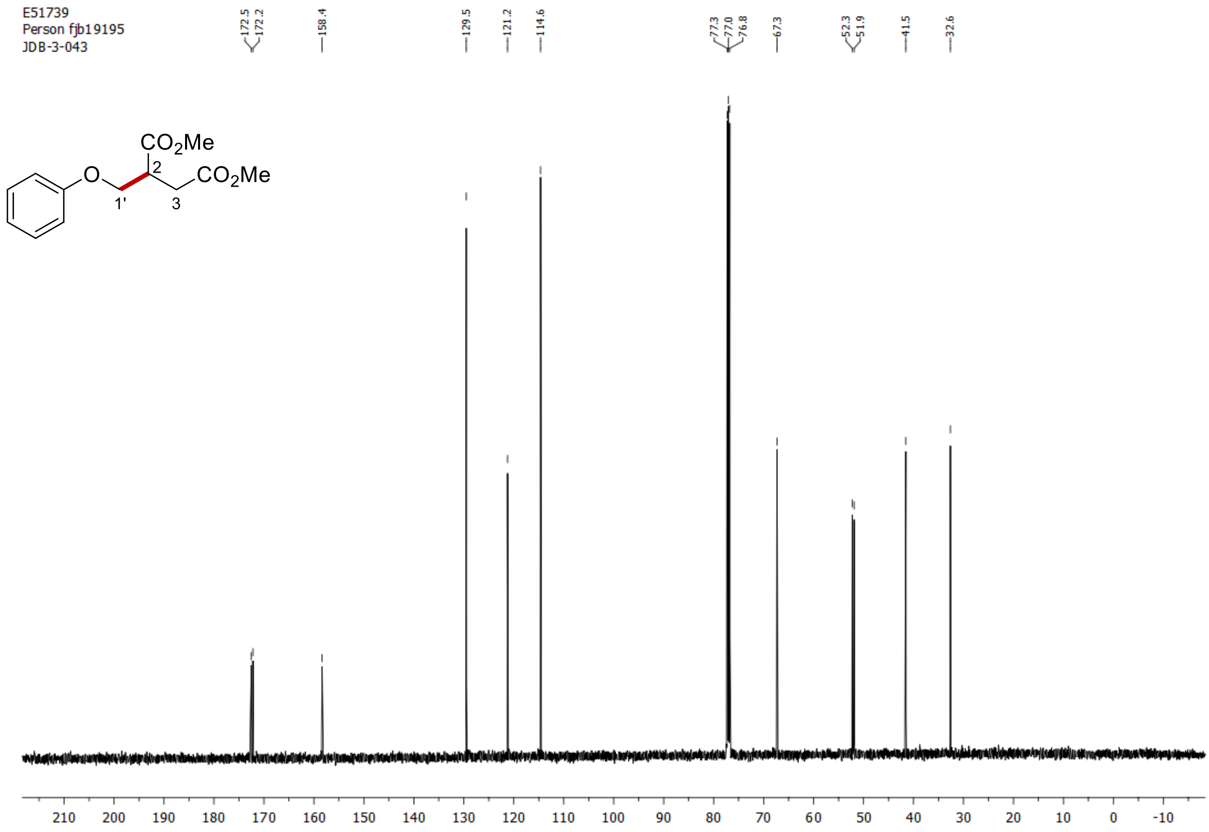

Dimethyl 2-[(4′′-fluorophenoxy)methyl]succinate (20b)

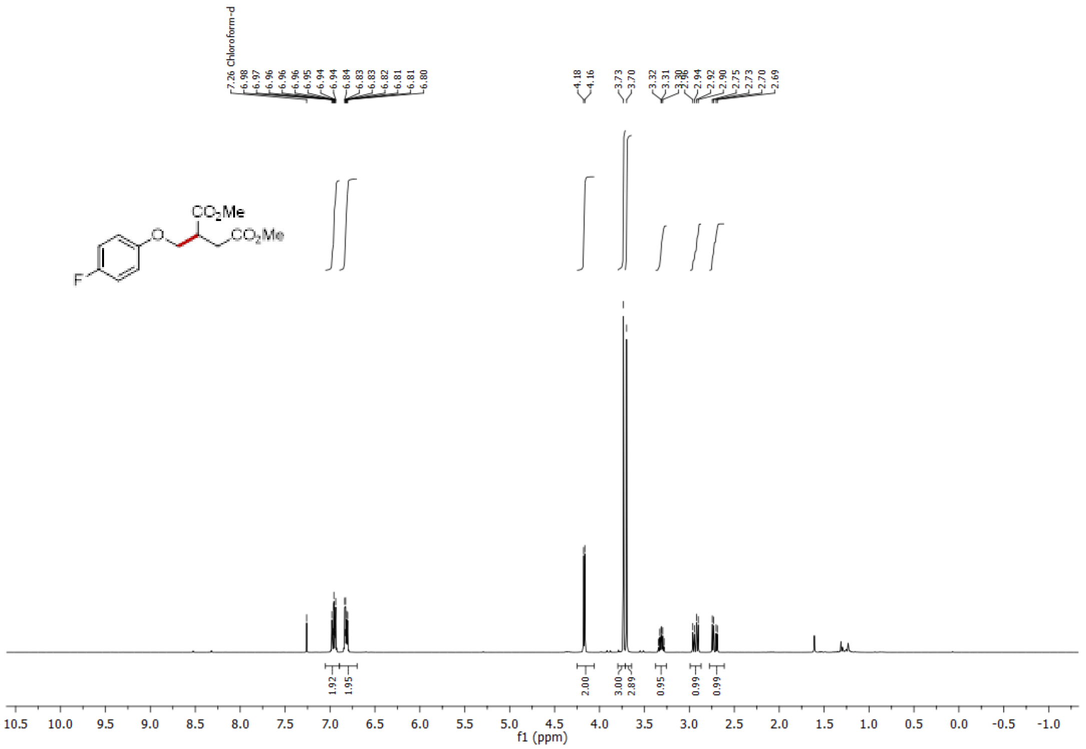

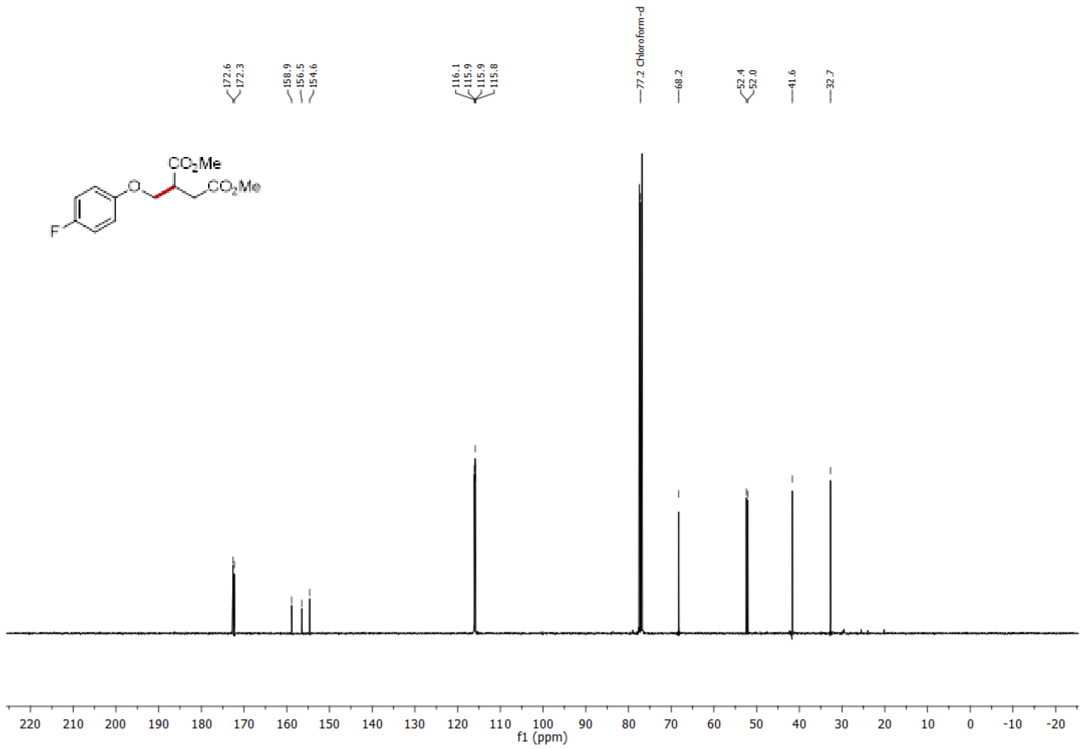

Dimethyl 2-[(4″-chlorophenoxy)methyl]succinate (20c)

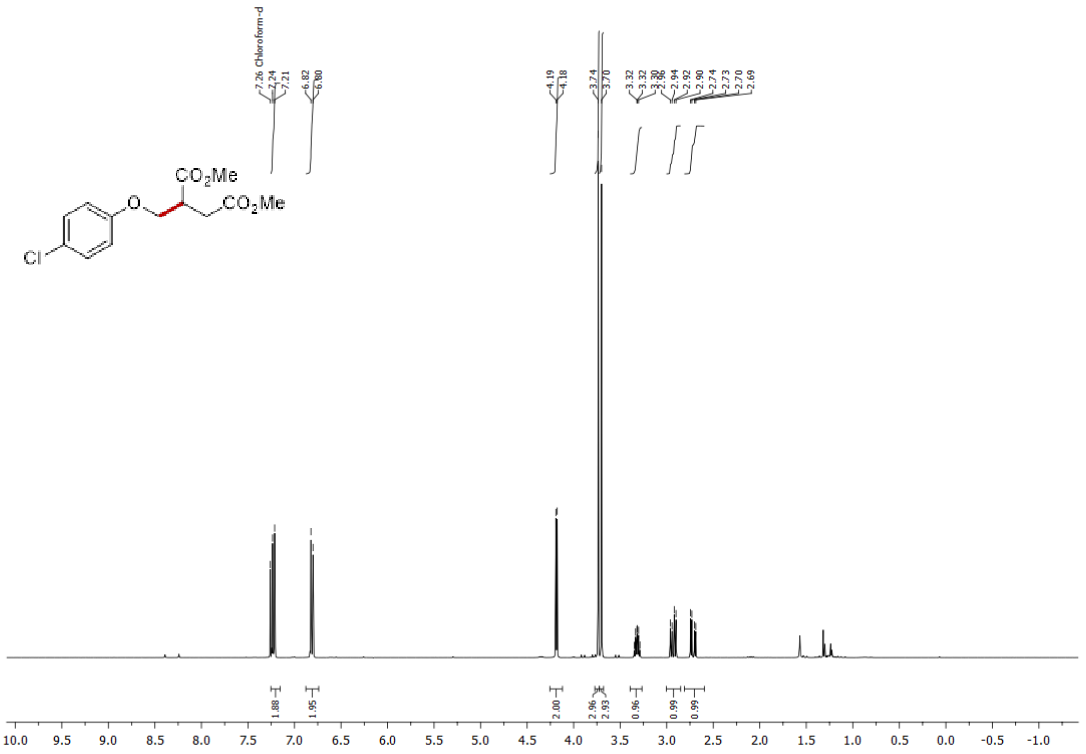

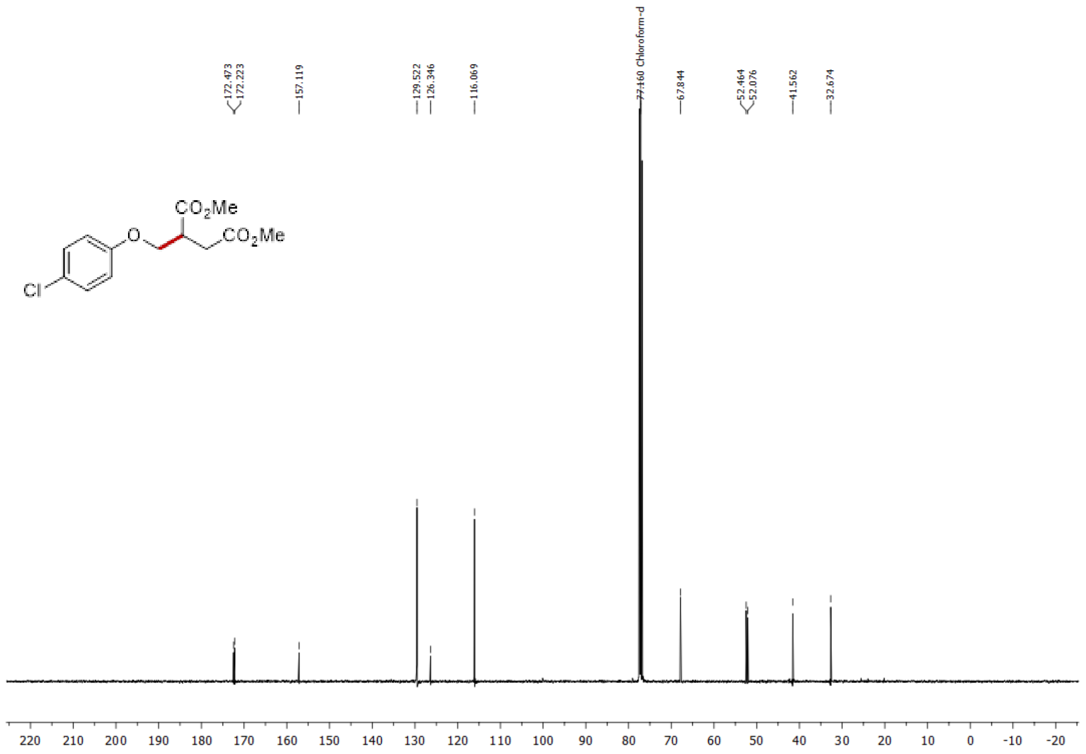

Dimethyl 2-((4″-(2‴-methyl-1‴,3‴-dioxolan-2‴-yl)phenoxy)methyl)succinate (20d)

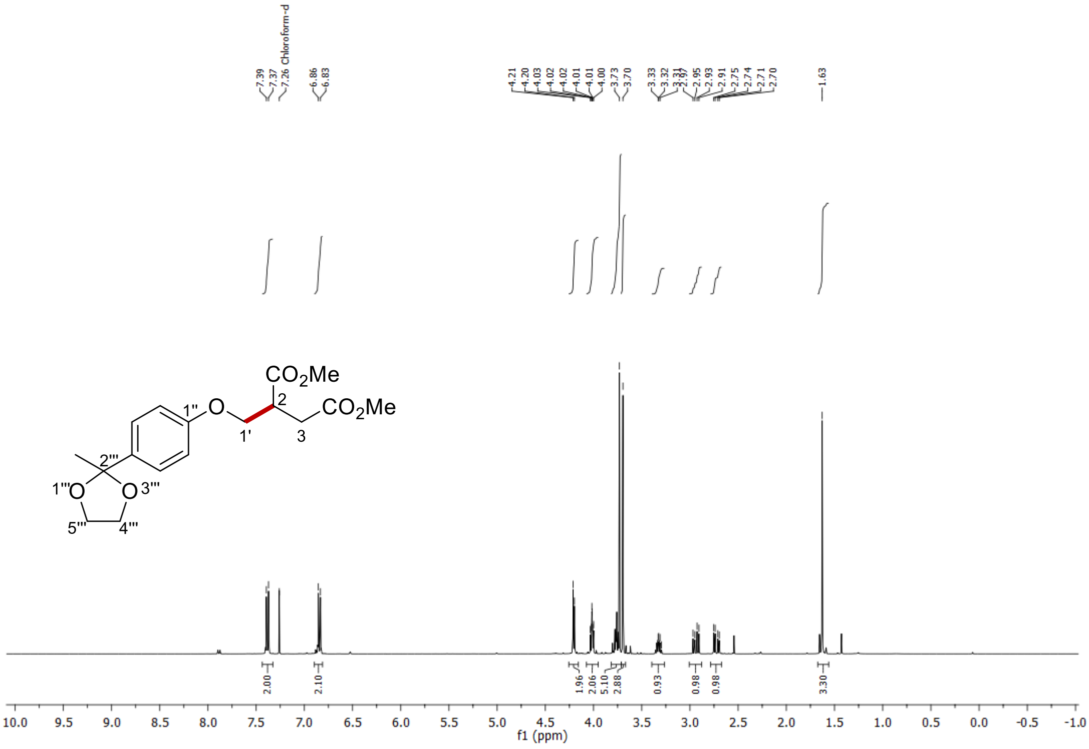

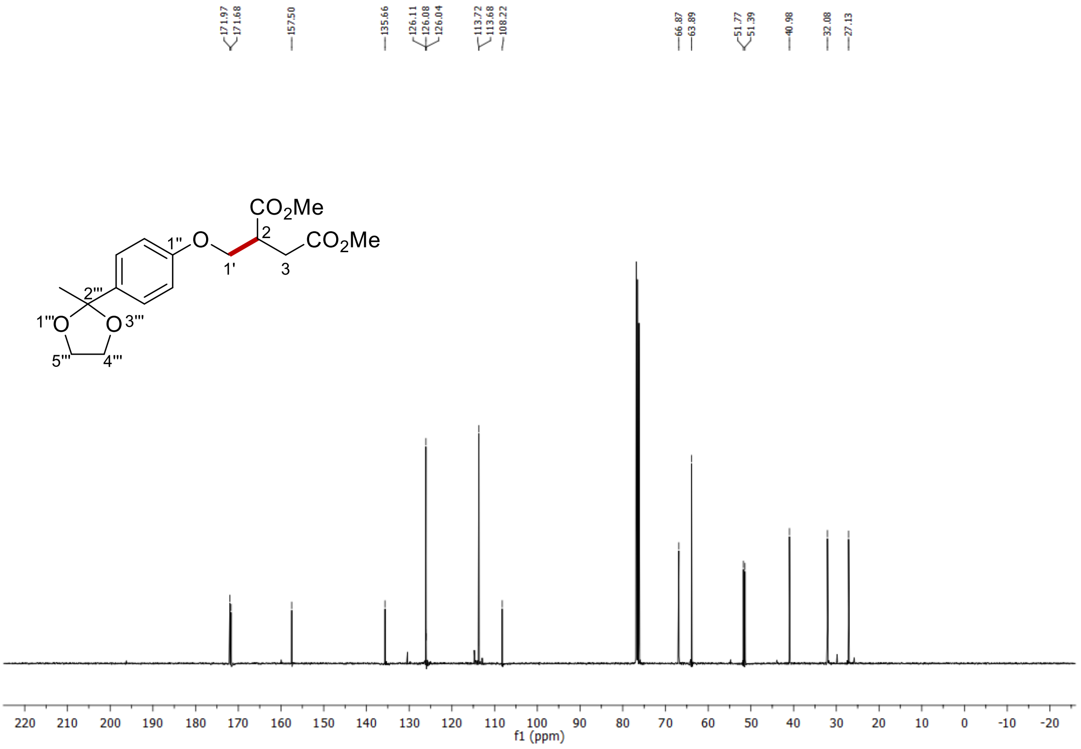

Dimethyl 2-[(3′′-methoxylphenoxy)methyl]succinate (20e)

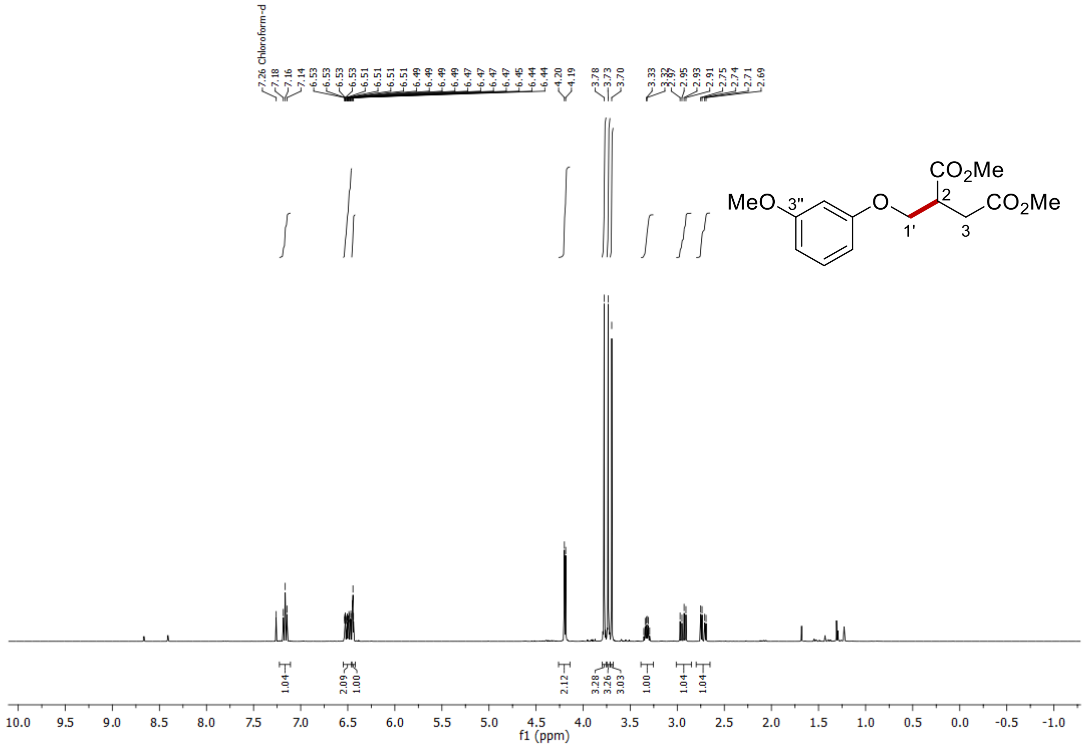

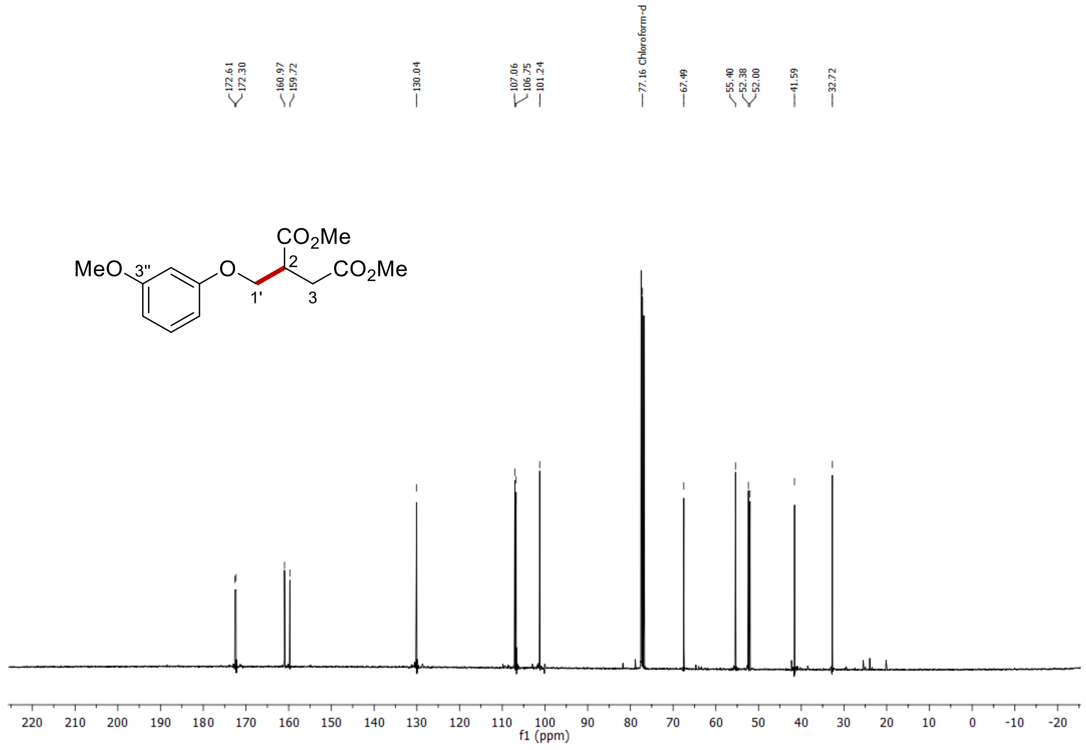

Dimethyl 2-[(4′′-methoxyphenoxy)methyl]succinate (20f)[20]

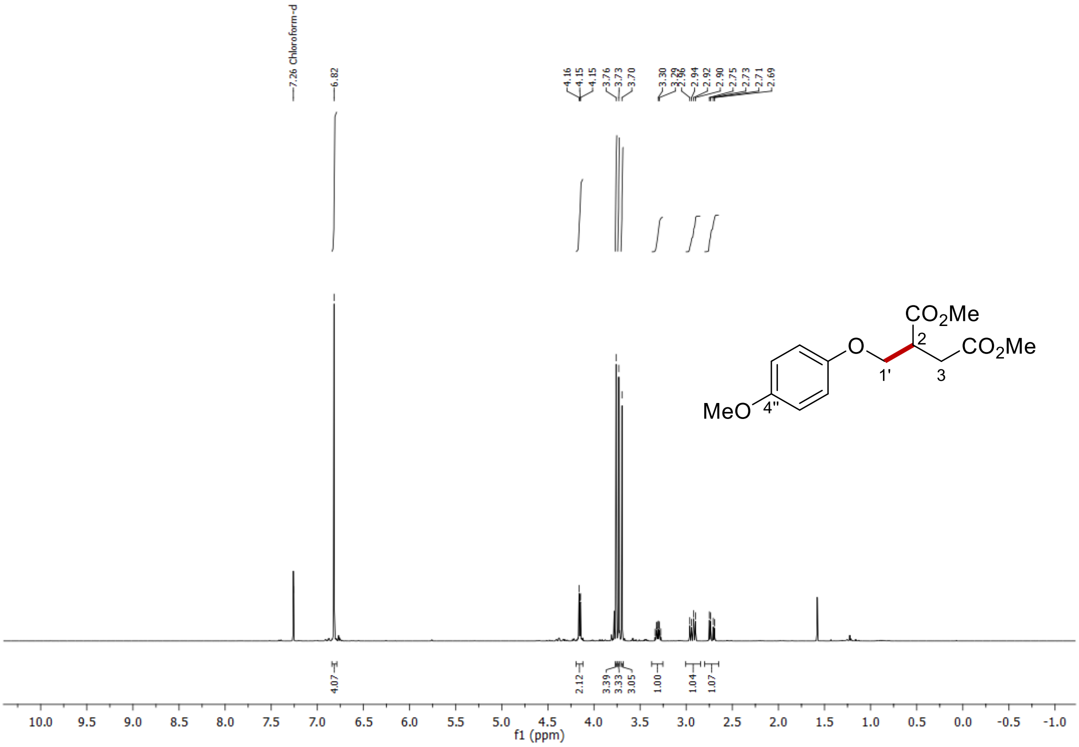

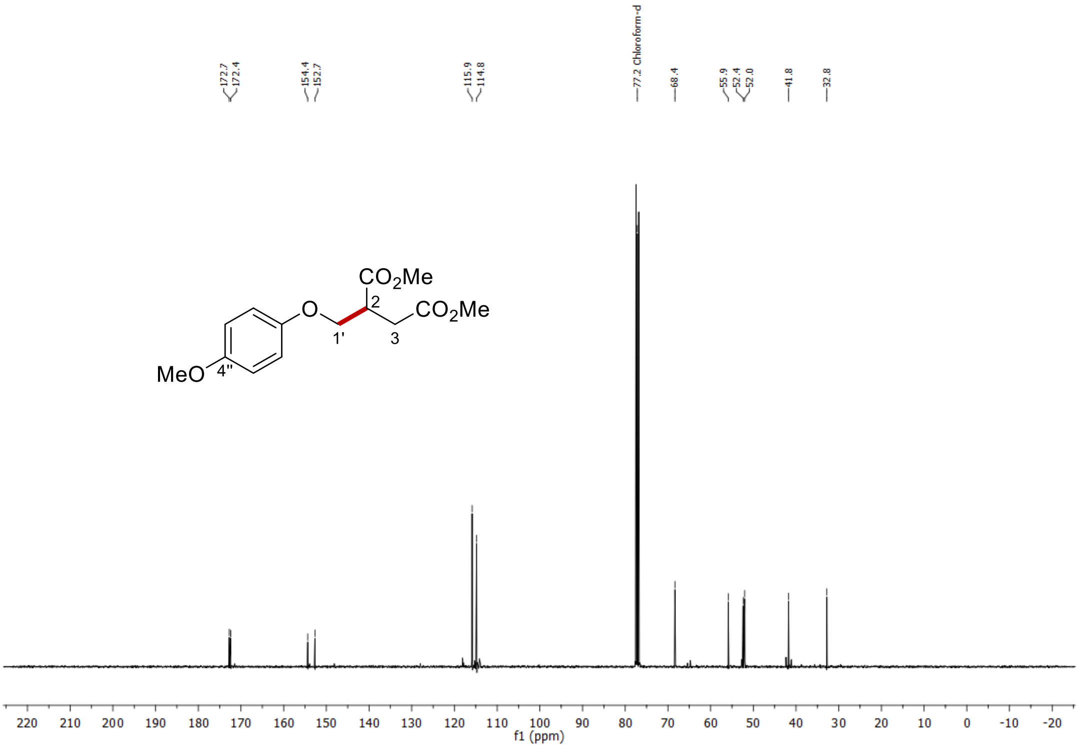

Dimethyl 2-(1′-phenoxyeth-1′-yl)succinate (20g)

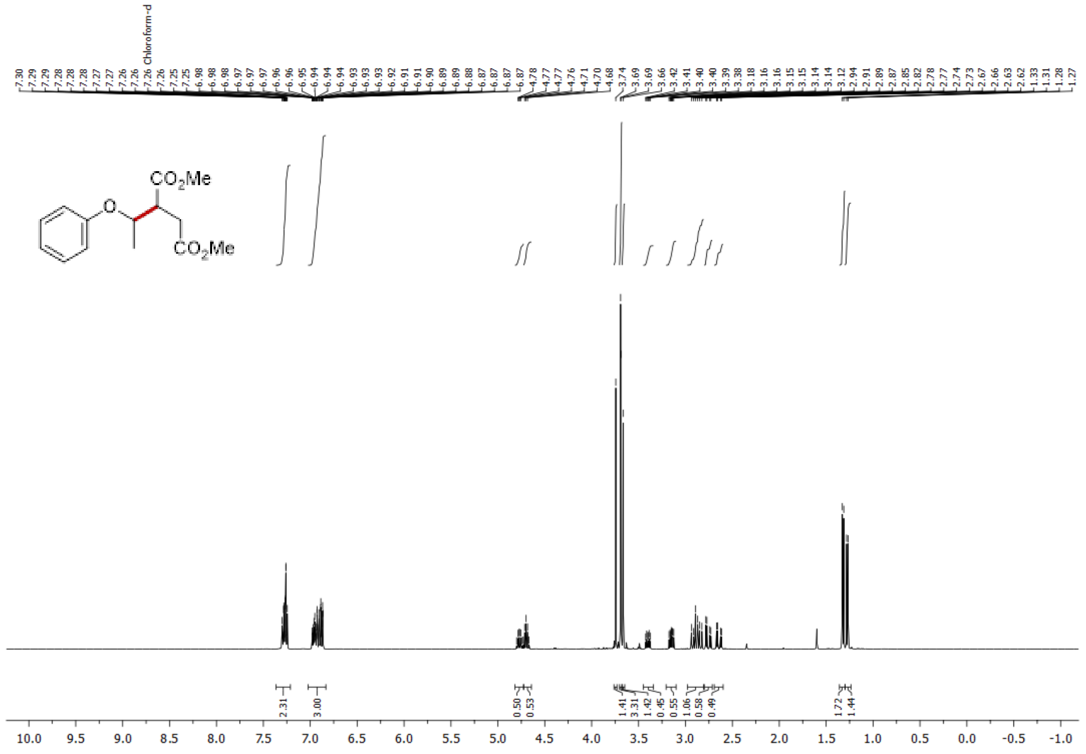

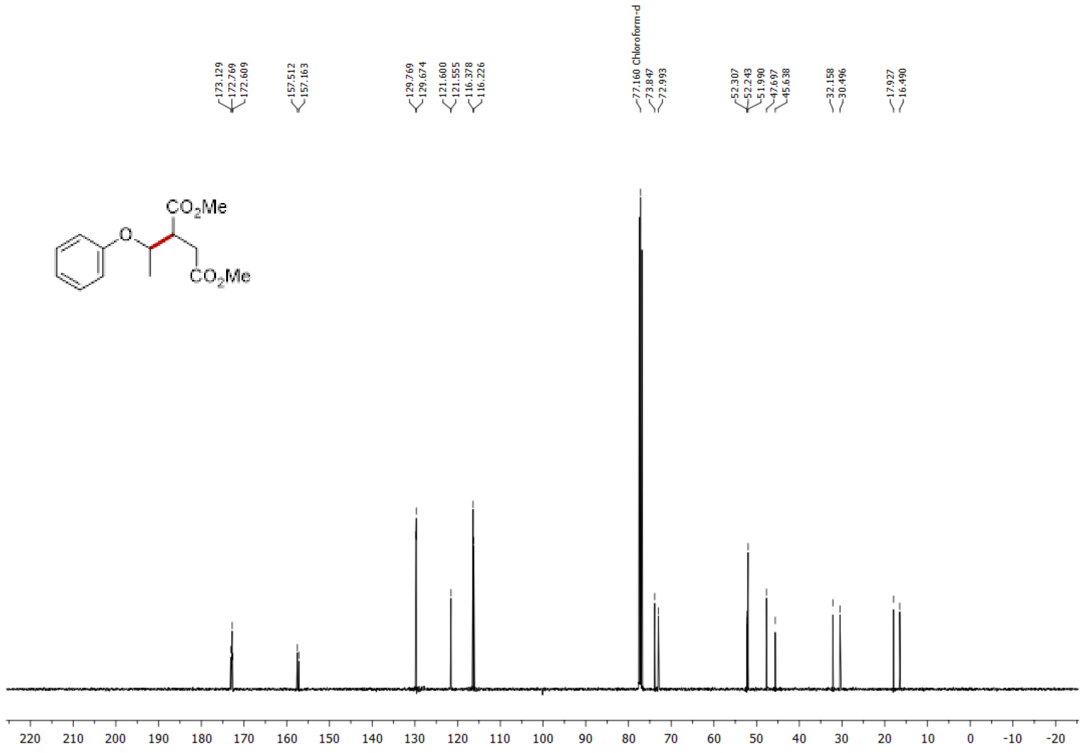

Dimethyl 2-(1′-phenoxyhex-1′-yl)succinate (20h)

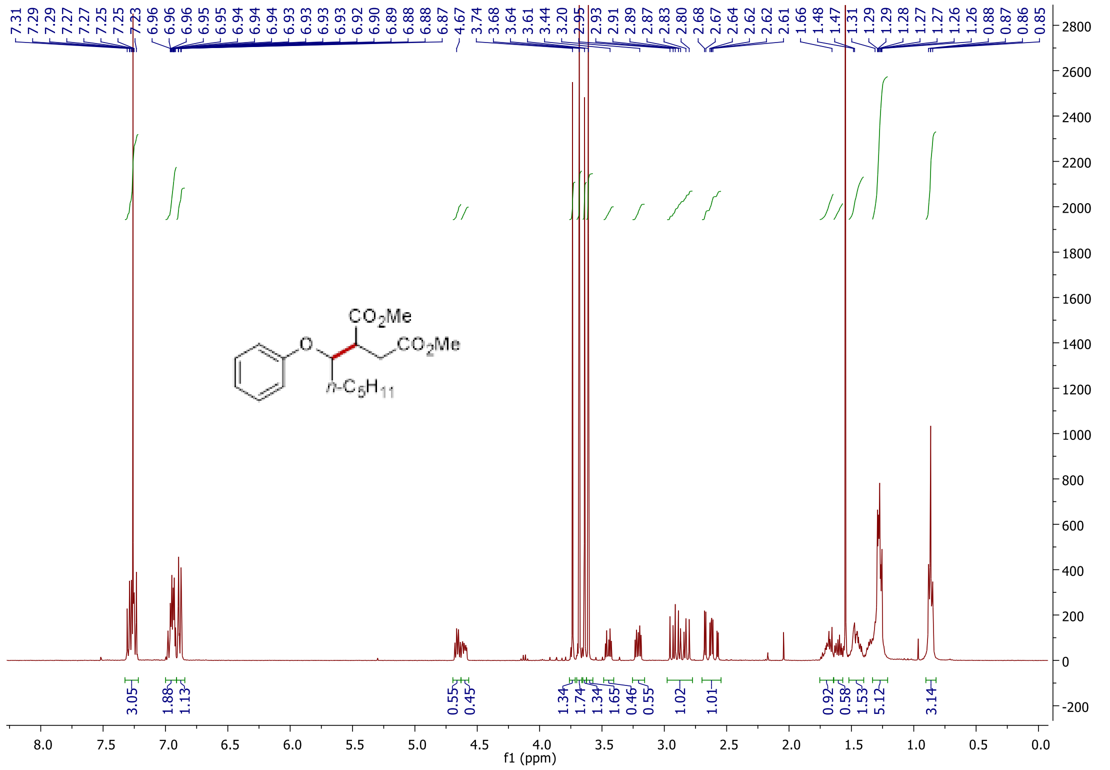

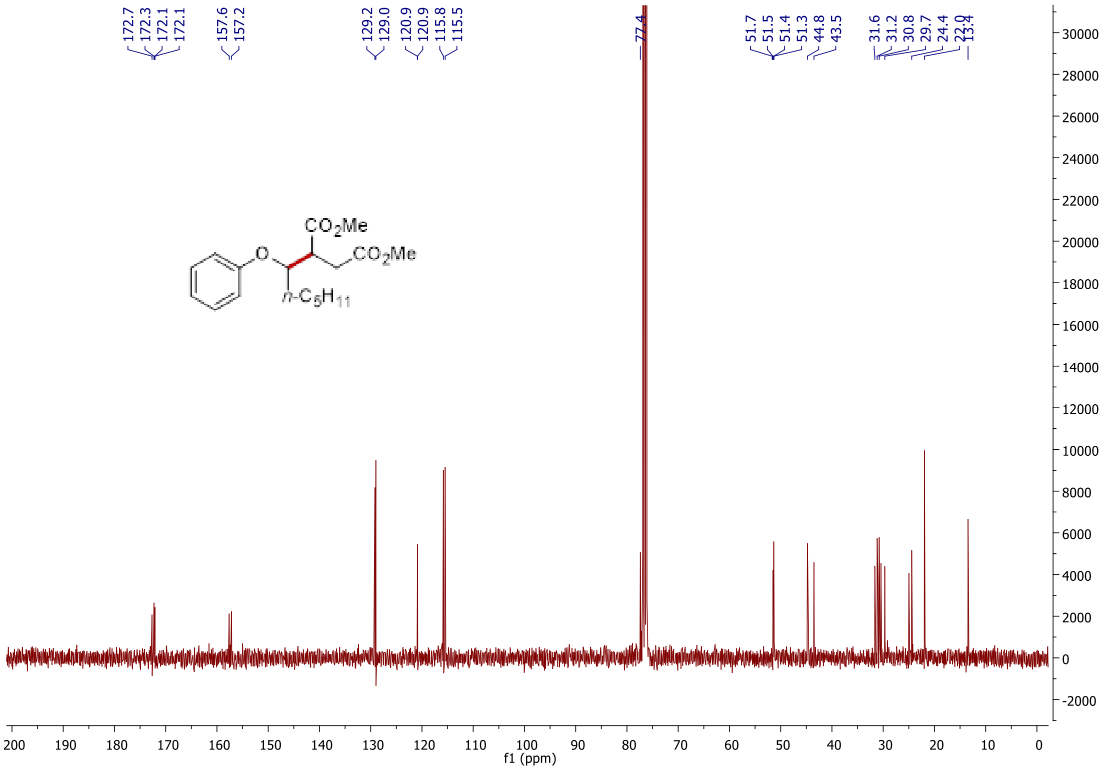

Dimethyl 2-[phenoxy(phenyl)methyl]succinate (20i)

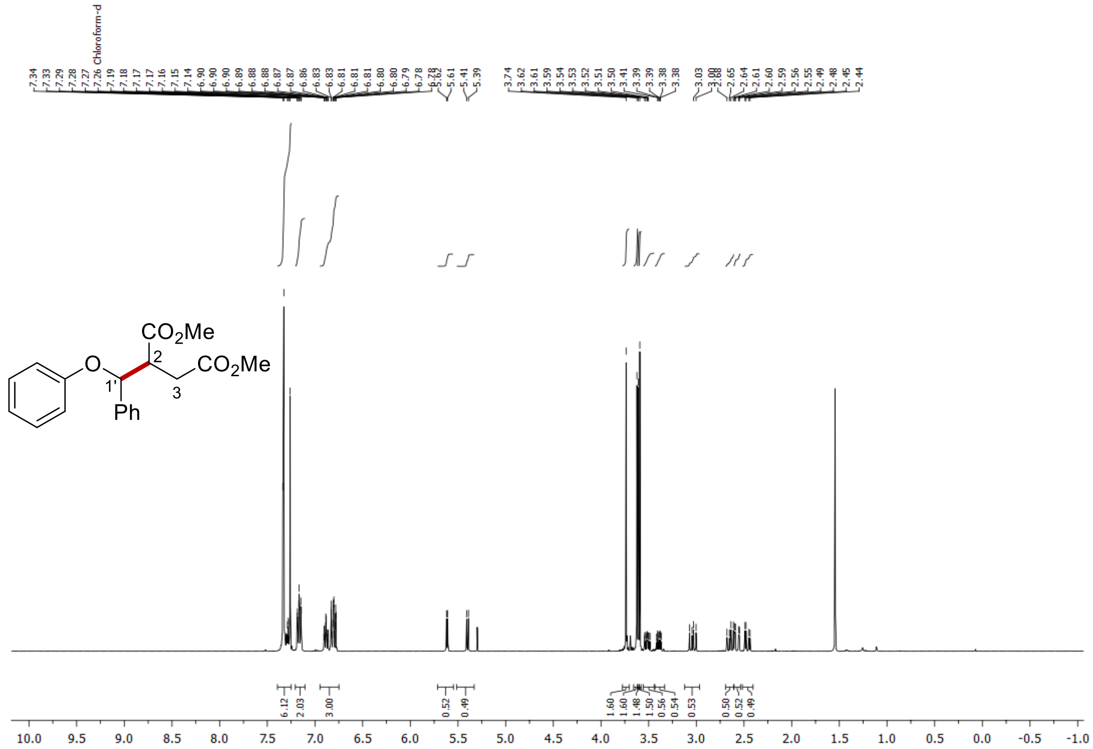

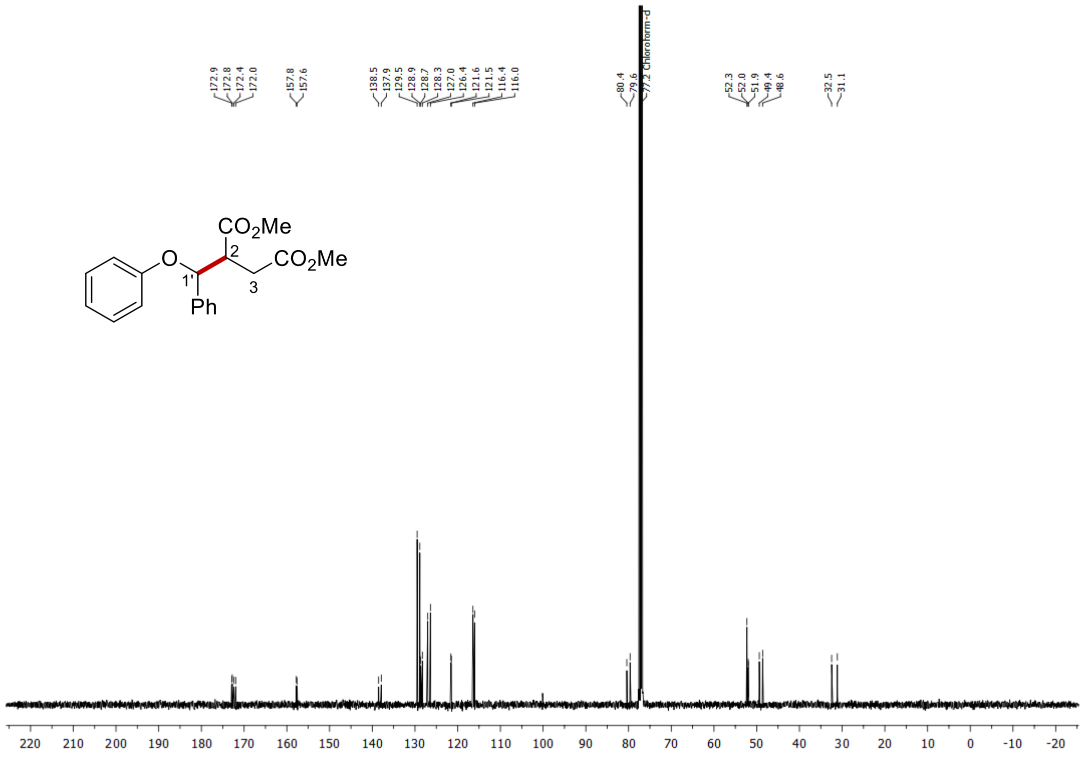

Dimethyl 2-[3′-(1″,3″-dioxoisoindolin-2″-yl)-1′-phenoxyprop-1′-yl]succinate (20j)

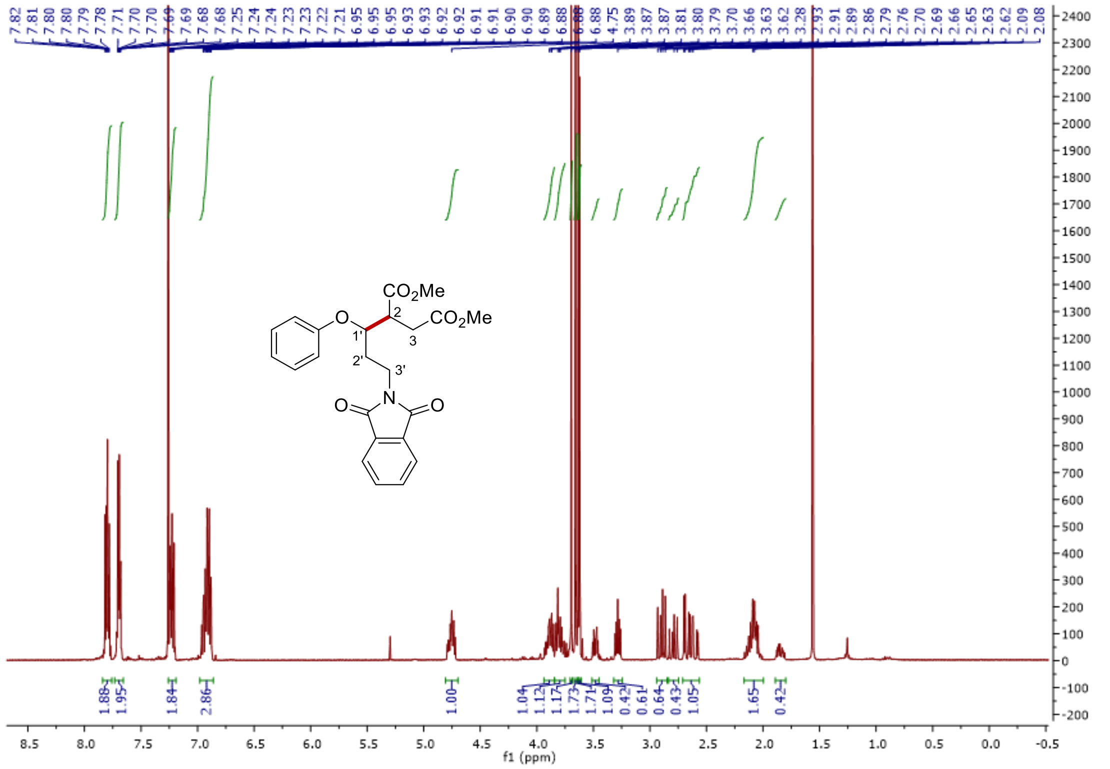

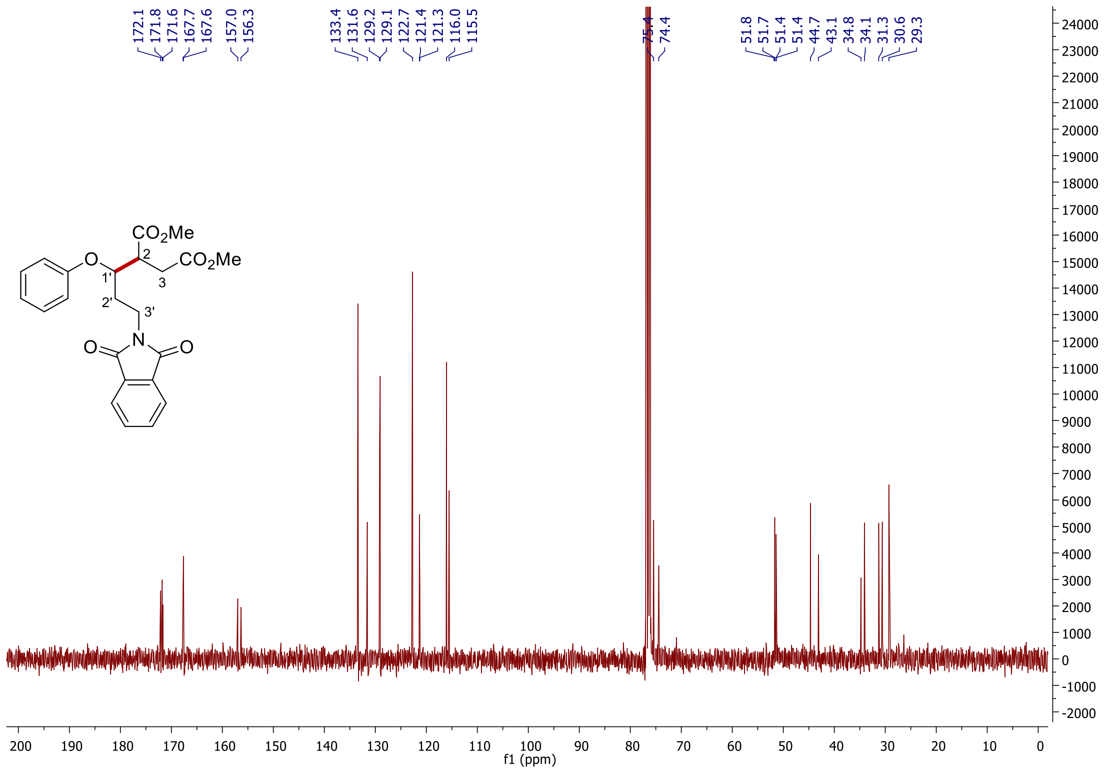

Dimethyl 2-(3′-acetoxy-1′-phenoxypropyl)succinate (20k)

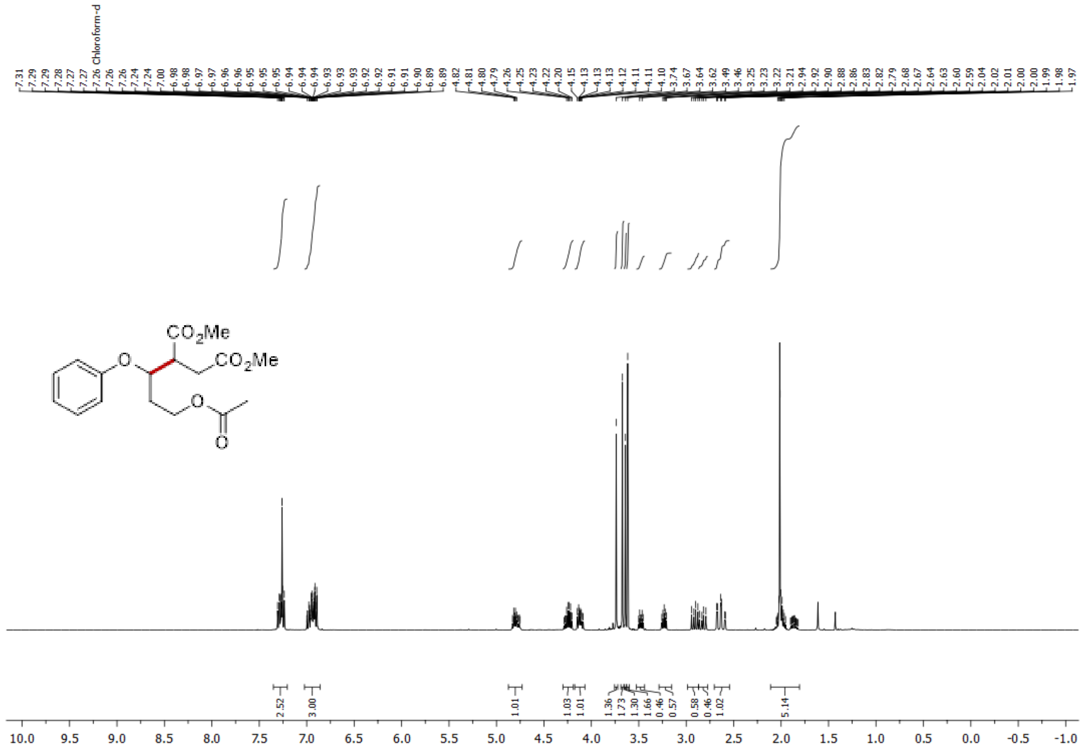

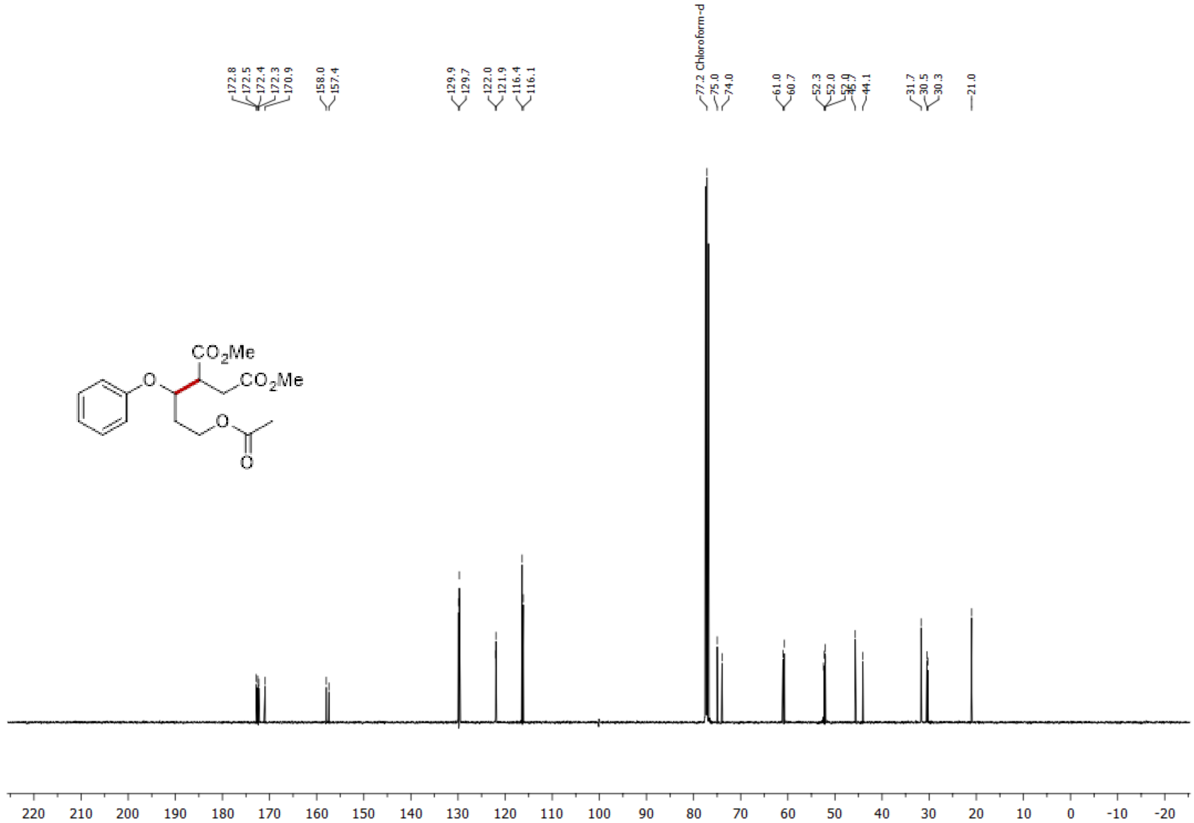

Dimethyl 2-(3′-benzoyloxy-1′-phenoxypropyl)succinate (20l)

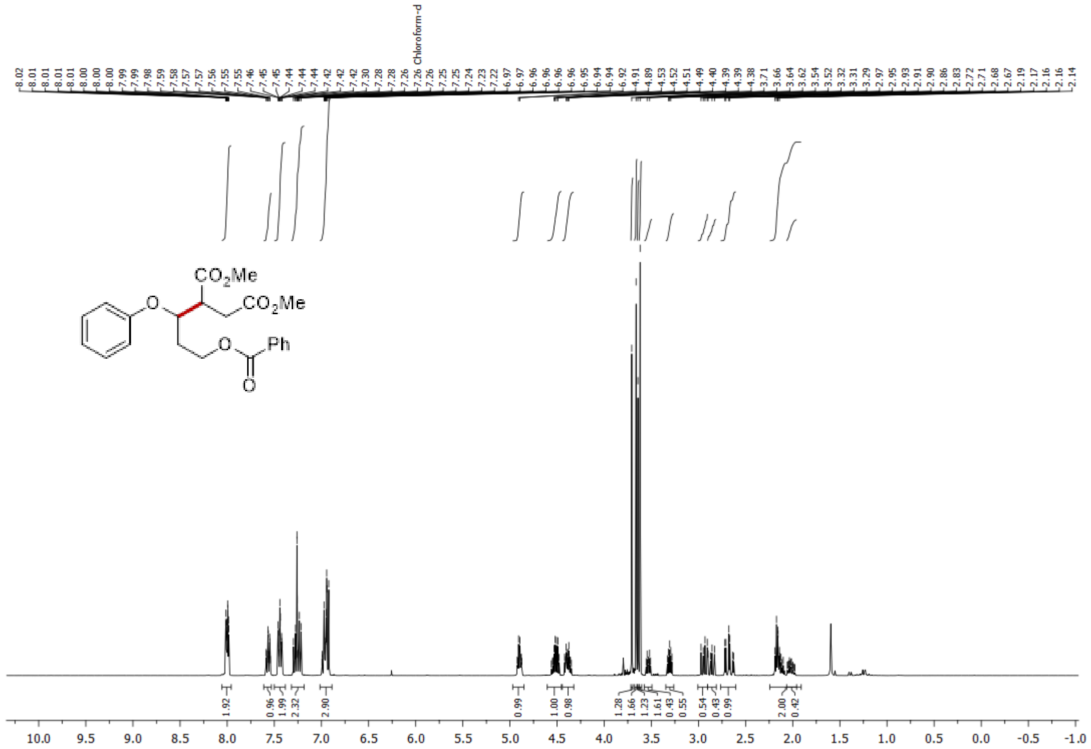

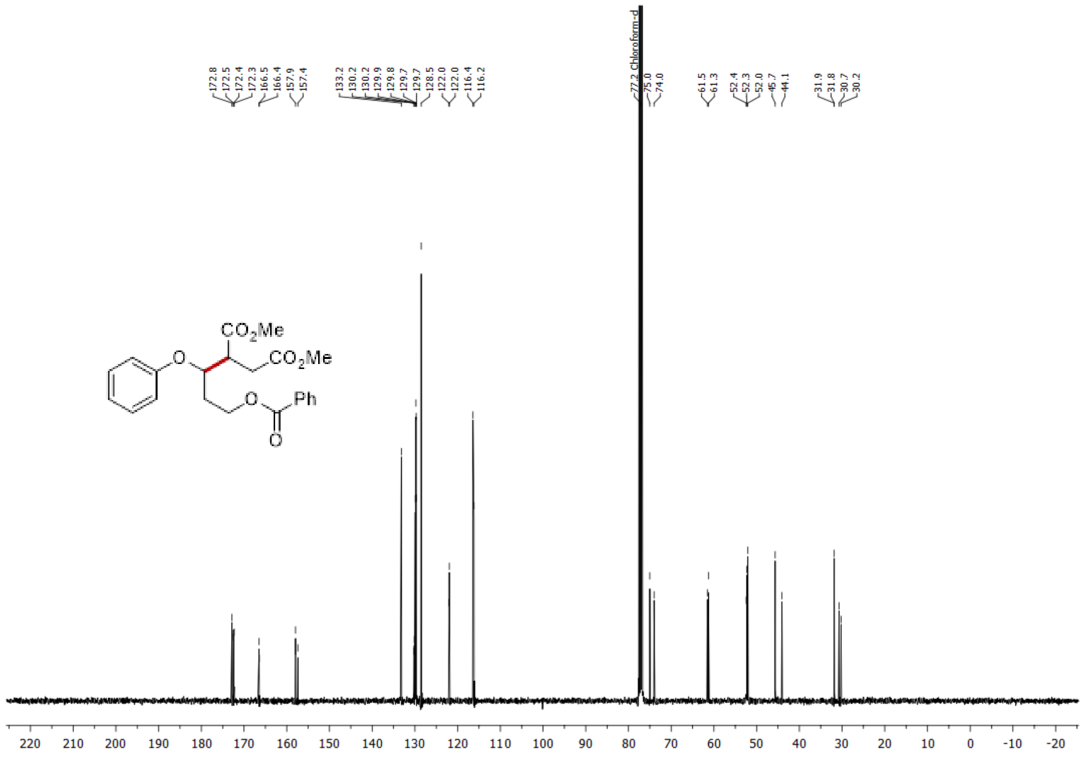

Dimethyl 2-(2′-phenoxypropan-2′-yl)succinate (20m)

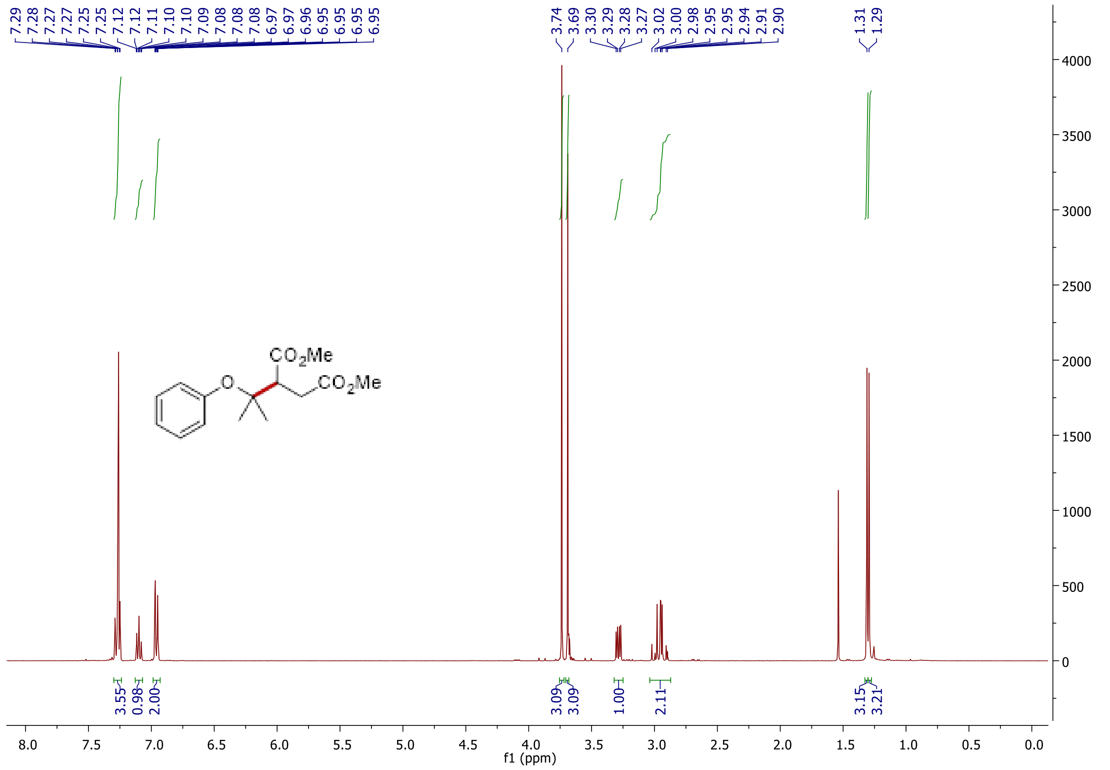

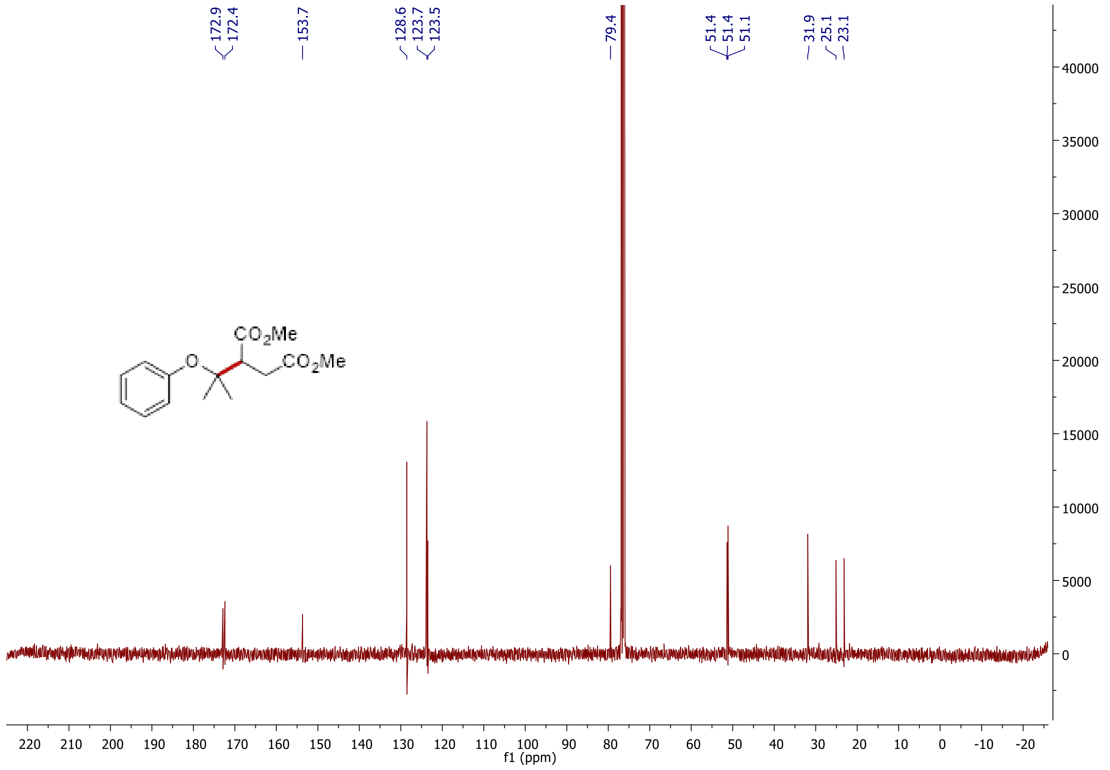

Dimethyl 2-(1′-phenoxycyclobut-1′-yl)succinate (20n)

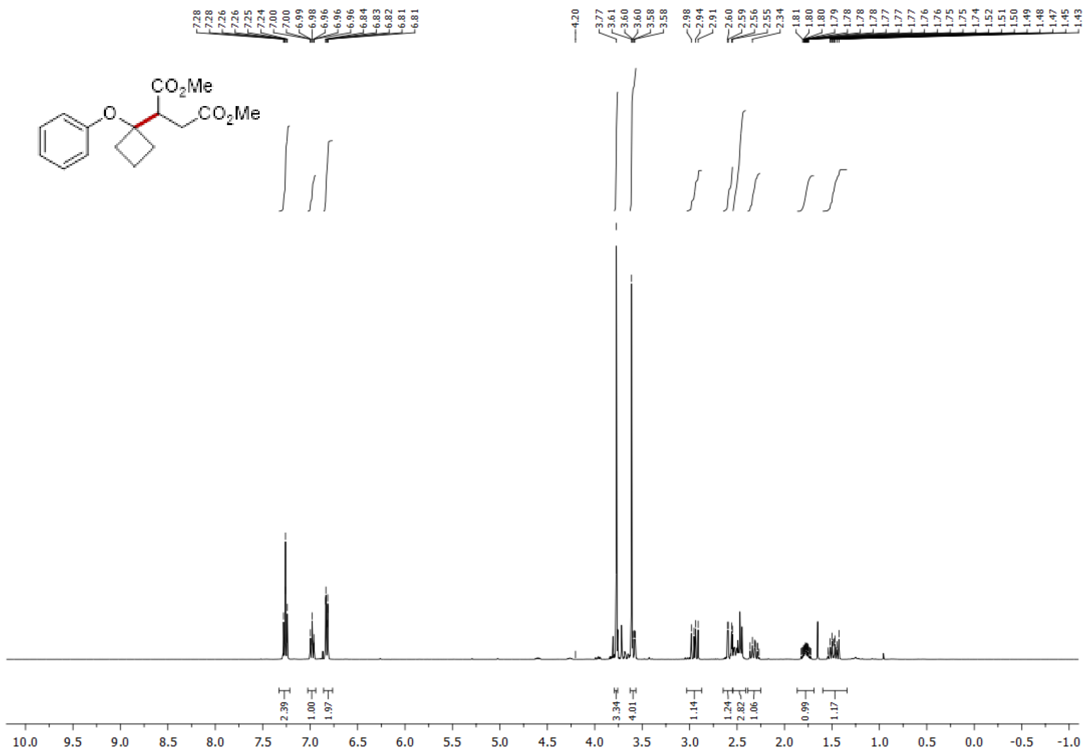

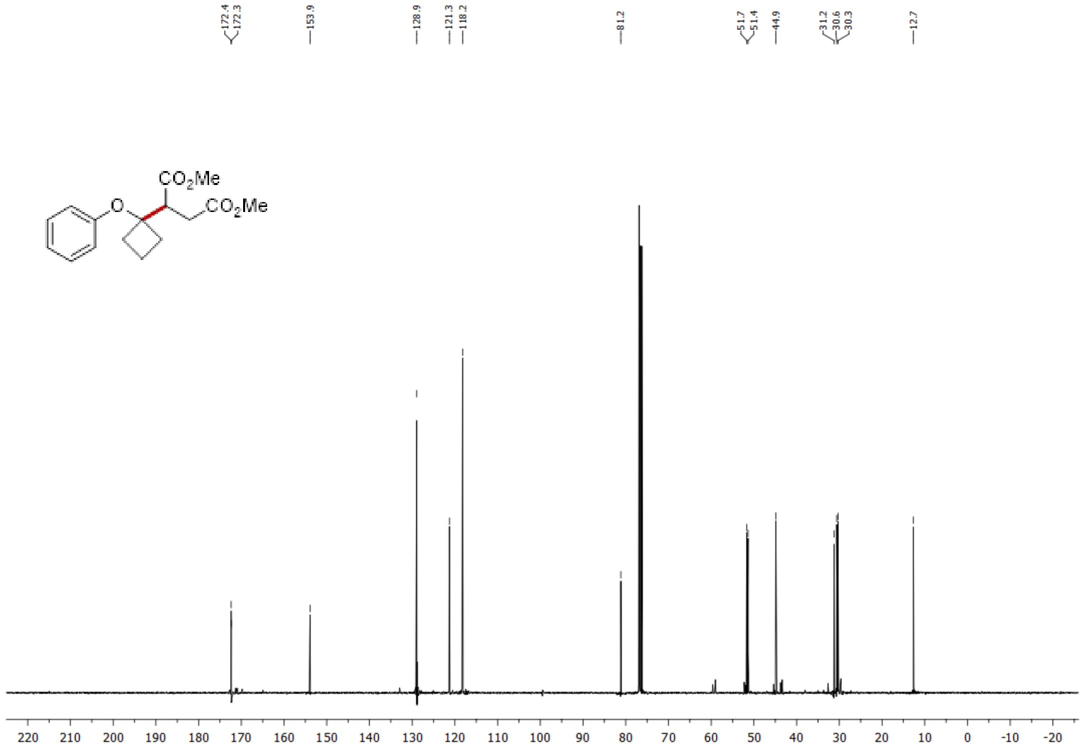

Dimethyl 2-(1′-phenoxycyclopent-1′-yl)succinate (20o)

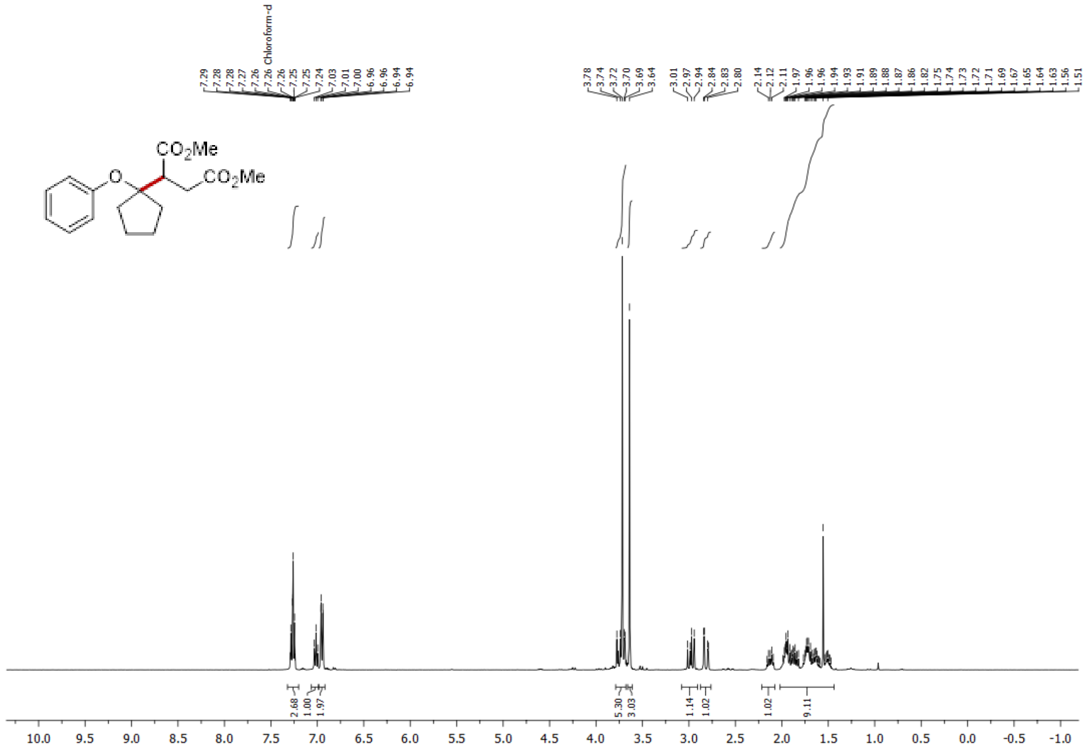

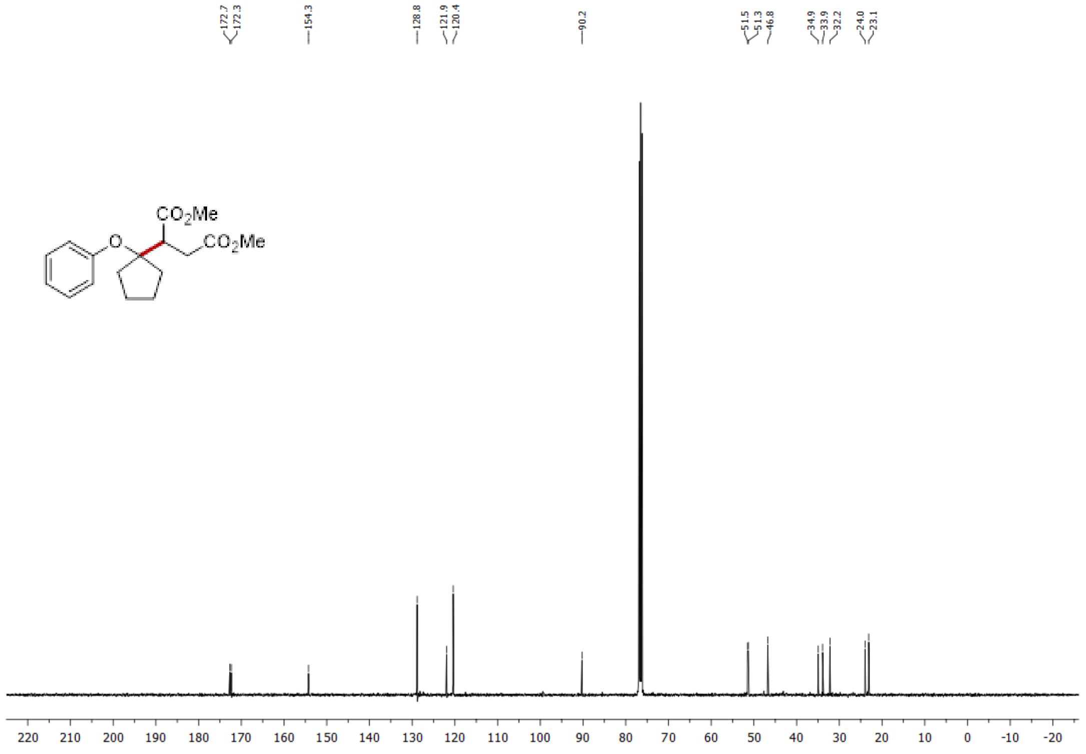

Dimethyl 2-[(3′′-methylphenoxy)methyl]succinate (20p)

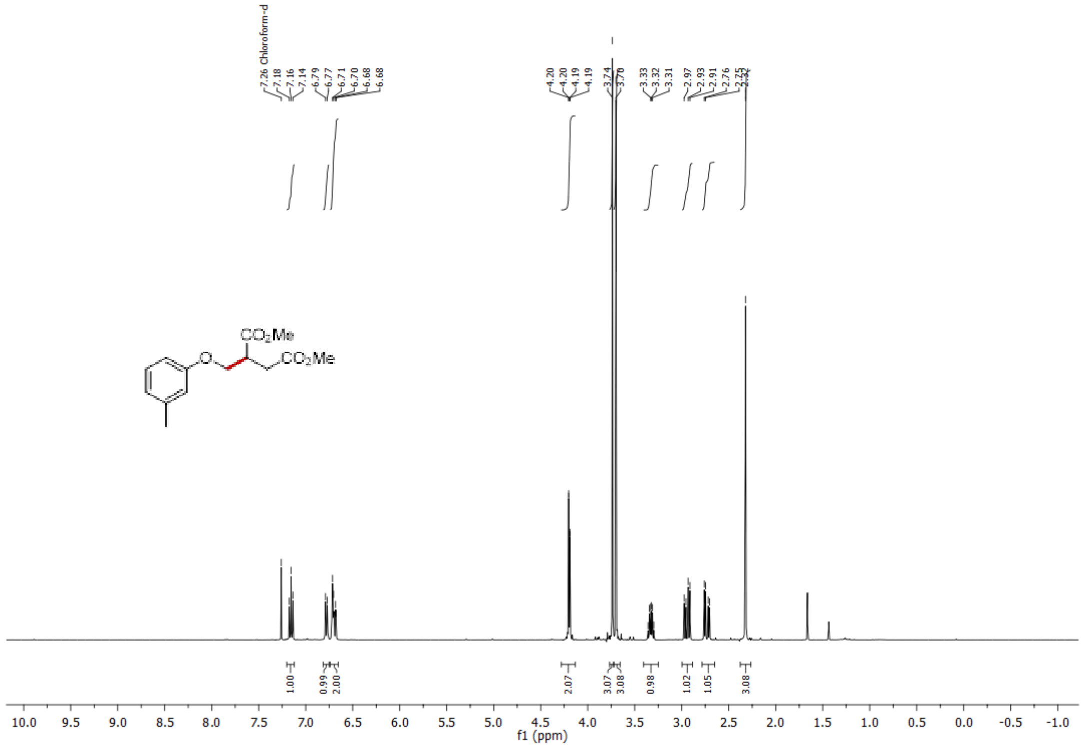

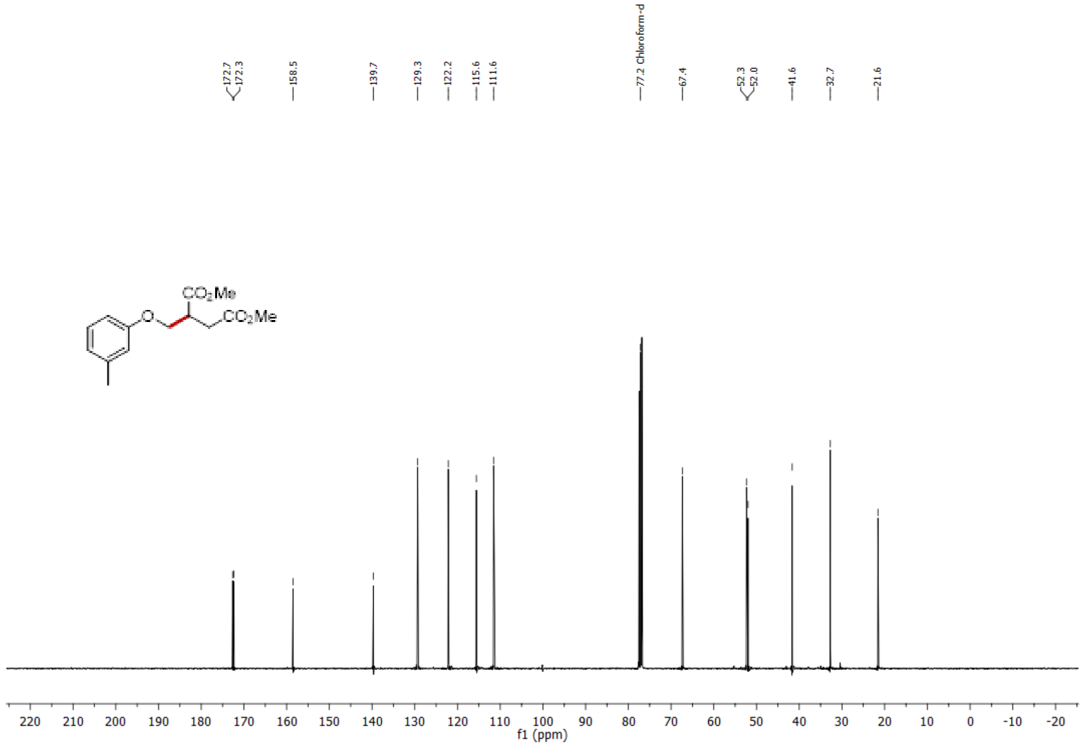

Dimethyl 2-[(4′′-methoxyphenyl)methyl]succinate (20q)

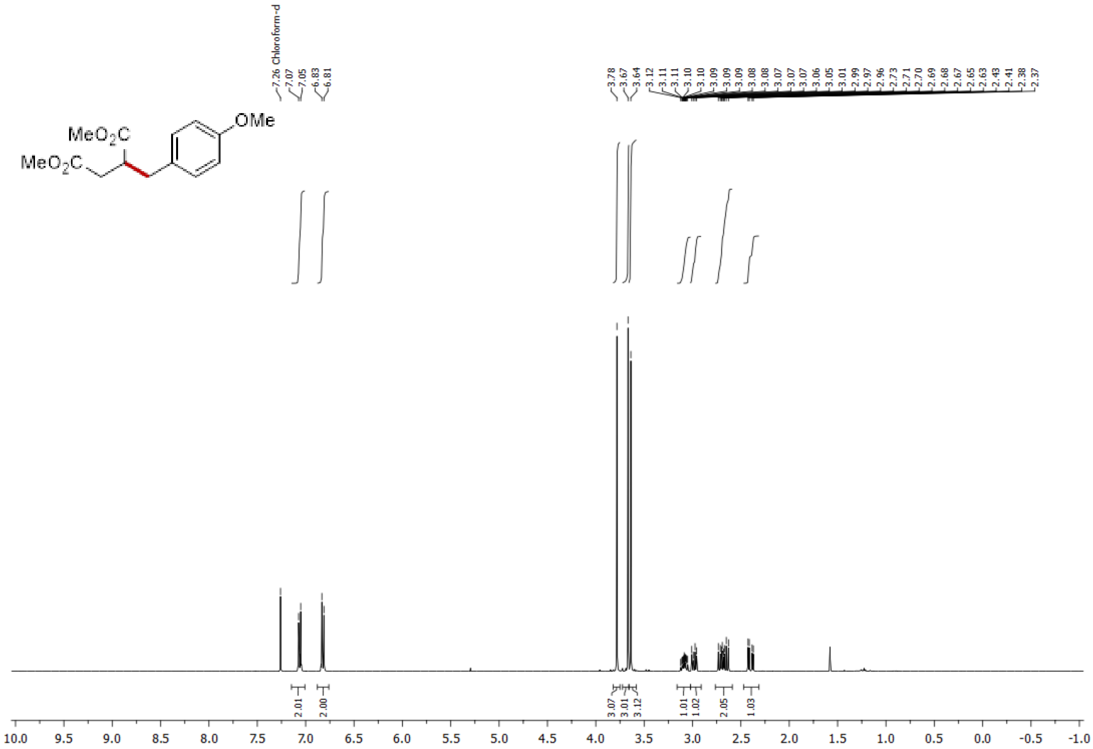

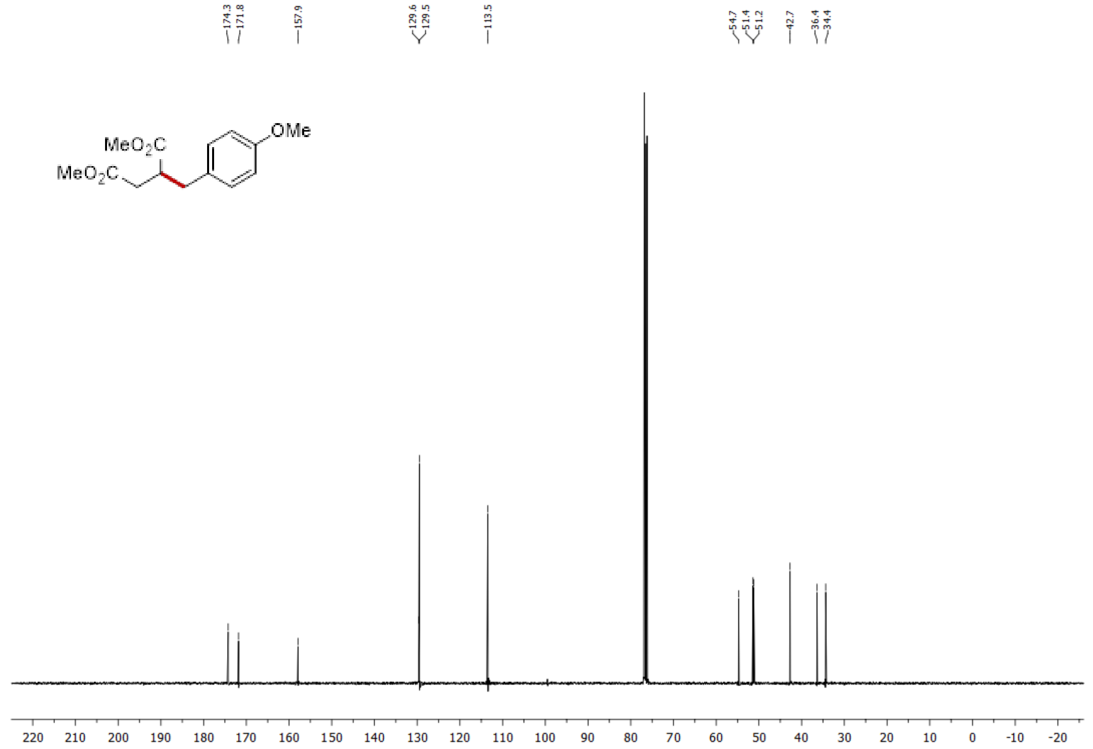

Dimethyl 2-(1′-phenoxy-3′-phenylprop-1′-yl)succinate (20r)

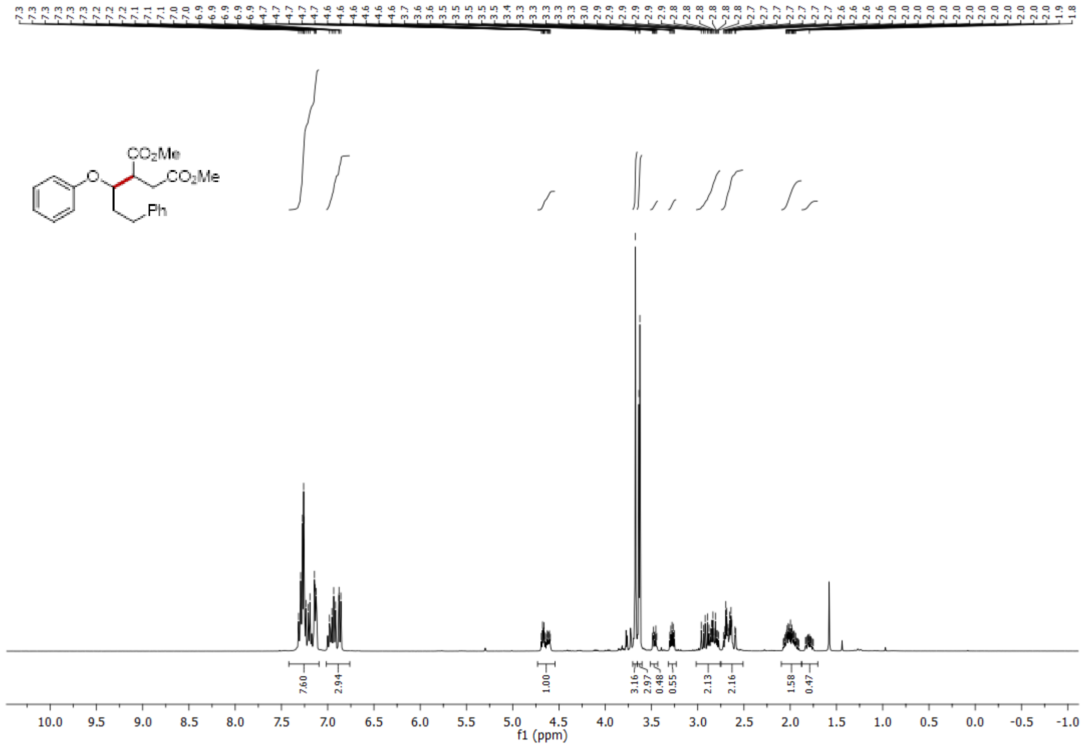

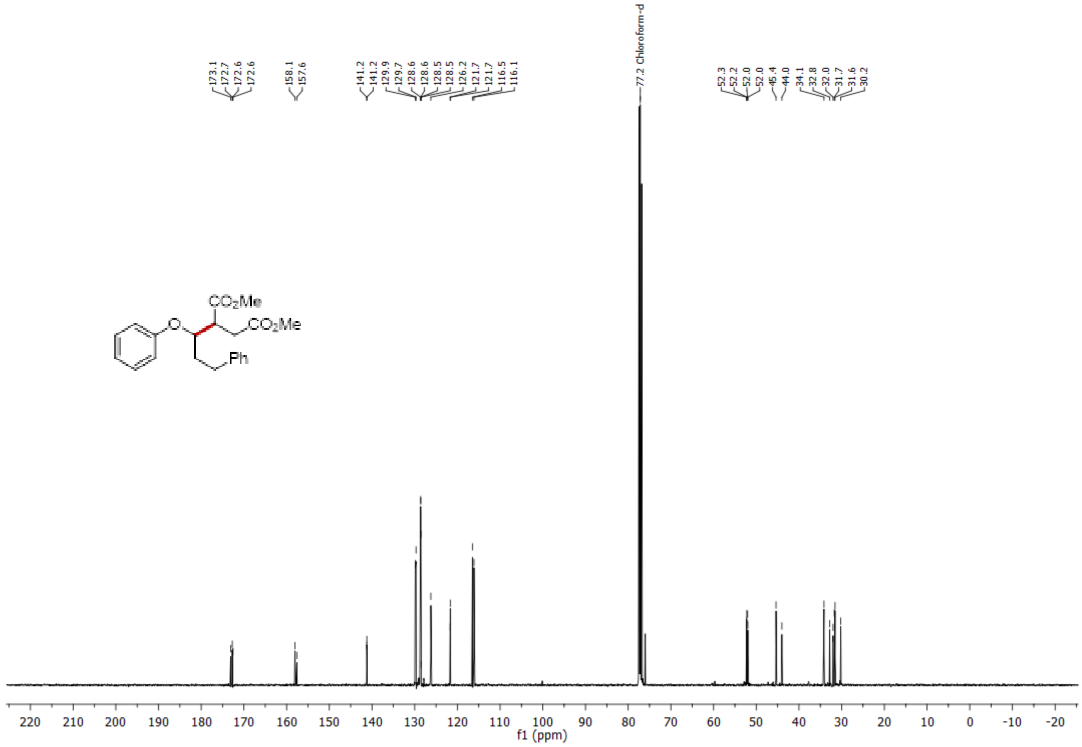

Dimethyl 2-[phenoxy(*p*-tolyl)methyl]succinate (20s)

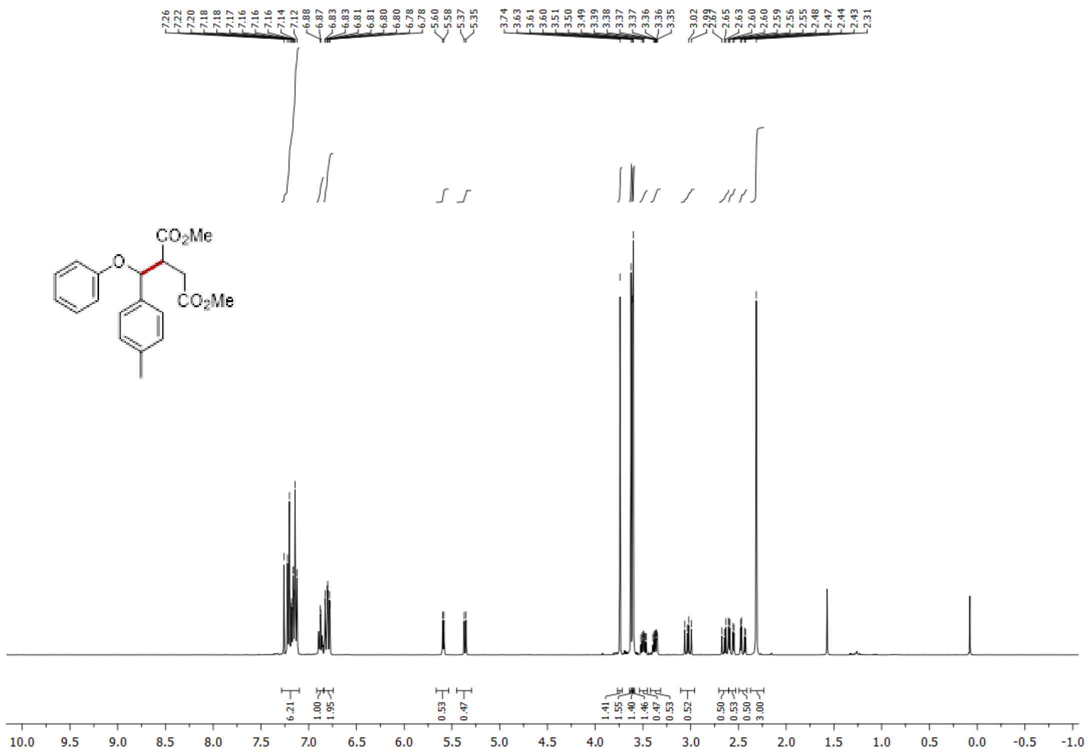

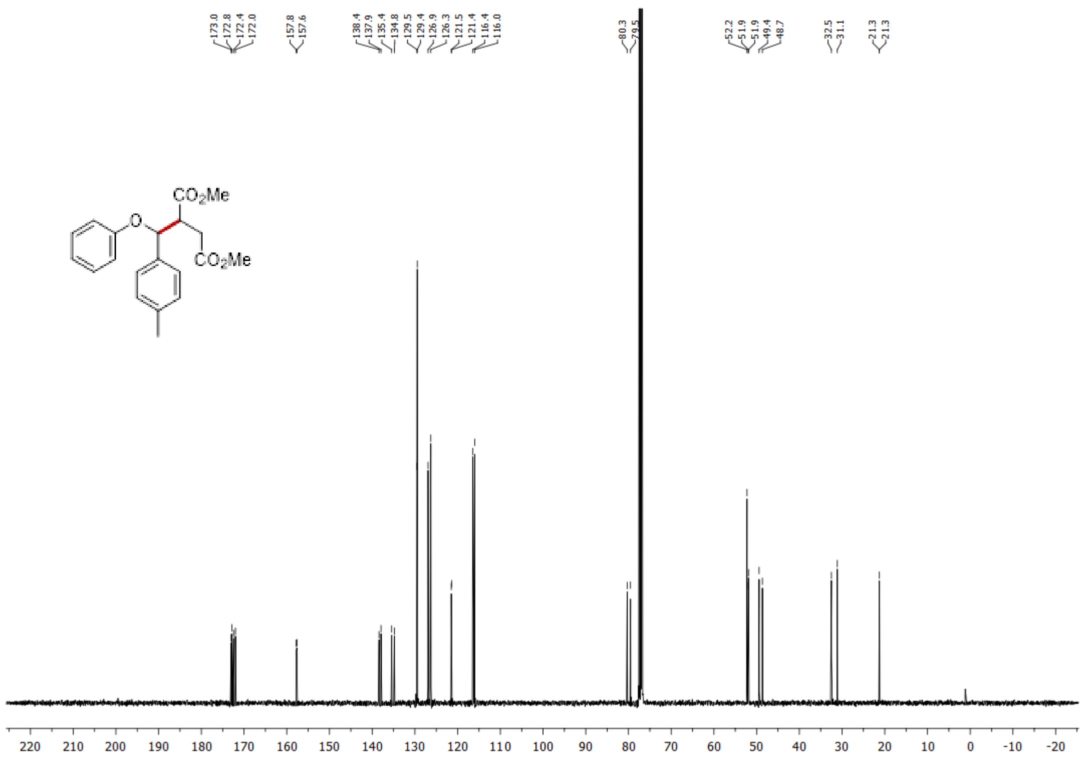

Dimethyl 2-{[(4′′-methoxyphenyl)thio]methyl}succinate (20t)

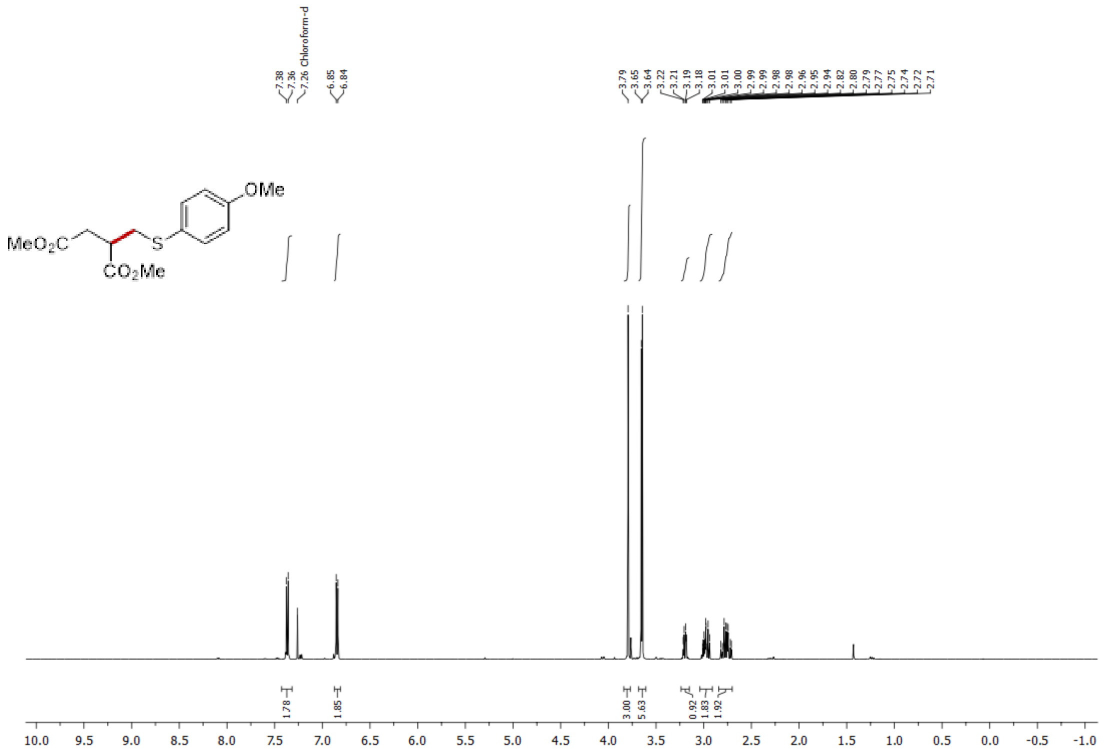

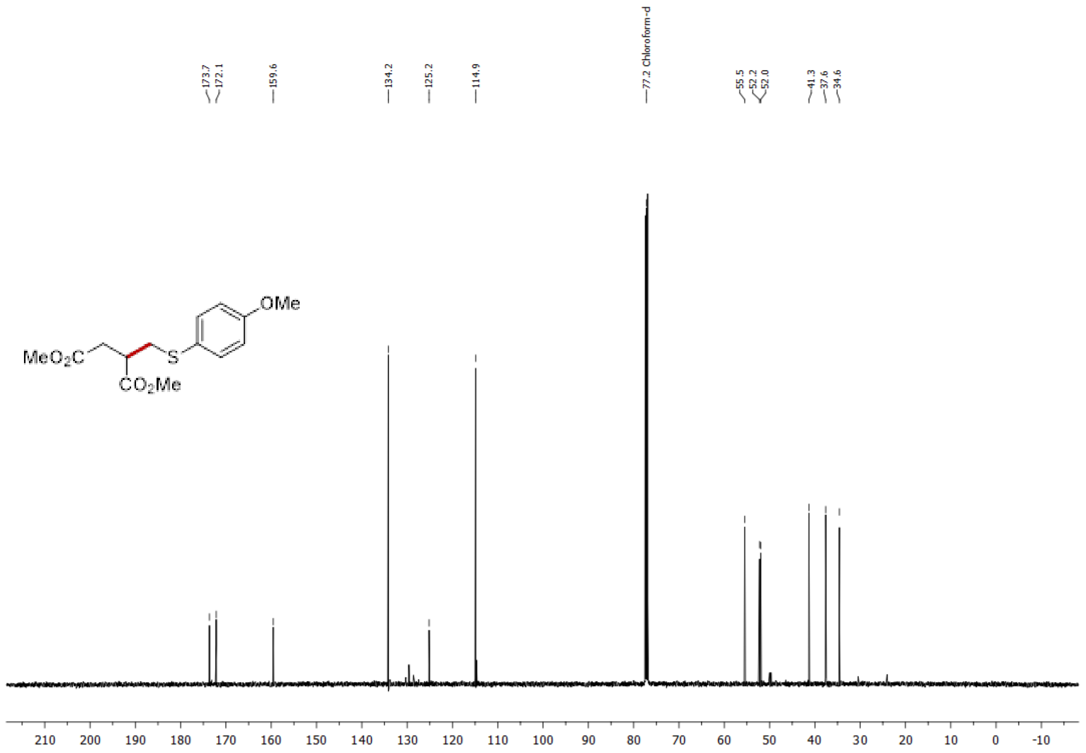

2-(2′-Phenoxy-1′-phenyleth-1′-yl)malononitrile (22a)

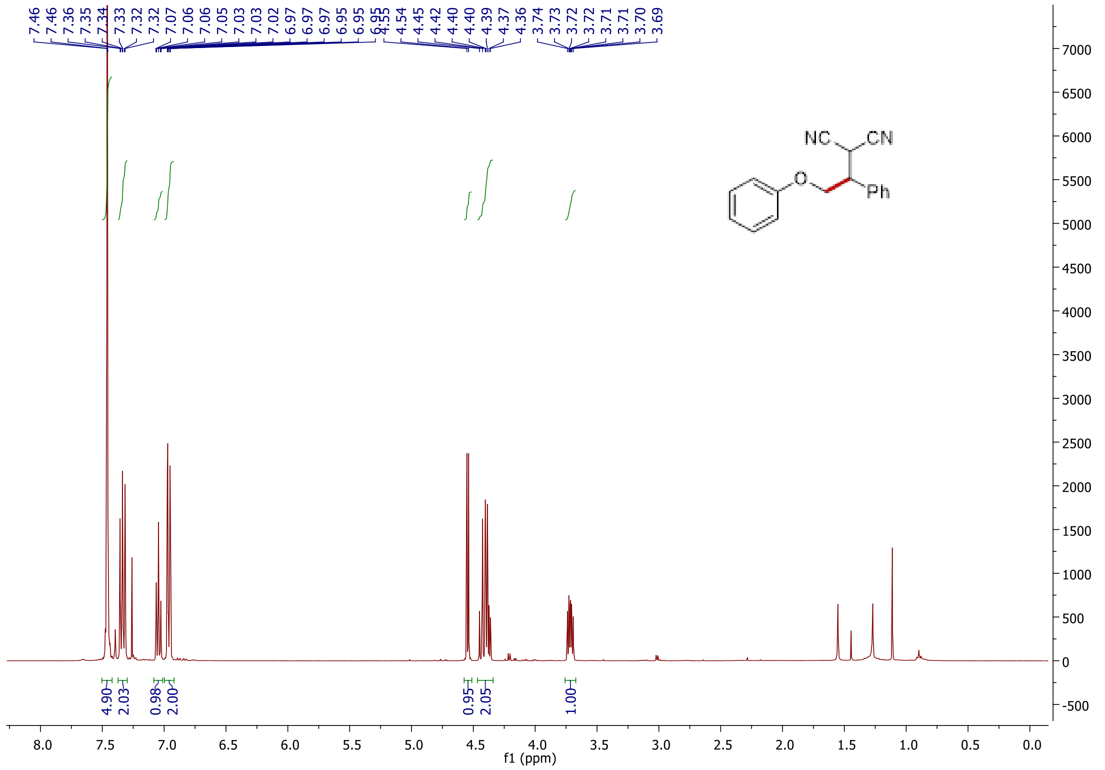

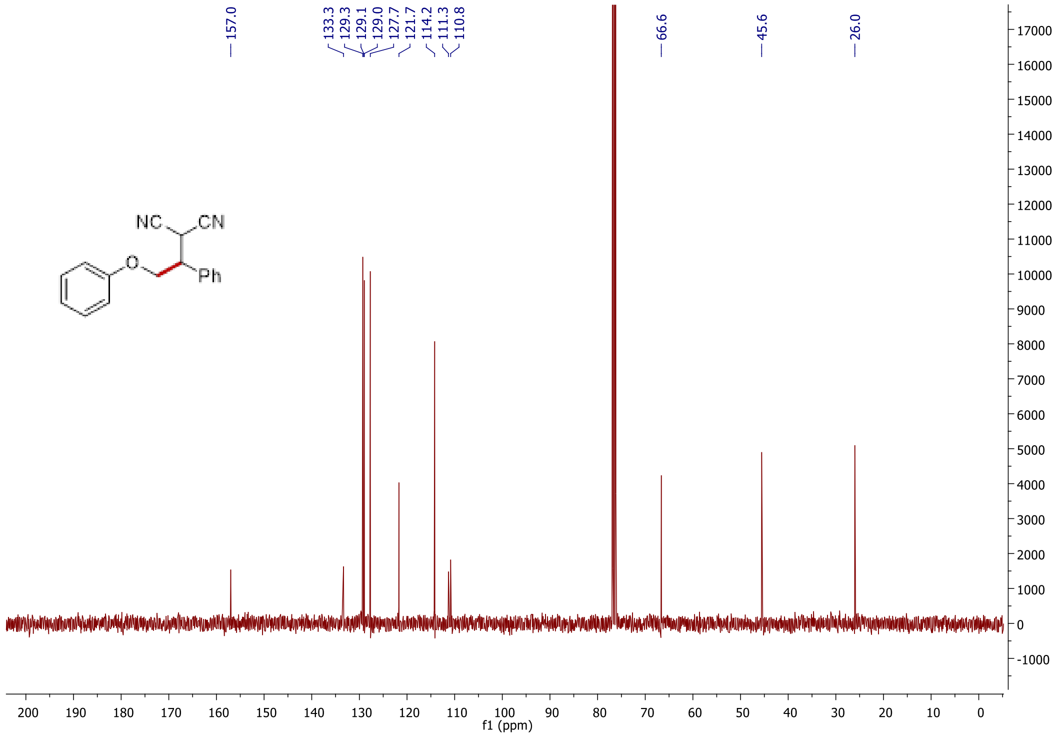

2-[2′-Phenoxy-1′-(4′′-methylphenyl)eth-1′-yl]malononitrile (22b)

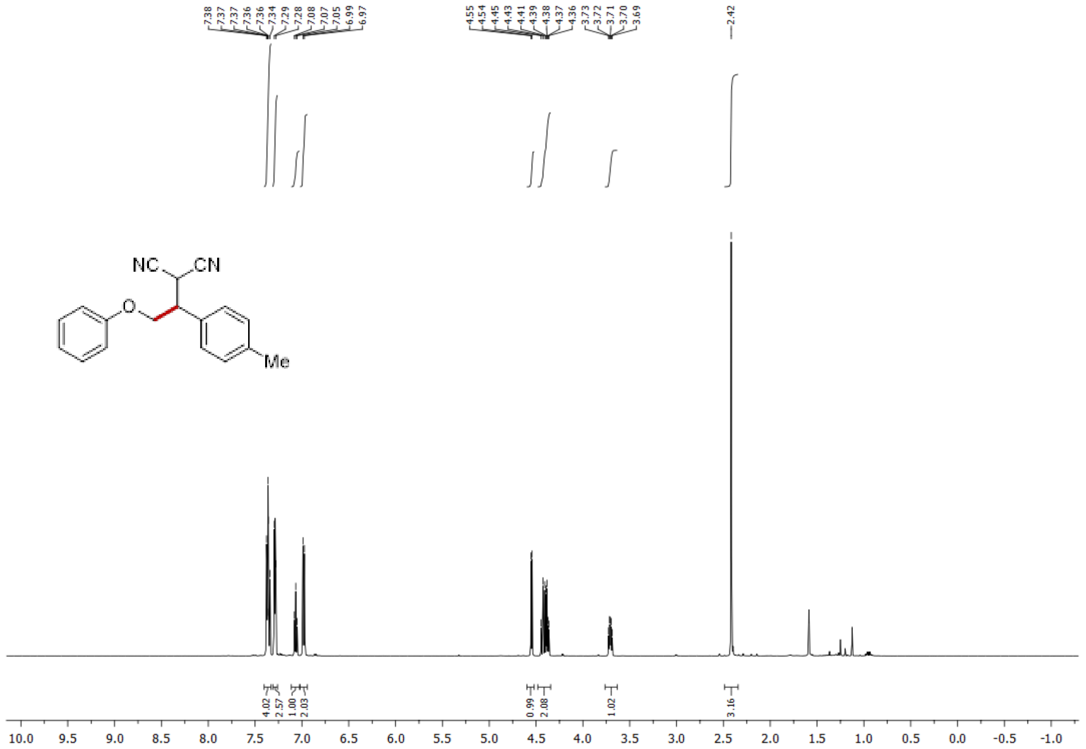

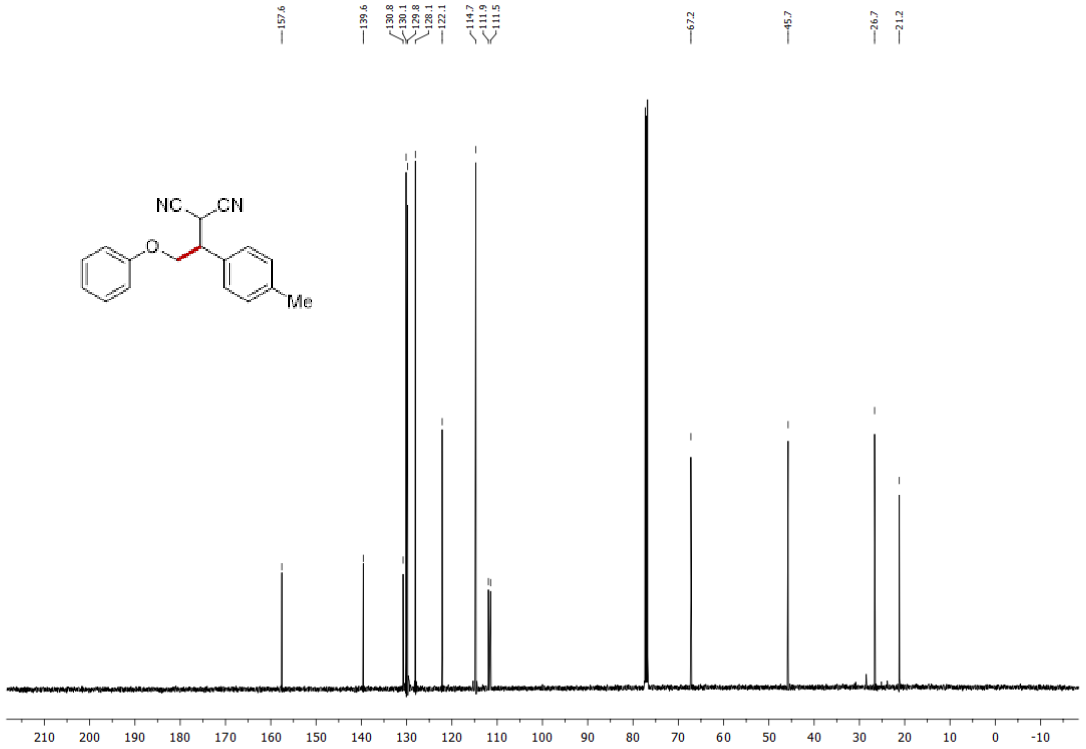

2-[2′-Phenoxy-1′-(4′′-fluorophenyl)eth-1′-yl]malononitrile (22c)

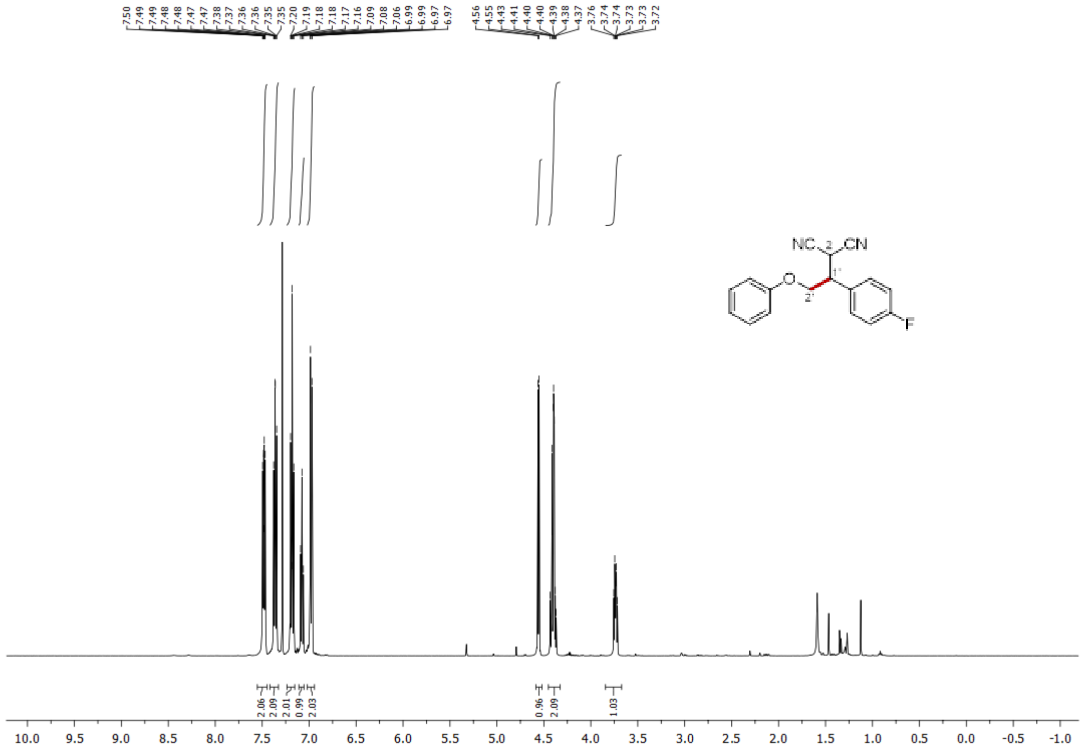

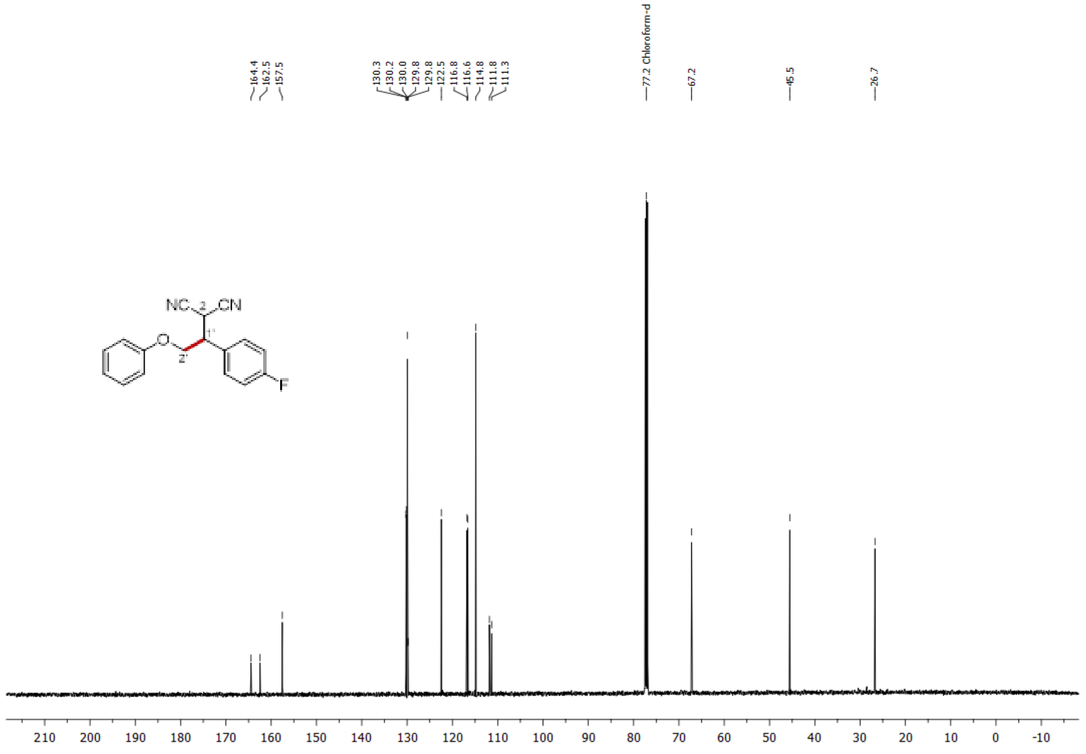

2-[2′-Phenoxy-1′-(4′′-chlorophenyl)eth-1′-yl]malononitrile (22d)

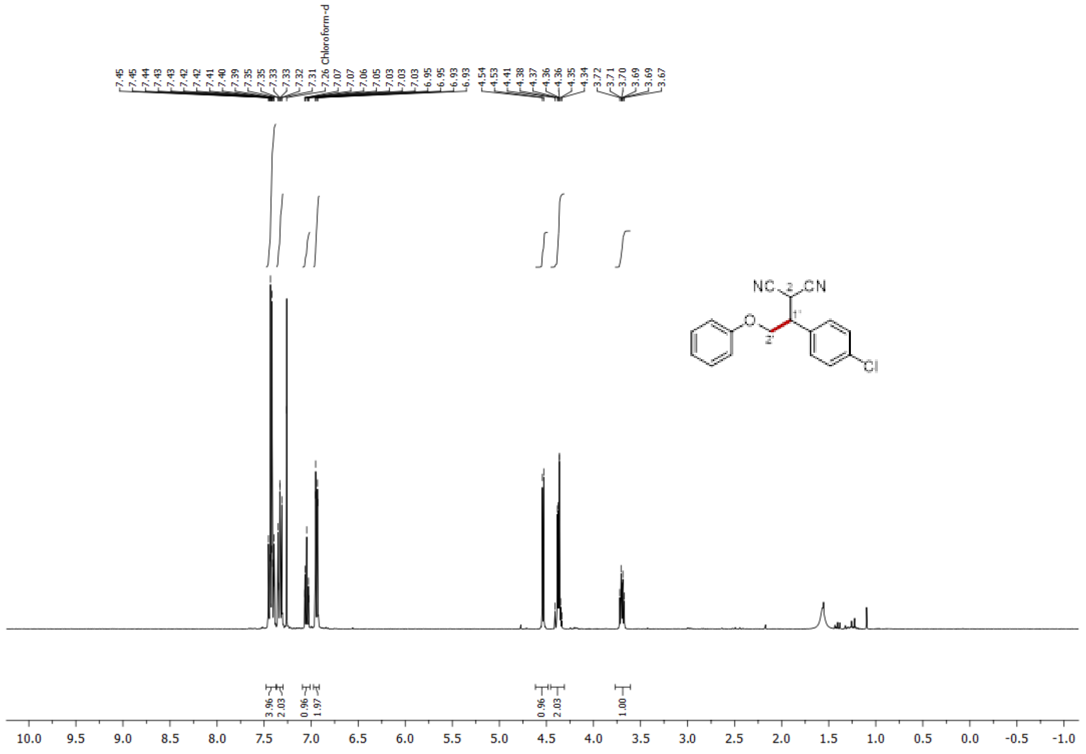

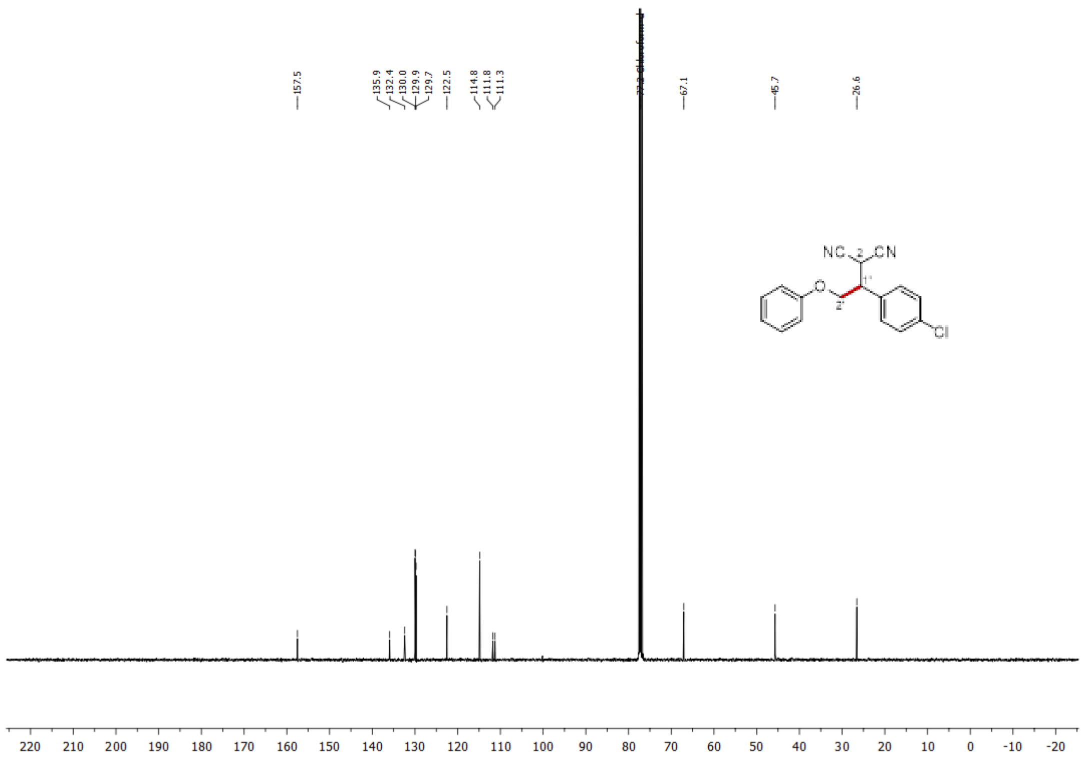

2-[2′-Phenoxy-1′-(4′′-bromophenyl)eth-1′-yl]malononitrile (22e)

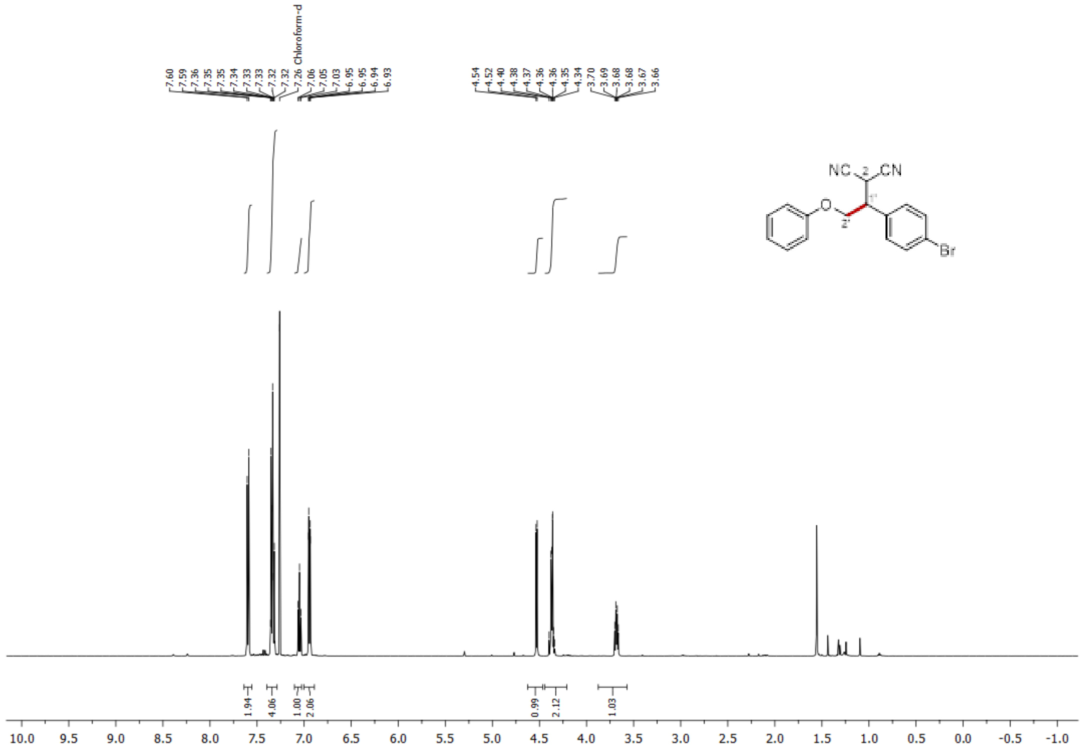

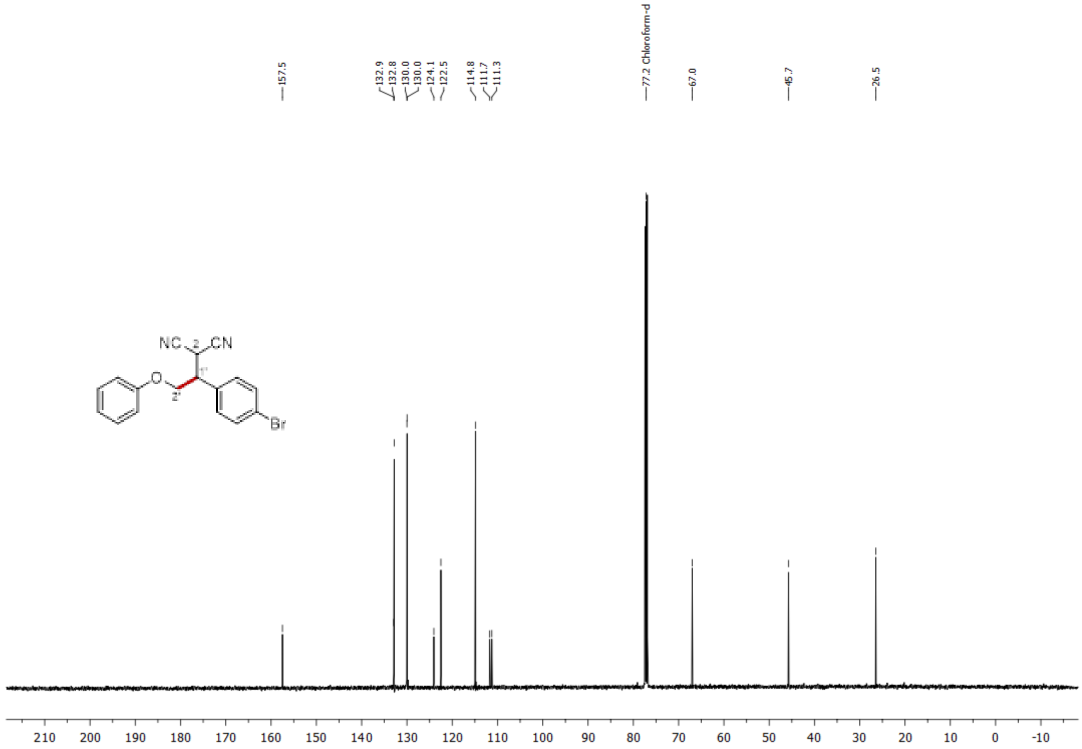

2-[2′-Phenoxy-1′-(4′′-iodophenyl)eth-1′-yl]malononitrile (22f)

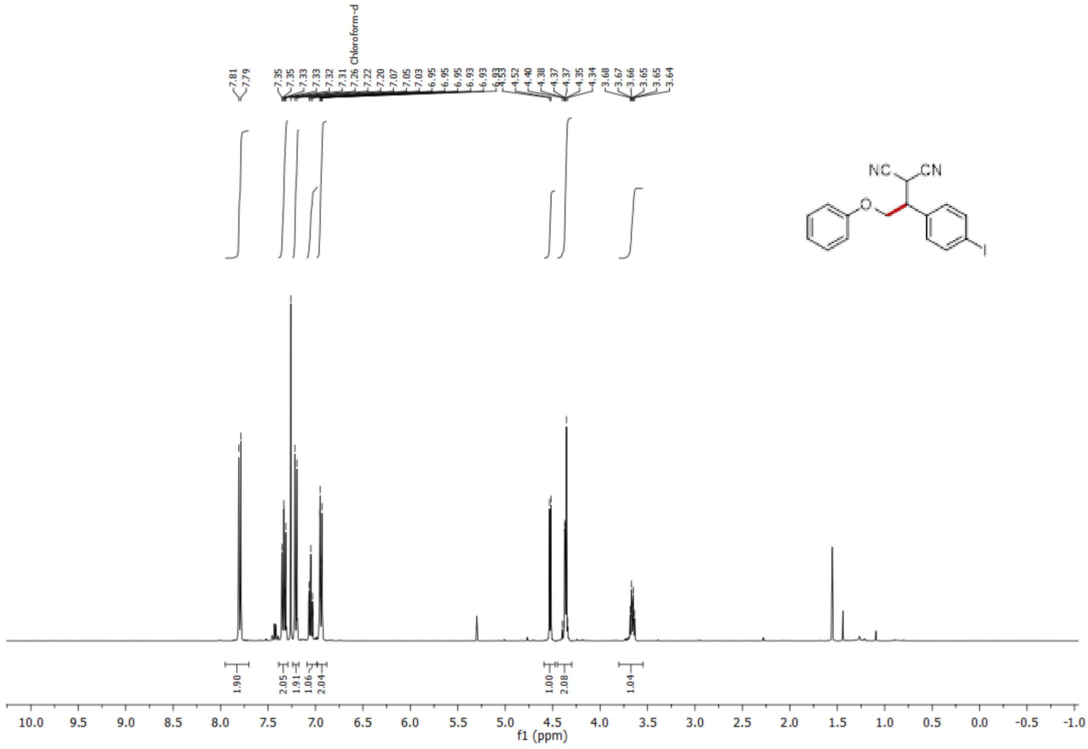

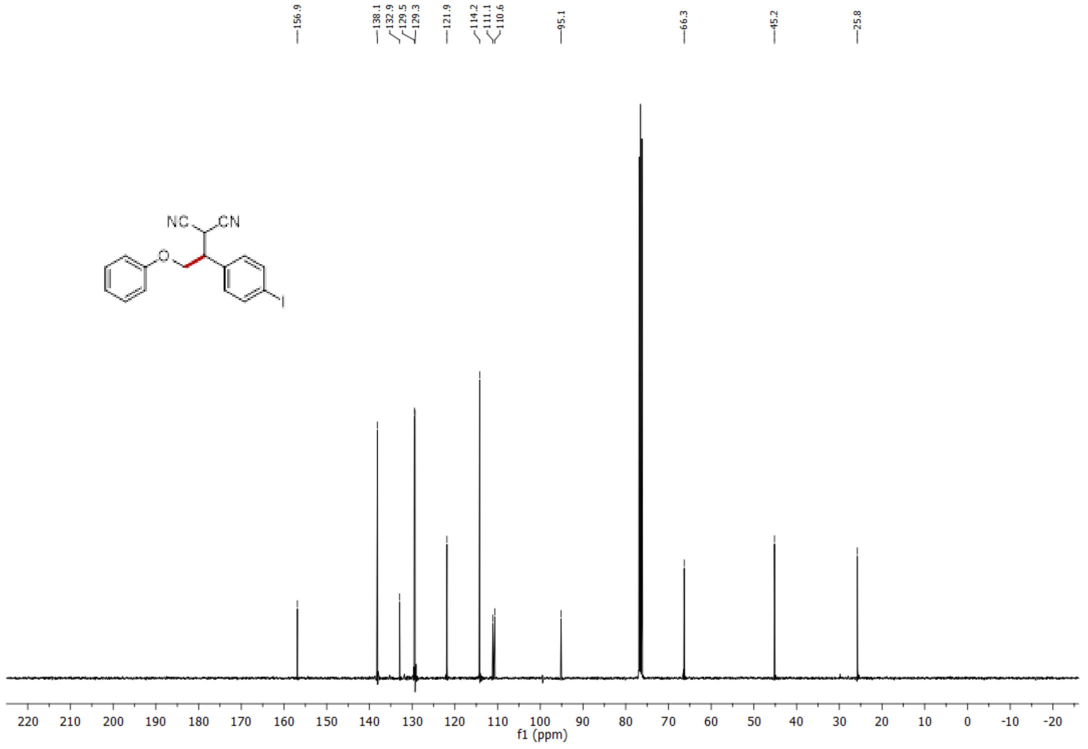

2-[2′-Phenoxy-1′-(4′′-methoxyphenyl)eth-1′-yl]malononitrile (22g)

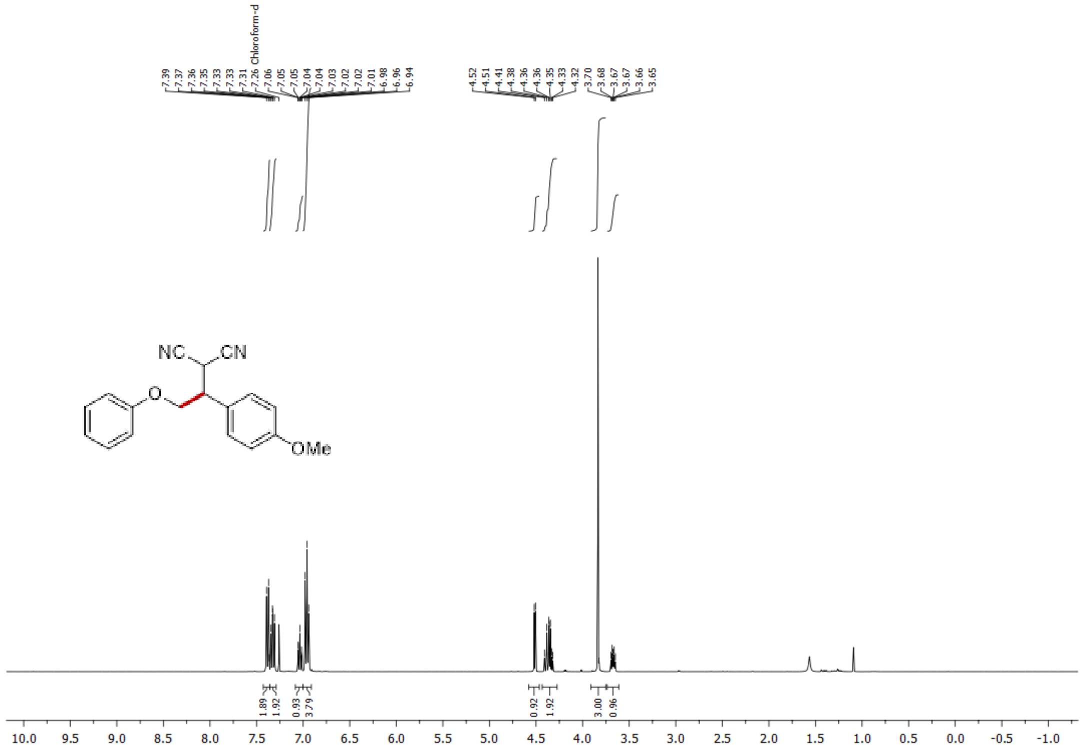

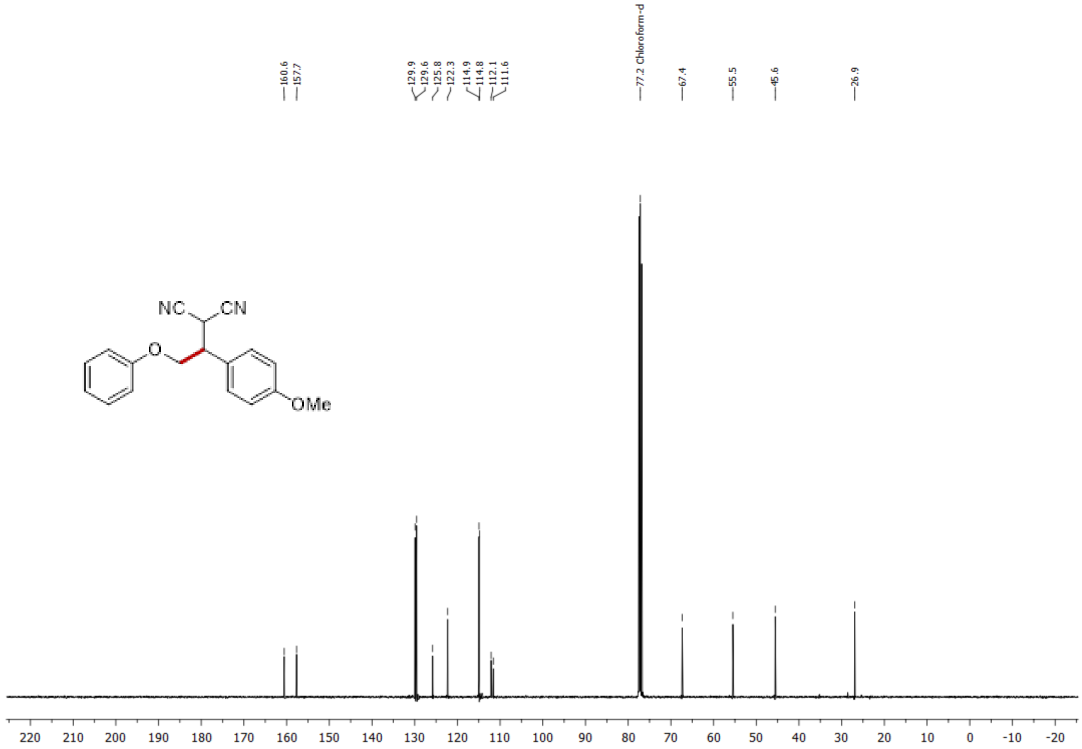

2-[2′-Phenoxy-1′-(4′′-nitrophenyl)eth-1′-yl]malononitrile (22h)

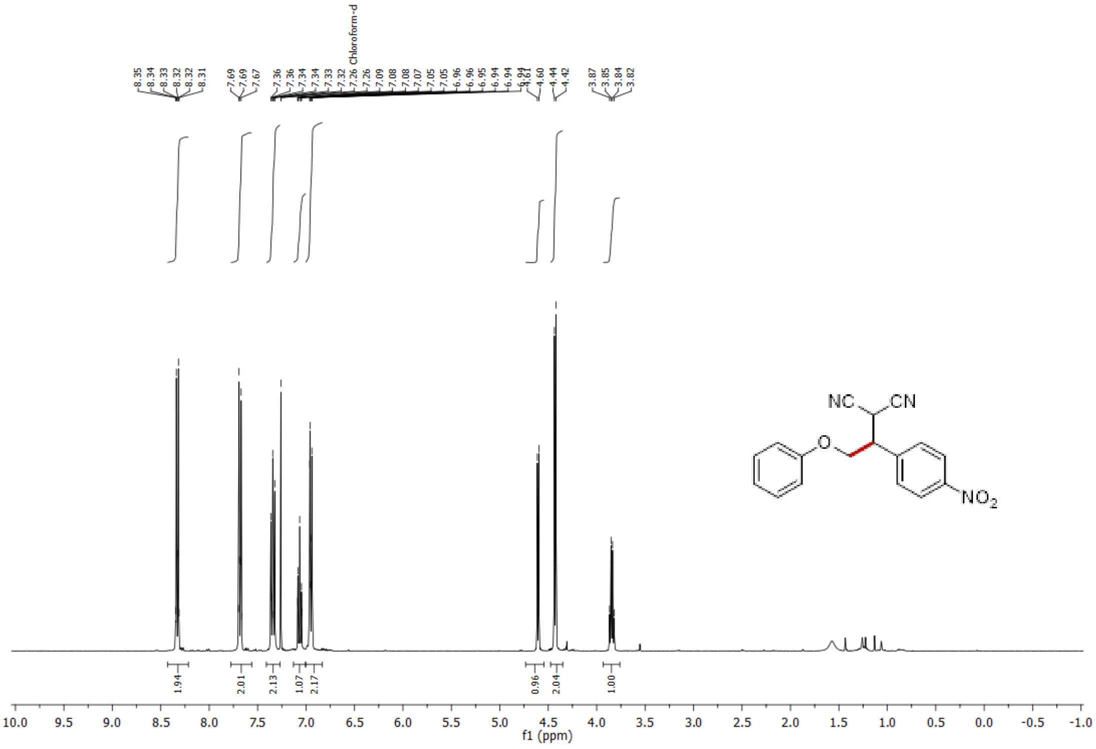

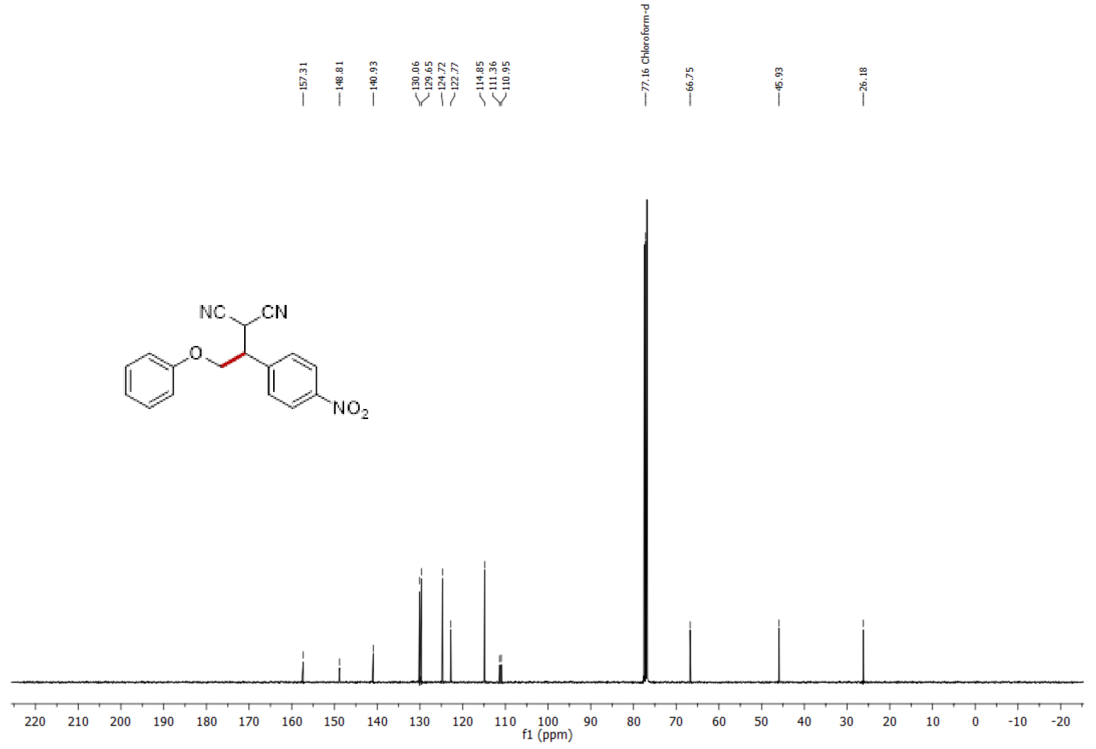

2-[2′-(3′′-Methylphenyl)-1′-phenyleth-1′-yl]malononitrile (22i)

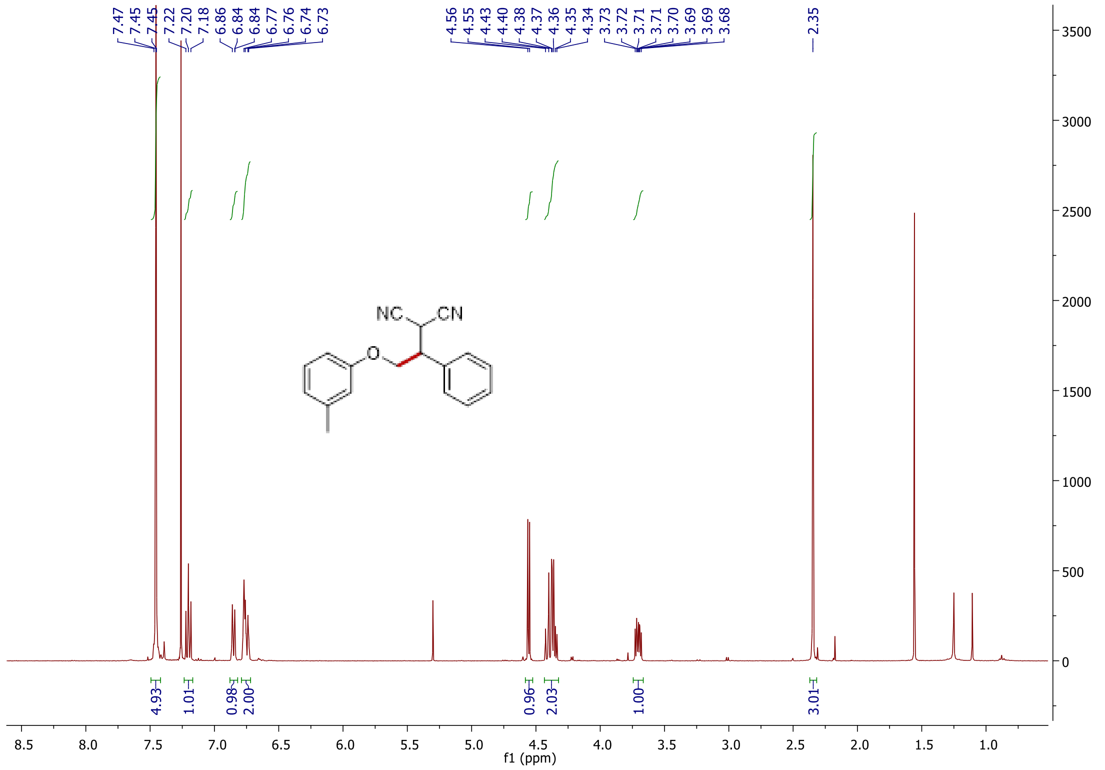

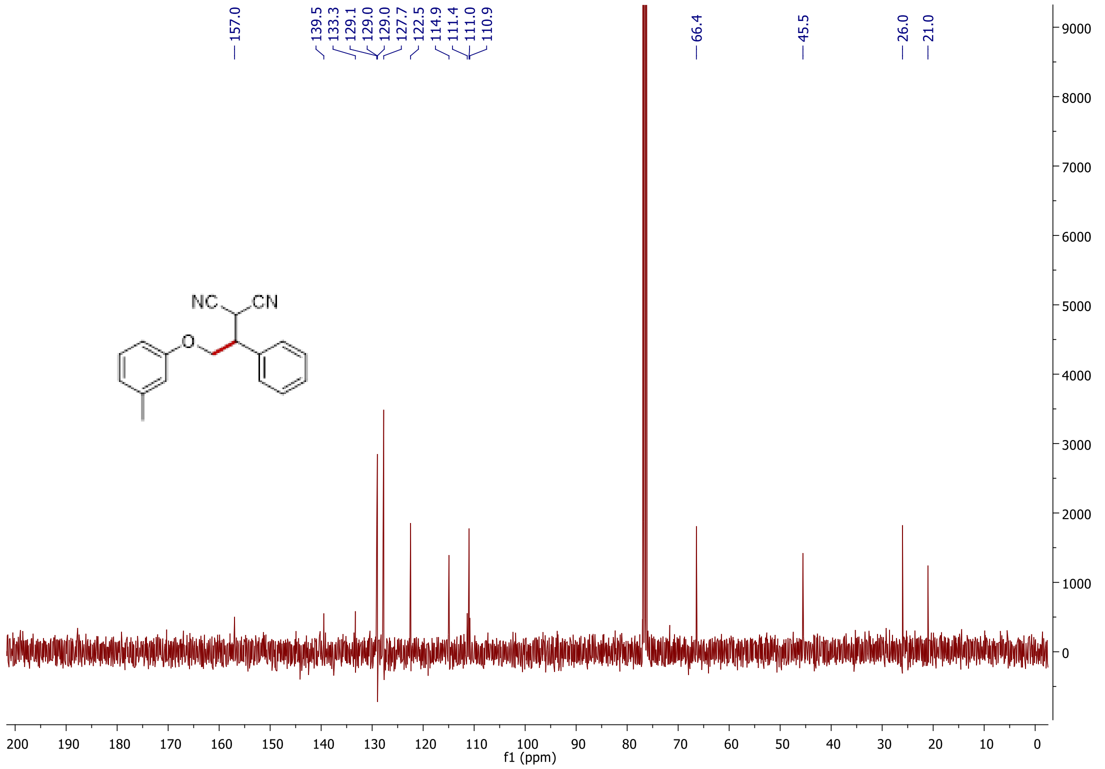

2-[2′-(3′′-Methylphenyl)-1′-(4′′′-methylphenyl)eth-1′-yl]malononitrile (22j)

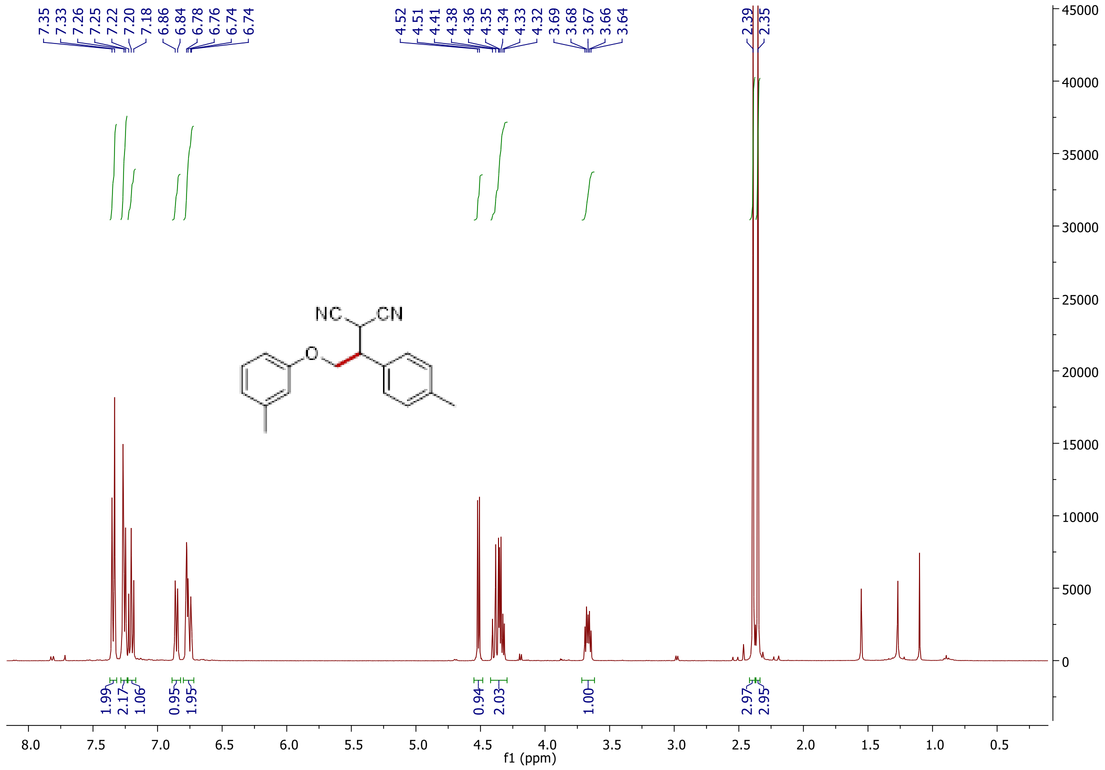

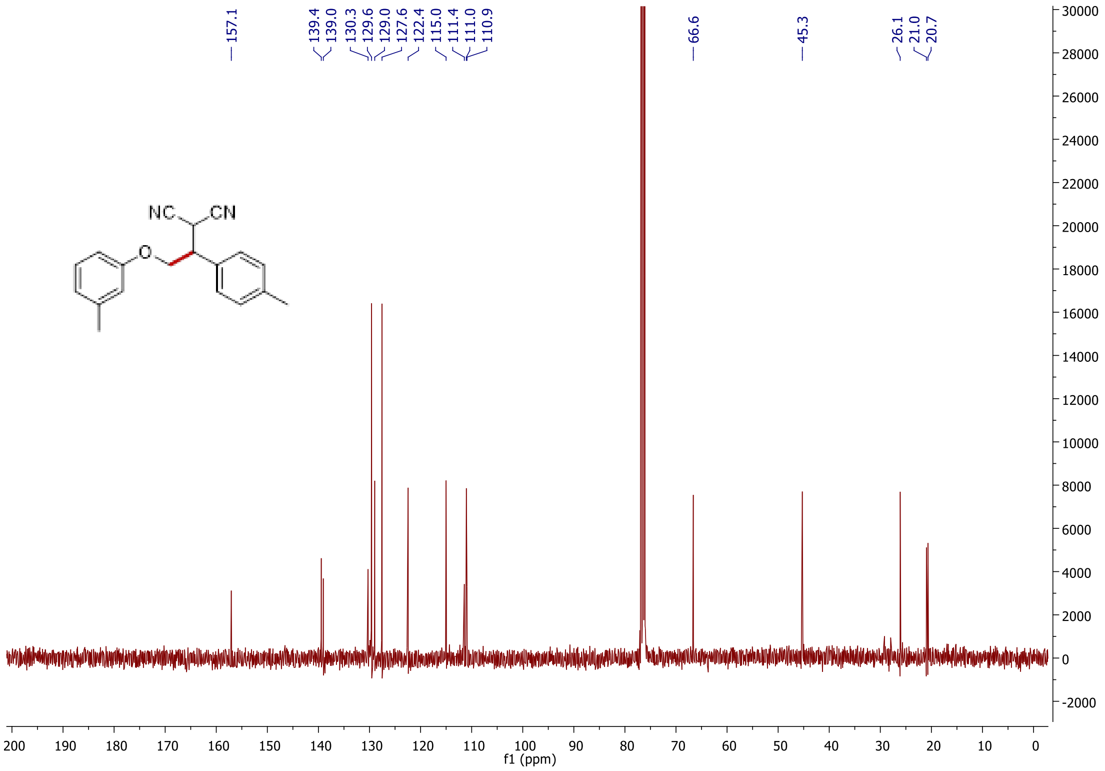

2-[2′-(3′′-Methylphenyl)-1′-(4′′′-chlorophenyl)eth-1′-yl]malononitrile (22k)

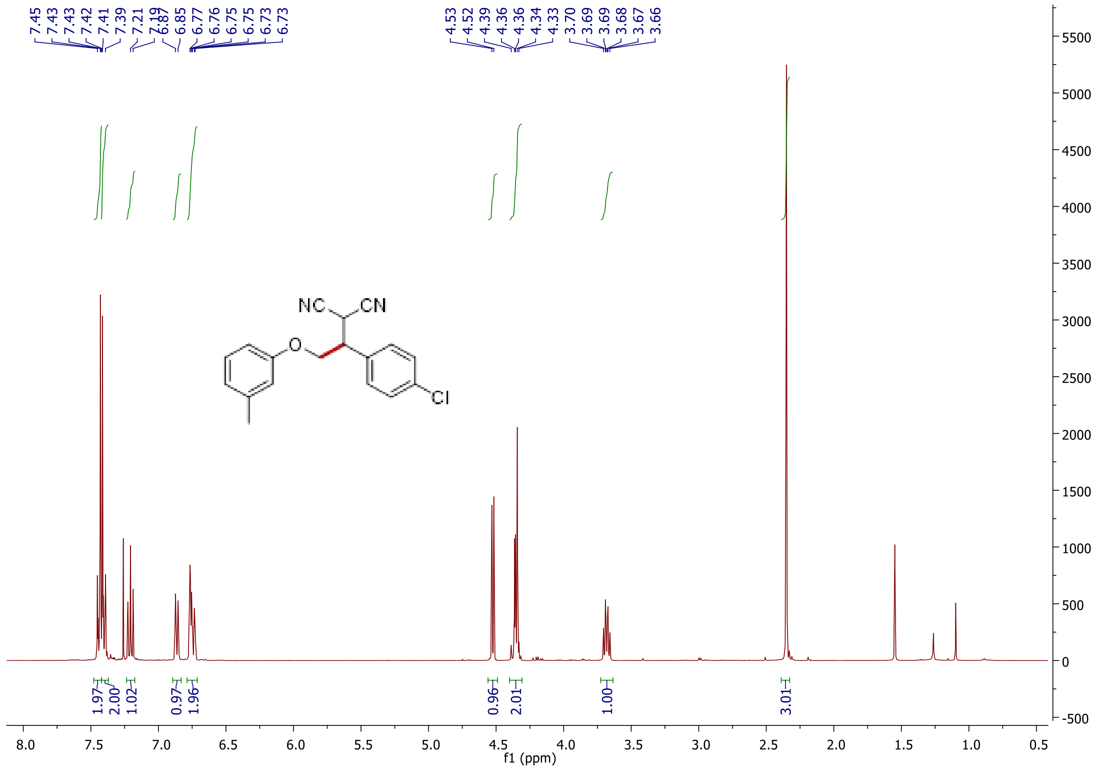

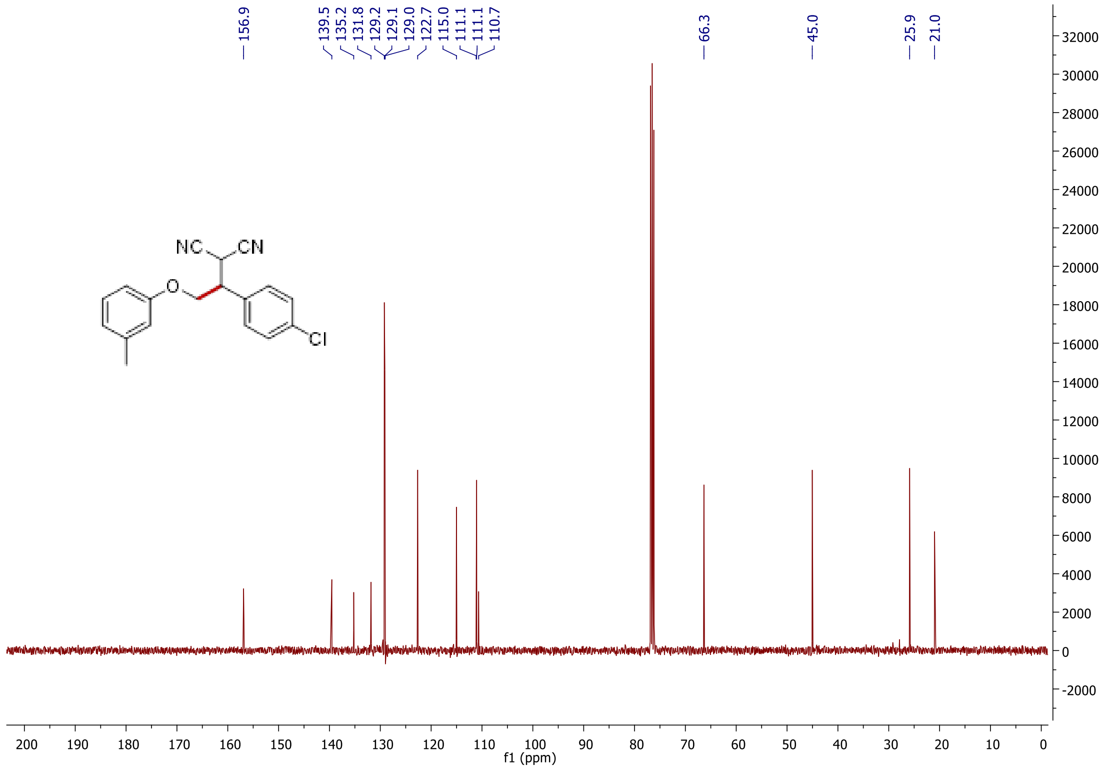

2-[2′-(4″-Fluorophenyl)-1′-phenyleth-1′-yl]malononitrile (22l)

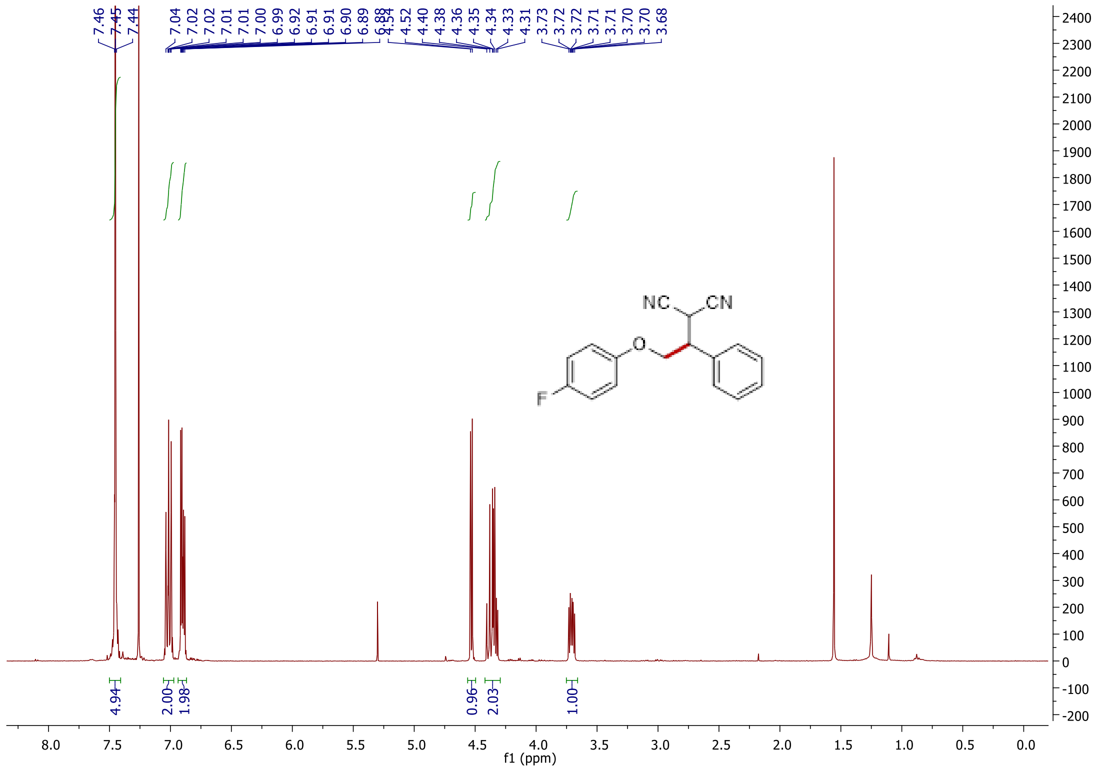

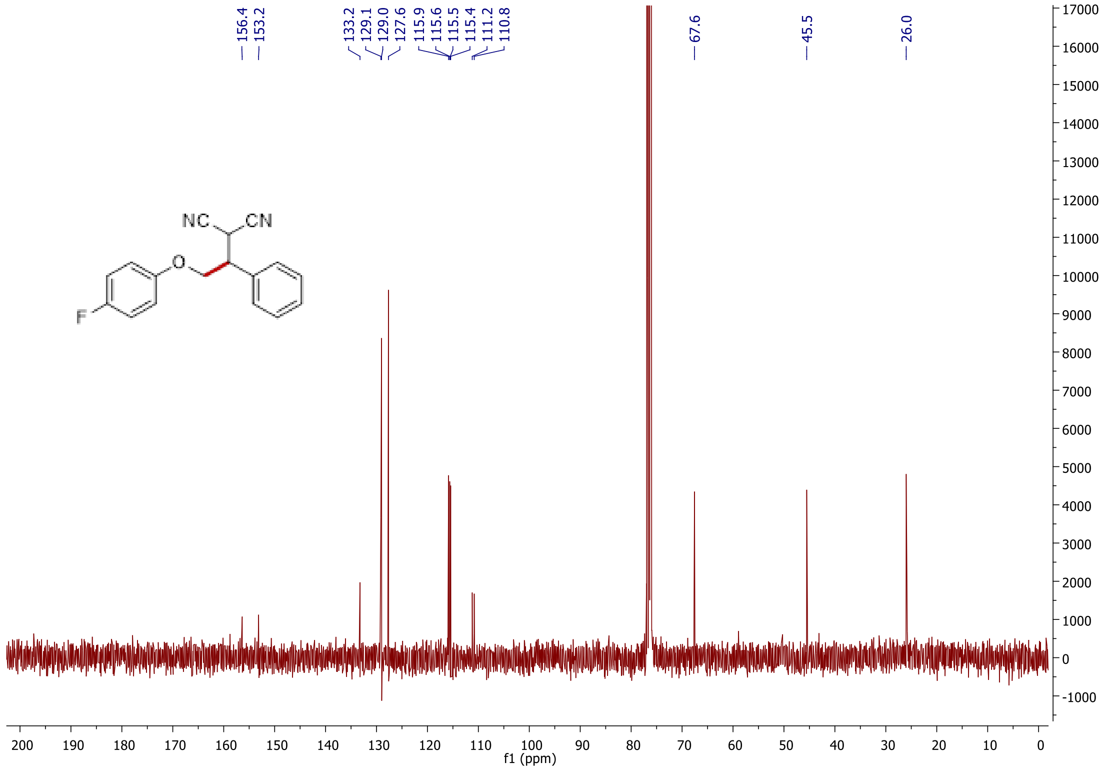

2-[2′-(4′′-Fluorophenyl)-1′-(4′′′-methylphenyl)eth-1′-yl]malononitrile (22m)

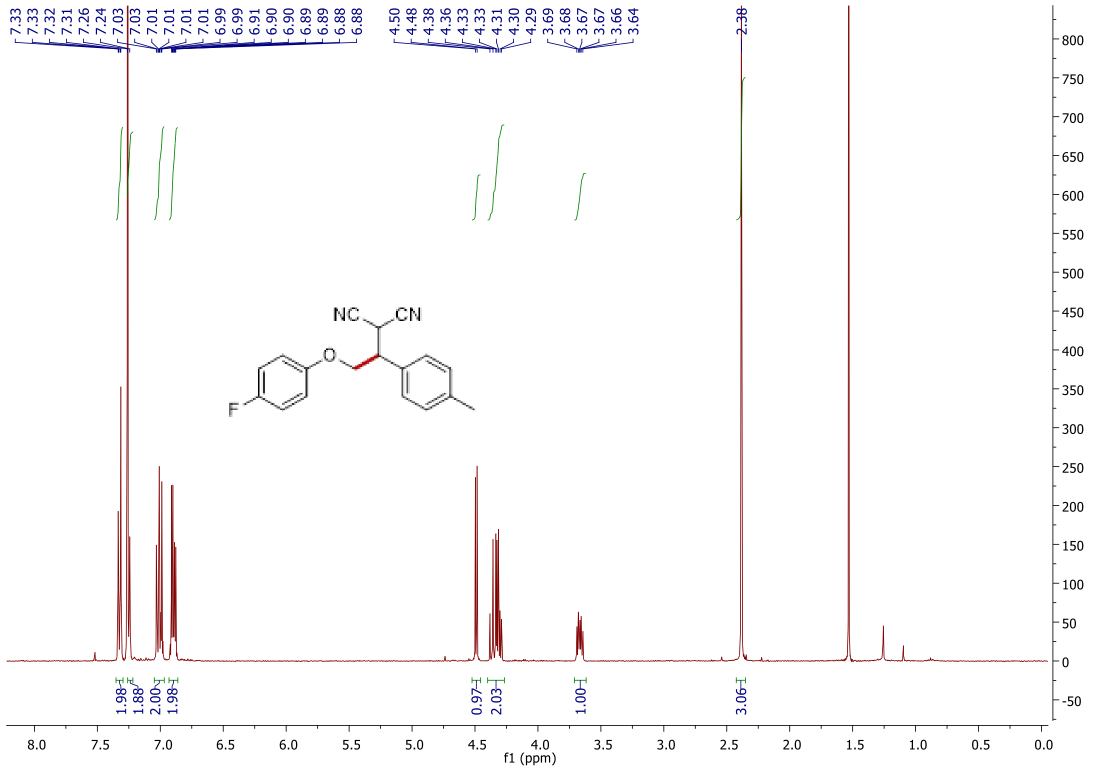

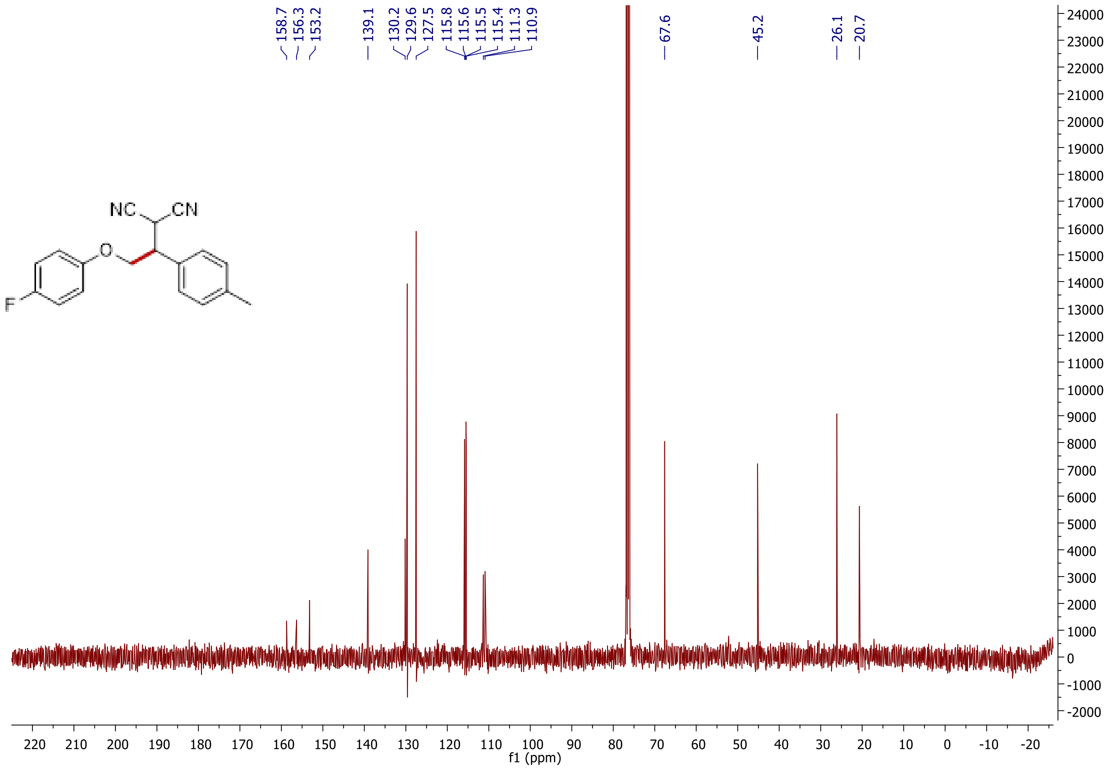

2-[2′-(4′′-Fluorophenyl)-1′-(4′′′-chlorophenyl)eth-1′-yl]malononitrile (22n)

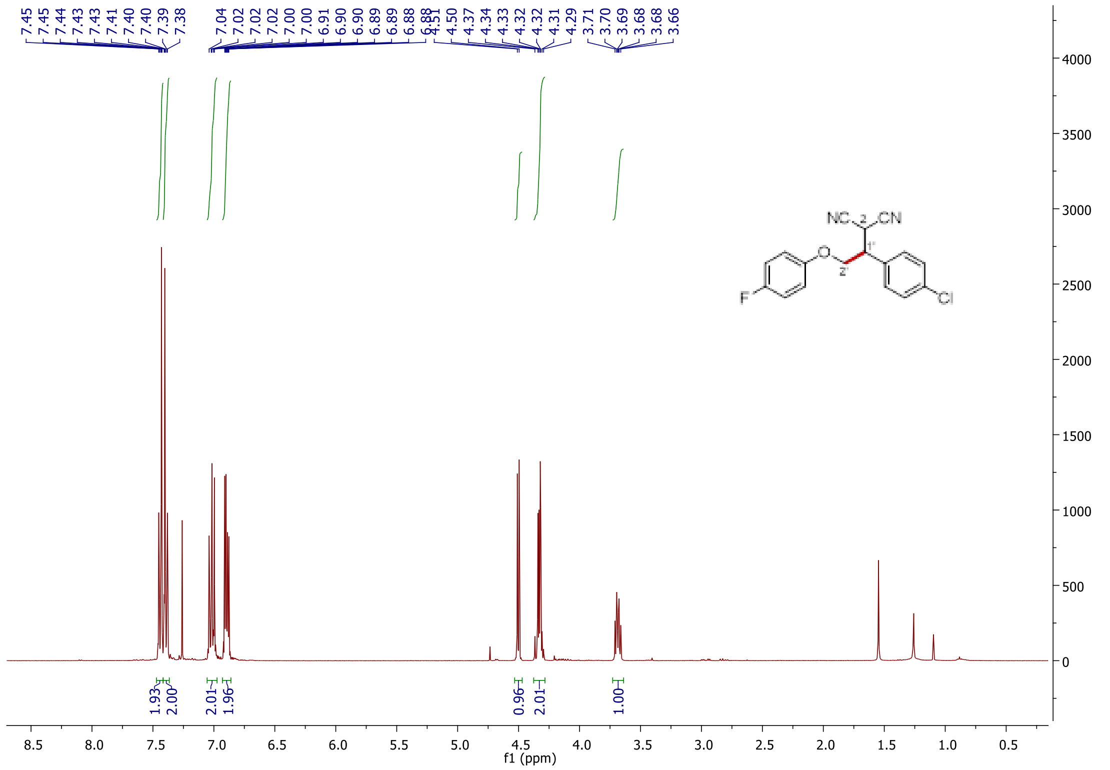

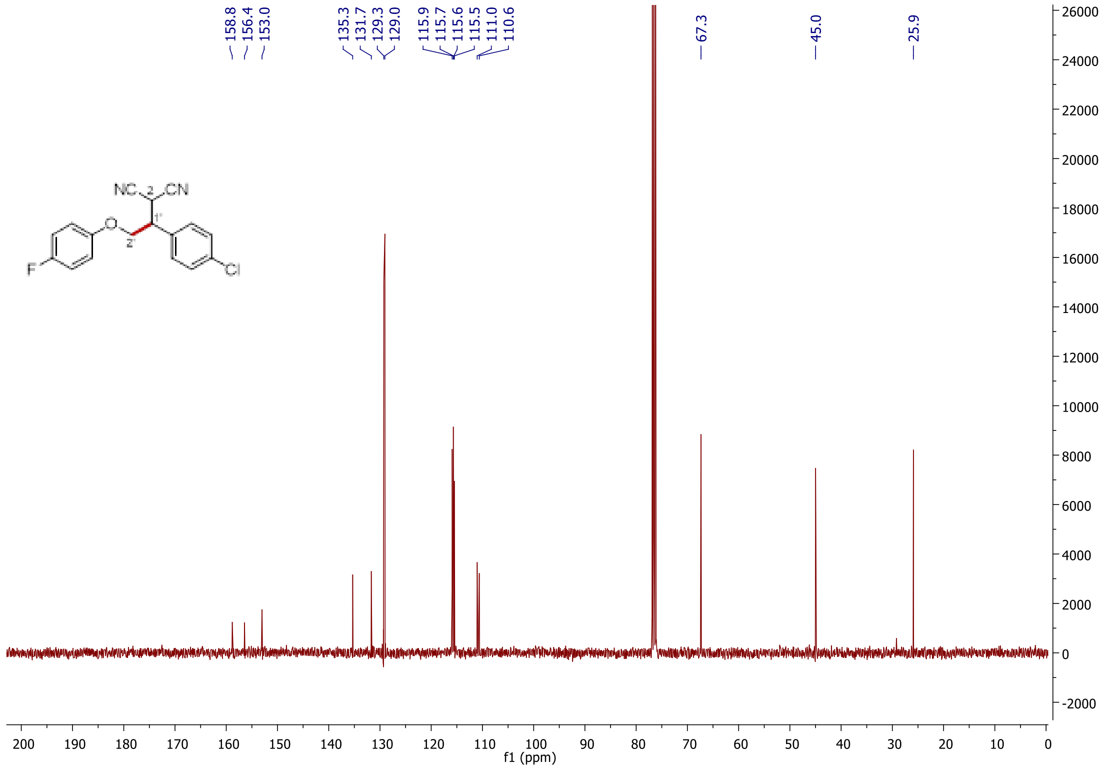

2-[2′-(4′′-Chlorophenyl)-1′-phenyleth-1′-yl]malononitrile (22o)

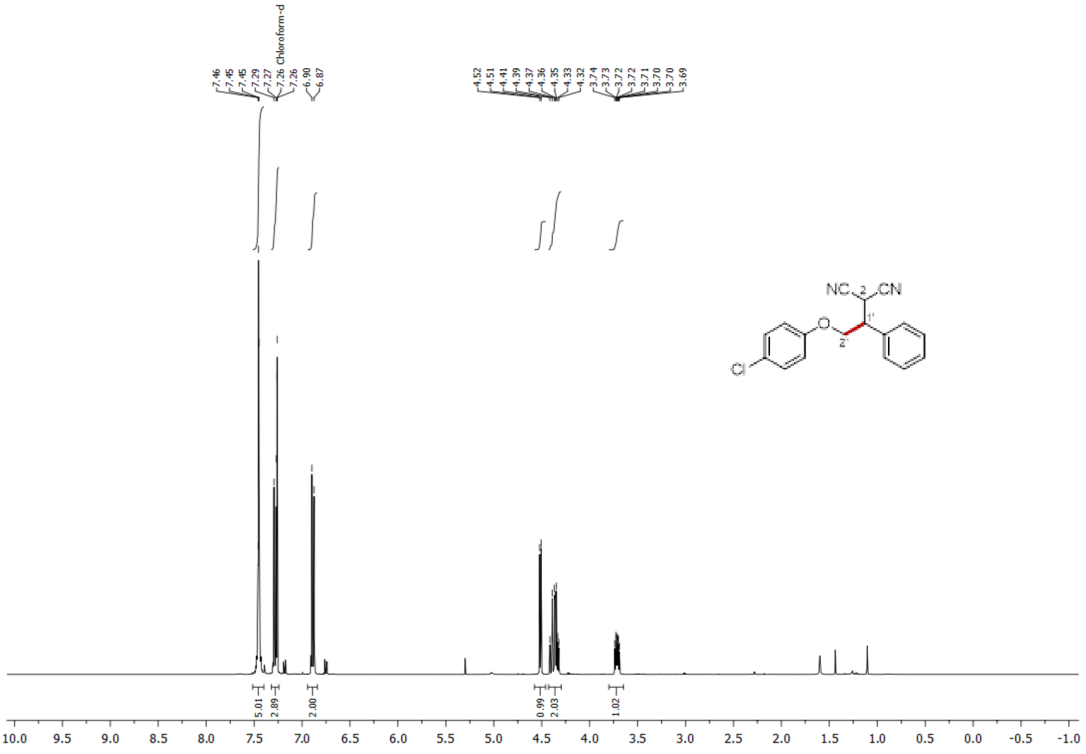

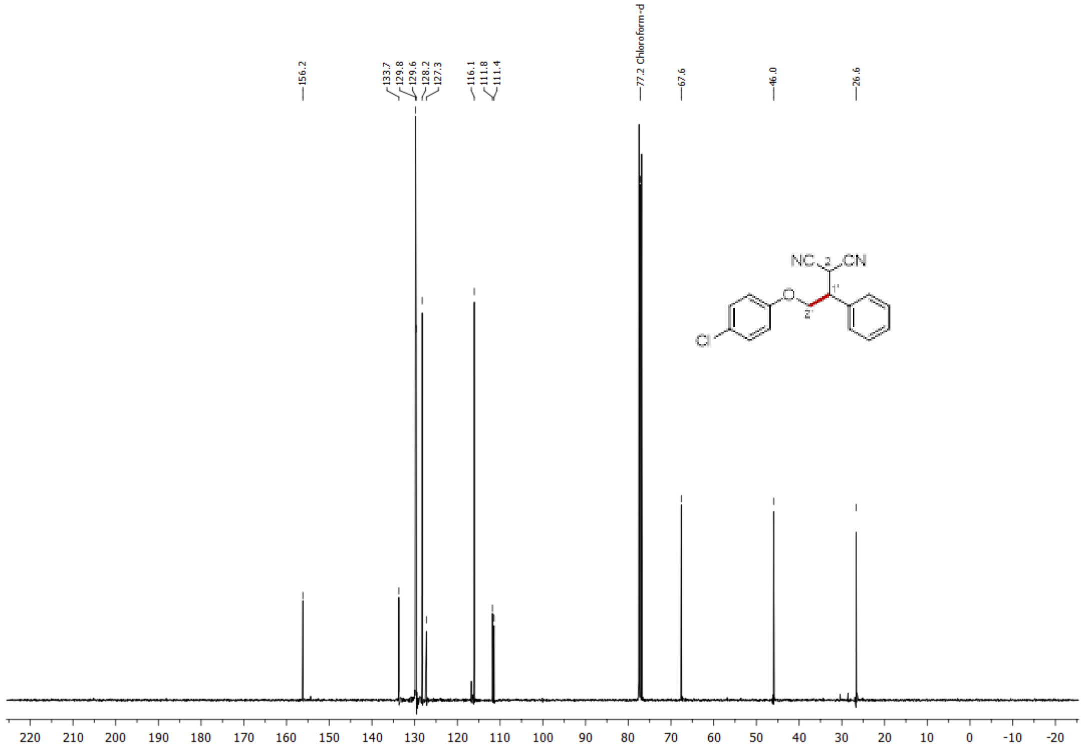

2-[2′-(4′′-Chlorophenyl)-1′-(4′′′-bromophenyl)eth-1′-yl]malononitrile (22p)

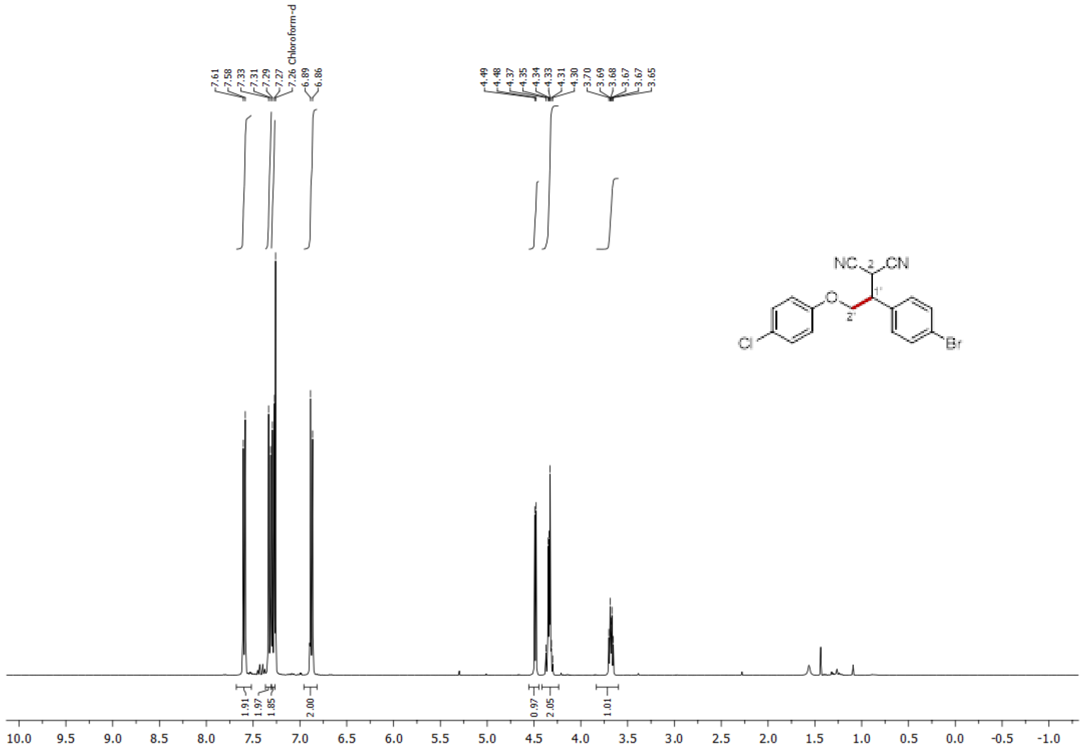

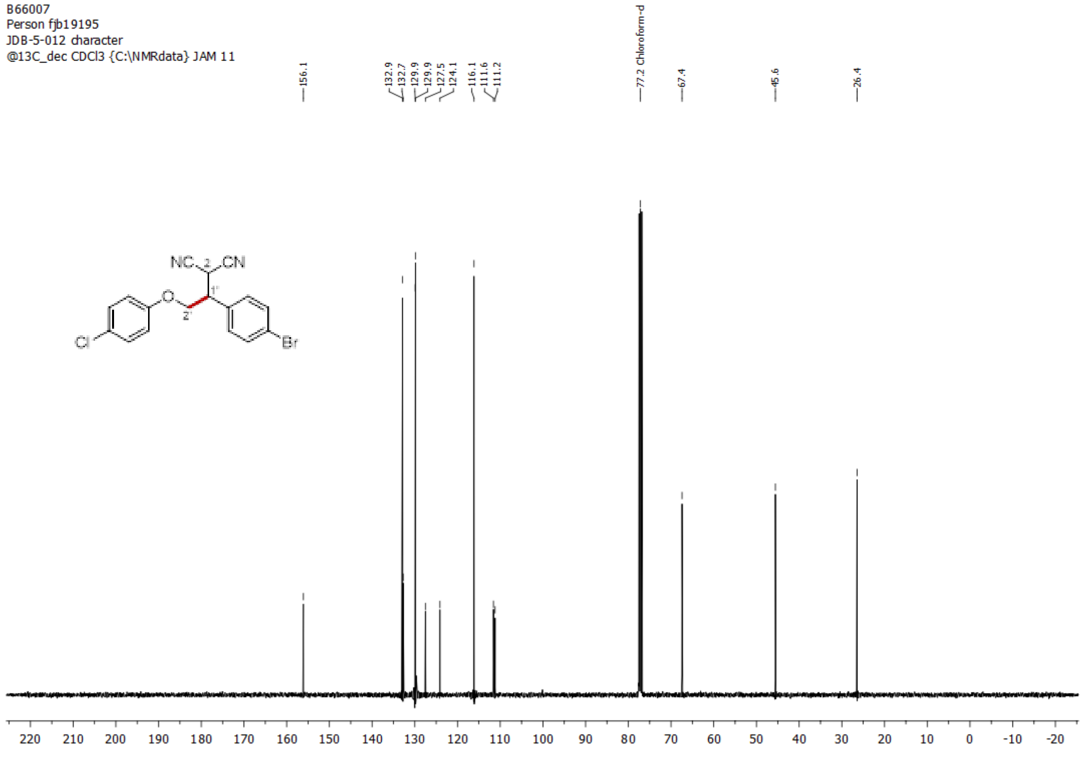

2-[2′-(4′′-Chlorophenyl)-1′-(4′′′-iodophenyl)eth-1′-yl]malononitrile (22q)

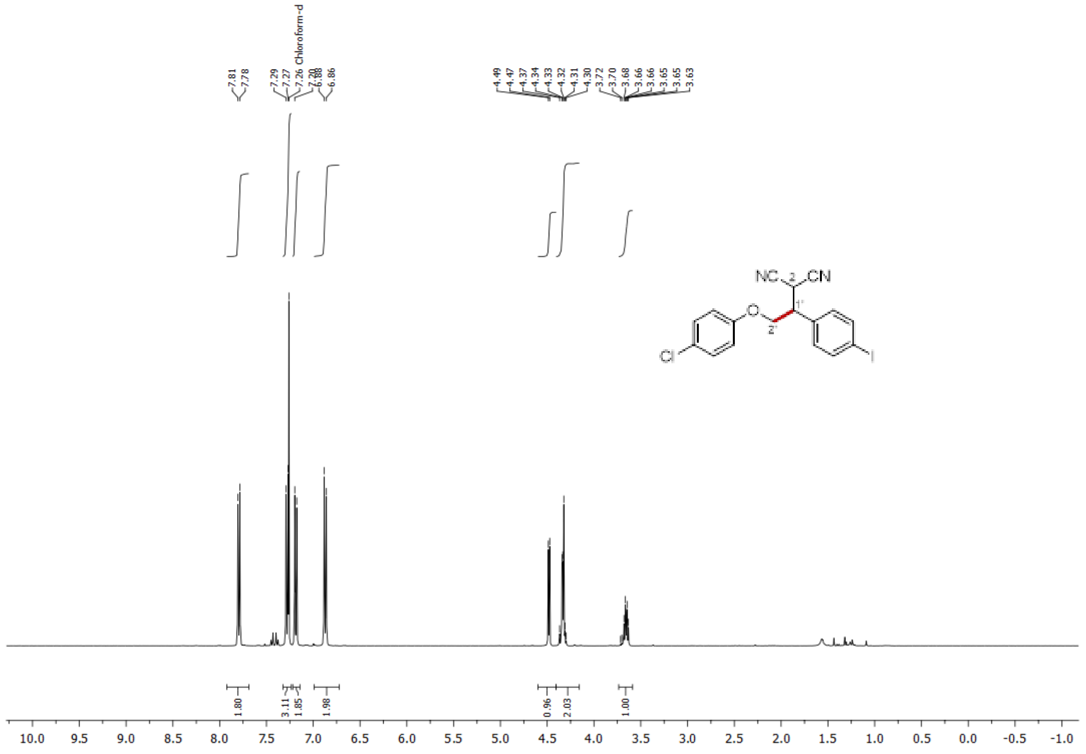

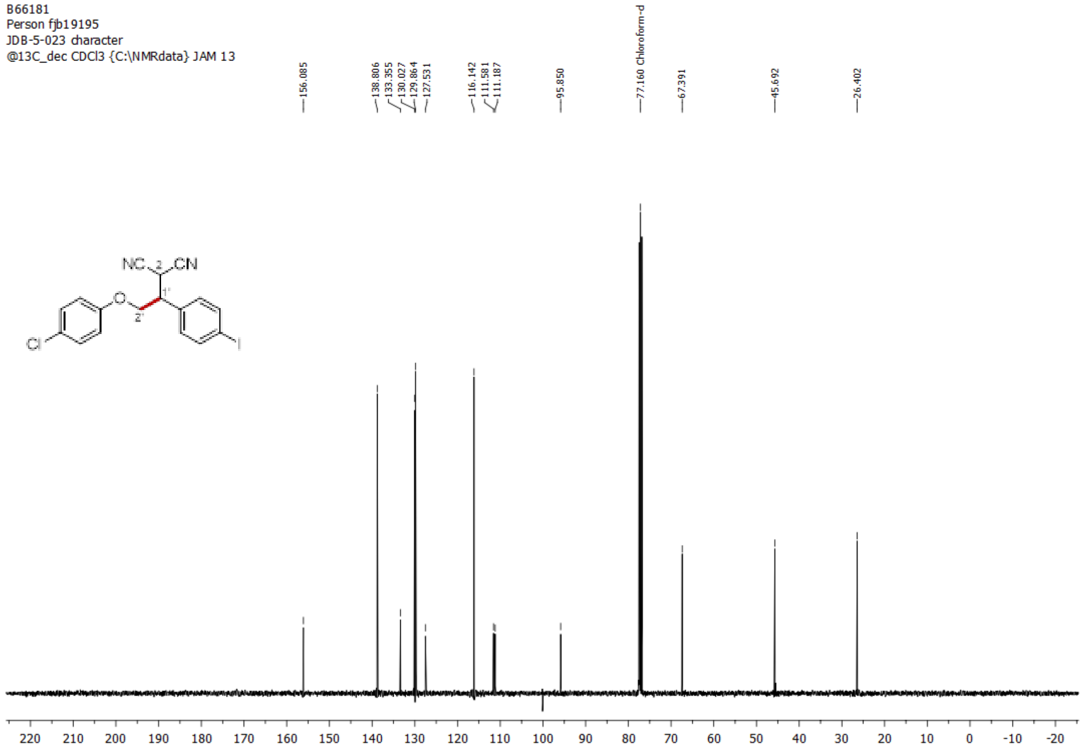

2-[2′-(4′′-Chlorophenyl)-1′-(4′′′-methoxyphenyl)eth-1′-yl]malononitrile (22r)

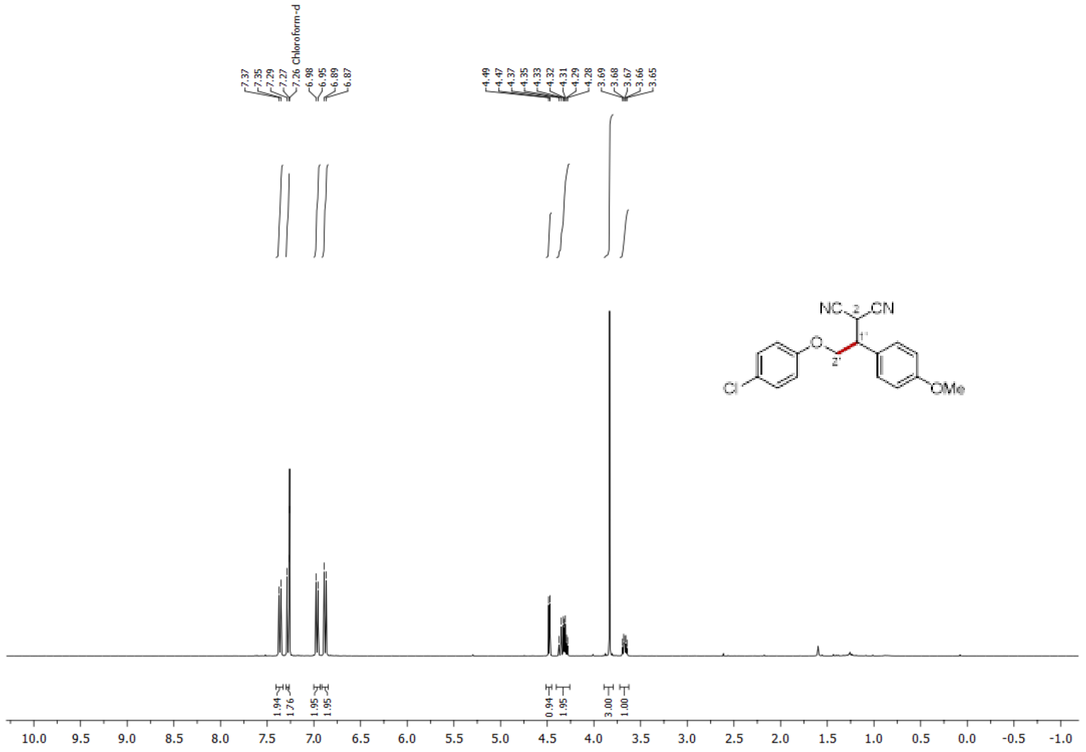

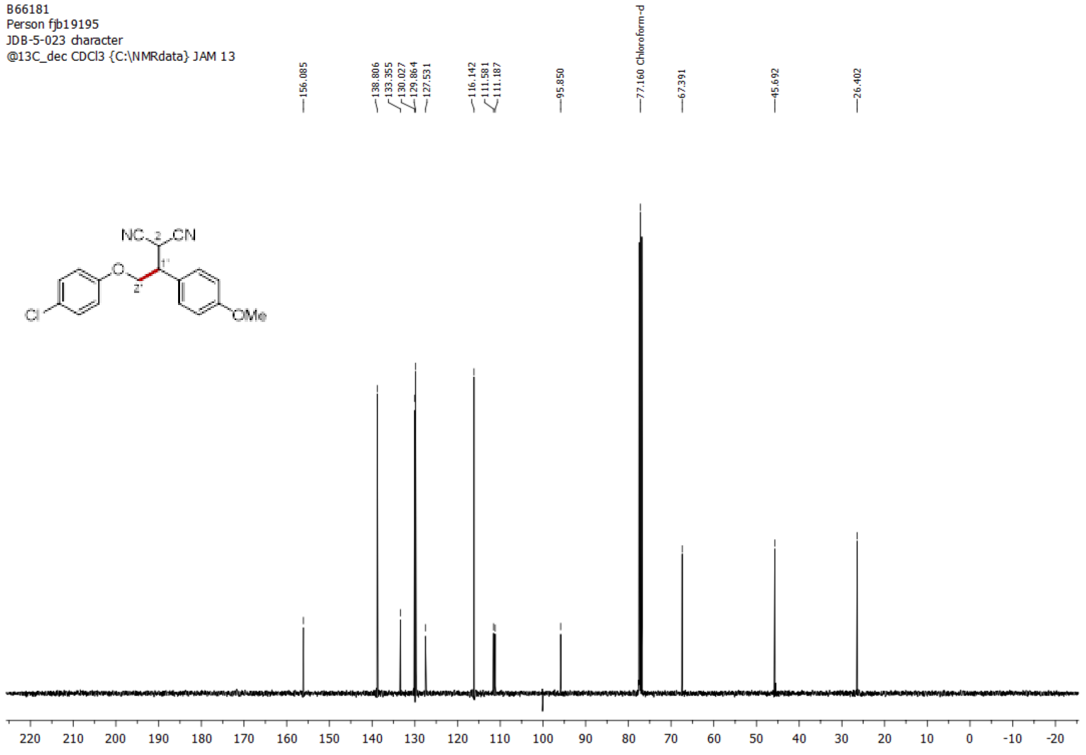

2-(2′-Methyl-2′-phenoxy-1′-phenylprop-2′-yl)malononitrile (22s)

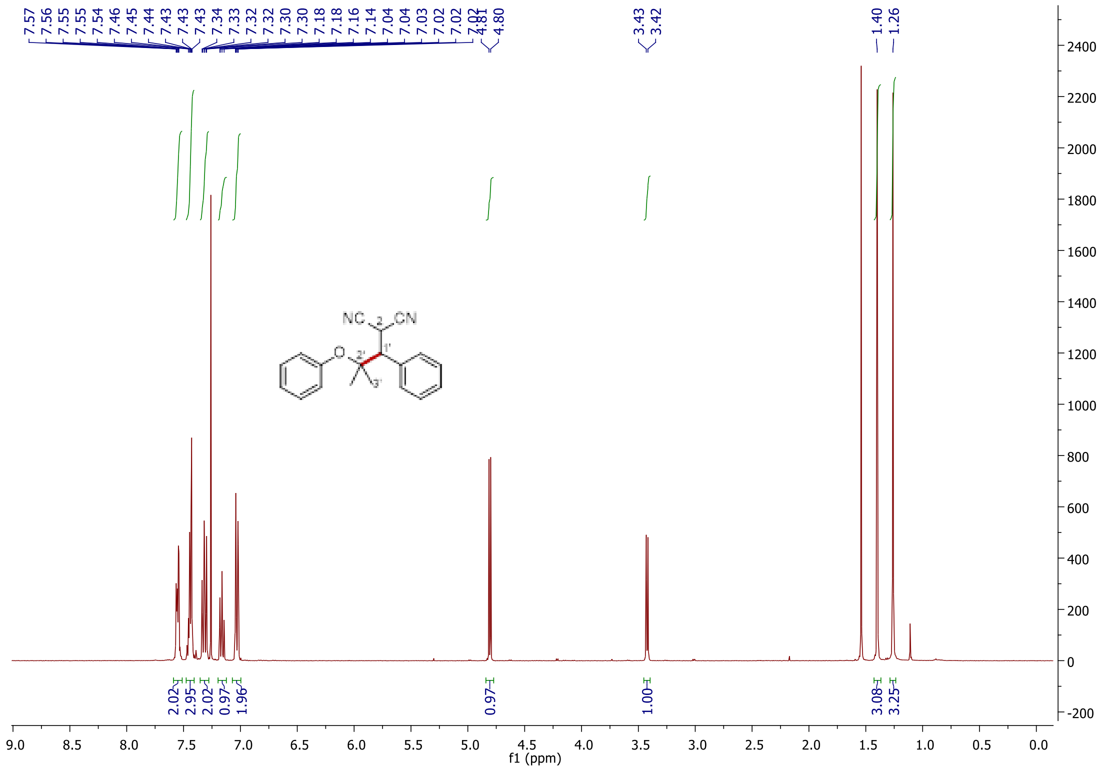

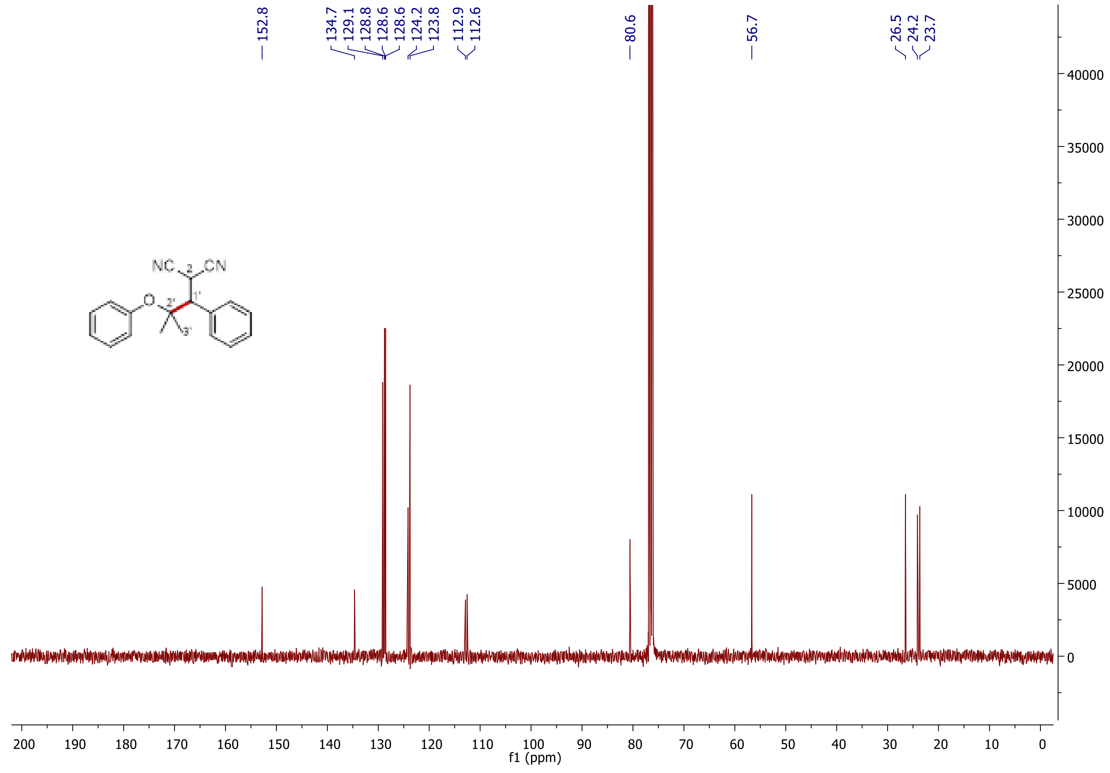

2-[2′-Methyl-2′-phenoxy-1′-(4′′-iodophenyl)prop-2′-yl]malononitrile (22t)

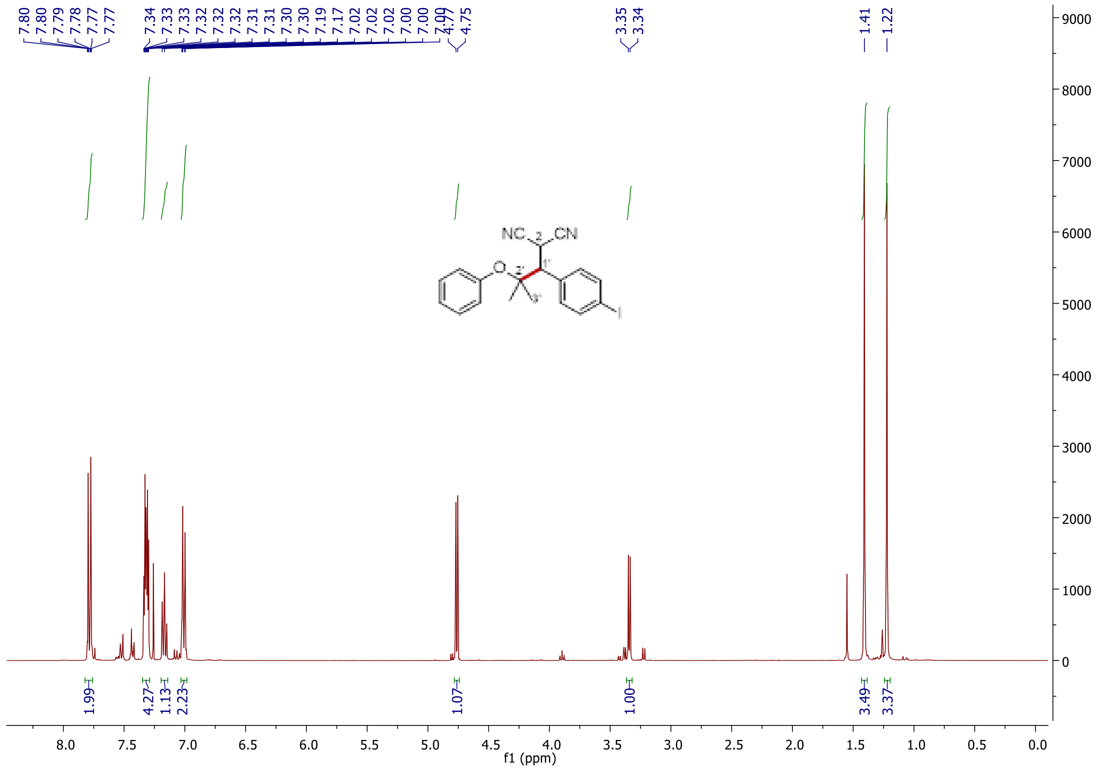

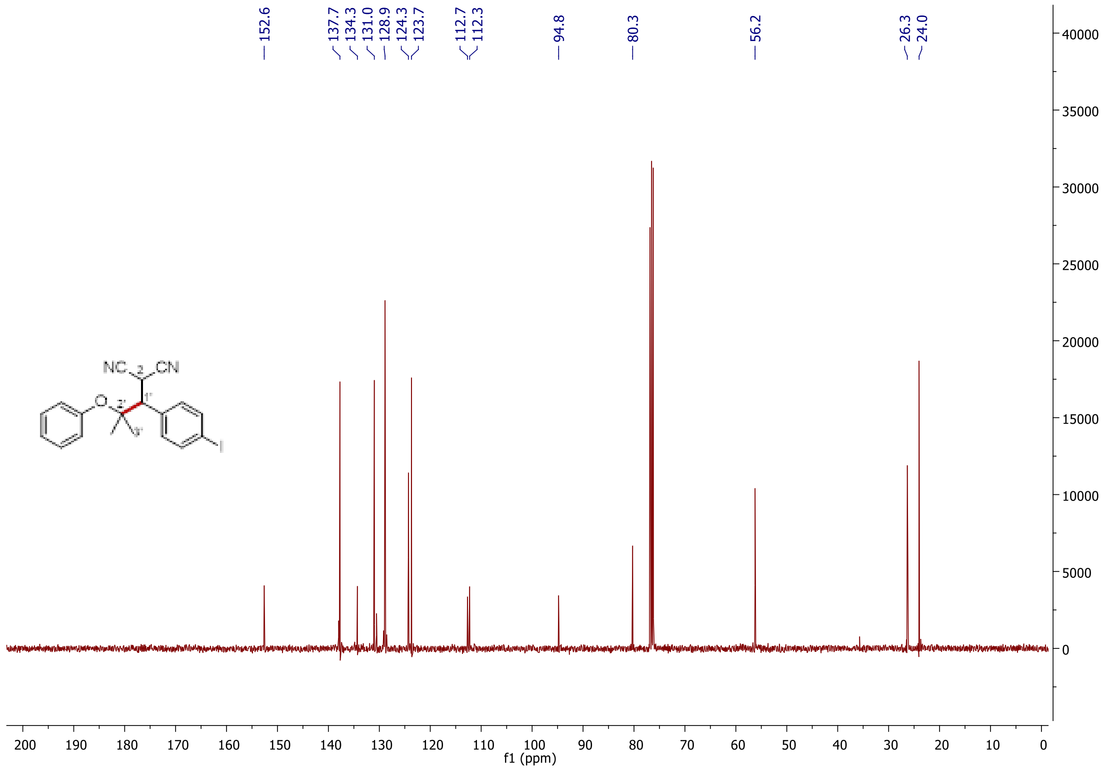

2-[(2′-Methyl-2′-phenoxy-1′-(4′′-methoxyphenyl)prop-2′-yl]malononitrile (22u)

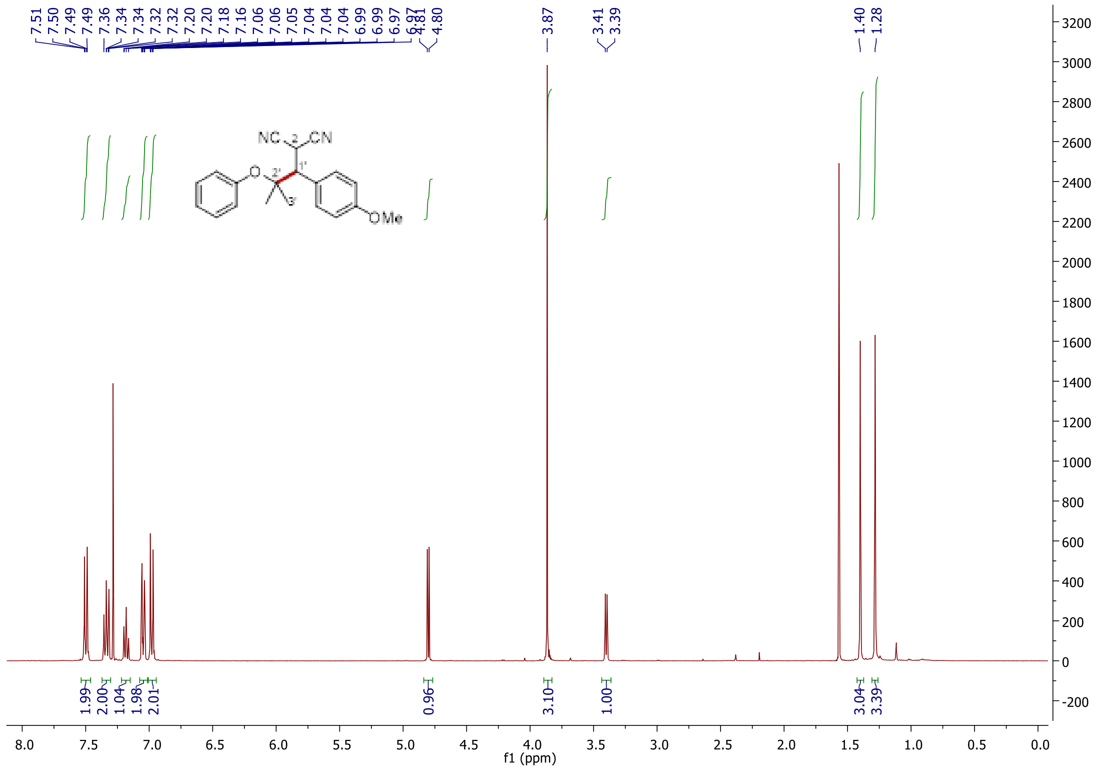

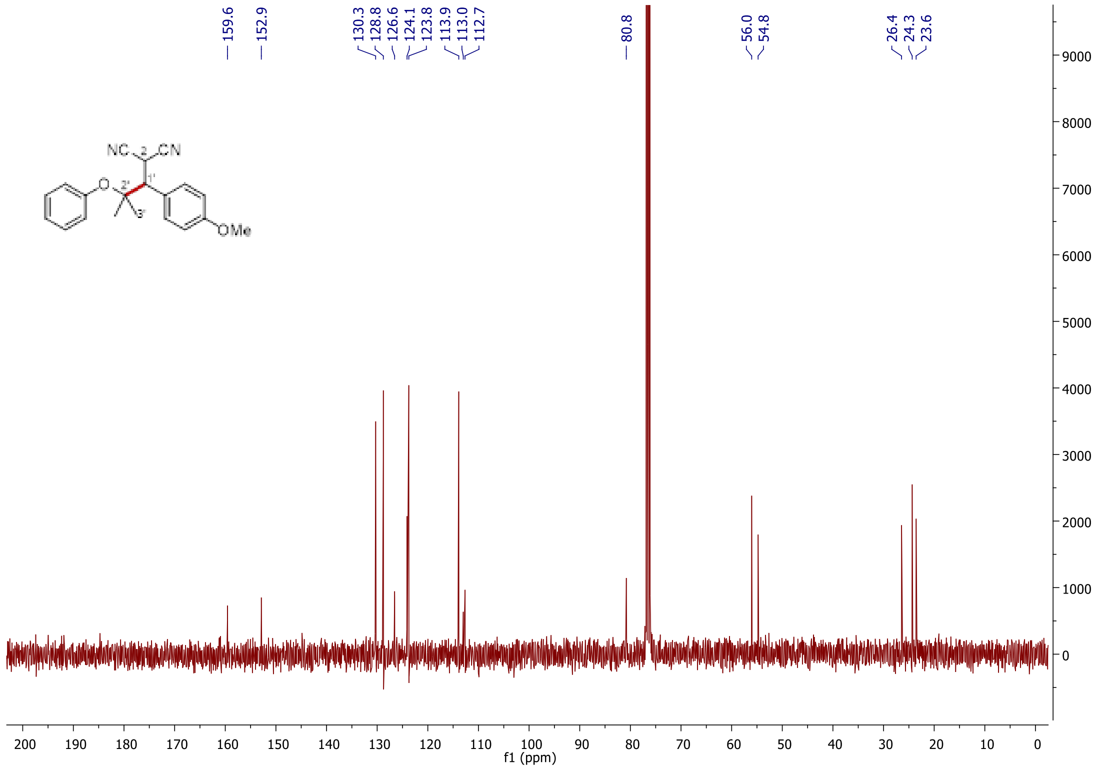

Tetraethyl 3-(phenoxy)propane-1,1-diphosphonate (23)

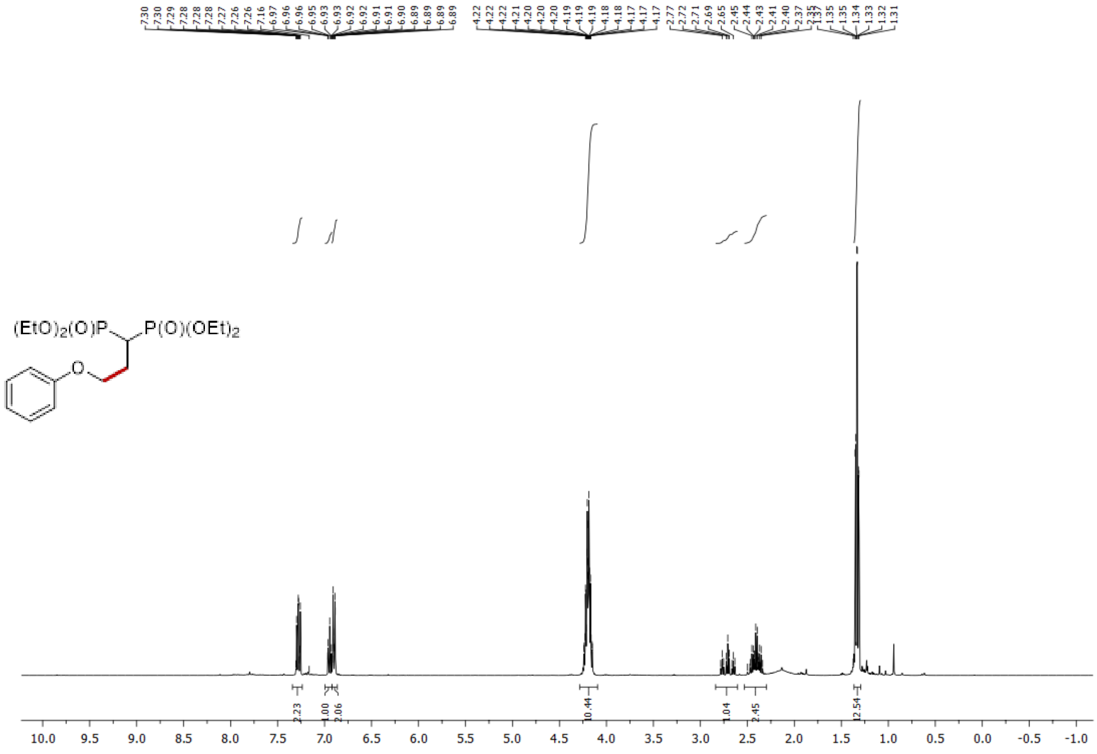

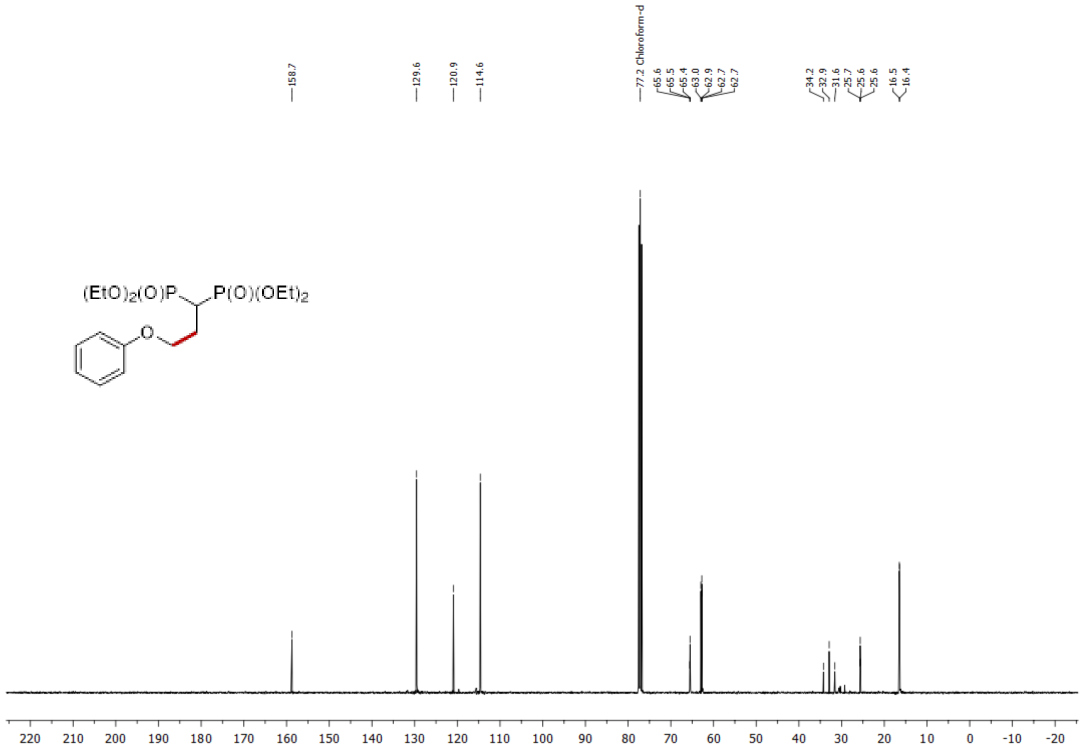

2-(2′-Phenoxyethyl)-1,3-diphenyl-1,3-dione (24)

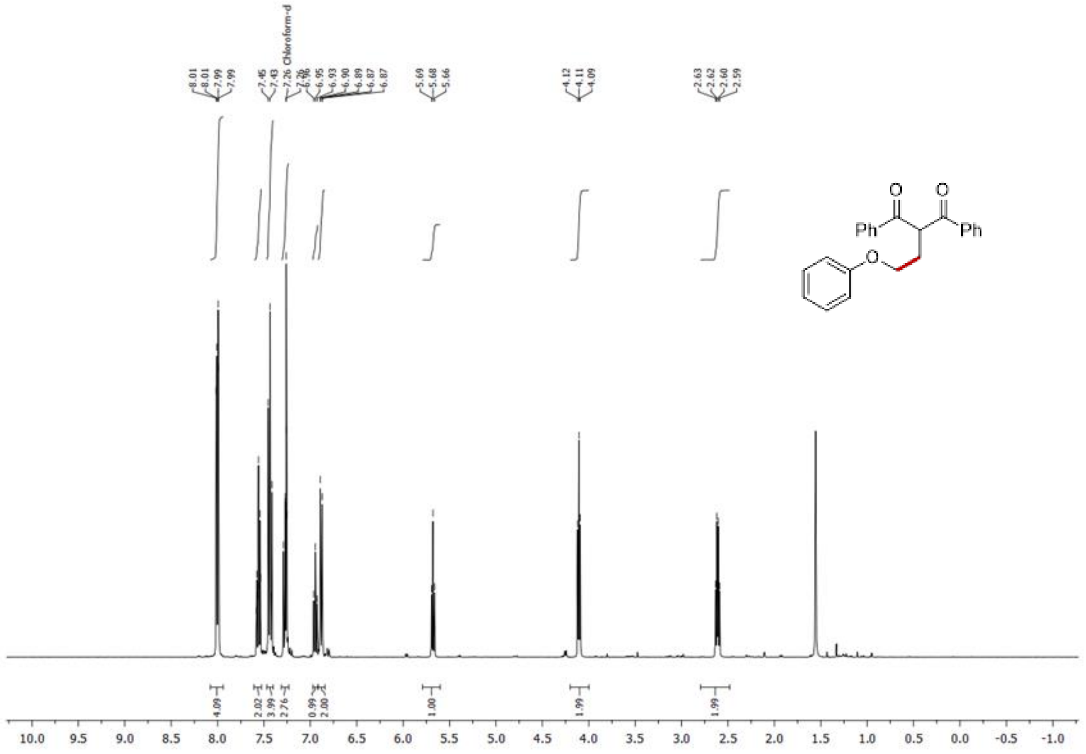

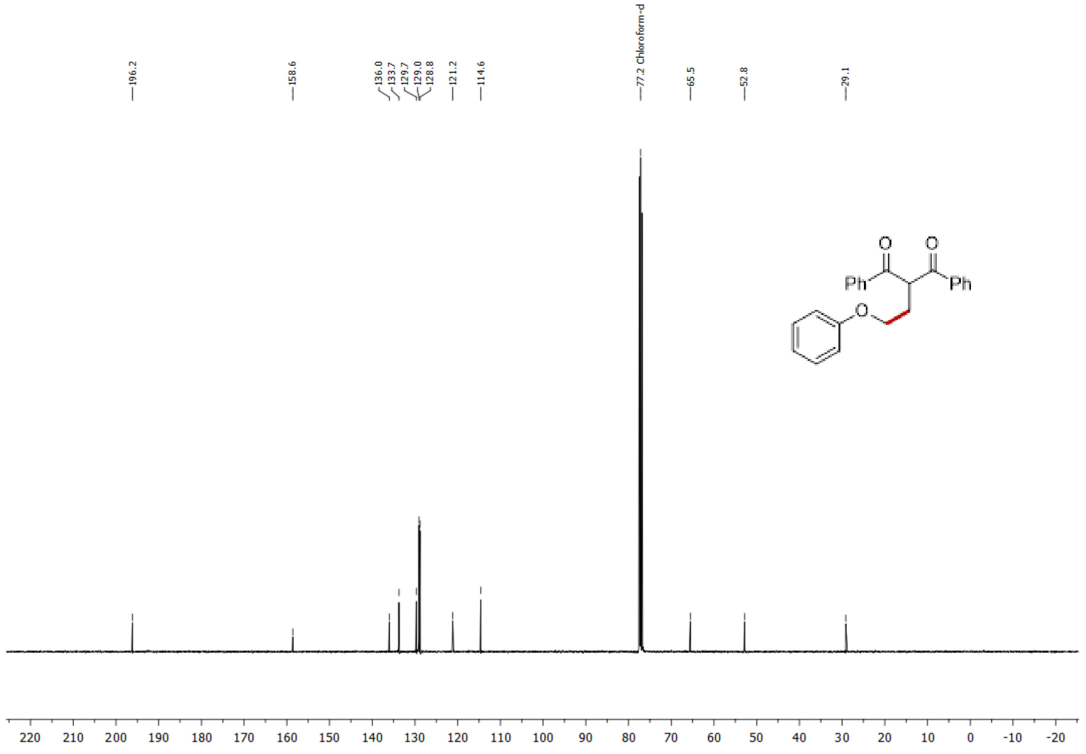

Diethyl 2-(1′-phenoxypropan-2′-yl)malonate (30)

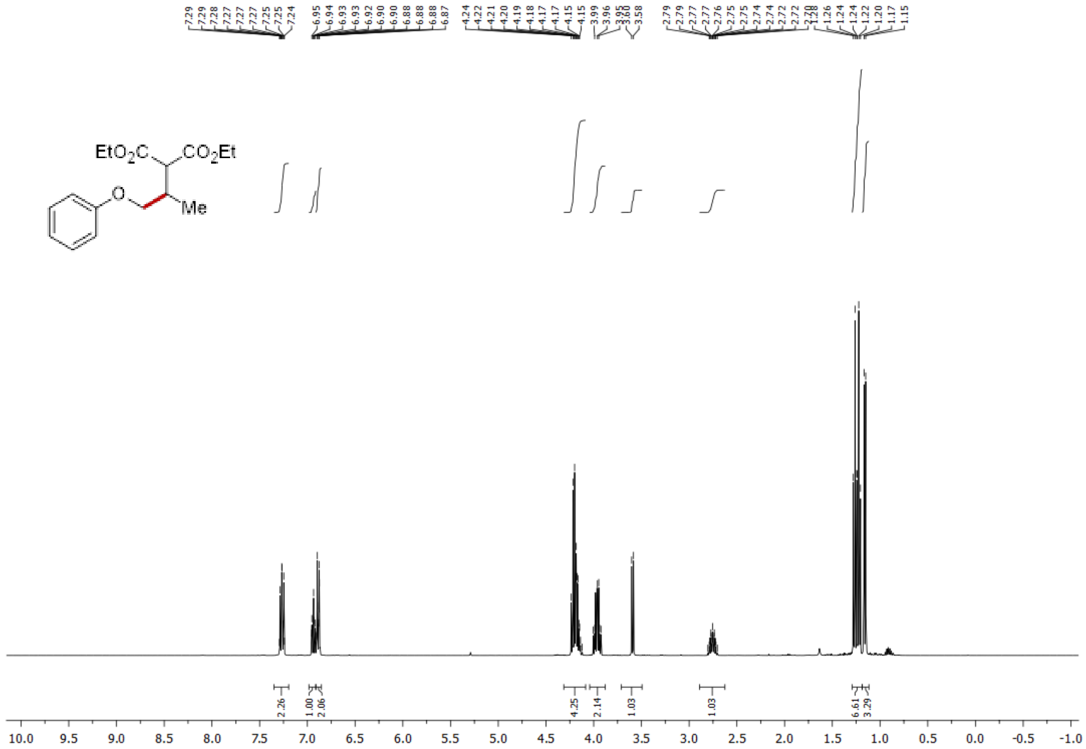

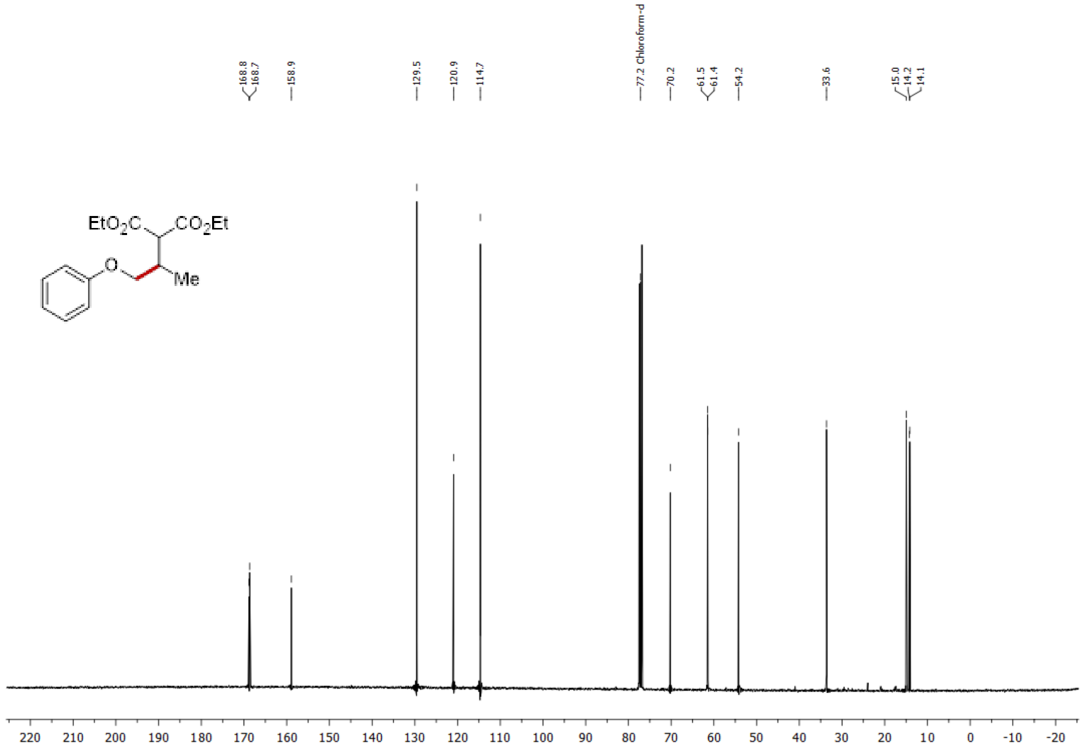

4-(3′-Methyl-3′-phenoxybutyl)pyridine (31a)

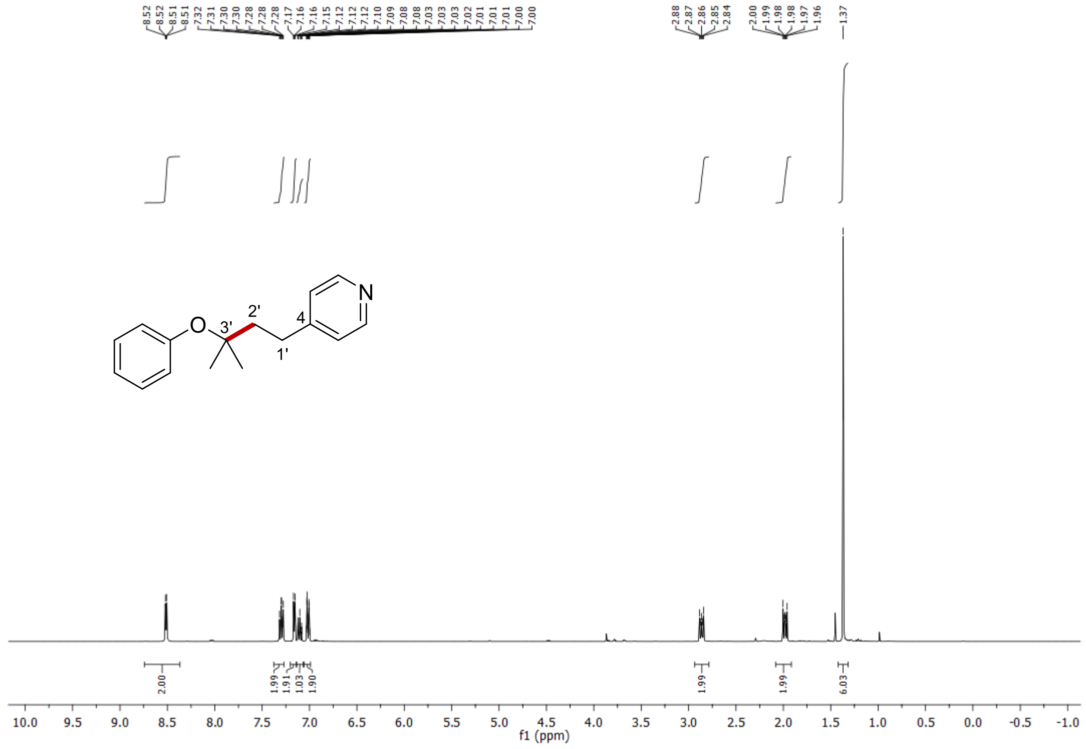

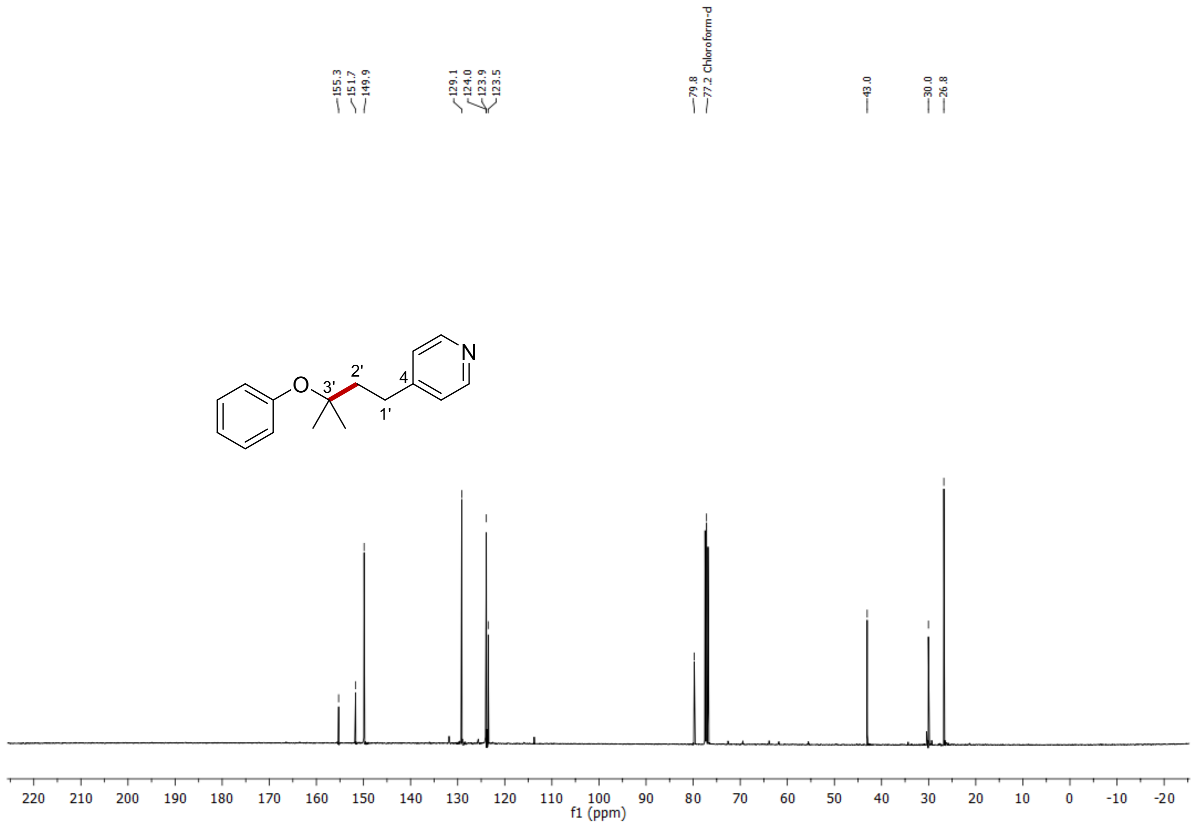

2-(3′-Methyl-3′-phenoxybutyl)pyridine (31b)

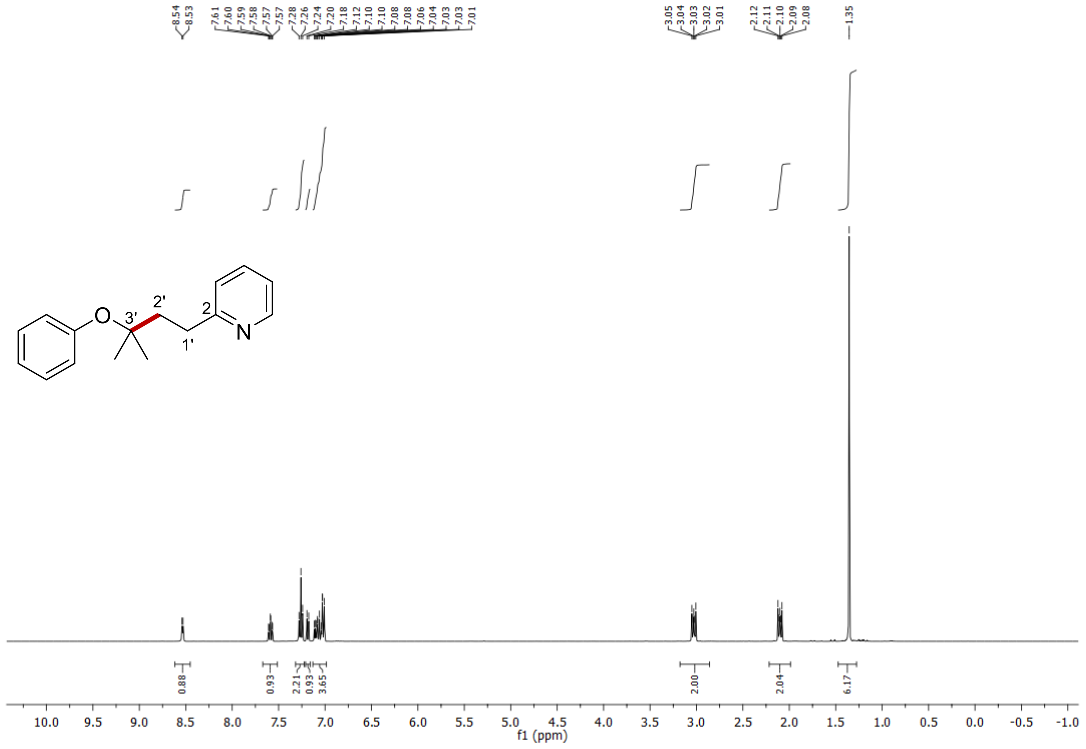

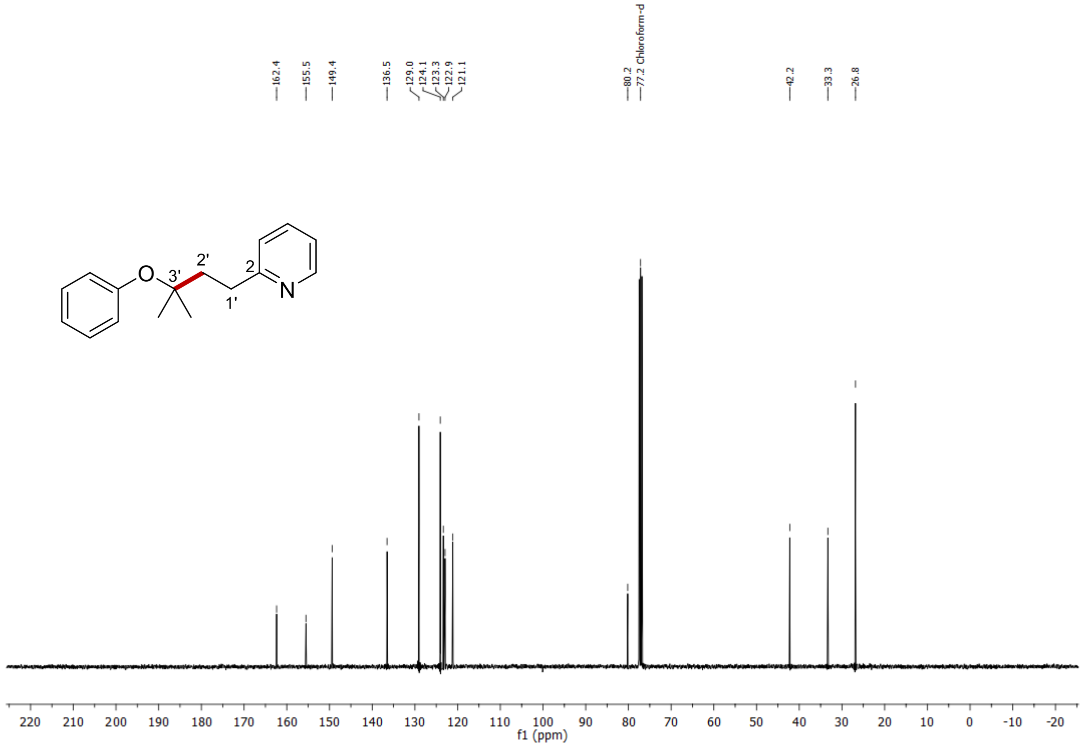

4-(2′-(1′′-Phenoxycyclobutyl)ethyl)pyridine (31c)

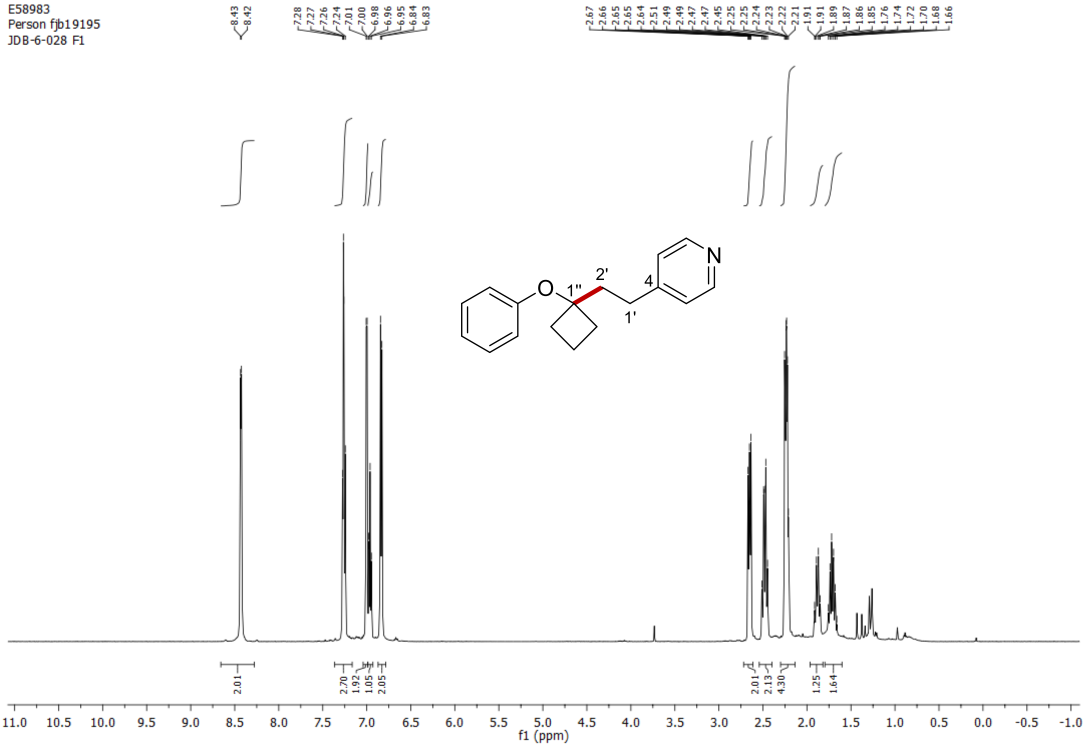

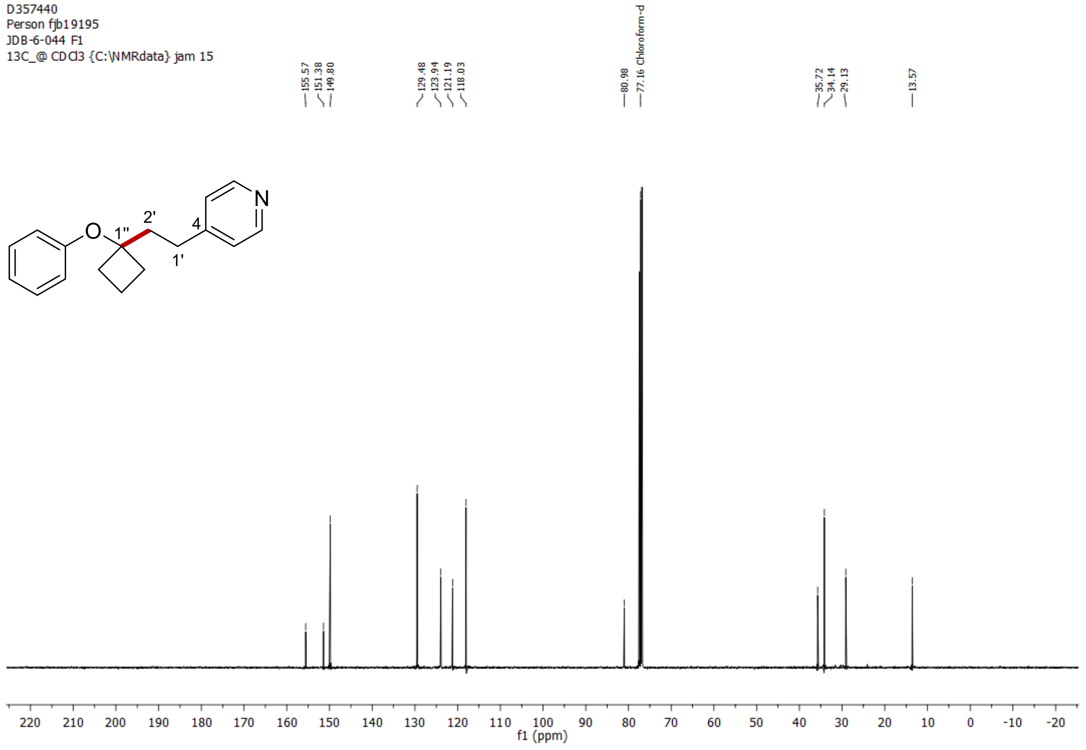

2-(2′-(1″-Phenoxycyclobutyl)ethyl)pyridine (31d)

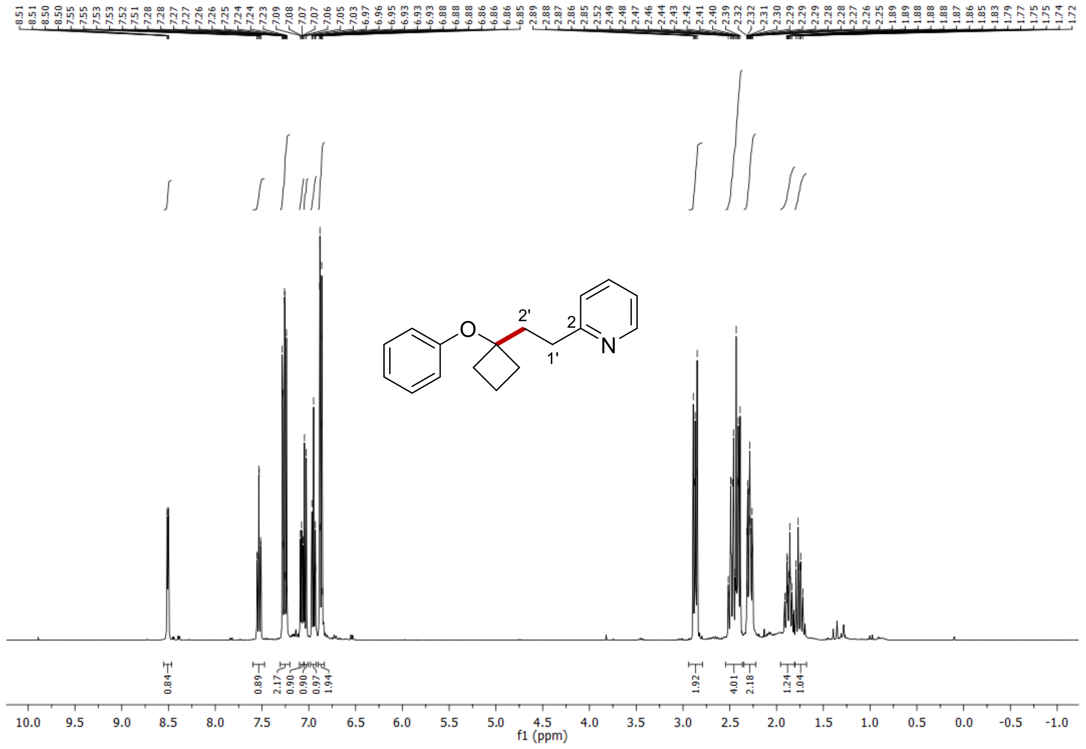

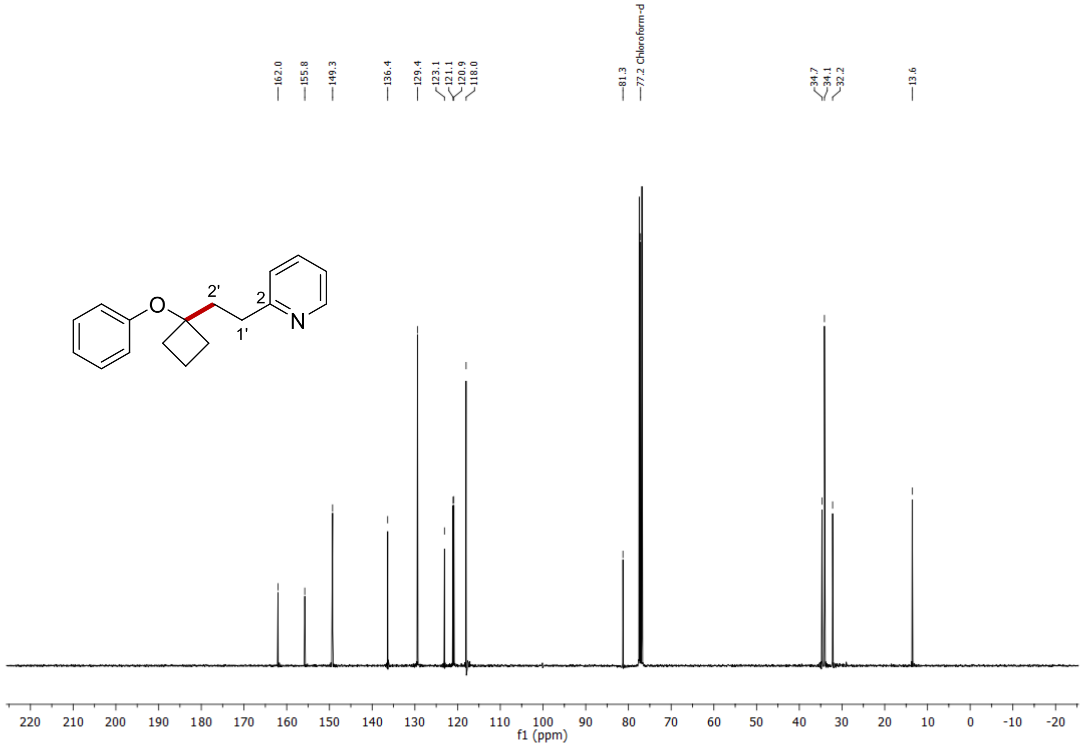

4-(2′-(1″-Phenoxycyclopentyl)ethyl)pyridine (31e)

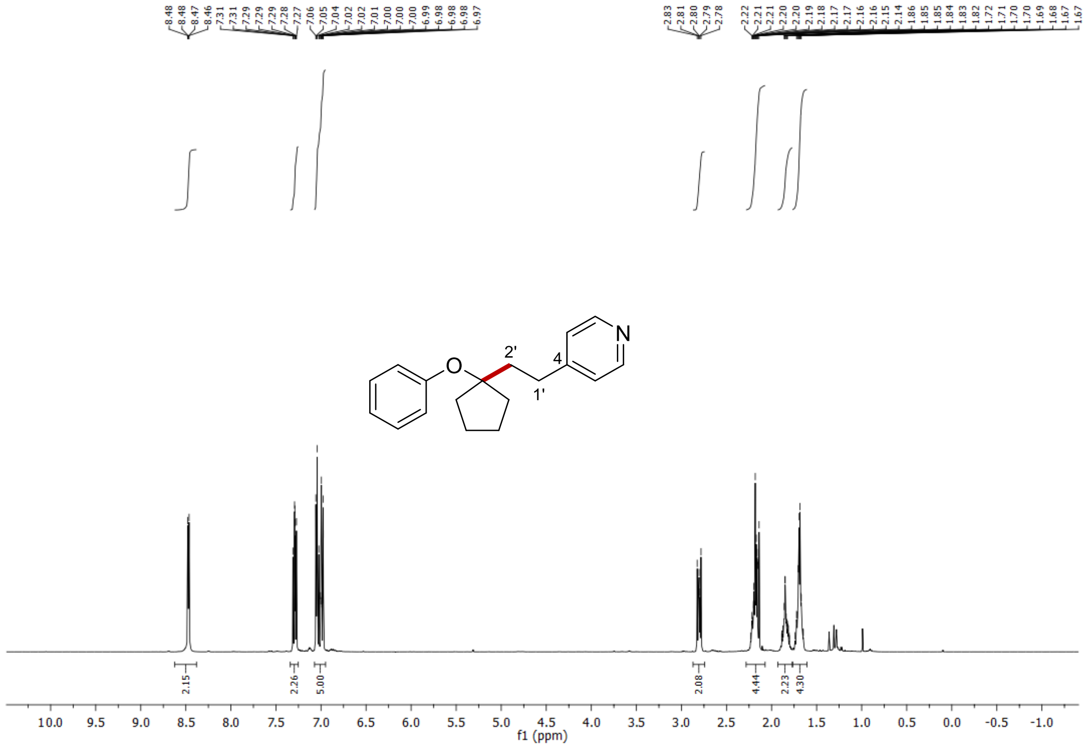

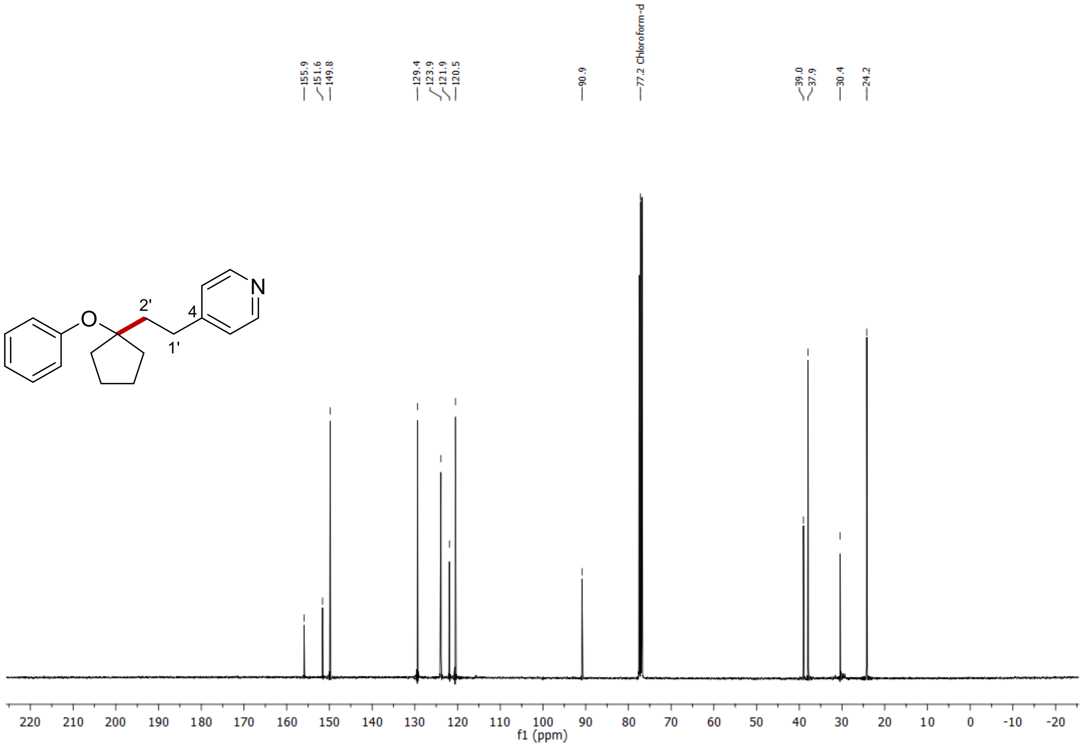

2-(2′-(1″-Phenoxycyclopentyl)ethyl)pyridine (31f)

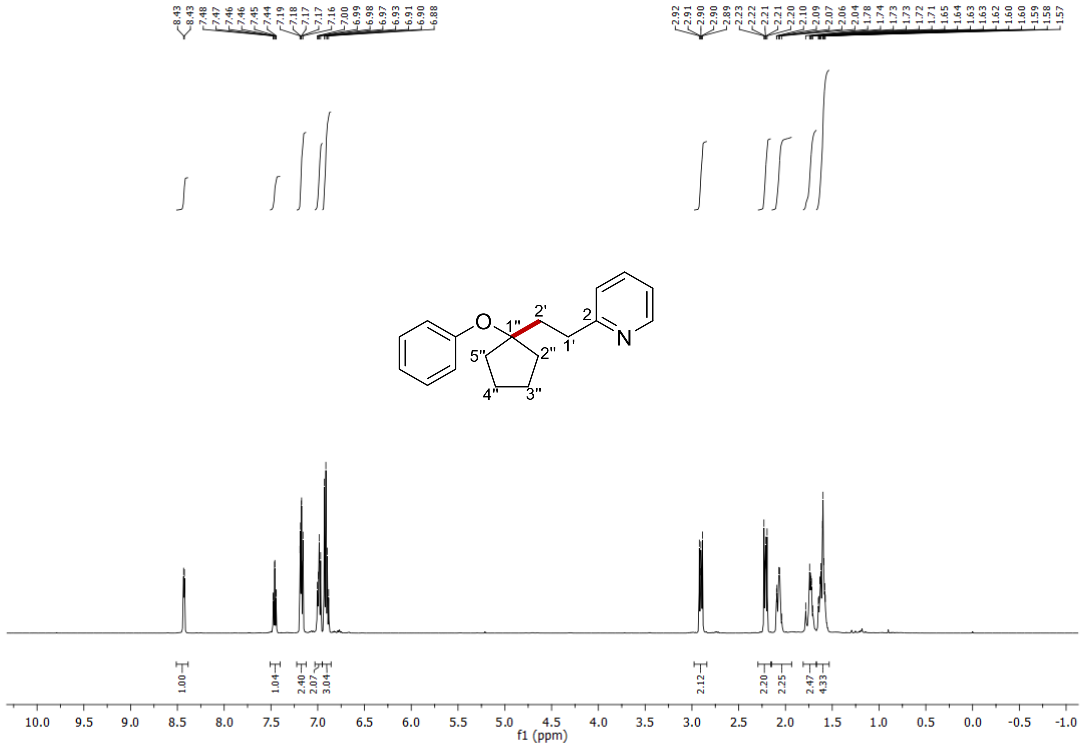

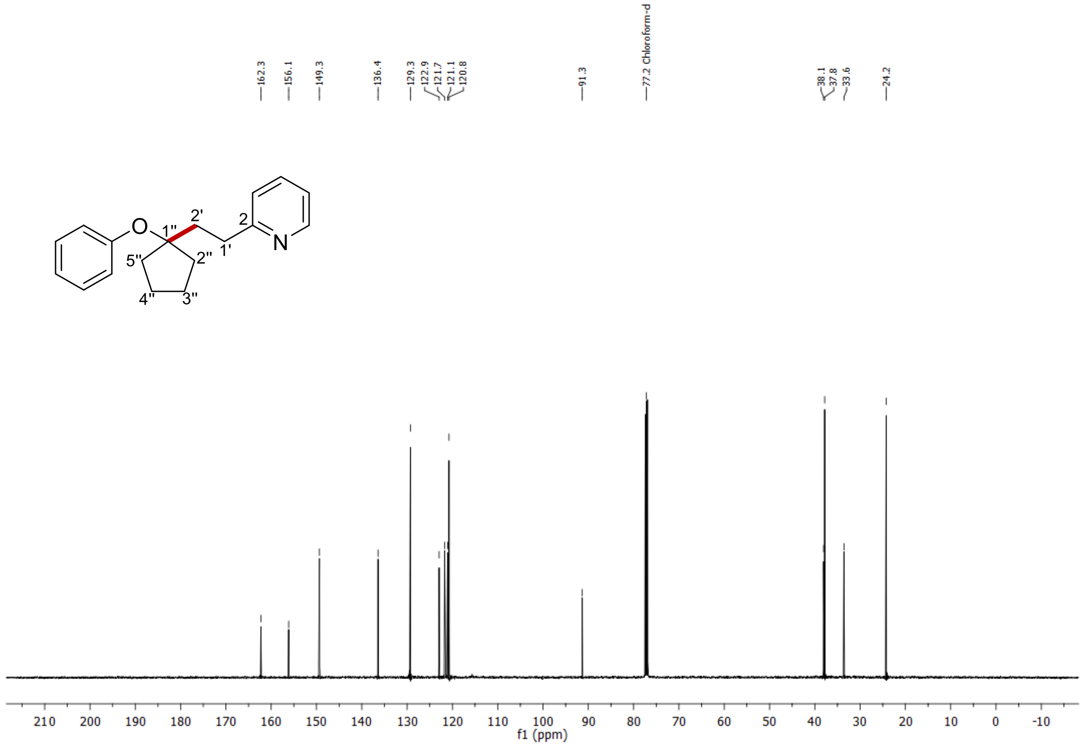

Methyl 4-phenoxybutanoate (S31)

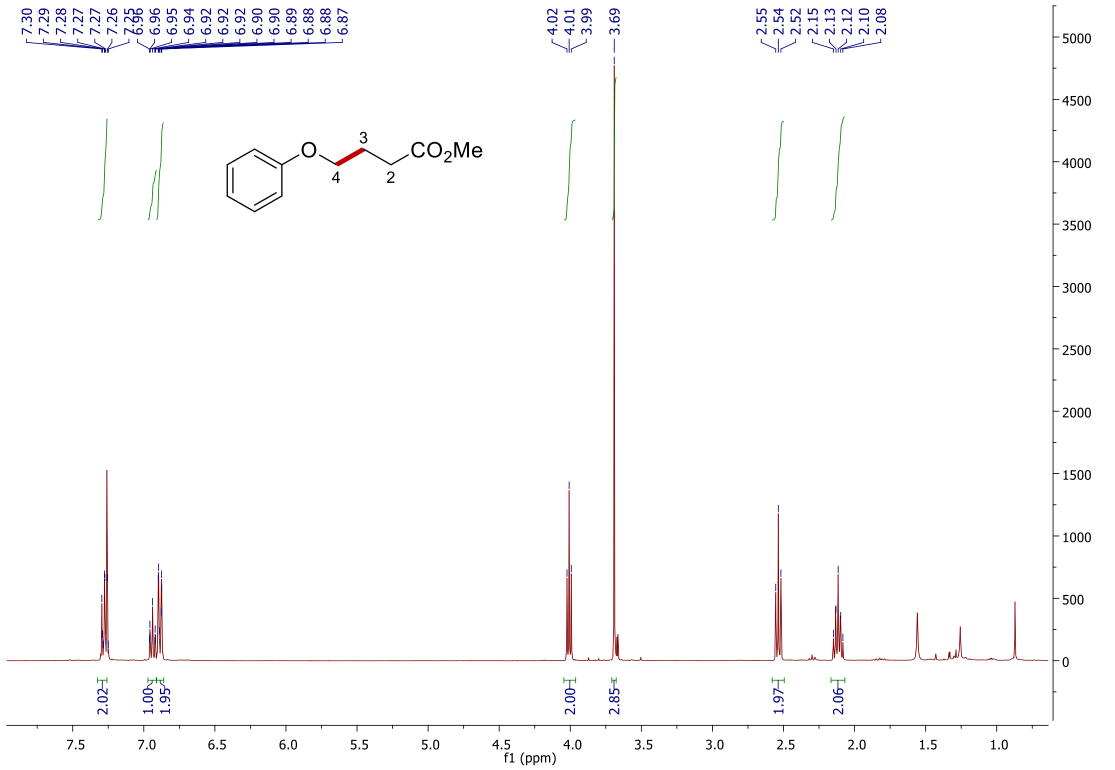

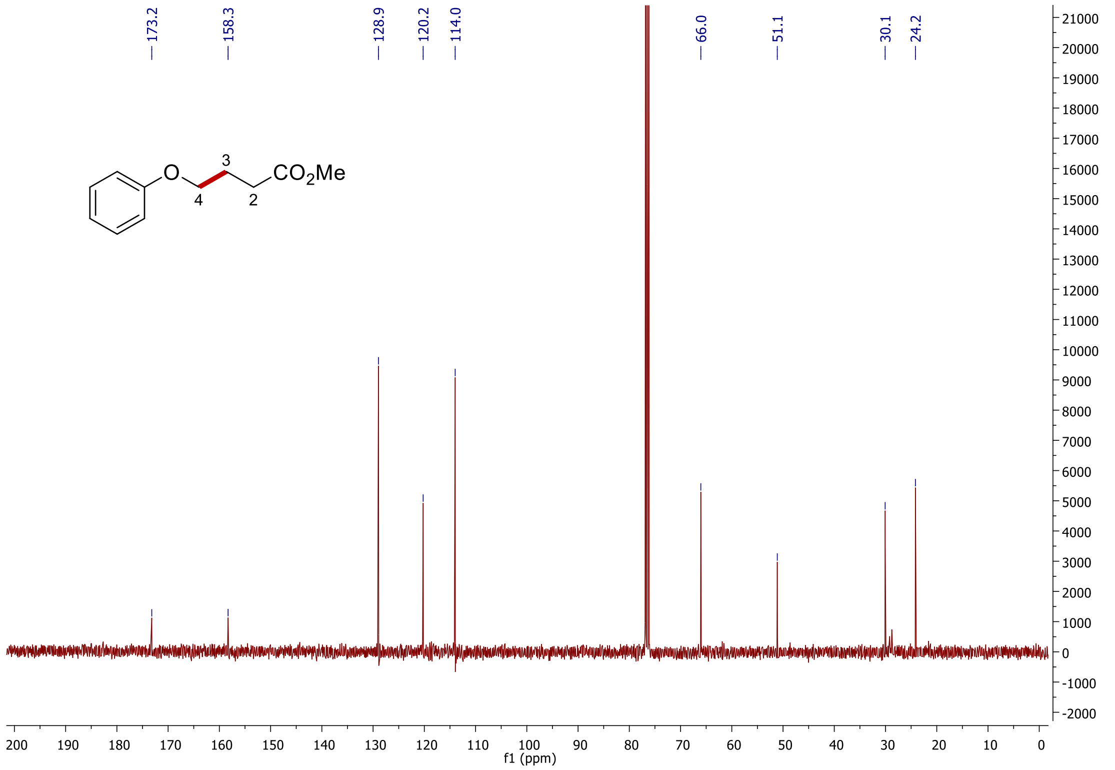

Methyl 4-methyl-4-phenoxypentanoate (S33)

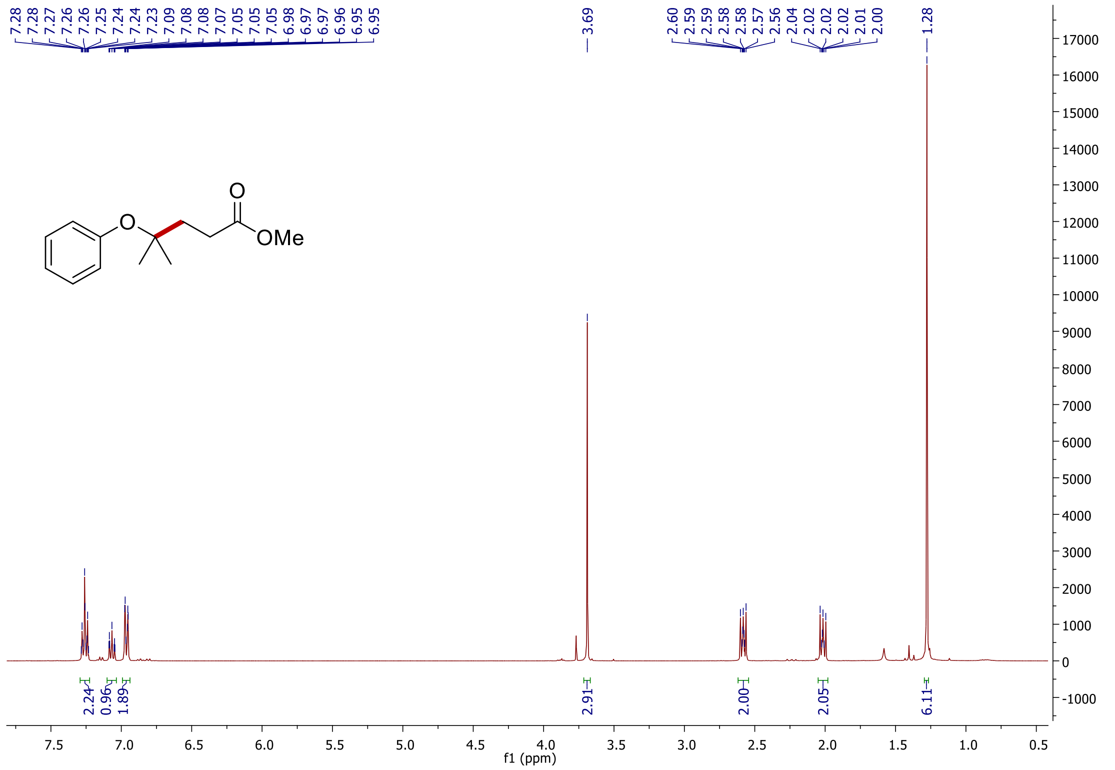

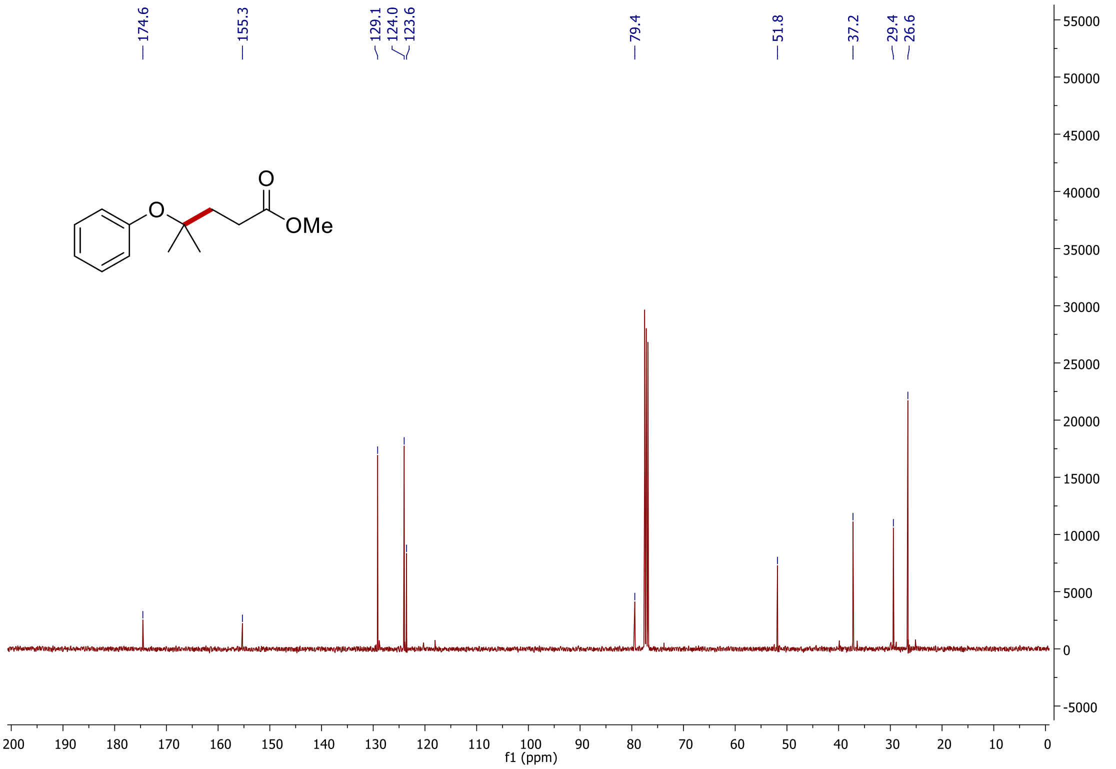

Methyl 4-methyl-4-phenoxypentanoate (S33) and methyl 2,2-dimethylchromane-4-carboxylate (S34)

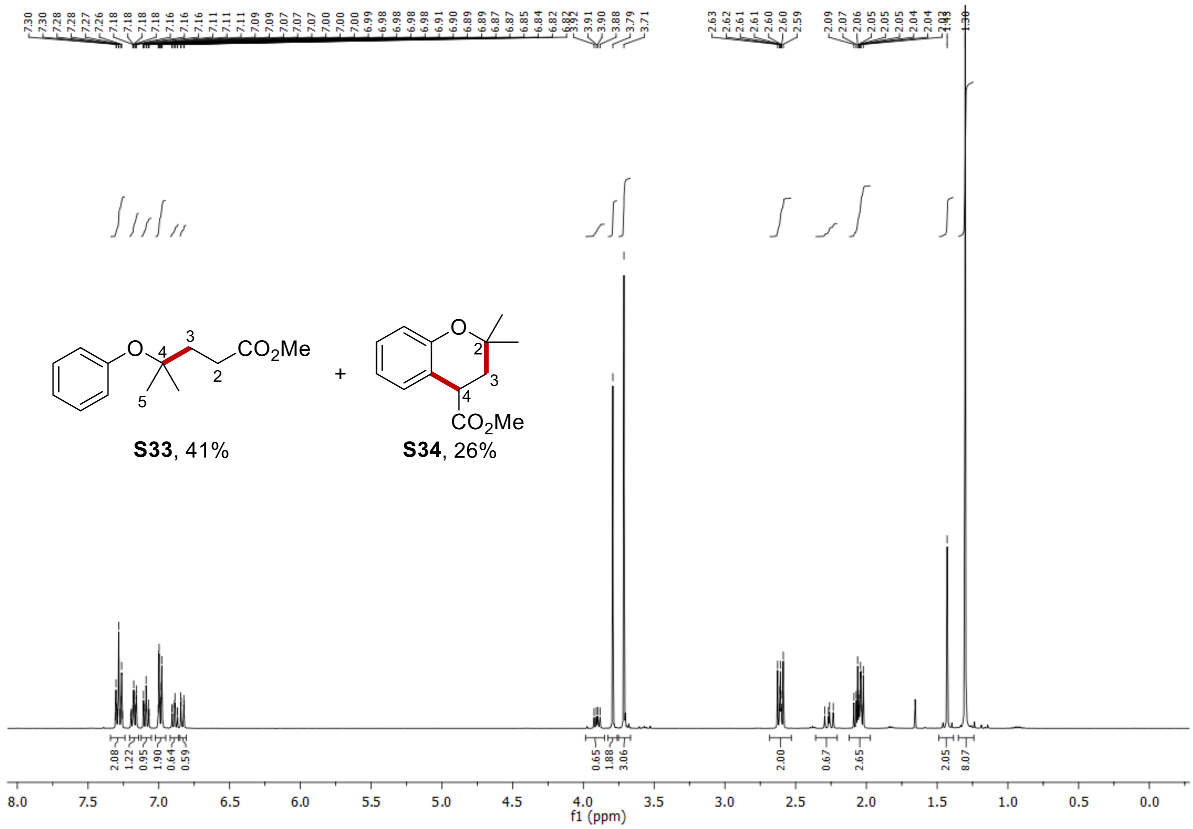

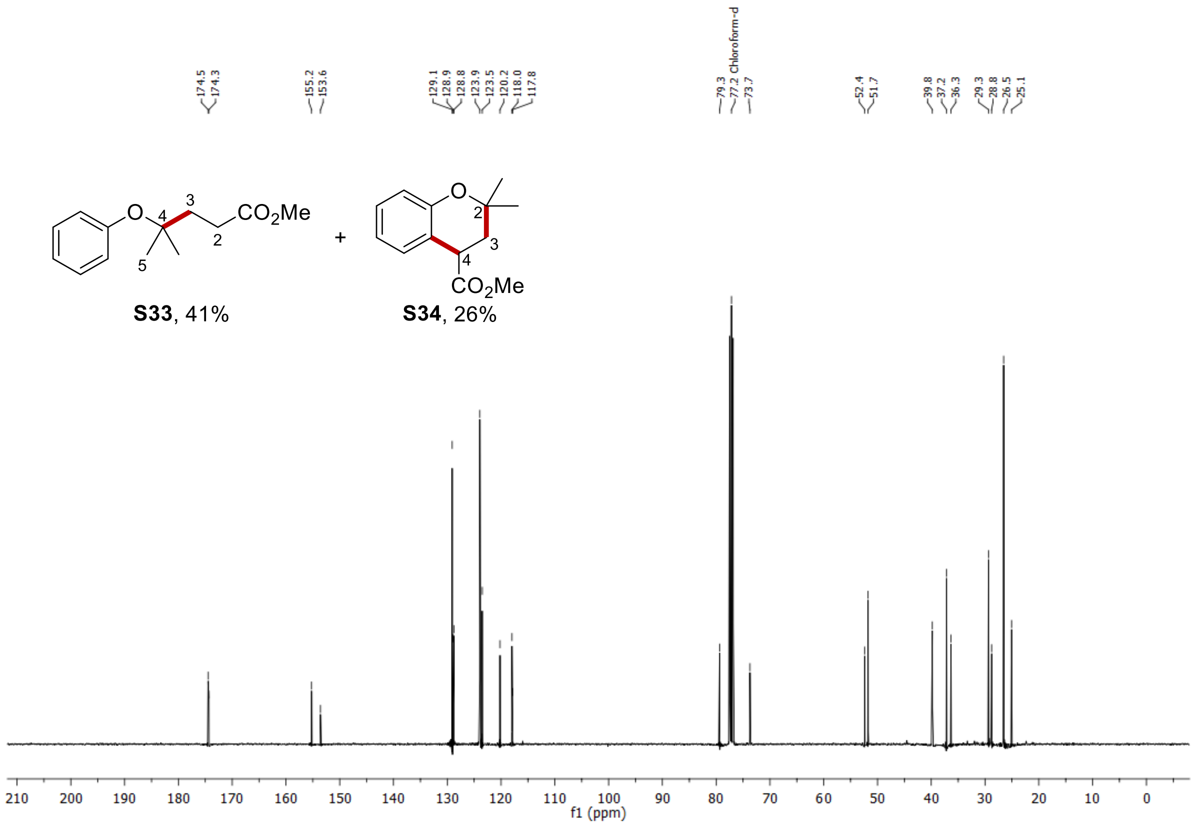

14. References

[1] S. M. Treacy and T. Rovis, *J. Am. Chem. Soc.*, 2021, **143**, 2729–2735.

[2] A. White, L. Wang and D. Nicewicz, *Synlett*, 2019, **30**, 827–832.

[3] Y.-M. Li, S.-J. Lou, Q.-H. Zhou, L.-W. Zhu, L.-F. Zhu and L. Li, *Eur. J. Org. Chem.*, 2015, 3044–3047.

[4] V. Kubíček, J. Kotek, P. Hermann and I. Lukeš, *Eur. J. Inorg. Chem.*, 2007, 333–344.

[5] A. P. Dieskau, M. S. Holzwarth and B. Plietker, *Chem. Eur. J.*, 2012, **18**, 2423–2429.

[6] N. Khatun, M. J. Kim and S. K. Woo, *Org. Lett.*, 2018, **20**, 6239–6243.

[7] F. V. Singh, S. R. Mangaonkar and P. B. Kole, *Synth. Commun*,. 2018, **48**, 2169–2176.

[8] D. A. R. Happer, S. M. McKerrow and A. L. Wilkinson, *Aust. J. Chem.*, 1977, **30**, 1715–1725.

[9] L. L. Santos, V. R. Ruiz, M. J. Sabater and A. Corma, *Tetrahedron*, 2008, **64**, 7902–7909.

[10] C. Matt, F. Kölblin and J. Streuff, *Org. Lett.*, 2019, **21**, 6983–6988.

[11] W. Zhao, R. P. Wurz, J. C. Peters and G. C. Fu, *J. Am. Chem. Soc.*, 2017, **139**, 12153–12156.

[12] X. Jiang, J. Zhou, Y. Zhou, H. Sun, J. Xu, F. Feng and W. Qu, *Molecules*, 2018, **23**, 1–14.

[13] L. Ren, M. M. Yang, C. H. Tung, L. Z. Wu and H. Cong, *ACS Catal.*, 2017, **7**, 8134–8138.

[14] I. Y. El-Deeb, M. Tian, T. Funakoshi, R. Matsubara and M. Hayashi, *Eur. J. Org. Chem.* 2017, 409–413.

[15] D. O. Ptashko, M. D. Khanova, V. A. Dokichev and Y. V. Tomilov, *Russ. J. Org. Chem.*, 2011, **47**, 1498–1500.

[16] Y. F. Liang, Y. Yuan, T. Shen, S. Song, and N. Jiao, *Chin. J. Chem.*, 2018, **36**, 233–240.

[17] S. S. Hanson, E. Doni, K. T. Traboulsee, G. Coulthard, J. A. Murphy and C. A. Dyker, *Angew. Chem. Int. Ed.*, 2015, **54**, 11236–11239.

[18] H. Wang, Y. Ma, H. Tian, A. Yu, J. Chang and Y. Wu, *Tetrahedron*, 2014, **70**, 2669–2673.

[19] G. A. Russell and R. C. Williamson, *J. Am. Chem. Soc.*, 1964, **86**, 2357–2364.

[20] G. J. Bodwell, J. Li and D. O. Miller, *Tetrahedron*, 1999, **55**, 12939–12956.

[21] S. Rohe, A. O. Morris, T. McCallum and L. Barriault, *Angew. Chem. Int. Ed.*, 2018, **57**, 15664–15669.

[22] F. El-Hage, C. Schöll and J. Pospech, *J. Org. Chem.*, 2020, **85**, 13853–13867.

[23] M. Ueda, E. Kondoh, Y. Ito, H. Shono, M. Kakiuchi, Y. Ichii, T. Kimura, T. Miyoshi, T. Naito and O. Miyata, *Org. Biomol. Chem.*, 2011, **9**, 2062–2064.

[24] H.-P. Deng, Q. Zhou and J. Wu, *Angew. Chem. Int. Ed.*, 2018, **57,** 12661–12665.

[25] G. Feng, X. Wang and J. Jin, *Eur. J. Org. Chem.*, 2019, 6728–6732.

[26] N. P. Ramirez and J. C. Gonzalez-Gomez, *Eur. J. Org. Chem.*, 2017, 2154–2163.

[27] L. K. Fraiji, D. M. Hayes and T. C. Werner, *J. Chem. Educ.*, 1992, **69**, 424.

[28] M. A. Cismesia and T. P. Yoon, *Chem. Sci.*, 2015, **6**, 5426–5434.

[29] C. G. Hatchard and C. A. Parker, *Proc. Roy. Soc. (London)*, 1956, **A23**, 518–536

[30] R. A. Garza-Sanchez, A. Tlahuext-Aca, G. Tavakoli and F. Glorius, *ACS Catal.*, 2017, **7**, 4057–4061.

[31] J. P. Phelan, S. B. Lang, J. S. Compton, C. B. Kelly, R. Dykstra, O. Gutierrez and G. A. Molander, *J. Am. Chem. Soc.*, 2018, **140**, 8037–8047.

[32] B. Wriedt and D. Ziegenbalg, *ChemPhotoChem*, 2021, **5**, 947–956.

[33] B. Vandekerckhove, N. Piens, B. Metten, C. V. Stevens and T. S. A. Heugebaert, *Org. Process Res. Dev.*, 2022, **26**, 2392–2402.

[34] B. Wriedt and D. Ziegenbalg, *J. Flow Chem.*, 2020, **10**, 295–306.

[35] W. D. Bowman and J. N. Demas, *J. Phys. Chem.*, 1976, **80**, 2434–2435.

[36] E. E. Wegner and A. W. Adamson, *J. Am. Chem. Soc.*, 1966, **88**, 394–404.
